# Synthesis of Mixed Phosphonate Esters and Amino Acid-Based Phosphonamidates, and Their Screening as Herbicides

**DOI:** 10.3390/ijms25094739

**Published:** 2024-04-26

**Authors:** Simon Backx, Willem Desmedt, Andreas Dejaegere, Andreas Simoens, Jef Van de Poel, Dorota Krasowska, Kris Audenaert, Christian V. Stevens, Sven Mangelinckx

**Affiliations:** 1 SynBioC Research Group, Department of Green Chemistry and Technology, Faculty of Bioscience Engineering, Ghent University, Coupure Links 653, 9000 Ghent, Belgium; simon.backx@ugent.be (S.B.); andreas.dejaegere@ugent.be (A.D.); andreas.simoens@ugent.be (A.S.); jef.vandepoel@oleon.com (J.V.d.P.); dorota.krasowska@cbmm.lodz.pl (D.K.); chris.stevens@ugent.be (C.V.S.); 2 Laboratory of Applied Mycology and Phenomics, Department of Plants and Crops, Faculty of Bioscience Engineering, Ghent University, Valentin Vaerwyckweg 1, 9000 Ghent, Belgium; willem.desmedt@ilvo.vlaanderen.be (W.D.); kris.audenaert@ugent.be (K.A.); 3 Plant Sciences Unit, Flanders Research Institute for Agriculture, Fisheries and Food (ILVO), Burg. van Gansberghelaan 96, 9820 Merelbeke, Belgium

**Keywords:** phosphorus chemistry, phosphonamidates, amino acids, AHLs, herbicides

## Abstract

While organophosphorus chemistry is gaining attention in a variety of fields, the synthesis of the phosphorus derivatives of amino acids remains a challenging task. Previously reported methods require the deprotonation of the nucleophile, complex reagents or hydrolysis of the phosphonate ester. In this paper, we demonstrate how to avoid these issues by employing phosphonylaminium salts for the synthesis of novel mixed *n*-alkylphosphonate diesters or amino acid-derived *n*-alkylphosphonamidates. We successfully applied this methodology for the synthesis of novel *N*-acyl homoserine lactone analogues with varying alkyl chains and ester groups in the phosphorus moiety. Finally, we developed a rapid, quantitative and high-throughput bioassay to screen a selection of these compounds for their herbicidal activity. Together, these results will aid future research in phosphorus chemistry, agrochemistry and the synthesis of bioactive targets.

## 1. Introduction

**Scheme 1:**
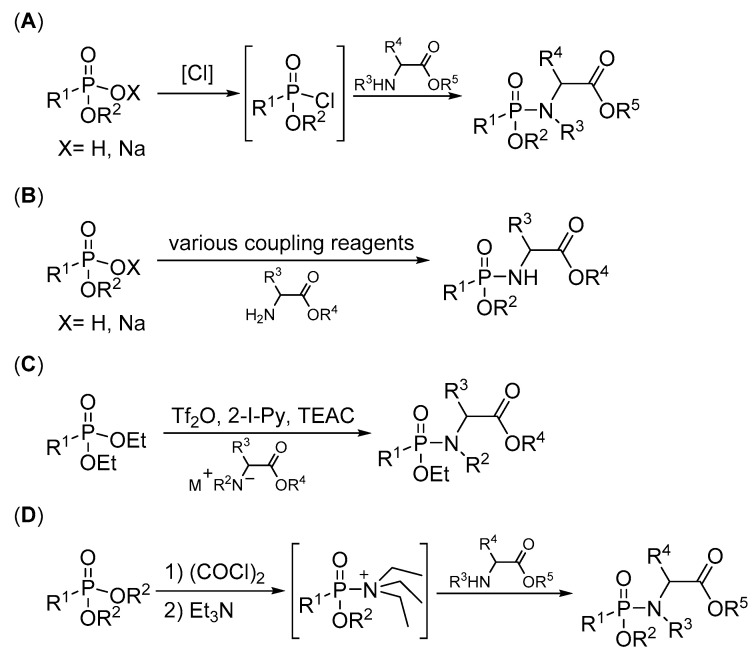
The scheme shows an overview of the published methodologies for the synthesis of amino acid-based phosphonamidates. These moieties can be synthesized via (**A**) the classical approach [2,3,4,5,6,7], (**B**) a direct coupling method [8,9,10,11,12], (**C**) triflic anhydride activation [13] or (**D**) our methodology [14].

The synthesis of phosphorus derivatives comprising amino acid moieties in targeted bioactive molecules can be a challenging task. In the case of Staudinger-phosphonite synthesis, the necessary phosphonite and/or azide reagents need to be synthesized in advance, which comes with inherent safety risks and/or synthetic difficulties. Alternatives, such as the Atherton–Todd reaction, usually share these disadvantages [1]. Therefore, examples of the synthesis of amino acid-based phosphonamidates in the literature are mostly based on the so-called ‘classical’ approach, consisting of the monochlorination of a phosphonate ester, which is available through a Michaelis–Arbuzov reaction or Hirao coupling, followed by substitution with the desired nucleophile. To achieve the monochlorination of the phosphorus centre, almost all reports first convert the symmetrical diester to the monoester, which is then chlorinated and substituted with the desired moiety (Scheme 1A) [2,3,4,5,6,7] or coupled with the target amino acid by the use of more complex coupling reagents (Scheme 1B) [8,9,10,11,12]. The only exception in terms of the use of amino acids is a procedure reported by the Maulide group, which achieves this transformation directly from diethyl phosphonates. However, this method requires the deprotonation of the used nucleophiles and is only demonstrated on *N*-tosyl or secondary amino acid esters (Scheme 1C) [13].

In a recent paper [14], we have demonstrated the use of phosphonylaminium salts for the synthesis of phosphonamidates without the need for harsh or expensive reagents while still ensuring that the protocol remains widely applicable. In this paper, we expand the application scope of these salts to reactions with phenols and amino acids, leading to a series of mixed *n*-alkylphosphonate diesters or *n*-alkylphosphonamidates, respectively (Scheme 1D, only shown for phosphonamidates). To the best of our knowledge, this is the first report of amino acid-based *n*-alkylphosphonamidates being directly synthesized from symmetrical phosphonate diesters without deprotonation of the targeted nucleophiles.

To demonstrate the potential of our methodology, we synthesized a library of phosphonamidate analogues of *N*-acyl homoserine lactones (AHLs) that had not been described in the literature before. These novel compounds may prove useful for the study of quorum sensing activation and inhibition. We were also intrigued by a patent describing *n*-alkylphosphonamidates as possible herbicides [15]. This patent describes the synthesis of a variety of *n*-alkylphosphonamidates, mainly based on dialkylamines, of which several–particularly di-*n*-propylamine derivatives–show significant herbicidal activity.

We wanted to use our library of phosphonamidates to further explore this herbicidal effect by testing the influence of different residues on this bioactivity. In order to undertake this rapidly, quantitatively and with minimal amounts of compound (to reduce the environmental impact of our experiments), a high-throughput bioassay based on chlorophyll fluorescence as a proxy for plant health in tomato (*Solanum lycopersicum* L.) leaf disks was developed. We used this bioassay to evaluate the herbicidal properties of the obtained compounds. A wide range of activity levels was observed, which confirms the utility of these functionalized phosphonamidates and provides a promising foundation for conducting more comprehensive structure–activity relationship studies.

## 2. Results and Discussion

### 2.1. Synthesis of Novel Phosphonyl Moieties

Recently, we described the use of phosphonylaminium salts as phosphonylating agents with good results in combination with amines and anilines [14]. An interesting expansion of this work would be in the direction of phenols and toward the formation of mixed *n*-alkylphosphonate diesters. The inclusion of arylesters (e.g., in the well-known tenofovir alafenamide) on a phosphorus moiety rarely happens directly [16], as phenolic phosphonate esters cannot be chlorinated with oxalyl chloride-type chlorinations nor can these arylesters be included via the Michaelis–Arbuzov reaction [17]. Therefore, expanding our reactivity to phenolic substrates would be very complimentary to our method.

**Scheme 2:**
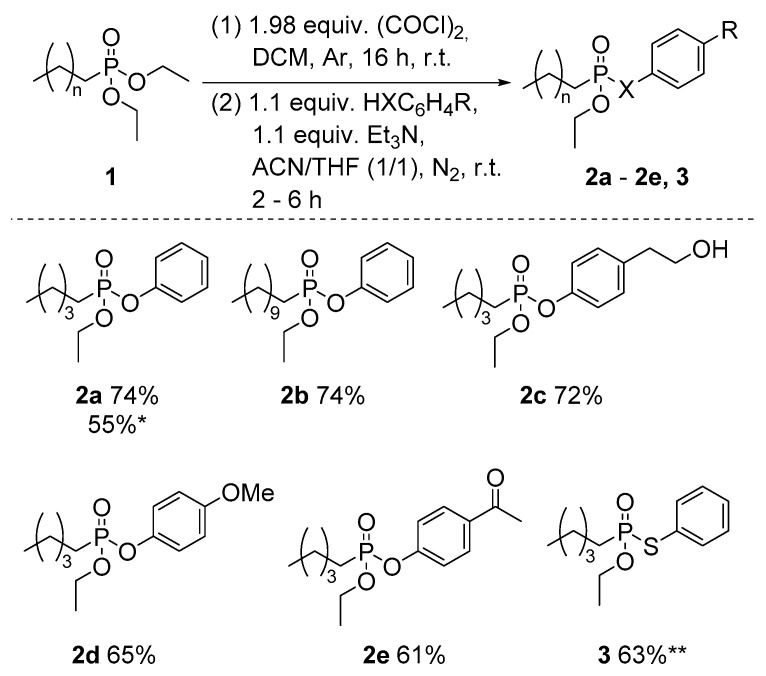
The scheme shows the synthesis of mixed phosphonate diesters (**2a**–**2e** and **3**) with various phenols, starting from symmetrical diethyl *n*-alkylphosphonates **1**. * Reaction performed at 36.8 mmol scale, instead of 3.7 mmol. ** Synthesis performed with 2.64 equiv. oxalyl chloride.

In contrast to the phosphonylation of anilines, which could only be achieved at higher temperatures, phenol could be coupled at room temperature with both diethyl pentylphosphonate **1a** and diethyl undecylphosphonate **1b**, yielding the desired mixed phosphonate diesters **2a** and **2b** in 74% yield (Scheme 2). Interestingly, a chemoselectivity test on tyrosol showed almost exclusive phosphonylation of the phenolic OH, and the corresponding product **2c** could be isolated with a 72% yield. Electron donating or withdrawing groups on the phenolic substrate were tolerated, with products **2d** and **2e** being isolated in 65% and 61% yield, respectively. Lastly, a similar reaction with thiophenol afforded the target phosphonothioate **3** in a final yield of 63%. Applying this method for the synthesis of ethyl phenyl pentylphosphonate **2a** on a tenfold larger scale of 36.8 mmol led to the isolation of 5.2 g (55%) of phosphonate **2a**, indicating that our method can also be scaled up for the synthesis of larger amounts of products.

**Scheme 3:**
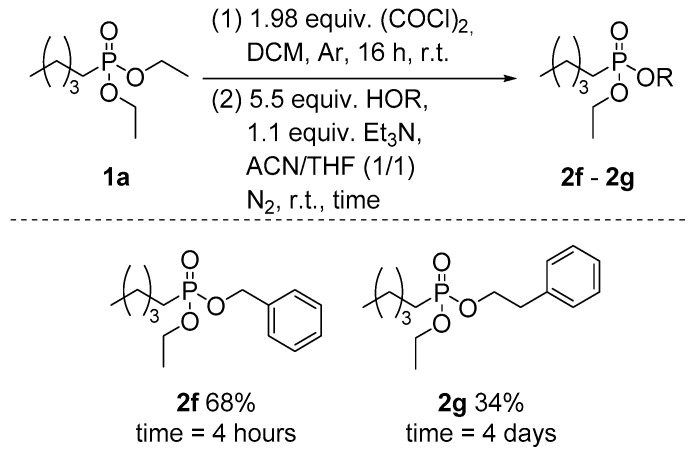
The scheme shows the synthesis of mixed phosphonate diesters **2f**–**2g**, starting from symmetrical diethyl pentylphosphonate **1a**.

As exemplified by the experiment with tyrosol, extending this phosphonylation methodology to alcoholic nucleophiles is not straightforward. A five-fold increase in the desired alcohol was necessary to mitigate competitive hydrolysis reactions. For benzyl alcohol, the desired product **2f** could be isolated in a 68% yield, whereas the reaction with phenethyl alcohol took much longer to complete and only yielded product **2g** in a 34% yield (Scheme 3). Since this last product required two chromatographic separations and a large excess of target alcohol, we decided not to further investigate the use of alcohols as substrates.

**Scheme 4:**
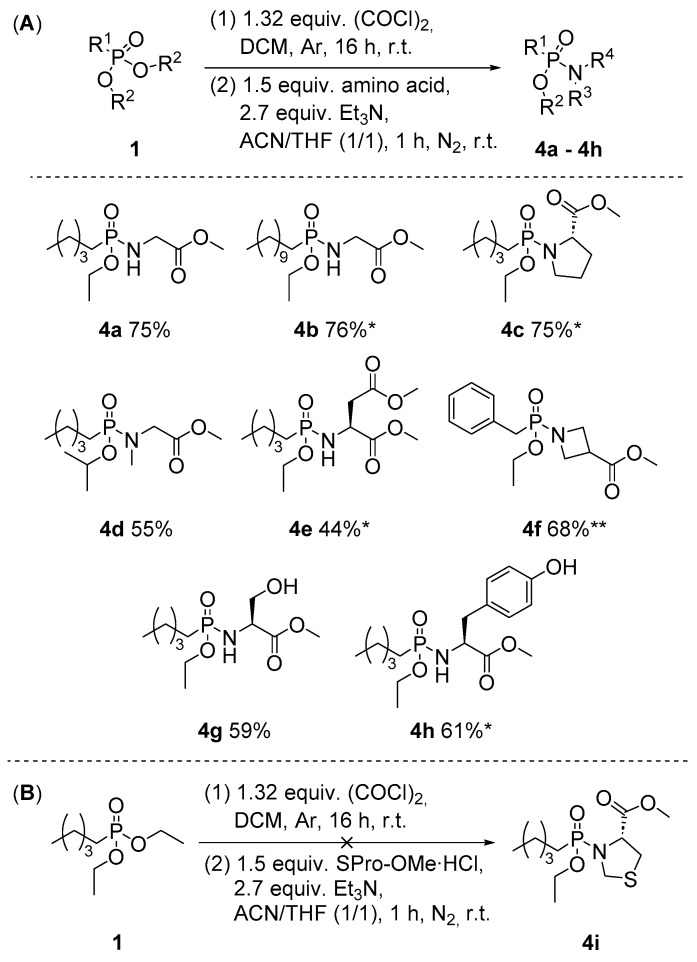
The scheme shows the (**A**) synthesis of amino acid–based phosphonamidates (**4a**–**4h**) from various symmetrical dialkyl alkylphosphonates. (**B**) Attempted route towards the use of thiaproline derivative for product **4i**. * Synthesis performed with 1.98 equiv. oxalyl chloride. ** Chlorination performed with a catalytic amount of DMF.

To enable similar transformations toward novel amino acid-based *n*-alkylphosphonamidates, a slight adaptation of the aforementioned procedure is necessary. Free amino acids are virtually insoluble in organic solvents, so we opted to use the widely available methyl ester hydrochlorides instead. As such, these hydrochloride salts are not much better in terms of solubility, but after neutralization with an organic base, such as triethylamine, the free amino ester readily dissolves. These amino esters are either commercially available or they can be synthesized from the native amino acid via a simple one-pot reaction in the desired alcoholic solvent [18]. Starting with the simplest amino acid derivative, glycine methyl ester was swiftly converted to the corresponding products **4a** and **4b** in 75% and 76% yields, respectively (Scheme 4A). Secondary amino acid derivatives and different ester groups on the phosphonate starting product were also tolerated with final yields of 75% (**4c**) and 55% (**4d**), respectively. Similar reactions with L-aspartic dimethyl ester and methyl azetidine-3-carboxylate also led to the isolation of the corresponding products (**4e** and **4f**, respectively) in moderate to good yields. Selectivity experiments on L-serine and L-tyrosine methyl ester revealed an almost exclusive reaction of the amino group, with the final products being isolated in 59% (**4g**) and 61% (**4h**) yields, respectively. Lastly, the L-thiaproline methyl ester was also evaluated, but the final reaction mixture was too complex for the target product **4i** to be purified (Scheme 4B).

**Scheme 5:**
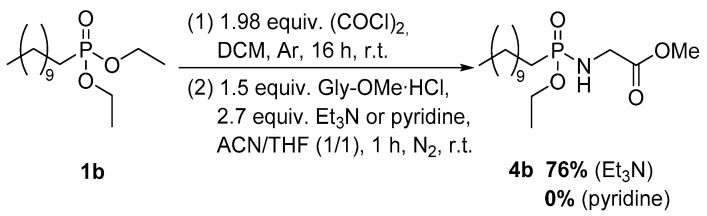
The scheme shows a comparison for the synthesis of glycine methyl ester phosphonamidate **4b** with triethylamine and pyridine.

Trying to summarize the work in this paper as well as the work from our previous paper [14], parallels can be drawn to the work of Mayr on the nucleophilicity parameters of organic compounds. In the same solvent, the difference in nucleophilicity between triethylamine (nucleophilicity parameter: 17.30 in CH_2_Cl_2_) and pyridine (nucleophilicity parameter: 12.9 in CH_2_Cl_2_) is almost five units. This makes it more understandable that triethylamine would play the role of a reaction mediator, whereas pyridine will likely act more as a base and thus fails to yield similar results to phosphonylaminium salts [19,20]. The fact that the choice of modulator makes a great difference is further supported by a comparative formation of glycine methyl ester phosphonamidate **4b** with either triethylamine or pyridine (Scheme 5). These data show that switching from triethylamine to pyridine drops the conversion to product **4b** entirely.

Secondly, as our transformations on amines, anilines and amino acid esters all proceed nicely, it is not surprising that these compounds have similar nucleophilicity parameters (13.77 for *tert*-butylamine, 15.7 for morpholine, 12.64 for aniline, and 14.75 for proline methyl ester, all in acetonitrile) [20,21,22]. In comparison, only a few alcohols were evaluated in the study conducted by Mishima and coworkers, albeit simple and different from those used in this study. Even then, these alcohols yield significantly lower values than the other discussed nucleophiles (nucleophilicity parameters: 7.13 for ethanol and 6.82 for isopropanol, both in acetonitrile), thus explaining why a larger excess of the nucleophilic alcohol is needed to ensure the formation of the target product [23]. The only group of nucleophiles missing from this library are phenols, which have not been studied so far.

### 2.2. Application for the Synthesis of N-acyl Homoserine Lactone Analogues

**Figure 1:**
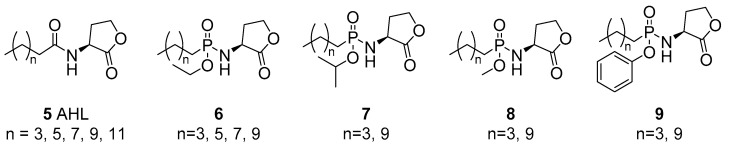
The figure shows an overview with naturally occurring *N*-acyl homoserine lactones **5** and the target phosphonamidate AHL analogues **6**–**9**.

As an illustration of potentially bioactive target compounds that can be accessed with this methodology and because of previous research of our group in the field of quorum sensing signalling molecules [24], these conditions were applied to the synthesis of a library of *N*-acyl homoserine lactone (AHL) analogues. Several AHL analogues with amide bond bioisosteres had already been described in the literature, such as sulfonamides [25], ureas [26], sulfonylureas [27], hydrazines [28] and even inverted amides [29]. However, corresponding phosphonamidates have not been reported previously. Therefore, different alkyl chains were targeted, accounting for the fact that the phosphorus moiety acts as a bio-isostere for the carbonyl moiety in the natural AHLs (**5**, Figure 1). Similarly, we wanted to include different phosphonester groups, expanding our scope from the ethyl and isopropyl esters (**6** and **7**, respectively) toward the less obvious methyl and phenyl esters (**8** and **9**).

**Scheme 6:**
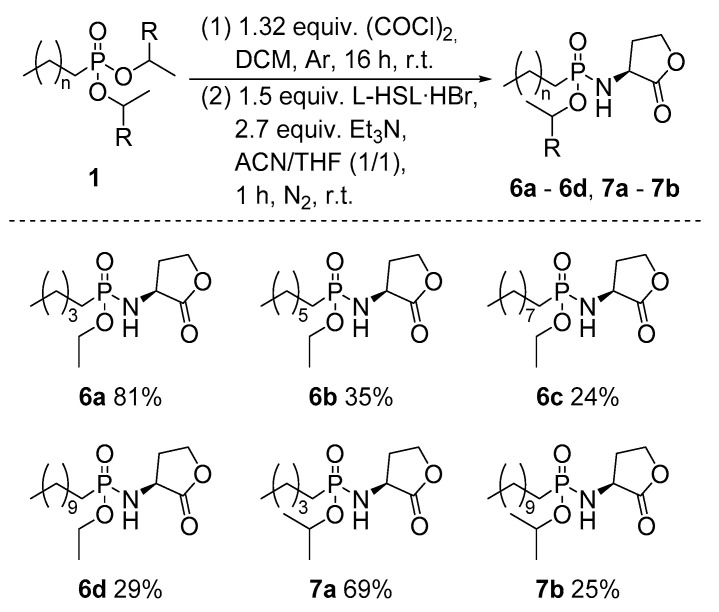
The scheme shows the synthesis of the targeted phosphonamidate AHL analogues (**6** and **7**) via the procedure in this paper.

The most straightforward analogues are those that can be synthesized via our method directly from diethyl or diisopropyl alkylphosphonates. These dialkyl *n*-alkylphosphonates need to be synthesized via the Michaelis–Arbuzov reaction from the corresponding alkyl halides (for details, see the Supplementary Materials). L-homoserine lactone hydrobromide, in turn, was prepared from L-methionine and bromoacetic acid via a modified version of a procedure presented in the literature [30]. The corresponding phosphonamidates with pentyl, heptyl, nonyl and undecyl chains and an ethyl ester group (Scheme 6, **6a** to **6d**) were isolated with variable yields, mainly due to the chromatographic purifications of the products. For the isopropyl analogues, we selected the shortest and longest chain lengths, with the yields showing a similar trend (**7a** and **7b**).

**Scheme 7:**
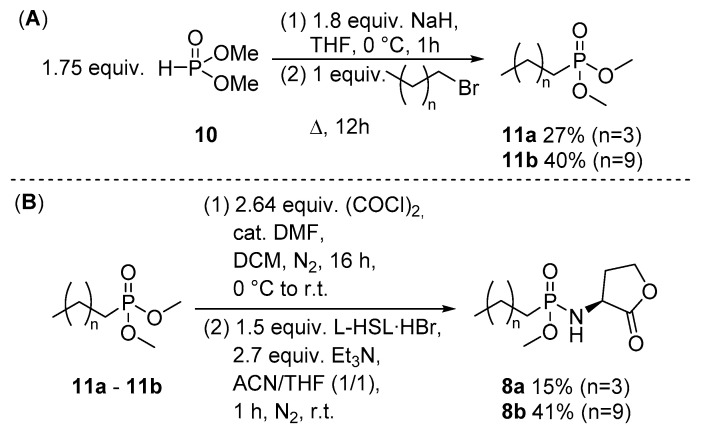
The scheme shows the (**A**) synthesis of the dimethyl *n*-alkylphosphonates **11** via the Michaelis-Becker reaction. (**B**) Synthesis of the targeted phosphonamidate AHL analogues **8** via a slightly adapted version of our procedure.

Due to their higher reactivity, the preparation of the corresponding methyl phosphonesters typically proves more challenging. This was already observed in the synthesis of the desired dimethyl *n*-alkylphosphonates, which had to be synthesized via a Michaelis–Becker reaction since no pure product could be obtained via the equivalent Michaelis–Arbuzov reaction (**11a** and **11b**, Scheme 7A). Continuing these observations, the reaction mixture had to be cooled down for the chlorination step to avoid side products in the process. This way, both novel targeted products **8a** and **8b** could be prepared in 15% and 41% yield, respectively (Scheme 7B). However, as reflected in the final yield, the chromatographic purification of product **8a** was not straightforward and led to the loss of product.

**Scheme 8:**
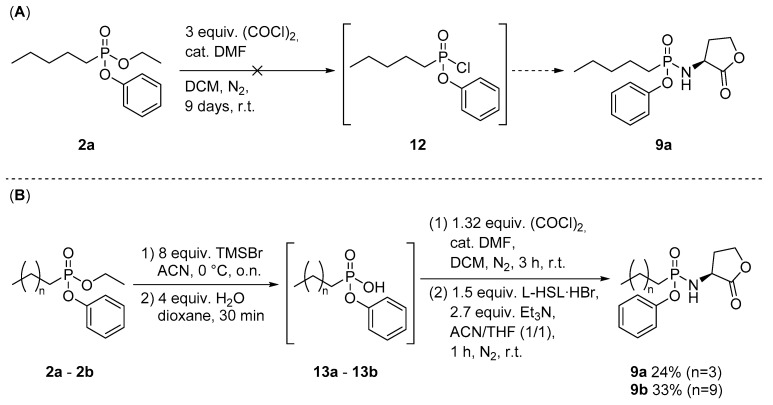
The scheme shows (**A**) attempted direct chlorination of product **2a**. (**B**) Synthesis of target products **9** via McKenna dealkylation and synthesis of the phosphonamidate moiety.

The analogues with phenyl ester moieties proved to be the most difficult since the phenyl ester usually needs to be introduced separately. This is the case because a hypothetical diphenyl *n*-alkylphosphonate can be made but cannot be chlorinated directly by oxalyl chloride or similar reagents. As in the mechanism, similar to the mechanism of the Michaelis–Arbuzov reaction, the chloride anion must be able to restore the P-O double bond by the elimination of the residual alkyl group, which is not possible with aryl residues. Therefore, we chose to start with mixed phosphonate **2a** (see above in Scheme 1). An attempt at direct chlorination of the ethoxy moiety of this mixed phosphonate proved to be slow, taking nine days for complete conversion, and the purity of this resulting chloridate **12** was insufficient to attempt the phosphonamidate coupling to target product **9a** (Scheme 8A). To ensure swift chlorination, we opted for a McKenna dealkylation of substrates **2a** and **2b**, which readily yielded the desired phosphonic acids **13a** and **13b**. These phosphonic acids were then directly used for the phosphonamidate coupling, yielding products **9a** and **9b** in yields of 24% and 33%, respectively, over two steps (Scheme 8B).

### 2.3. Application for the Use of Phosphoryl Chlorides

**Scheme 9:**
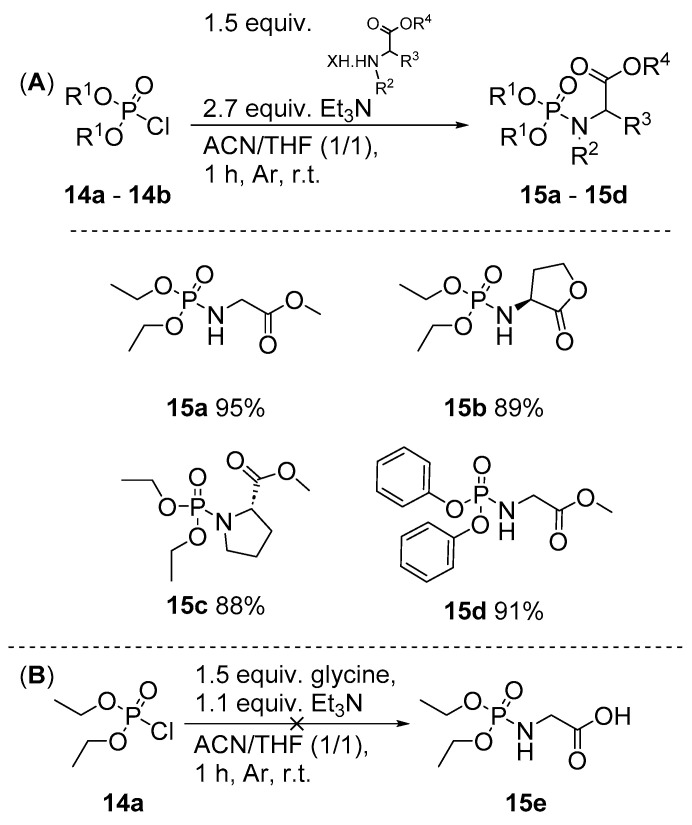
The scheme shows the (**A**) synthesis of amino acid-based phosphoramidates **15a**–**15d**, starting from commercial phosphorochloridates **14**. (**B**) Attempted synthesis of glycine phosphoramidate **15e**.

Similar to our previous work, we also wanted to extend our methodology to commercially available phosphoryl chlorides [14]. However, to our surprise, symmetric *N*-phosphorylated glycine or proline methyl esters have not yet been described in the literature. Nevertheless, *N*-phosphorylations of amino acid residues in the literature have been reported in mediocre to good yields [31,32,33,34]. These novel products can be accessed from commercially available diethyl phosphorochloridate **14a**, with the corresponding *N*-phosphonylated glycine methyl ester (**15a**, 95% yield), L-homoserine lactone (**15b**, 89% yield) and L-proline methyl ester (**15c**, 88% yield) being isolated in excellent yields (Scheme 9A). A similar reaction with diphenyl phosphorochloridate **14b** also proceeded nicely (**15d**, 91%). Finally, a comparable experiment with glycine as free acid did not yield any detectable target product (**15e**, Scheme 9B).

### 2.4. Screening for Herbicidal Activity

**Scheme 10:**
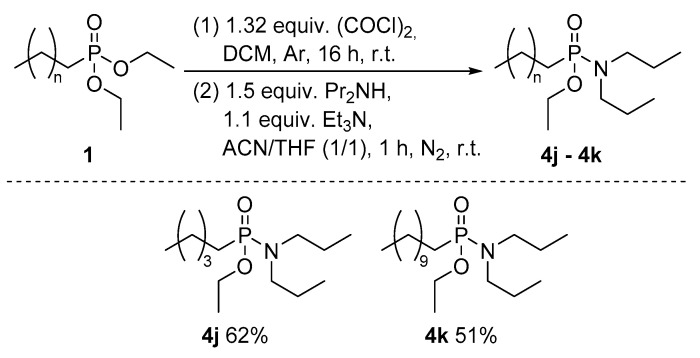
The scheme shows the synthesis of the di-*n*-propylamine phosphonamidates **4j** and **4k**.

**Figure 2:**
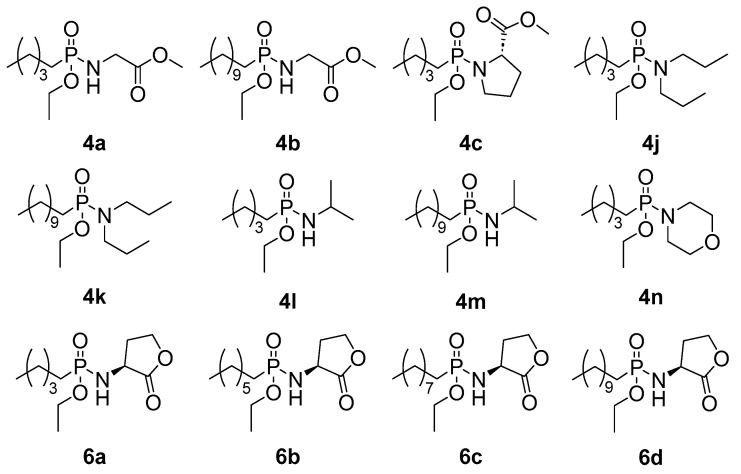
The figure shows an overview of the compounds selected for the herbicidal testing [14].

Based on the aforementioned patent [15], we wanted to test a selection of compounds to evaluate and expand on those results. As derivatives of di-*n*-propylamine showed the best activity, we used the procedure from our previous paper to synthesize *N*,*N*-dipropyl-phosphonamidates **4j** and **4k** in 62% and 51% yield, respectively (Scheme 10). In addition to these compounds, the isopropylamine and morpholine phosphonamidates (**4l**–**4n**, Figure 2) from our previous work were evaluated to increase the diversity in amine groups [14]. Similarly, glycine and L-proline phosphonamidates **4a**–**4c** (Scheme 4) were included as examples of amino acid-based products. Finally, the effect of the alkyl chain was also explored with the synthesized AHL analogues **6** (Scheme 5).

To enable rapid, quantitative herbicidal screening with small amounts of compound, a multispectral imaging-based in vitro assay was implemented in which tomato leaf disks in 96-well plates were exposed to the test compound by applying a 10 µL droplet at the center of each disk and monitoring changes in chlorophyll fluorescence (F_v_/F_m_), a proxy variable for the health of leaf tissue [35]. As a positive control, a high (10 mM) dose of the potent photosystem II-inhibiting herbicide diuron (DCMU) was used. IC_50_ values were determined by calculating the theoretical dose needed to achieve a 50% F_v_/F_m_ reduction induced by diuron (i.e., the F_v_/F_m_ value corresponding to dead tissue). Similar approaches have previously been used successfully to rapidly screen for resistance to existing herbicides in natural weed populations [36].

**Figure 3:**
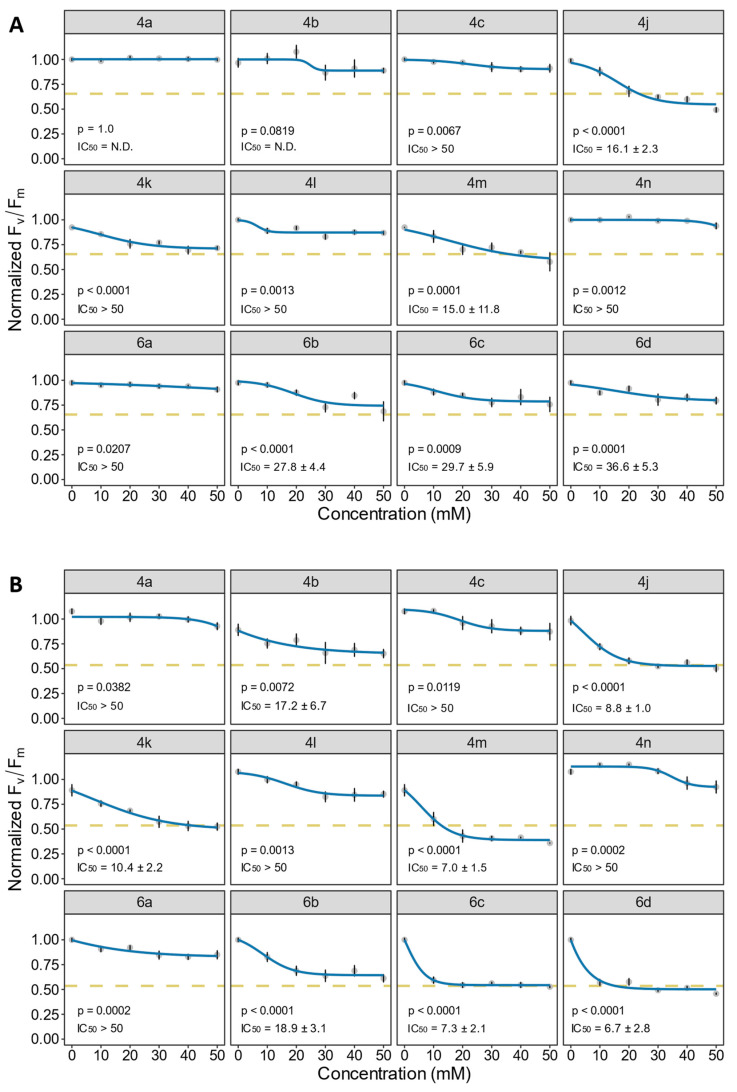
The figure shows the effect of selected phosphonamidates on chlorophyll fluorescence in *Solanum lycopersicum* leaf disks. F_v_/F_m_ measurements are shown 24 h (**A**) and 72 h (**B**) after application of the test compounds. Points with error bars show mean ± SEM, logistic regression curves superimposed in blue. The yellow dotted line is the residual F_v_/F_m_ displayed by dead leaf disks, i.e., leaf disks treated with a 10 mM diuron solution. IC_50_ values are the theoretical compound concentration required to achieve 50% of the F_v_/F_m_ reduction caused by diuron; *p*-values indicate the significance of the dose–effect relationship for each compound. No IC_50_ estimates were calculated where the dose–response relationship was not significant (N.D.), and IC_50_ values greater than the highest tested concentration were reported simply as ‘>50 mM’. N ≥ 4.

As shown in Figure 3, a wide range of activity was observed in our library of test compounds—ranging from no activity in **4a** to IC_50_ after 72 h of around 7 mM for compounds **4m**, **6c** and **6d**. The amine moiety clearly plays a critical role in determining herbicidal activity, with di-n-propylamine product **4j** showing an amplified activity as compared to the almost inactive **4l** and **4n** (IC_50_ after 72 h: >50 mM for both compounds) to an IC_50_ value of 8.8 mM. Similar effects on bioactivity can be seen for the amino acid moieties, where proline- and homoserine lactone-derived products **4c** and **6a** show detectable activity, but glycine derivative **4a** remains inactive or where AHL analogue **6d** shows an IC_50_ value of 6.7 mM, whereas the corresponding glycine phosphonamidate **4b** exhibits a much weaker activity with an IC_50_ value of 17.2 mM. There is a clear tendency for longer phosphonyl side chains to significantly increase herbicidal activity (**4a**–**4b**, **4l**–**4m**, and **6a**–**6d**), although **4j**–**4k** were an exception to this pattern as their IC_50_ values do not differ significantly (*p* = 0.52). However, for these last compounds, it can be said that the amine moiety is already very apolar, in contrast to the other tested amines or amino acid derivatives. This trend can be observed most clearly in the series of AHL analogues **6**, where the activity increases with longer phosphonyl chain lengths, reaching the lowest IC_50_ value in this study for **6d** with an IC_50_ value of 6.7 mM.

## 3. Materials and Methods

Full details on the equipment, reagents used, synthesis of the starting products and characterisation data of the final compounds can be found in the Supplementary Materials. The Supplementary Materials contain the following references that are not mentioned elsewhere [37,38,39,40,41,42,43,44,45,46,47].

### 3.1. General Procedure for the Synthesis of Phosphorus-Containing Products ***2***, ***3***, ***4***, ***6*** and ***7***

Under a nitrogen atmosphere, oxalyl chloride (1.32–2.64 equiv., 4.9–9.7 mmol) was added dropwise to a stirred solution of the corresponding phosphonate **1** (1 equiv., 3.7 mmol) in 20 mL dry DCM. This solution was stirred for 16 h at room temperature, and the conversion was monitored via ^31^P NMR. After the completion of the reaction, the solvent was evaporated under reduced pressure to yield a crude mixture of phosphonochloridate. Due to the instability of the compound, the crude compound was immediately used in subsequent reactions.

The corresponding phosphonochloridate and triethylamine (1.1 equiv., 4.0 mmol) were dissolved in 30 mL dry THF under a nitrogen atmosphere and stirred for 30 min at room temperature, after which the reaction mixture was analysed using ^31^P NMR. The desired phenol (1.1 equiv., 4.0 mmol), alcohol (5.5 equiv., 20.2 mmol), amine (1.5 equiv., 5.5 mmol) or amino acid hydrochloride (1.5 equiv., 5.5 mmol and 1.6 equiv., 5.9 mmol extra Et_3_N) was dissolved in 30 mL of dry ACN under nitrogen atmosphere while stirring at room temperature. The solution containing the phosphonochloridate was added dropwise to the solution of nucleophiles at room temperature and stirred for 1 h or until completion (monitored with ^31^P NMR). Subsequently, the solids were removed via filtration after the addition of 60 mL diethyl ether, and the solvent was evaporated under reduced pressure. The remaining mixture was purified via normal phase automatic flash chromatography.

### 3.2. Chlorophyll Fluorescence (Fv/Fm) Measurement

F_v_/F_m_ of leaf disks was measured using the PathoViewer multispectral imaging system, as described in previous work [37,38], and images were processed using the CropReporter software (v. 5.4.6-64b, PhenoVation). The herbicidal activity was assessed by measuring F_v_/F_m_, the maximum quantum efficiency of photosystem II (PSII) [35]. This parameter is a reliable quantitative proxy for stress in leaf tissues due to its robust correlation to the severity of biotic and abiotic stresses [35], including herbicide damage [36]. F_v_/F_m_ was measured after a fifteen-minute dark adaptation period at 24, 48, 72 and 96 h after treatment. In between treatments, leaf disks were incubated in a growth chamber (21 °C, 14/10 h light/day, 120 µmol m^−2^ s^−1^ at canopy level).

### 3.3. Plant Materials and Chemical Treatments

Compounds were applied to tomato leaf disks (*Solanum lycopersicum* ‘Moneymaker’). Tomato seeds were bought from Vreeken’s Zaden (Dordrecht, the Netherlands) and germinated in standard potting soil (Structural Type 1, Snebbout) at 21 °C. After germination, tomato seedlings were transplanted into individual 200 mL pots filled with the same potting substrate. All of the plants were kept at 21 °C with 16 h of full-spectrum LED light and were fertilized with tomato fertilizer weekly (NPK 19-8-16 + 4MgO + ME, Haifa Chemicals, 1 g L^−1^) and watered as needed. Plants were grown until the five-leaf stage, after which fully developed third or fourth leaves were detached and used to produce leaf disks with a 0.6 cm diameter cork bore. Leaf disks were placed in 96-well plates containing 200 µL of sterile distilled water per well and were left at room temperature for six hours before treatment to allow leaf disks to de-stress and adapt.

Chemical compounds were formulated by first preparing a concentrate consisting of 60% active ingredient, 35% DMSO and 5% Tween 20 by mass and then diluting this concentrate with distilled water to the desired concentration and vortexing until a homogeneous suspension was achieved. In all experiments, a formulation control consisting of Tween 20 and DMSO at the highest concentration used in the experiment was included. All experiments included 10 mM diuron (DCMU, Sigma-Aldrich, St. Louis, MO, USA) formulated in the same manner as the test compounds as a positive control. A single 10 µL droplet was placed in the center of each leaf disk. At least five disks, randomly sampled from different plants, were used per treatment. After exposure, leaf disks were kept at 21 °C under ambient lighting conditions.

### 3.4. Statistical Analysis

F_v_/F_m_ values were normalized by dividing them by the mean F_v_/F_m_ of negative control disks within the same experiment at the same time point. This has the effect of rescaling F_v_/F_m_ to the (0,1) interval and facilitating comparisons between experiments by removing the minor batch-to-batch variation in mean F_v_/F_m_ between experiments.

Dose–response curves based on normalized F_v_/F_m_ were fitted using four-parameter log-logistic models using the *drm* function in the R *drc* package (R v. 4.3.0.; *drc* v. 3.0-1) [48]. The lower asymptote was the mean value of the DCMU-treated controls, as we observed that even fully bleached, wilted and/or necrotic leaf tissues retain an appreciable normalized F_v_/F_m_. In our assays, even 1 mM DCMU led to rapid and systemic tissue death, so the F_v_/F_m_ value of leaf disks treated with a 10 mM dose provides a reliable baseline for the residual F_v_/F_m_ of dead tissue. The significance of the dose–response relationship was determined by comparing the fitted logistic regression curve to a horizontal line using the *noEffect* function. IC_50_ values ± SEM were then calculated using the *ED* function in the *drc* package [48].

## 4. Conclusions

To summarize, a novel set of mixed *n*-alkylphosphonate diesters was synthesized in good yields for a wide library of phenolic substrates. The same reaction conditions were also adopted for a tenfold increase in scale. Extending this methodology to alcohols proved more difficult, but benzyl and phenethyl alcohol could be included, nevertheless. Subsequently, this paper marks one of the only instances in the literature where amino acid-based *n*-alkylphosphonamidates can be synthesized directly from symmetrical phosphonate diesters without the need for the deprotonation of the nucleophile or complex reagents. These transformations were demonstrated for a wide range of amino acid derivatives, indicating a clear preference for *N*-phosphonylation over *O*-phosphonylation for both alcoholic hydroxyl groups as well as phenolic hydroxyl groups.

This phosphonylation protocol was then utilized for the synthesis of *N*-acyl homoserine lactone (AHL) analogues, where ethyl, isopropyl and methyl analogues with varying *n*-alkyl chains were readily synthesized. The targeted phenyl ester AHL analogues needed more care, requiring a McKenna dealkylation before ensuring the isolation of the targeted AHL analogues. Commercial chlorophosphates were, in turn, used to expand the chemistry towards the *N*-phosphorylation of amino acid esters and showed very nice yields for all substrates. Finally, we showcase a multispectral imaging-based assay for a rapid screening of herbicidal activity with minimal product use that was demonstrated on a selection of phosphonamidates from our work. Our results indicate that there is clearly potential for discovering herbicidal candidates in this class of phosphonamidates and that our methodology can facilitate future research in this direction.

## Data Availability

The original contributions presented in the study are included in the Supplementary Materials, further inquiries can be directed to the corresponding author.

## Supplementary Materials

The following supporting information can be downloaded at: https://www.mdpi.com/article/10.3390/ijms25094739/s1.
