# Supplementary material for: Synthesis of Mixed Phosphonate Esters and Amino Acid-Based Phosphonamidates, and Their Screening as Herbicides
Source: Int J Mol Sci. 2024 Apr 26;25(9):4739. doi: 10.3390/ijms25094739 (PMC11083600; doi:10.3390/ijms25094739)
Supplement: Supplementary file 1 [file ijms-25-04739-s001.zip › ijms-2963196-supplementary.pdf]

Supporting Information

Instrumentation

General procedure A: synthesis of dialkyl *P*-alkylphosphonates 1a – 1b, 1d – 1g

Synthesis of diethyl benzylphosphonate 1d

Synthesis of sarcosine methyl ester hydrochloride

Synthesis of homoserine lactone hydrobromide

General procedure B: Synthesis of phosphorus-containing products 2, 3, 4, 6 and 7

Synthesis of dimethyl alkylphosphonates 11a – 11b

Synthesis of methyl *P*-alkyl-*N*-((S)-2-oxotetrahydrofuran-3-yl) phosphonamidates 8a – 8b

Synthesis of phenyl *P*-alkyl-*N*-((S)-2-oxotetrahydrofuran-3-yl) phosphonamidates 9a – 9b

General procedure C: synthesis of phosphoramidates 15a – 15d

Bibliography

NMR spectra

Instrumentation

Commercially available starting materials, reagents and solvents were purchased from common chemical suppliers and used without further purification. Dry solvents were obtained via the MBraun SPS-800 solvent purification system. Under pressure of N_2 gas the solvents flow through two filter/dry columns. The columns are made of stainless steel (1.4301/US 304) with an internal volume of 4.8 L. The molecular sieves, as packing material, varies in accordance to the desired solvent. Methanol was dried over activated molecular sieves and the absence of water was confirmed by ^{1}H NMR analysis.

GC-MS analysis was performed on an Agilent 8890 GC System equipped with an Agilent J&W HP-5ms (30 m x 0.25 mm x 0.25 µm) column and an Agilent 5977B mass spectrometer with quadrupole mass analyser and Extractor EI source (Electron Ionization, 70 eV). HRMS analysis was conducted on an Agilent 1100 high-performance liquid chromatograph (HPLC) coupled to an Agilent 6220A TOF MS (Time of Flight Mass Spectrometer) equipped with an ESI/APCI (Electrospray Ionisation/ Atmospheric Pressure Chemical Ionization) multimode ionisation source. IR spectra with a S/N-ratio of 30,000:1 were obtained from samples in neat form with a Quest ATR (Attenuated Total Reflectance) accessory with diamond crystal puck using a Shimadzu IRAFFINITY-1S Fourier Transform Infrared Spectrophotometer (FTIR). LC-MS analyses were conducted with an Agilent 1200 Series High performance liquid chromatograph equipped with a Supelco Ascentic Express C18 column (3 cm x 4.6 mm, 2.7 µm fused-core particles, 90 Å), Phenomenex Guard column (SecurityGuard Standard). The mobile phase was a mixture of acetonitrile (ACN), methanol and/or water, depending on the selected program. The HPLC was coupled to an UV-DAD (Ultra-Violet Diode Array Detector) and an Agilent 1100 Series MS with Electrospray Ionisation (ESI, 4000 V) with a single quadrupole detector. Melting points of solid compounds were determined using a Kofler Bench, type WME Heizbank of Wagner & Munz or a Büchi M-560 melting point system. Automatic flash chromatography was performed on a Büchi Reveleris X2 flash chromatography system or a Büchi C815 Pure Chromatography System. Reusable columns (SiO_2, particle size of 0.040-0.063 mm) were used for the purification of the crude products. The effluent was analysed by an Evaporative Light Scattering Detector (ELSD) and three ultraviolet detectors of which the wavelengths were adjusted depending on the mixture to be purified. ^{1}H, ^{13}C and ^{31}P NMR spectra, as well as the 2D NMR spectra, were recorded at 400, 100.6 or 161.9 MHz respectively, on a Bruker Avance III, equipped with 1H/BB z-gradient probe (BBO, 5 mm). Before analysis, samples were dissolved in deuterated solvents ($CDCl_3$ and [D_6]DMSO) and tetramethylsilane (TMS) was added as internal chemical shift standard. All spectra were processed using TOPSPIN 3.6.2. The chemical shift as δ-value was reported in ppm. TLC was used to determine a suitable mobile phase for the automated flash chromatography via determination of the R_f-value. Compound mixtures were spotted on silica plates (Merck Silicagel 60 F254, precoated, thickness 0.25 mm). Detection of the compounds was done using a UV detector and iodine crystals in an enclosed recipient.

General procedure A: synthesis of dialkyl *P*-alkylphosphonates 1a – 1b, 1d – 1g

For the synthesis of these products, the same procedure was used as in our previous paper, the procedure and spectral data for diethyl pentylphosphonate **1a**, diethyl undecylphosphonate **1b** and diisopropyl undecylphosphonate **1g** can also be found there.[5]

The corresponding alkyl bromides (1 equiv.) and trialkyl phosphite (2.8 equiv.) are added to a 250 mL flask containing an Allihn condenser. The mixture was left for 16 hours while stirring at a temperature of 150 °C or 160 °C (for triethyl and triisopropyl phosphite respectively). The title compound was purified using vacuum distillation and the purity was determined using ^{31}P NMR. The resulting product was used without further purification.

Diisopropyl pentylphosphonate **1c**

Following general procedure A, 1-bromopentane (70 mmol) and freshly distilled triisopropyl phosphite (196 mmol) were used as starting products. Finally, 16.06 g diisopropyl pentylphosphonate **1c** was collected as a transparent oil with a yield of 97%.

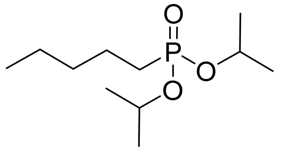

^{1}H NMR (400 MHz, CDCl$_3$): δ = 0.89 (t, *J* = 7.1 Hz, 3H, $C\underline{H}_3CH_2CH_2$), 1.28 – 1.40 (m, 16H, $CH_3(C\underline{H}_2)_2$ and 2 x $(C\underline{H}_3)_2CHO$), 1.53 – 1.72 (m, 4H, $C\underline{H}_2CH_2P$ and $C\underline{H}_2P$), 4.62 – 4.75 (m, 2H, 2 x $(CH_3)_2C\underline{H}O$) ppm; **^{13}C NMR** (100.6 MHz, CDCl$_3$): δ = 13.9 (s, $\underline{C}H_3CH_2CH_2$), 22.2 (d, *J* = 1.5 Hz, $CH_3\underline{C}H_2CH_2$), 22.3 (d, *J* = 5.1 Hz, $\underline{C}H_2CH_2P$), 24.2 (d, *J* = 3.7 Hz, 2 x $(\underline{C}H_3)_2CHO$), 27.0 (d, *J* = 142.3 Hz, $\underline{C}H_2P$), 32.9 (d, *J* = 16.9 Hz, $\underline{C}H_2(CH_2)_2P$), 69.7 (d, *J* = 6.6 Hz, 2 x $(CH_3)_2\underline{C}HO$) ppm; **^{31}P NMR** (161.9 MHz, CDCl$_3$): δ = 30.58 ($\underline{P}$) ppm; **IR** (ATR, cm^{-1}): ν = 2976 (C-H), 2932 (C-H), 1466, 1385, 1240, 1109, 978 (ν_{max}), 893; **MS** (ES$^+$): *m/z* (%): 237 (M+H$^+$, 42), 473 (2M+H$^+$, 100); **EI-MS** *m/z* (rel. int.): 236 [M] (1), 221 (6), 195 (11), 179 (29), 152 (100), 135 (14), 123 (14), 109 (7), 96 (26), 43 (10); **HRMS** (ESI$^+$): m/z calculated: $C_{11}H_{26}O_3P^+$ [M+H$^+$] 237.1614, found: 237.1622.

Diethyl heptylphosphonate **1e**

Following general procedure A, 1-bromoheptane (45 mmol) and triethyl phosphite (126 mmol) were used as starting products. Finally, 8.97 g diethyl heptylphosphonate **1e** was collected as a transparent oil with a yield of 84%.

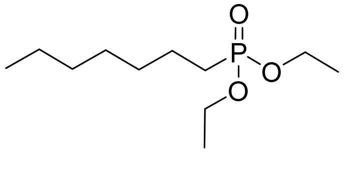

^{1}H NMR (400 MHz, CDCl$_3$): δ = 0.81 (3H, t, *J* = 6.4 Hz, $C\underline{H}_3CH_2CH_2$), 1.15-1.35 (14H, m, $CH_3(C\underline{H}_2)_4$ and 2 x $C\underline{H}_3CH_2O$), 1.46-1.59 (2H, m, $C\underline{H}_2CH_2P$), 1.59-1.71 (2H, m, $C\underline{H}_2P$), 3.94-4.09 (4H, m, 2 x $CH_3C\underline{H}_2O$) ppm; **^{13}C NMR** (100.5 MHz, CDCl$_3$): δ = 13.9 (s, $\underline{C}H_3CH_2CH_2$), 16.4 (d, *J* = 5.9 Hz, $\underline{C}H_3CH_2O$), 23.0 (d, *J* = 5.1 Hz, $\underline{C}H_2CH_2P$), 22.5 (s, $CH_3\underline{C}H_2CH_2$), 25.6 (d, *J* = 140.5 Hz, $\underline{C}H_2P$), 28.6 (s, $CH_3(CH_2)_2\underline{C}H_2$), 30.5 (d, *J* = 16.7, $\underline{C}H_2(CH_2)_2P$), 31.5 (s, $CH_3CH_2\underline{C}H_2$), 61.2 (d, *J* = 6.6 Hz, $CH_3\underline{C}H_2O$) ppm; **^{31}P NMR** (161.9 MHz, CDCl$_3$): δ = 32.48 ($\underline{P}$) ppm; **IR** (ATR, cm^{-1}): ν = 2930 (C-H), 2857 (C-H), 1456, 1390, 1242, 1026 (ν_{max}), 953, 785; **MS** (ES$^+$): *m/z* (%): 237 (M+H$^+$, 100), 473 (2M+H$^+$, 100); **EI-MS** *m/z* (rel. int.):

236 [M] (4), 207 (14), 193 (13), 179 (27), 165 (48), 152 (100), 138 (32), 125 (62), 111 (21), 97 (19); **HRMS** (ESI^+): m/z calculated: $C_{11}H_{26}O_3P^+$ [$M+H^+$] 237.1614, found: 237.1616.

Diethyl nonylphosphonate **1f**

Following general procedure A, 1-bromononane (35 mmol) and triethyl phosphite (98 mmol) were used as starting products. Finally, 7.85 g diethyl nonylphosphonate **1f** was collected as a transparent oil with a yield of 85%.

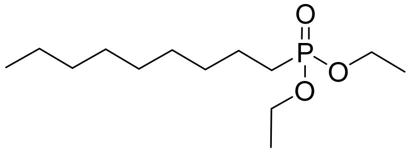

^{1}H NMR (400 MHz, $CDCl_3$): δ = 0.88 (3H, *J* = 6.8 Hz, $C\underline{H}_3CH_2CH_2$), 1.22-1.40 (18H, m, $CH_3(C\underline{H}_2)_6$ and 2 x $C\underline{H}_3CH_2O$), 1.54-1.66 (2H, m, $C\underline{H}_2CH_2P$), 1.67-1.76 (2H, m, $C\underline{H}_2P$), 4.02-4.16 (4H, m, 2 x $CH_3C\underline{H}_2O$) ppm; **^{13}C NMR** (100.6 MHz, $CDCl_3$): δ = 14.1 (s, $\underline{C}H_3CH_2CH_2$), 16.5 (app. t, *J* = 5.9 Hz, 2 x $\underline{C}H_3CH_2O$), 22.5 (d, *J* = 5.1 Hz, $\underline{C}H_2CH_2P$), 22.7 (s, $CH_3\underline{C}H_2CH_2$), 25.8 (d, *J* = 140 Hz, $\underline{C}H_2P$), 29.2 (s, $CH_3(CH_2)_2\underline{C}H_2$), 29.3 (s, $CH_3(CH_2)_3\underline{C}H_2$), 29.4 (s, $CH_3(CH_2)_4\underline{C}H_2$), 30.7 (d, *J* = 16.9 Hz, $\underline{C}H_2(CH_2)_2P$), 31.9 (s, $CH_3CH_2\underline{C}H_2$), 61.4 (d, *J* = 6.6 Hz, 2 x $CH_3\underline{C}H_2O$) ppm; **^{31}P NMR** (161.9 MHz, $CDCl_3$): δ = 32.61 ($\underline{P}$) ppm; **IR** (ATR, cm^{-1}): ν = 2924 (C-H), 2853 (C-H), 1458, 1391, 1244, 1030 (ν_{max}), 959, 787; **MS** (ES^+): *m/z* (%): 265 ($M+H^+$, 20), 529 ($2M+H^+$, 100), 551 ($2M+Na^+$, 100); **EI-MS** *m/z* (rel. int.): 264 [M] (3), 193 (13), 179 (24), 166 (17), 164 (47), 152 (100), 137 (17), 125 (48), 111 (15), 108 (13); **HRMS** (ESI^+): m/z calculated: $C_{13}H_{30}O_3P^+$ [$M+H^+$] 265.1927, found: 265.1931.

Synthesis of diethyl benzylphosphonate 1d

The title compound was synthesized according to Koehne et al., with benzyl bromide (42.1 mol) and triethyl phosphite (46.3 mmol) used as starting products. Finally, 8.7 g diethyl benzylphosphonate **1d** was collected as a transparent oil with a yield of 90%. The physical data, ^{1}H, ^{13}C and ^{31}P NMR data were in agreement with those reported by Koehne and Pietschnig.[6]

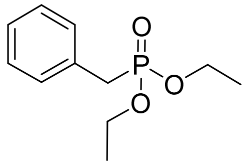

^{1}H NMR (400 MHz, $CDCl_3$): δ = 1.23 (t, *J* = 7.1 Hz, 6H, 2 x $C\underline{H}_3CH_2O$), 3.14 (d, *J* = 21.5 Hz, 2H, $C\underline{H}_2P$), 3.93 – 4.07 (m, 4H, 2 x $CH_3C\underline{H}_2O$), 7.18 – 7.35 (m, 5H, 5 x $C\underline{H}_{arom}$) ppm; **^{13}C NMR** (100.6 MHz, $CDCl_3$): δ = 16.3 (d, *J* = 5.9 Hz, 2 x $\underline{C}H_3CH_2O$), 33.8 (d, *J* = 138.1 Hz, $\underline{C}H_2P$), 62.1 (d, *J* = 6.6 Hz, 2 x $CH_3\underline{C}H_2O$), 126.8 (d, *J* = 3.7 Hz, $\underline{C}H_{arom,para}$), 128.5 (d, *J* = 3.0 Hz, 2 x $\underline{C}H_{arom,meta}$), 129.8 (d, *J* = 6.6 Hz, 2 x $\underline{C}H_{arom,ortho}$), 131.6 (d, *J* = 9.5 Hz, $\underline{C}_{arom,quat}$) ppm; **^{31}P NMR** (161.9 MHz, $CDCl_3$): δ = 26.36 ($\underline{P}$) ppm; **EI-MS** *m/z* (rel. int.): 228 [M] (50), 200 (17), 172 (36), 124 (30), 118 (42), 109 (28), 91 (100), 65 (19).

Synthesis of sarcosine methyl ester hydrochloride

Dry methanol (60 equiv., 230 mL) was added to a flame dried flask under nitrogen atmosphere and cooled down to 0 °C. Acetyl chloride (6 equiv., 41 mL) is subsequently added via a dropping funnel and stirred for 20 minutes at 0 °C. Subsequently the corresponding sarcosine (5.12 g, 57.5 mmol, 1 equiv.) is added and

the mixture is stirred for 18 hours under nitrogen atmosphere at room temperature. The solvent was removed *in vacuo* to obtain the target product as a white powder in a yield of 93%. Spectral data were in accordance with literature.[7]

Synthesis of homoserine lactone hydrobromide

The synthesis of homoserine lactone hydrobromide proceeded similarly as already described in our group by Syrpas et al.[8,9] In a 500 mL flask, (S)-methionine (15.2 g; 0.10 mol) and bromoacetic acid (1.1 equiv.; 15.4 g; 0.11 mol) were added to a 150 mL solvent mixture of H_2O-iPrOH-AcOH (5:5:2 v:v:v). The solution was left stirring overnight under reflux. After solvent removal under reduced pressure, the orange sticky oil was dissolved in 50 mL of a 4:1 mixture (v:v) of iPrOH – HBr (33% in AcOH) and crystallisation of the title compound set in. The first batch of homoserine lactone hydrobromide was collected by filtration and the purification procedure was repeated starting by evaporation of the filtrate to dryness. Additionally, both batches underwent a washing step with iPrOH, followed by evaporation under reduced pressure to obtain the pure, crystallized homoserine lactone hydrobromide salt (yield: 58-69%). All physical data, the LC-MS analyses and ^{1}H and ^{13}C NMR data were in agreement with those previously reported.[10,11]

General procedure B: Synthesis of phosphorus-containing products 2, 3, 4, 6 and 7

Under nitrogen atmosphere, oxalyl chloride (1.32 – 2.64 equiv., 4.9 - 9.7 mmol) is added dropwise to a stirred solution of the corresponding phosphonate **1** (1 equiv., 3.7 mmol) in 20 mL dry DCM. This solution is stirred for 16 hours at room temperature and the conversion is monitored via ^{31}P NMR. After completion of the reaction, solvent is evaporated under reduced pressure to yield a crude mixture of phosphonochloridate. Due to the instability of the compound, the crude compound is immediately used in subsequent reactions.

The corresponding phosphonochloridate and triethylamine (1.1 equiv., 4.0 mmol) were dissolved in 30 mL dry THF under nitrogen atmosphere and left stirring for 30 min at room temperature, after which the reaction mixture was analysed using ^{31}P NMR. The desired phenol (1.1 equiv., 4.0 mmol), alcohol (5.5 equiv., 20.2 mmol), amine (1.5 equiv., 5.5 mmol) or amino acid hydrochloride (1.5 equiv., 5.5 mmol and 1.6 equiv., 5.9 mmol extra Et_3N) was dissolved in 30 mL dry ACN under nitrogen atmosphere, while stirring at room temperature. The solution containing the phosphonochloridate was added dropwise to the solution of nucleophile at room temperature and stirred for 1 hour or until completion (monitored with ^{31}P NMR). Subsequently, the solids were removed via filtration, after addition of 60 mL diethyl ether and the solvent was evaporated under reduced pressure. The remaining mixture was purified via normal phase automatic flash chromatography.

Ethyl phenyl *P*-pentylphosphonate **2a**

Diethyl pentylphosphonate **1a** (3.7 mmol, 1 equiv.), oxalyl chloride (1.98 equiv.) and 1.1 equivalents of phenol (4.0 mmol) were used as starting products for general procedure B. After completion of the phosphonamidate coupling, the reaction mixture was extracted twice with 40 mL 1N NaOH, dried over $MgSO_4$ and the solvent was evaporated under reduced pressure. The crude product was purified using normal phase automatic flash chromatography (SiO_2, 30% ethyl acetate in hexane, 5% Et_3N) after which 700 mg of product **2a** was obtained as a pale yellow oil in a yield of 74%.

For the large scale synthesis of this product, general procedure B was followed on a scale of 36.8 mmol, with the modifications described above. Finally, 5207 mg product **2a** was obtained as a pale yellow oil in a yield of 55 %.

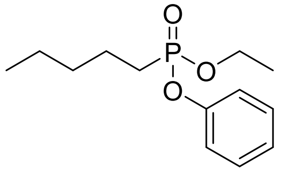

^{1}**H NMR** (400 MHz, $CDCl_3$): δ = 0.90 (t, *J* = 7.1 Hz, 3H, $C\underline{H}_3CH_2CH_2$), 1.20 – 1.45 (m, 7H, $CH_3(C\underline{H}_2)_2$ and $C\underline{H}_3CH_2O$), 1.62 – 1.77 (m, 2H, $C\underline{H}_2CH_2P$), 1.81 – 1.96 (m, 2H, $C\underline{H}_2P$), 4.05 – 4.28 (m, 2H, $CH_3C\underline{H}_2O$), 7.11 – 7.18 (m, 1H, $C\underline{H}_{arom,para}$), 7.18 – 7.25 (m, 2H, 2 x $C\underline{H}_{arom,ortho}$), 7.29 – 7.35 (m, 2H, 2 x $C\underline{H}_{arom,meta}$) ppm; ^{13}**C NMR** (100.6 MHz, $CDCl_3$): δ = 13.9 (s, $\underline{C}H_3CH_2CH_2$), 16.5 (d, *J* = 6.0 Hz, $\underline{C}H_3CH_2O$), 22.1 (d, *J* = 5.3 Hz, $\underline{C}H_2CH_2P$), 22.2 (d, *J* = 1.4 Hz, $CH_3\underline{C}H_2CH_2$), 25.8 (d, *J* = 140.4 Hz, $\underline{C}H_2P$), 32.7 (d, *J* = 17.0 Hz, $\underline{C}H_2CH_2CH_2P$), 62.4 (d, *J* = 7.0 Hz, $CH_3\underline{C}H_2O$), 120.6 (d, *J* = 4.2 Hz, 2 x $\underline{C}H_{arom,ortho}$), 124.8 (s, $\underline{C}H_{arom,para}$), 129.8 (s, 2 x $\underline{C}H_{arom,meta}$), 150.8 (d, *J* = 8.4 Hz, $\underline{C}_{arom,quat}$) ppm; ^{31}**P NMR** (161.9 MHz, $CDCl_3$): δ = 29.64 ($\underline{P}$) ppm; **IR** (ATR, cm^{-1}): ν = 2957 (C-H), 2932 (C-H), 2872 (C-H), 1593, 1489, 1246, 1204, 1036, 916 (ν_{max}), 760, 690; **MS** (ES^+): *m/z* (%): 257 ($M+H^+$, 95), 513 ($2M+H^+$, 100), 535 ($2M+Na^+$, 45); **EI-MS** *m/z* (rel. int.): 256 [M] (45), 227 (15), 213 (48), 200 (18), 185 (35), 172 (100), 135 (53), 118 (18), 104 (45), 94 (94), 77 (25), 65 (13), 43 (13); **HRMS** (ESI^+): m/z calculated: $C_{13}H_{22}O_3P^+$ [$M+H^+$] 257.1301, found: 257.1306.

Ethyl phenyl *P*-undecylphosphonate **2b**

Diethyl undecylphosphonate **1b** (3.7 mmol, 1 equiv.), oxalyl chloride (1.98 equiv.) and 1.1 equivalents of phenol (4.0 mmol) were used as starting products for general procedure B. After completion of the phosphonamidate coupling, the reaction mixture was extracted twice with 40 mL 1N NaOH, dried over $MgSO_4$ and the solvent was evaporated under reduced pressure. The crude product was purified using normal phase automatic flash chromatography (SiO_2, 30% ethyl acetate in hexane, 5% Et_3N) after which 920 mg of product **2b** was obtained as a pale yellow oil in a yield of 74%.

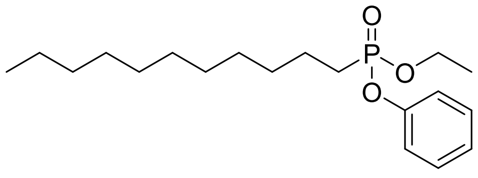

^{1}**H NMR** (400 MHz, $CDCl_3$): δ = 0.86 (t, *J* = 7.0 Hz, 3H, $C\underline{H}_3CH_2CH_2$), 1.16 – 1.46 (m, 19H, $CH_3(C\underline{H}_2)_8$ and $C\underline{H}_3CH_2O$), 1.61 – 1.76 (m, 2H, $C\underline{H}_2CH_2P$), 1.81 – 1.94 (m, 2H, $C\underline{H}_2P$), 4.01 – 4.37 (m, 2H, $CH_3C\underline{H}_2O$), 7.12 – 7.18 (m, 1H, $C\underline{H}_{arom,para}$), 7.18 – 7.23 (m, 2H, 2 x $C\underline{H}_{arom,ortho}$), 7.28 – 7.35 (m, 2H, 2 x $C\underline{H}_{arom,meta}$) ppm; ^{13}**C NMR** (100.6 MHz, $CDCl_3$): δ = 14.2 (s, $\underline{C}H_3CH_2CH_2$), 16.5 (d, *J* = 6.0 Hz, $\underline{C}H_3CH_2O$), 22.4 (d, *J* = 5.3 Hz, $\underline{C}H_2CH_2P$), 22.8 (s, $CH_3\underline{C}H_2CH_2$), 25.9 (d, *J* = 140.3 Hz, $\underline{C}H_2P$), 29.1, 29.1, 29.4, 29.4, 29.7 (s, $CH_3CH_2CH_2(\underline{C}H_2)_5$), 30.6 (d, *J* = 17.1 Hz, $\underline{C}H_2(CH_2)_2P$), 32.0 (s, $CH_3CH_2\underline{C}H_2$), 62.3 (d, *J* = 7.0 Hz, $CH_3\underline{C}H_2O$), 120.6 (d, *J* = 4.3 Hz, 2 x $\underline{C}H_{arom,ortho}$), 124.8 (s, $\underline{C}H_{arom,para}$), 129.8 (s, 2 x $\underline{C}H_{arom,meta}$), 150.8 (d, *J* = 8.4 Hz,

$\underline{C}_{arom,quat}$) ppm; **^{31}P NMR** (161.9 MHz, $CDCl_3$): δ = 29.65 ($\underline{P}$) ppm; **IR** (ATR, cm^{-1}): ν = 2924 (C-H), 2853 (C-H), 1595, 1491, 1258, 1206, 1036, 918 (ν_{max}), 760, 691; **MS** (ES^+): *m/z* (%): 341 ($M+H^+$, 100), 681 ($2M+H^+$, 90), 703 ($2M+Na^+$, 30); **EI-MS** *m/z* (rel. int.): 340 [M] (24), 311 (10), 283 (10), 269 (12), 255 (20), 241 (16), 227 (32), 213 (64), 200 (24), 185 (20), 172 (100), 118 (18), 104 (36), 94 (56), 77 (10), 55 (12), 41 (8); **HRMS** (ESI^+): m/z calculated: $C_{19}H_{34}O_3P^+$ [$M+H^+$] 341.2240, found: 341.2246.

Ethyl (4-(2-hydroxyethyl)phenyl) *P*-pentylphosphonate **2c**

Diethyl pentylphosphonate **1a** (3.9 mmol, 1 equiv.), oxalyl chloride (1.98 equiv.) and 1.1 equivalents of tyrosol (4.3 mmol) were used as starting products for general procedure B. After completion of the phosphonamidate coupling, the reaction mixture was extracted twice with 40 mL 1N NaOH, dried over $MgSO_4$ and the solvent was evaporated under reduced pressure. The crude product was purified using normal phase automatic flash chromatography (SiO_2, 0% – 40% (EtOAc:iPA, 3:1 v:v) in hexane), after which 842 mg of product **2c** was obtained as an orange oil in a yield of 72%.

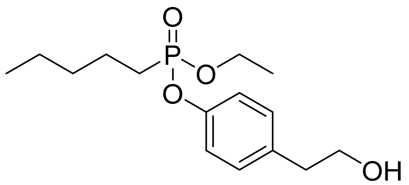

^{1}H NMR (400 MHz, $CDCl_3$): δ = 0.90 (t, *J* = 7.1 Hz, 3H, $C\underline{H}_3CH_2CH_2$), 1.28 – 1.40 (m, 7H, $CH_3(C\underline{H}_2)_2$ and $C\underline{H}_3CH_2O$), 1.57 – 1.79 (m, 2H, $C\underline{H}_2CH_2P$), 1.80 – 1.95 (m, 2H, $C\underline{H}_2P$), 2,22 (br s, 1H, OH), 2.82 (t, *J* = 6.7 Hz, 2H, $C\underline{H}_2CH_2OH$), 3.80 (t, *J* = 6.7 Hz, 2H, $CH_2C\underline{H}_2OH$), 4.11 – 4.23 (m, 2H, $CH_3C\underline{H}_2O$), 7.08 – 7.22 (m, 4H, 4 x $C\underline{H}_{arom}$) ppm; **^{13}C NMR** (100.6 MHz, $CDCl_3$): δ = 13.9 (s, $\underline{C}H_3CH_2CH_2$), 16.5 (d, *J* = 6.0 Hz, $\underline{C}H_3CH_2O$), 22.1 (d, *J* = 5.5 Hz, $\underline{C}H_2CH_2P$), 22.2 (d, *J* = 1.4 Hz, $CH_3\underline{C}H_2CH_2$), 25.8 (d, *J* = 140.3 Hz, $\underline{C}H_2P$), 32.7 (d, *J* = 17.0 Hz, $\underline{C}H_2CH_2CH_2P$), 38.6 (s, $\underline{C}H_2CH_2OH$), 62.4 (d, *J* = 7.0 Hz, $CH_3\underline{C}H_2O$), 63.6 (s, $CH_2\underline{C}H_2OH$), 120.6 (d, *J* = 4.3 Hz, 2 x $POC_q\underline{C}H$), 130.3 (s, 2 x $CH_2C_q\underline{C}H$), 135.3 (s, $CH_2\underline{C}_q$), 149.3 (d, *J* = 8.5 Hz, $PO\underline{C}_q$) ppm; **^{31}P NMR** (161.9 MHz, $CDCl_3$): δ = 29.81 ($\underline{P}$) ppm; **IR** (ATR, cm^{-1}): ν = 3414 (O-H), 2932 (C-H), 2875 (C-H), 1713, 1506, 1209, 1036, 916 (ν_{max}), 827, 528; **MS** (ES^+): *m/z* (%): 301 ($M+H^+$, 100), 323 ($M+Na^+$, 20), 601 ($2M+H^+$, 14); **EI-MS** *m/z* (rel. int.): 300 [M] (6), 270 (100), 242 (31), 214 (6), 186 (8), 163 (18), 135 (31), 107 (38), 77 (6), 43 (6); **HRMS** (ESI^+): m/z calculated: $C_{15}H_{26}O_4P^+$ [$M+H^+$] 301.1563, found: 301.1554.

Ethyl 4-methoxyphenyl *P*-pentylphosphonate **2d**

Diethyl pentylphosphonate **1a** (3.7 mmol, 1 equiv.), oxalyl chloride (1.98 equiv.) and 1.1 equivalents of 4-methoxyphenol (4.0 mmol) were used as starting products for general procedure B. After completion of the phosphonamidate coupling, the reaction mixture was extracted twice with 40 mL 1N NaOH, dried over $MgSO_4$ and the solvent was evaporated under reduced pressure. The crude product was purified using normal phase automatic flash chromatography SiO_2, 30% ethyl acetate in hexane, 5% Et_3N), after which 687 mg of product **2d** was obtained as a pale yellow oil in a yield of 65%.

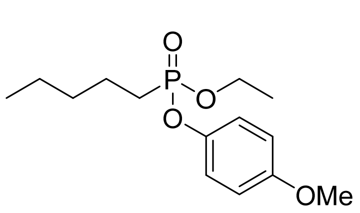

^{1}H NMR (400 MHz, $CDCl_3$): δ = 0.90 (t, *J* = 7.1 Hz, 3H, $C\underline{H}_3CH_2CH_2$), 1.22 – 1.46 (m, 7H, $CH_3(C\underline{H}_2)_2$ and $C\underline{H}_3CH_2O$), 1.57 – 1.75 (m, 2H, $C\underline{H}_2CH_2P$), 1.79 – 1.93 (m, 2H, $C\underline{H}_2P$), 3.78 (s, 3H, $OC\underline{H}_3$), 4.03 – 4.26 (m, 2H, $CH_3C\underline{H}_2O$), 6.81 – 6.87 (m, 2H, 2 x $CH_3OC_qC\underline{H}$), 7.09 – 7.16 (m, 2H, 2 x $POC_qC\underline{H}$) ppm;

^{13}C NMR (100.6 MHz, $CDCl_3$): δ = 13.8 (s, $\underline{C}H_3CH_2CH_2$), 16.4 (d, *J* = 6.0 Hz, $\underline{C}H_3CH_2O$), 22.0 (d, *J* = 5.4 Hz, $\underline{C}H_2CH_2P$), 22.1 (d, *J* = 1.3 Hz, $CH_3\underline{C}H_2CH_2$), 25.6 (d, *J* = 140.4 Hz, $\underline{C}H_2P$), 32.7 (d, *J* = 17.1 Hz, $\underline{C}H_2CH_2CH_2P$), 55.6 (s, $O\underline{C}H_3$), 62.3 (d, *J* = 6.9 Hz, $CH_3\underline{C}H_2O$), 114.7 (s, 2 x $CH_3OC_q\underline{C}H$), 121.4 (d, *J* = 4.1 Hz, 2 x $POC_q\underline{C}H$), 144.2 (d, *J* = 8.5 Hz, $PO\underline{C}_q$), 156.6 (s, $CH_3O\underline{C}_q$) ppm; **^{31}P NMR** (161.9 MHz, $CDCl_3$): δ = 29.95 ($\underline{P}$) ppm; **IR** (ATR, cm^{-1}): ν = 2932 (C-H), 2872 (C-H), 1504 (ν_{max}), 1248, 1200, 1032, 912; **MS** (ES^+): *m/z* (%): 287 ($M+H^+$, 84), 573 ($2M+H^+$, 100), 595 ($2M+Na^+$, 40); **EI-MS** *m/z* (rel. int.): 286 [M] (100), 243 (29), 215 (23), 202 (19), 138 (42), 124 (94), 121 (87), 109 (26); **HRMS** (ESI^+): m/z calculated: $C_{14}H_{24}O_4P^+$ [$M+H^+$] 287.1407, found: 287.1411.

4-Acetylphenyl ethyl *P*-pentylphosphonate **2e**

Diethyl pentylphosphonate **1a** (3.7 mmol, 1 equiv.), oxalyl chloride (1.98 equiv.) and 1.1 equivalents of 4-acetylphenol (4.0 mmol) were used as starting products for general procedure B. After completion of the phosphonamidate coupling, the reaction mixture was extracted twice with 40 mL 1N NaOH, dried over $MgSO_4$ and the solvent was evaporated under reduced pressure. The crude product was purified using normal phase automatic flash chromatography SiO_2, 30% ethyl acetate in hexane, 5% Et_3N), after which 670 mg of product **2e** was obtained as a yellow oil in a yield of 61%.

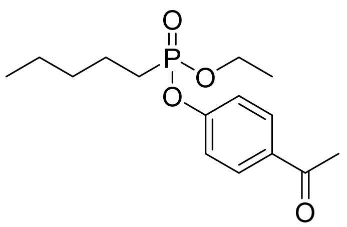

^{1}H NMR (400 MHz, $CDCl_3$): δ = 0.90 (t, *J* = 7.0 Hz, 3H, $C\underline{H}_3CH_2CH_2$), 1.27 – 1.44 (m, 7H, $CH_3(C\underline{H}_2)_2$ and $C\underline{H}_3CH_2O$), 1.62 – 1.75 (m, 2H, $C\underline{H}_2CH_2P$), 1.84 – 1.98 (m, 2H, $C\underline{H}_2P$), 2.59 (s, 3H, $C\underline{H}_3C{=}O$), 4.06 – 4.28 (m, 2H, $CH_3C\underline{H}_2O$), 7.28 – 7.33 (m, 2H, 2 x $POC_qC\underline{H}$), 7.91 – 8.02 (m, 2H, 2 x $O{=}CC_qC\underline{H}$) ppm; **^{13}C NMR** (100.6 MHz, $CDCl_3$): δ = 13.8 (s, $\underline{C}H_3CH_2CH_2$), 16.4 (d, *J* = 6.0 Hz, $\underline{C}H_3CH_2O$), 22.0 (d, *J* = 5.5 Hz, $\underline{C}H_2CH_2P$), 22.1 (d, *J* = 1.3 Hz, $CH_3\underline{C}H_2CH_2$), 25.9 (d, *J* = 140.2 Hz, $\underline{C}H_2P$), 26.5 (s, $\underline{C}H_3C{=}O$), 32.6 (d, *J* = 17.0 Hz, $\underline{C}H_2CH_2CH_2P$), 62.6 (d, *J* = 7.0 Hz, $CH_3\underline{C}H_2O$), 120.4 (d, *J* = 4.5 Hz, 2 x $POC_q\underline{C}H$), 130.4 (s, 2 x $O{=}CC_q\underline{C}H$), 133.8 (s, 2 x $O{=}C\underline{C}_q$), 154.7 (d, *J* = 8.3 Hz, $PO\underline{C}_q$), 196.8 (s, $\underline{C}{=}O$) ppm; **^{31}P NMR** (161.9 MHz, $CDCl_3$): δ = 29.99 ($\underline{P}$) ppm; **IR** (ATR, cm^{-1}): ν = 2932 (C-H), 2872 (C-H), 1682, 1597, 1504, 1263, 1217, 1034, 912 (ν_{max}); **MS** (ES^+): *m/z* (%): 299 ($M+H^+$, 100), 597 ($2M+H^+$, 25), 619 ($2M+Na^+$, 20); **EI-MS** *m/z* (rel. int.): 298 [M] (35), 283 (100), 255 (35), 227 (53), 214 (23), 199 (31), 146 (17), 135 (16), 121 (50), 43 (13); **HRMS** (ESI^+): m/z calculated: $C_{15}H_{24}O_4P$ [$M+H^+$] 299.1407, found: 299.1408.

Benzyl ethyl *P*-pentylphosphonate **2f**

Diethyl pentylphosphonate **1a** (3.7 mmol, 1 equiv.), oxalyl chloride (1.98 equiv.) and 5.5 equivalents of benzyl alcohol (20.21 mmol) were used as starting products for general procedure B. The crude product was purified using reversed-phase automatic flash chromatography (C18, 5 CV at 1/1 ACN/H_2O, then in 10 CV to 100% ACN), after which 680 mg of product **2f** was obtained as a yellow oil in a yield of 68%.

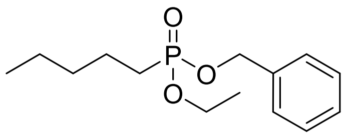

^{1}H NMR (400 MHz, $CDCl_3$): δ = 0.88 (t, *J* = 7.2 Hz, 3H, $C\underline{H}_3CH_2CH_2$), 1.23 – 1.38 (m, 7H, $CH_3(C\underline{H}_2)_2$ and $C\underline{H}_3CH_2O$), 1.52 – 1.64 (m, 2H, $C\underline{H}_2CH_2P$), 1.67 – 1.78 (m, 2H, $C\underline{H}_2P$), 3.94 – 4.16 (m, 2H, $CH_3C\underline{H}_2O$), 5.00 – 5.13 (m, 2H,

$C_{arom,quat}C\underline{H}_2O$), 7.28 – 7.43 (m, 5H, 5 x $C\underline{H}_{arom}$) ppm; **^{13}C NMR** (100.6 MHz, $CDCl_3$): δ = 13.9 (s, $\underline{C}H_3CH_2CH_2$), 16.5 (d, *J* = 6.0 Hz, $\underline{C}H_3CH_2O$), 22.1 (d, *J* = 5.2 Hz, $\underline{C}H_2CH_2P$), 22.2 (d, *J* = 1.4 Hz, $CH_3\underline{C}H_2CH_2$), 25.9 (d, *J* = 140.1 Hz, $\underline{C}H_2P$), 32.8 (d, *J* = 16.9 Hz, $\underline{C}H_2CH_2CH_2P$), 61.6 (d, *J* = 6.6 Hz, $CH_3\underline{C}H_2O$), 67.0 (d, *J* = 6.5 Hz, $C_{arom,quat}\underline{C}H_2O$), 127.9 (s, 2 x $\underline{C}H_{arom,ortho}$), 128.4 (s, $\underline{C}H_{arom,para}$), 128.7 (s, 2 x $\underline{C}H_{arom,meta}$), 136.8 (d, *J* = 5.9 Hz, $\underline{C}_{arom,quat}$) ppm; **^{31}P NMR** (161.9 MHz, $CDCl_3$): δ = 33.20 ($\underline{P}$) ppm; **IR** (ATR, cm^{-1}): ν = 2955 (C-H), 2932 (C-H), 2872 (C-H), 1456, 1238, 1005, 1001 ($ν_{max}$), 959, 696; **MS** (ES^+): *m/z* (%): 271 ($M+H^+$, 52), 541 ($2M+H^+$, 100); **EI-MS** *m/z* (rel. int.): 270 [M] (23), 214 (8), 179 (54), 151 (58), 124 (19), 108 (35), 91 (100), 65 (19); **HRMS** (ESI^+): m/z calculated: $C_{14}H_{24}O_3P^+$ [$M+H^+$] 271.1458, found: 271.1463.

Ethyl phenethyl *P*-pentylphosphonate **2g**

Diethyl pentylphosphonate **1a** (3.7 mmol, 1 equiv.), oxalyl chloride (1.98 equiv.) and 5.5 equivalents of phenethyl alcohol (20.21 mmol) were used as starting products for general procedure B. The crude product was purified using reversed-phase automatic flash chromatography (C18, 5 CV at 1/1 ACN/H_2O, then in 10 CV to 100% ACN) and normal phase automatic flash chromatography (SiO_2, 0% - 100% ethyl acetate in hexane), after which 350 mg of product **2g** was obtained as a pale oil in a yield of 34%.

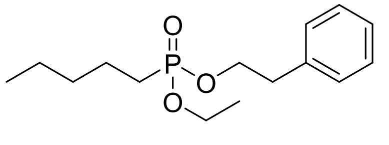

^{1}H NMR (400 MHz, $CDCl_3$): δ = 0.88 (t, *J* = 6.9 Hz, 3H, $C\underline{H}_3CH_2CH_2$), 1.20 – 1.38 (m, 7H, $CH_3(C\underline{H}_2)_2$ and $C\underline{H}_3CH_2O$), 1.46 – 1.58 (m, 2H, $C\underline{H}_2CH_2P$), 1.58 – 1.75 (m, 2H, $C\underline{H}_2P$), 2.97 (t, *J* = 6.3 Hz, 2H, $C_{arom,quat}C\underline{H}_2CH_2O$), 3.83 – 4.10 (m, 2H, $CH_3C\underline{H}_2O$), 4.13 – 4.36 (m, 2H, $C_{arom,quat}CH_2C\underline{H}_2O$), 7.12 – 7.44 (m, 5H, 5 x $C\underline{H}_{arom}$) ppm; **^{13}C NMR** (100.6 MHz, $CDCl_3$): δ = 14.0 (s, $\underline{C}H_3CH_2CH_2$), 16.6 (d, *J* = 6.1 Hz, $\underline{C}H_3CH_2O$), 22.1 (d, *J* = 5.4 Hz, $\underline{C}H_2CH_2P$), 22.3 (d, *J* = 1.4 Hz, $CH_3\underline{C}H_2CH_2$), 25.7 (d, *J* = 140.3 Hz, $\underline{C}H_2P$), 32.9 (d, *J* = 16.9 Hz, $\underline{C}H_2CH_2CH_2P$), 37.2 (d, *J* = 6.2 Hz, $C_{arom,quat}\underline{C}H_2CH_2O$), 61.5 (d, *J* = 6.6 Hz, $CH_3\underline{C}H_2O$), 65.9 (d, *J* = 6.7 Hz, $C_{arom,quat}CH_2\underline{C}H_2O$), 126.8 (s, $\underline{C}H_{arom,para}$), 128.6 (s, 2 x $\underline{C}H_{arom,meta}$), 129.2 (s, 2 x $\underline{C}H_{arom,ortho}$), 137.6 (s, $\underline{C}_{arom,quat}CH_2CH_2O$) ppm; **^{31}P NMR** (161.9 MHz, $CDCl_3$): δ = 32.79 ($\underline{P}$) ppm; **IR** (ATR, cm^{-1}): ν = 2955 (C-H), 2930 (C-H), 2872 (C-H), 1454, 1391, 1236, 1011 ($ν_{max}$), 959; **MS** (ES^+): *m/z* (%): 285 ($M+H^+$, 100), 569 ($2M+H^+$, 83), 591 ($2M+Na^+$, 36); **EI-MS** *m/z* (rel. int.): 284 [M] (1), 104 (100), 91 (5), 78 (5); **HRMS** (ESI^+): m/z calculated: $C_{15}H_{26}O_3P^+$ [$M+H^+$] 285.1614, found: 285.1618.

O-Ethyl *S*-phenyl *P*-pentylphosphonothioate **3**

Diethyl pentylphosphonate **1a** (3.7 mmol, 1 equiv.), oxalyl chloride (2.64 equiv.) and 1.1 equivalents of thiophenol (4.0 mmol) were used as starting products for general procedure B. After completion of the phosphonamidate coupling, the reaction mixture was extracted twice with 40 mL 1N NaOH, dried over $MgSO_4$ and the solvent was evaporated under reduced pressure. The crude product was purified using normal phase automatic flash chromatography (SiO_2, 30% ethyl acetate in hexane, 5% Et_3N), after which 630 mg of product **3** was obtained as a clear oil in a yield of 63%.

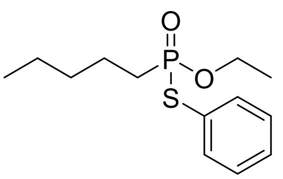

^{1}H NMR (400 MHz, $CDCl_3$): δ = 0.87 (t, *J* = 7.2 Hz, 3H, $C\underline{H}_3CH_2CH_2$), 1.21 – 1.40 (m, 7H, $CH_3(C\underline{H}_2)_2$ and $C\underline{H}_3CH_2O$), 1.53 – 1.77 (m, 2H, $C\underline{H}_2CH_2P$), 1.81 – 1.91 (m, 2H, $C\underline{H}_2P$), 4.09 – 4.38 (m, 2H, $CH_3C\underline{H}_2O$), 7.31 – 7.39 (m, 3H, 2 x $C\underline{H}_{arom,ortho}$ and $C\underline{H}_{arom,para}$), 7.50 – 7.59 (m, 2H, 2 x $C\underline{H}_{arom,meta}$) ppm; **^{13}C NMR** (100.6 MHz, $CDCl_3$): δ = 13.8 (s, $\underline{C}H_3CH_2CH_2$), 16.2 (d, *J* = 6.7 Hz, $\underline{C}H_3CH_2O$), 21.7 (d, *J* = 5.0 Hz, $\underline{C}H_2CH_2P$), 22.1 (d, *J* = 1.4 Hz, $CH_3\underline{C}H_2CH_2$), 31.4 (d, *J* = 104.6 Hz, $\underline{C}H_2P$), 32.5 (d, *J* = 17.4 Hz, $\underline{C}H_2CH_2CH_2P$), 61.6 (d, *J* = 7.5 Hz, $CH_3\underline{C}H_2O$), 127.5 (d, *J* = 5.2 Hz, $\underline{C}_{arom,quat}$), 129.0 (d, *J* = 2.5 Hz, $\underline{C}H_{arom,para}$), 129.4 (d, *J* = 1.9 Hz, 2 x $\underline{C}H_{arom,ortho}$), 135.0 (d, *J* = 4.1 Hz, 2 x $\underline{C}H_{arom,meta}$) ppm; **^{31}P NMR** (161.9 MHz, $CDCl_3$): δ = 55.91 ($\underline{P}$) ppm; **IR** (ATR, cm^{-1}): ν = 2930 (C-H), 2870 (C-H), 1440, 1231, 1020 (ν_{max}), 953, 744, 691; **MS** (ES^+): *m/z* (%): 273 ($M+H^+$, 100), 545 ($2M+H^+$, 60), 567 ($2M+Na^+$, 40); **EI-MS** *m/z* (rel. int.): 272 [M] (38), 230 (14), 216 (28), 188 (34), 163 (40), 135 (100), 110 (66), 71 (22), 69 (28), 65 (22), 43 (20); **HRMS** (ESI^+): m/z calculated: $C_{13}H_{22}O_2PS^+$ [$M+H^+$] 273.1073, found: 273.1076.

Methyl *N*-(ethoxy(pentyl)phosphoryl)glycinate **4a**

Diethyl pentylphosphonate **1a** (3.7 mmol, 1 equiv.), oxalyl chloride (1.98 equiv.) and glycine methyl ester hydrochloride (5.5 mmol, 1.5 equiv.) were used as starting products for general procedure B. Together with the amino acid, 1.6 equiv. of triethylamine (5.9 mmol) was added to neutralize the hydrochloride salt. The crude mixture was purified via normal phase automatic flash chromatography (SiO_2, 20% (EtOAc:iPA, 3:1 v:v) in hexane), after which 690 mg of product **4a** was obtained as a yellow oil in a yield of 75%.

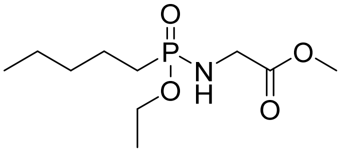

^{1}H NMR (400 MHz, $CDCl_3$): δ = 0.89 (t, *J* = 6.9 Hz, 3H, $C\underline{H}_3CH_2CH_2$), 1.22 – 1.43 (m, 7H, $CH_3(C\underline{H}_2)_2$ and $C\underline{H}_3CH_2O$), 1.48 – 1.87 (m, 4H, $C\underline{H}_2CH_2P$ and $C\underline{H}_2P$), 2.93 – 3.17 (m, 1H, $N\underline{H}CH_2$), 3.63 – 3.87 (m, 5H, $COOC\underline{H}_3$ and $NHC\underline{H}_2$), 3.95 – 4.24 (m, 2H, $CH_3C\underline{H}_2O$) ppm; **^{13}C NMR** (100.6 MHz, $CDCl_3$): δ = 13.9 (s, $\underline{C}H_3CH_2CH_2$), 16.5 (d, *J* = 6.5 Hz, $CH_3\underline{C}H_2O$), 22.1 (d, *J* = 5.2 Hz, $\underline{C}H_2CH_2P$), 22.3 (s, $CH_3\underline{C}H_2CH_2$), 27.8 (d, *J* = 131.0 Hz, $\underline{C}H_2P$), 32.9 (d, *J* = 17.1 Hz, $CH_3CH_2\underline{C}H_2$), 42.6 (s, $NH\underline{C}H_2$), 52.3 (s, $COO\underline{C}H_3$), 59.8 (d, *J* = 6.9 Hz, $CH_3\underline{C}H_2O$), 172.1 (d, *J* = 5.4 Hz, $\underline{C}OO$) ppm; **^{31}P NMR** (161.9 MHz, $CDCl_3$): δ = 34.94 ($\underline{P}$) ppm; **IR** (ATR, cm^{-1}): ν = 3199 (N-H), 2931 (C-H), 1753 (C=O), 1454, 1436, 1195 (C-O), 1147 (ν_{max}), 1035, 950, 856; **MS** (ES^+): *m/z* (%): 252 ($M+H^+$, 100), 503 ($2M+H^+$, 80), 525 ($2M+Na^+$, 18); **EI-MS** *m/z* (rel. int.): 251 [M] (8), 219 (8), 208 (8), 192 (100), 163 (37), 148 (15), 135 (43), 122 (7), 108 (15), 71 (7), 43 (7); **HRMS** (ESI^+): m/z calculated: $C_{10}H_{23}NO_4P^+$ [$M+H^+$] 252.1359, found: 252.1365.

Methyl *N*-(ethoxy(undecyl)phosphoryl)glycinate **4b**

Diethyl undecylphosphonate **1b** (3.7 mmol, 1 equiv.), oxalyl chloride (1.32 equiv.) and glycine methyl ester hydrochloride (5.5 mmol, 1.5 equiv.) were used as starting products for general procedure B. Together with the amino acid, 1.6 equiv. of triethylamine (5.9 mmol) was added to neutralize the hydrochloride salt. The crude product was purified using normal phase automatic flash chromatography (SiO_2, EtOAc:hexane 3:1 v:v, gradient MeOH 0-7 v%), after which 940 mg of product **4b** was obtained as a white powder in a yield of 76%.

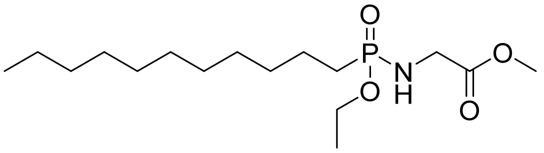

Melting point: 60 °C; **^{1}H NMR** (400 MHz, $CDCl_3$): δ = 0.88 (t, *J* = 6.8 Hz, 3H, $C\underline{H}_3CH_2CH_2$), 1.23 – 1.42 (m, 19H, $CH_3(C\underline{H}_2)_8$ and $C\underline{H}_3CH_2O$), 1.51 – 1.79 (m, 4H, $C\underline{H}_2CH_2P$ and $C\underline{H}_2P$), 2.84-2.94 (m, 1H, $N\underline{H}CH_2$), 3.72-3.80 (m, 5H, $COOC\underline{H}_3$ and $NHC\underline{H}_2$), 3.93 – 4.17 (m, 2H, $CH_3C\underline{H}_2O$) ppm; **^{13}C NMR** (100.6 MHz, $CDCl_3$): δ = 14.1 (s, $\underline{C}H_3CH_2CH_2$), 16.4 (d, *J* = 6.6 Hz, $\underline{C}H_3CH_2O$), 22.4 (d, *J* = 4.8 Hz, $\underline{C}H_2CH_2P$), 22.7 (s, $CH_3\underline{C}H_2CH_2$), 27.8 (d, *J* = 130.5 Hz, $\underline{C}H_2P$), 29.2, 29.3, 29.4, 29.6 (s, $CH_3CH_2CH_2(\underline{C}H_2)_5$), 30.7 (d, *J* = 17.1 Hz, $\underline{C}H_2(CH_2)_2P$), 31.9 (s, $CH_3CH_2\underline{C}H_2$), 42.5 (s, $NH\underline{C}H_2$), 52.3 (s, $COO\underline{C}H_3$), 59.8 (d, *J* = 6.9 Hz, $CH_3\underline{C}H_2O$), 172.0 (d, *J* = 5.7 Hz, $\underline{C}OO$) ppm; **^{31}P NMR** (161.9 MHz, $CDCl_3$): δ = 34.86 ($\underline{P}$) ppm; **IR** (ATR, cm^{-1}): ν = 3197 (N-H), 2914 (C-H), 2848 (C-H), 1749 (C=O), 1463, 1197 (ν_{max}), 1159 (C-O), 1041, 945, 866, 802, 769; **MS** (ES^+): *m/z* (%): 336 ($M+H^+$, 100), 671 ($2M+H^+$, 75), 693 ($2M+Na^+$, 35); **EI-MS** *m/z* (rel. int.): 335 [M] (16), 276 (100), 248 (25), 208 (23), 195 (27), 163 (61), 135 (25), 108 (30), 90 (9), 65 (8), 43 (10); **HRMS** (ESI^+): m/z calculated: $C_{16}H_{35}NO_4P^+$ [$M+H^+$] 336.2298, found: 336.2300.

Methyl *N*-(ethoxy(pentyl)phosphoryl)-L-prolinate **4c**

Diethyl pentylphosphonate **1a** (3.7 mmol, 1 equiv.), oxalyl chloride (1.98 equiv.) and L-proline methyl ester hydrochloride (5.5 mmol, 1.5 equiv.) were used as starting products for general procedure B. Together with the amino acid, 1.6 equiv. of triethylamine (5.9 mmol) was added to neutralize the hydrochloride salt. The crude mixture was purified via normal phase automatic flash chromatography (SiO_2, EtOAc:hexane 3:1 v:v, gradient MeOH 0-7 v%), after which 800 mg of product **4c** was obtained as a yellow oil in a yield of 75%.

Spectral data derived from the mixture of diastereomers (dr= 47/53).

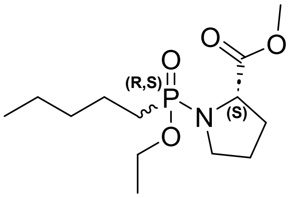

^{1}H NMR (400 MHz, $CDCl_3$): δ = 0.86 – 0.93 (m, 6H, 2 x $C\underline{H}_3CH_2CH_2$), 1.25 – 1.41 (m, 14H, 2 x $CH_3(C\underline{H}_2)_2$ and 2 x $C\underline{H}_3CH_2O$), 1.43 – 2.07 (m, 14H, 2 x $C\underline{H}_2CH_2P$, 2 x $C\underline{H}_2P$, 2 x $C\underline{H}_2CH_2CHN$ and 2 x $C\underline{H}_aH_bCHN$), 2.09 – 2.24 (m, 2H, 2 x $CH_a\underline{H}_bCHN$), 3.14 – 3.31 (m, 4H, 2 x $C\underline{H}_2N$), 3.69 – 3.73 (m, 6H, 2 x $COOC\underline{H}_3$), 3.79 – 3.87 (m, 1H, $CH_3C\underline{H}_2O$), 4.02 – 4.20 (m, 3H, $CH_3C\underline{H}_2O$), 4.25 – 4.30 (m, 1H, $C\underline{H}N$), 4.35 – 4.42 (m, 1H, $C\underline{H}N$) ppm; **^{13}C NMR** (100.6 MHz, $CDCl_3$): δ = 14.0 (s, $\underline{C}H_3CH_2CH_2$), 14.0 (s, $\underline{C}H_3CH_2CH_2$), 16.4 (d, *J* = 4.3 Hz, $\underline{C}H_3CH_2O$), 16.5 (d, *J* = 5.1 Hz, $\underline{C}H_3CH_2O$), 21.8 (d, *J* = 5.0 Hz, $\underline{C}H_2CH_2P$), 21.8 (d, *J* = 5.0 Hz, $\underline{C}H_2CH_2P$), 22.3 (s, $CH_3\underline{C}H_2CH_2$), 25.4 (d, *J* = 2.9 Hz, $\underline{C}H_2CH_2CHN$), 25.4 (d, *J* = 3.7 Hz, $\underline{C}H_2CH_2CHN$), 25.9 (d, *J* = 131.3 Hz, $\underline{C}H_2P$), 26.7 (d, *J* = 130.6 Hz, $\underline{C}H_2P$), 31.2 (d, *J* = 6.6 Hz, $\underline{C}H_2CHN$), 31.4 (d, *J* = 7.3 Hz, $\underline{C}H_2CHN$), 33.0 (d, *J* = 16.9 Hz, $CH_3CH_2\underline{C}H_2$), 33.1 (d, *J* = 17.6 Hz, $CH_3CH_2\underline{C}H_2$), 46.4 (d, *J* = 5.1 Hz, CH_2N), 46.6 (d, *J* = 5.1 Hz, CH_2N), 52.1 (s, $COO\underline{C}H_3$), 52.1 (s, $COO\underline{C}H_3$), 59.2 (d, *J* = 6.7 Hz, $CH_3\underline{C}H_2O$), 59.7 (d, *J* = 4.4 Hz, $\underline{C}HN$), 59.8 (d, *J* = 5.1 Hz, $\underline{C}HN$), 59.8 (d, *J* = 7.3 Hz, $CH_3\underline{C}H_2O$), 174.8 (s, $\underline{C}OO$), 175.1 (s, $\underline{C}OO$) ppm; **^{31}P NMR** (161.9 MHz, $CDCl_3$): δ = 33.06 ($\underline{P}$), 33.82 ($\underline{P}$) ppm; **IR** (ATR, cm^{-1}): ν = 2953 (C-H), 1745 (C=O), 1444, 1205, 1147 (C-O), 1087, 1031, 1020 (ν_{max}), 947, 761; **MS** (ES^+): *m/z* (%): 292 ($M+H^+$, 100), 583 ($2M+H^+$, 16), 605 ($2M+Na^+$, 11); **EI-MS** *m/z* (rel. int.): 291 [M] (1), 232 (100), 204 (30), 135 (7), 70 (2); **HRMS** (ESI^+): m/z calculated: $C_{13}H_{27}NO_4P^+$ [$M+H^+$] 292.1672, found: 292.1676.

Methyl *N*-(isopropoxy(pentyl)phosphoryl)sarcosinate **4d**

Diisopropyl pentylphosphonate **1c** (3.9 mmol, 1 equiv.), oxalyl chloride (1.32 equiv.) and sarcosine methyl ester hydrochloride (5.9 mmol, 1.5 equiv.) were used as starting products for general procedure B. Together with the amino acid, 1.6 equiv. of triethylamine (6.2 mmol) was added to neutralize the hydrochloride salt. The crude mixture was purified via normal phase automatic flash chromatography (SiO_2, 0-75 v% (EtOAc + 5v% Et_3N) in hexane), after which 600 mg of product **4d** was obtained as a pale yellow oil in a yield of 55%.

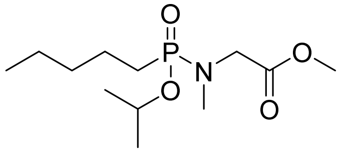

^{1}H NMR (400 MHz, $CDCl_3$): δ = 0.90 (t, *J* = 7.1 Hz, 3H, $C\underline{H}_3CH_2CH_2$), 1.22 – 1.41 (m, 10H, $CH_3(C\underline{H}_2)_2$ and $(C\underline{H}_3)_2CHO$), 1.49 – 1.64 (m, 2H, $C\underline{H}_2CH_2P$), 1.64 – 1.82 (m, 2H, $C\underline{H}_2P$), 2.66 (d, *J* = 8.4 Hz, 3H, $NC\underline{H}_3$), 3.72 (s, 3H, $COOC\underline{H}_3$), 3.89 (d, *J* = 9.9 Hz, 2H, $NC\underline{H}_2$), 4.72 (dxhept, *J* = 8.8, 6.2 Hz, 1H, $C\underline{H}O$) ppm; **^{13}C NMR** (100.6 MHz, $CDCl_3$): δ = 14.0 (s, $\underline{C}H_3CH_2CH_2$), 21.9 (d, *J* = 4.6 Hz, $\underline{C}H_2CH_2P$), 22.4 (d, *J* = 1.3 Hz, $CH_3\underline{C}H_2CH_2$), 23.6 (d, *J* = 5.9 Hz, $\underline{C}H_3CHO$), 24.4 (d, *J* = 3.7 Hz, $\underline{C}H_3CHO$), 26.2 (d, *J* = 132.7 Hz, $\underline{C}H_2P$), 33.0 (d, *J* = 17.0 Hz, $CH_3CH_2\underline{C}H_2$), 34.2 (d, *J* = 4.8 Hz, $NC\underline{H}_3$), 50.3 (d, *J* = 4.4 Hz, $NC\underline{H}_2$), 51.9 (s, $COO\underline{C}H_3$), 68.2 (d, *J* = 7.1 Hz, $CH_3\underline{C}HO$), 171.7 (d, *J* = 1.9 Hz, $\underline{C}$=O) ppm; **^{31}P NMR** (161.9 MHz, $CDCl_3$): δ = 35.19 ($\underline{P}$) ppm; **IR** (ATR, cm^{-1}): ν = 2955 (C-H), 1755 (C=O), 1665, 1206, 983, 950, 727 ($ν_{max}$); **MS** (ES^+): *m/z* (%): 280 ($M+H^+$, 100), 581 ($2M+Na^+$, 15); **EI-MS** *m/z* (rel. int.): 279 [M] (11), 220 (32), 178 (100), 44 (21); **HRMS** (ESI^+): m/z calculated: $C_{12}H_{27}NO_4P^+$ [$M+H^+$] 280.1672, found: 280.1672.

Dimethyl *N*-(ethoxy(pentyl)phosphoryl)-L-aspartate **4e**

Diethyl pentylphosphonate **1a** (3.7 mmol, 1 equiv.), oxalyl chloride (1.98 equiv.) and dimethyl L-aspartate hydrochloride (5.5 mmol, 1.5 equiv.) were used as starting products for general procedure B. Together with the amino acid, 1.6 equiv. of triethylamine (5.9 mmol) was added to neutralize the hydrochloride salt. The crude product was purified using normal phase automated column chromatography (SiO_2, 55% - 75% ethyl acetate in hexane), after which, 520 mg of product **4e** was obtained as an orange oil with a yield of 44%.

Spectral data derived from the mixture of diastereomers (dr = 51/49).

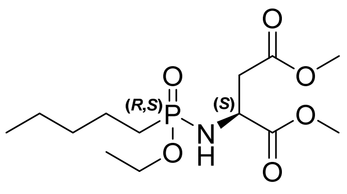

^{1}H NMR (400 MHz, $CDCl_3$): δ = 0.89, 0.90 (2 x t, *J* = 7.0, 3H, $C\underline{H}_3CH_2CH_2$), 1.23 – 1.45 (m, 7H, $CH_3(C\underline{H}_2)_2$ and $C\underline{H}_3CH_2O$), 1.49 – 1.85 (m, 4H, $C\underline{H}_2CH_2P$ and $C\underline{H}_2P$), 2.73 – 2.83 (m, 1H, $C\underline{H_a}H_bCHNH$), 2.92 – 3.02 (m, 1H, $CH_a\underline{H_b}CHNH$), 3.45 (t, *J* = 10.6 Hz, 1H, $CHN\underline{H}$), 3.70 (2 x s, 3H, $CH_2COOC\underline{H}_3$), 3.75, 3.76 (2 x s, 3H, $CHCOOC\underline{H}_3$), 3.87 – 4.17 (m, 2H, $CH_3C\underline{H}_2O$), 4.22 – 4.38 (m, 1H, $C\underline{H}NH$) ppm; **^{13}C NMR** (100.6 MHz, $CDCl_3$): δ = 13.9 (s, 2 x $\underline{C}H_3CH_2CH_2$), 16.4 (app t, *J* = 6.7 Hz, 2 x $\underline{C}H_3CH_2O$), 22.0 (d, *J* = 5.1 Hz, $\underline{C}H_2CH_2P$), 22.2 (d, *J* = 5.1 Hz, $\underline{C}H_2CH_2P$), 22.3 (s, 2 x $CH_3\underline{C}H_2CH_2$), 28.3 (d, *J* = 129.1 Hz, $\underline{C}H_2P$), 28.4 (d, *J* = 132.0 Hz, $\underline{C}H_2P$), 32.9 (d, *J* = 17.3 Hz, $CH_3CH_2\underline{C}H_2$), 33.0 (d, *J* = 17.6 Hz, $CH_3CH_2\underline{C}H_2$), 39.1 (d, *J* = 4.0 Hz, $\underline{C}H_2CHNH$), 39.1 (d, *J* = 3.6 Hz, $\underline{C}H_2CHNH$), 50.3 (s, $\underline{C}HNH$), 50.6 (s, $\underline{C}HNH$), 52.0 (s, 2 x $CH_2COO\underline{C}H_3$), 52.8 (s, 2 x $CHCOO\underline{C}H_3$), 59.8 (d, *J* = 7.0 Hz, $CH_3\underline{C}H_2O$), 60.2 (d, *J* = 6.8 Hz, $CH_3\underline{C}H_2O$), 171.4 (s, $CH_2\underline{C}OOCH_3$), 171.6 (s, $CH_2\underline{C}OOCH_3$), 172.8 (2 x d, *J* = 4.2 Hz, 2 x $CH\underline{C}OOCH_3$) ppm; **^{31}P NMR** (161.9 MHz, $CDCl_3$): δ = 33.76 ($\underline{P}$), 34.68 ($\underline{P}$) ppm; **IR** (ATR, cm^{-1}): ν = 2955 (C-H), 2932 (C-H), 2873 (C-H), 1736 (C=O), 1732 (C=O), 1437,

1275, 1198, 1167 (C-O, ν_{max}), 1036, 962; **MS** (ES$^+$): *m/z* (%): 324 (M+H$^+$, 100), 669 (2M+Na$^+$, 17); **EI-MS** *m/z* (rel. int.): 323 [M] (4), 292 (4), 264 (100), 232 (35), 204 (50), 164 (8), 135 (18), 104 (10), 70 (5), 43 (8); **HRMS** (ESI$^+$): m/z calculated: $C_{13}H_{27}NO_6P^+$ [M+H$^+$] 324.1571, found: 324.1574.

Methyl *N*-(benzyl(ethoxy)phosphoryl)azetidine-3-carboxylate **4f**

Diethyl benzylphosphonate **1d** (3.9 mmol, 1 equiv.) and methyl azetidine-3-carboxylate hydrochloride (5.9 mmol, 1.5 equiv.) were used as starting products for general procedure B. The chlorination step was executed with 1.32 equiv. of oxalyl chloride and a catalytic amount of DMF (5 drops). Together with the amino acid, 1.6 equiv. of triethylamine (6.2 mmol) was added to neutralize the hydrochloride salt. The crude product was purified using normal phase automated column chromatography (SiO_2, 0% – 40% (EtOAc:iPA, 3:1 v:v) in hexane), after which, 790 mg of product **4f** was obtained as an orange oil with a yield of 68%.

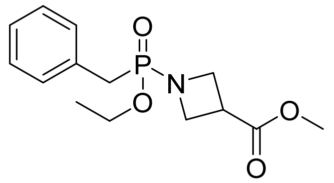

^{1}H NMR (400 MHz, $CDCl_3$): δ = 1.30 (t, *J* = 7.0 Hz, 3H, $C\underline{H}_3CH_2O$), 3.14 (d, *J* = 20.2 Hz, 2H, $C\underline{H}_2P$), 3.35 (ttd, *J* = 8.8, 6.5, 2.3 Hz, 1H, $C\underline{H}COO$), 3.71 (s, 3H, $COOC\underline{H}_3$), 3.77 – 3.98 (m, 4H, $C\underline{H}_2NC\underline{H}_2$), 3.99 – 4.15 (m, 2H, $CH_3C\underline{H}_2O$), 7.19 – 7.38 (m, 5H, 5 x $C\underline{H}_{arom}$) ppm; **^{13}C NMR** (100.6 MHz, $CDCl_3$): δ = 16.4 (d, *J* = 6.1 Hz, $\underline{C}H_3CH_2O$), 33.7 (d, *J* = 124.1 Hz, $\underline{C}H_2P$), 34.1 (d, *J* = 16.8 Hz, $\underline{C}HCOO$), 51.2 (2 x d, *J* = 7.0 Hz, $\underline{C}H_2N\underline{C}H_2$), 52.1 (s, $COO\underline{C}H_3$), 60.7 (d, *J* = 7.0 Hz, $CH_3\underline{C}H_2O$), 126.8 (d, *J* = 3.4 Hz, $\underline{C}H_{arom,para}$), 128.5 (d, *J* = 3.0 Hz, 2 x $\underline{C}H_{arom,meta}$), 129.8 (d, *J* = 6.2 Hz, 2 x $\underline{C}H_{arom,ortho}$), 131.9 (d, *J* = 8.5 Hz, $\underline{C}_{arom,quat}$), 172.9 (d, *J* = 2.9 Hz, $\underline{C}OO$) ppm; **^{31}P NMR** (161.9 MHz, $CDCl_3$): δ = 27.33 ($\underline{P}$) ppm; **IR** (ATR, cm^{-1}): ν = 2980 (C-H), 2889 (C-H), 1732 (C=O), 1437, 1200 (C-O), 1026 (ν_{max}), 945, 698, 519; **MS** (ES$^+$): *m/z* (%): 298 (M+H$^+$, 100), 595 (2M+H$^+$, 55), 617 (2M+Na$^+$, 35); **EI-MS** *m/z* (rel. int.): 297 [M] (50), 266 (11), 206 (36), 182 (18), 152 (68), 124 (21), 114 (57), 91 (100), 55 (29); **HRMS** (ESI$^+$): m/z calculated: $C_{14}H_{21}NO_4P^+$ [M+H$^+$] 298.1203, found: 298.1206.

Methyl *N*-(ethoxy(pentyl)phosphoryl)-L-serinate **4g**

Diethyl pentylphosphonate **1a** (3.9 mmol, 1 equiv.), oxalyl chloride (1.32 equiv.) and L-serine methyl ester hydrochloride (5.9 mmol, 1.5 equiv.) were used as starting products for general procedure B. Together with the amino acid, 1.6 equiv. of triethylamine (6.2 mmol) was added to neutralize the hydrochloride salt. The crude product was purified using normal phase automated column chromatography (SiO_2, 0% - 40% (EtOAc:iPA, 3:1 v:v) in hexane), after which, 650 mg of product **4g** was obtained as a yellowish oil with a yield of 59%.

Spectral data derived from the mixture of diastereomers (dr = 52/48).

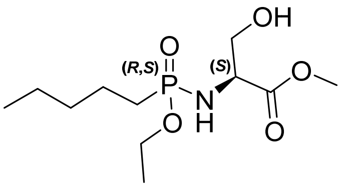

^{1}H NMR (400 MHz, $CDCl_3$): δ = 0.89 (t, *J* = 6.3 Hz, 3H, $C\underline{H}_3CH_2CH_2$), 1.25 – 1.38 (m, 7H, $CH_3(C\underline{H}_2)_2$ and $C\underline{H}_3CH_2O$), 1.52 – 1.64 (m, 2H, $C\underline{H}_2CH_2P$), 1.67 – 1.81 (m, 2H $C\underline{H}_2P$), 3.77 (s, 3H, $COOC\underline{H}_3$), 3.80 – 3.90 (m, 3H, $C\underline{H}_2OH$ and $N\underline{H}CH$), 3.95 – 4.17 (m, 3H, $NHC\underline{H}$ and $CH_3C\underline{H}_2O$) ppm; **^{13}C NMR** (100.6 MHz, $CDCl_3$): δ = 13.9 (s, 2 x $\underline{C}H_3CH_2CH_2$), 16.4 (d, *J* = 6.9 Hz, $CH_3\underline{C}H_2O$), 16.4 (d, *J*

= 6.7 Hz, $CH_3\underline{C}H_2O$), 22.0 (d, *J* = 5.1 Hz, $\underline{C}H_2CH_2P$), 22.1 (d, *J* = 5.1 Hz, $\underline{C}H_2CH_2P$), 22.3 (s, $CH_3\underline{C}H_2CH_2$), 27.8 (d, *J* = 131.0 Hz, $\underline{C}H_2P$), 27.8 (d, *J* = 131.0 Hz, $\underline{C}H_2P$), 32.9 (d, *J* = 17.6 Hz, $CH_3CH_2\underline{C}H_2$), 32.9 (d, *J* = 17.6 Hz, $CH_3CH_2\underline{C}H_2$), 52.6 (s, $COO\underline{C}H_3$), 56.4 (s, $\underline{C}HNH$), 56.5 (s, $\underline{C}HNH$), 60.2 (d, *J* = 6.9 Hz, $CH_3\underline{C}H_2O$), 65.1 (app t, *J* = 3.3 Hz, $C\underline{H}_2OH$), 65.3 (app t, *J* = 4.4 Hz, $C\underline{H}_2OH$), 172.5 (s, $\underline{C}OO$) ppm; **^{31}P NMR** (161.9 MHz, $CDCl_3$): δ = 35.55 ($\underline{P}$), 35.59 ($\underline{P}$) ppm; **IR** (ATR, cm^{-1}): ν = 3289 (N-H & O-H), 2955 (C-H), 1746 (C=O), 1439, 1194 (C-O), 1032 (ν_{max}), 955; **MS** (ES^+): *m/z* (%): 282 ($M+H^+$, 100), 585 ($2M+Na^+$, 18); **EI-MS** *m/z* (rel. int.): 281 [M] (1), 176 (100), 106 (11); **HRMS** (ESI^+): m/z calculated: $C_{11}H_{25}NO_5P^+$ [$M+H^+$] 282.1465, found: 282.1468.

Methyl *N*-(ethoxy(pentyl)phosphoryl)-L-tyrosinate **4h**

Diethyl pentylphosphonate **1a** (3.7 mmol, 1 equiv.), oxalyl chloride (1.98 equiv.) and L-tyrosine methyl ester hydrochloride (5.5 mmol, 1.5 equiv.) were used as starting products for general procedure B. Together with the amino acid, 1.6 equiv. of triethylamine (5.9 mmol) was added to neutralize the hydrochloride salt. The crude product was purified using normal phase automated column chromatography (SiO_2, 50% - 75% ethyl acetate in hexane) after which, 805 mg of product **4h** was obtained as a viscous orange oil with a yield of 61%.

Spectral data derived from the mixture of diastereomers (dr = 54/46).

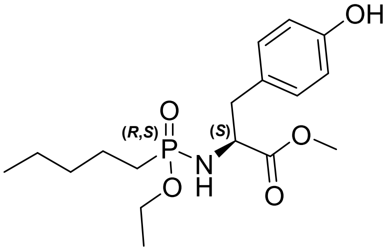

^{1}H NMR (400 MHz, $CDCl_3$): δ = 0.85, 0.86 (2 x t, *J* = 6.9 Hz, 3H, $C\underline{H}_3CH_2CH_2$), 1.13, 1.19 (2 x t, *J* = 7.1 Hz, 3H, $C\underline{H}_3CH_2O$), 1.22 – 1.80 (m, 8H, $CH_3(C\underline{H}_2)_4P$), 2.65 – 2.92 (m, 2H, $C\underline{H}_aH_bCHN$ and $N\underline{H}CH$), 2.97 – 3.19 (m, 1H, $CH_a\underline{H}_bCHN$), 3.53 – 3.97 (m, 5H, $CH_3C\underline{H}_2O$ and $COOC\underline{H}_3$), 4.04 – 4.28 (m, 1H, $C\underline{H}NH$), 6.72 – 6.82 (m, 2H, 2 x $HOC_qC\underline{H}$), 6.99 – 7.04 (m, 2H, 2 x $CH_2C_qC\underline{H}$), 8.15 (br s, 1H, OH) ppm; **^{13}C NMR** (100.6 MHz, $CDCl_3$): δ = 14.0 (s, 2 x $\underline{C}H_3CH_2CH_2$), 16.4, 16.5 (2 x d, *J* = 6.8 Hz, 2 x $\underline{C}H_3CH_2O$), 22.0 (app t, *J* = 4.7 Hz, 2 x $\underline{C}H_2CH_2P$), 22.3 (d, *J* = 3.7 Hz, 2 x $CH_3\underline{C}H_2CH_2$), 27.6 (d, *J* = 130.6, $\underline{C}H_2P$), 28.0 (d, *J* = 131.3, $\underline{C}H_2P$), 32.9 (d, *J* = 17.6 Hz, $\underline{C}H_2CH_2CH_2P$), 32.9 (d, *J* = 17.4 Hz, $\underline{C}H_2CH_2CH_2P$), 40.3, 40.4 (2 x d, *J* = 5.1 Hz, 2 x $\underline{C}H_2CHNH$), 52.4 (2 x s, 2 x $COO\underline{C}H_3$), 55.5 (s, $\underline{C}HNH$), 55.7 (s, $\underline{C}HNH$), 59.9, 60.0 (2 x d, *J* = 11.8, 2 x $CH_3\underline{C}H_2O$), 115.7 (s, 4 x $HOC_q\underline{C}H$), 127.0, 127.1 (s, 2 x $CH_2\underline{C_q}CH$), 130.5 (s, 4 x $CH_2C_q\underline{C}H$), 156.4, 156.5 (s, 2 x $HO\underline{C_q}$), 174.0 (d, *J* = 2.9 Hz, $\underline{C}OO$), 174.2 (d, *J* = 2.2 Hz, $\underline{C}OO$) ppm; **^{31}P NMR** (161.9 MHz, $CDCl_3$): δ = 34.51 ($\underline{P}$), 34.89 ($\underline{P}$) ppm; **IR** (ATR, cm^{-1}): ν = 3150 (N-H), 2953 (C-H), 2918 (C-H), 2851 (C-H), 1772 (C=O), 1518, 1454, 1375, 1169, 1028 (C-O, ν_{max}), 951; **MS** (ES^+): *m/z* (%): 358 ($M+H^+$, 100), 715 ($2M+H^+$, 19), 737 ($2M+Na^+$, 25); **HRMS** (ESI^+): m/z calculated: $C_{17}H_{29}NO_5P^+$ [$M+H^+$] 358.1778, found: 358.1782.

Ethyl *N*,*N*-dipropyl-*P*-pentylphosphonamidate **4j**

Diethyl pentylphosphonate **1a** (3.7 mmol, 1 equiv.), oxalyl chloride (2.64 equiv.) and distilled di-*n*-propylamine (5.5 mmol, 1.5 equiv.) were used as starting products for general procedure B. The crude product was purified using normal phase automatic flash chromatography (SiO_2, EtOAc:hexane 3:1 v:v, 5% Et_3N, gradient MeOH 0-7 v%) after which 600 mg of product **4j** was obtained as a pale yellow oil in a yield of 62%.

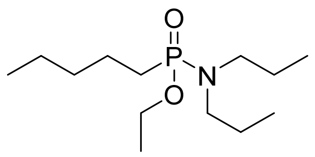

^{1}H NMR (400 MHz, $CDCl_3$): δ = 0.78 – 0.92 (m, 9H, $C\underline{H}_3CH_2CH_2CH_2$ and 2 x $C\underline{H}_3CH_2CH_2N$), 1.14 – 1.37 (m, 7H, $CH_3(C\underline{H}_2)_2CH_2$ and $C\underline{H}_3CH_2O$), 1.40 – 1.75 (m, 8H, $C\underline{H}_2CH_2P$, $C\underline{H}_2P$ and 2 x $CH_3C\underline{H}_2CH_2N$), 2.84 – 2.95 (m, 4H, 2 x $CH_3CH_2C\underline{H}_2N$), 3.71 – 4.08 (m, 2H, $CH_3C\underline{H}_2O$) ppm; **^{13}C NMR** (100.6 MHz, $CDCl_3$): δ = 11.4 (s, 2 x $\underline{C}H_3CH_2CH_2N$), 14.0 (s, 2 x $\underline{C}H_3CH_2CH_2CH_2$), 16.4 (d, *J* = 6.9 Hz, $CH_3\underline{C}H_2O$), 22.1 – 22.5 (m, $\underline{C}H_2CH_2P$, $CH_3\underline{C}H_2CH_2$ and 2 x $CH_3\underline{C}H_2CH_2N$), 26.9 (d, *J* = 131.2 Hz, $\underline{C}H_2P$), 33.1 (d, *J* = 17.2 Hz, $CH_3CH_2\underline{C}H_2$), 46.9 (d, *J* = 4.2 Hz, 2 x $CH_3CH_2\underline{C}H_2N$), 58.9 (d, *J* = 6.7 Hz, $CH_3\underline{C}H_2O$) ppm; **^{31}P NMR** (161.9 MHz, $CDCl_3$): δ = 36.38 ($\underline{P}$) ppm; **IR** (ATR, cm^{-1}): ν = 2960 (C-H), 2873 (C-H), 1460, 1381, 1226, 1209, 1035 (ν_{max}), 947, 821, 729; **MS** (ES^+): *m/z* (%): 264 ($M+H^+$, 32), 527 ($2M+H^+$, 100); **EI-MS** *m/z* (rel. int.): 263 [M] (3), 234 (100), 206 (16), 190 (9), 164 (11), 135 (22), 100 (9), 72 (7); **HRMS** (ESI^+): m/z calculated: $C_{13}H_{31}NO_2P^+$ [$M+H^+$] 264.2087, found: 264.2093.

Ethyl *N*,*N*-dipropyl-*P*-undecylphosphonamidate **4k**

Diethyl undecylphosphonate **1b** (3.7 mmol, 1 equiv.), oxalyl chloride (2.64 equiv.) and distilled di-*n*-propylamine (5.5 mmol, 1.5 equiv.) were used as starting products for general procedure B. The crude product was purified using normal phase automatic flash chromatography (SiO_2, EtOAc:hexane 3:1 v:v, 5% Et_3N, gradient MeOH 0-7 v%) after which 650 mg of product **4k** was obtained as a pale yellow oil in a yield of 51 %.

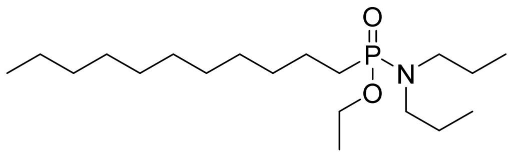

^{1}H NMR (400 MHz, $CDCl_3$): δ = 0.84 (t, *J* = 7.3 Hz, 9H, $C\underline{H}_3CH_2CH_2$ and 2 x $C\underline{H}_3CH_2CH_2N$), 1.15 – 1.39 (m, 19H, $CH_3(C\underline{H}_2)_8$ and $C\underline{H}_3CH_2O$), 1.40 – 1.74 (m, 8H, $C\underline{H}_2CH_2P$, $C\underline{H}_2P$ and 2 x $CH_3C\underline{H}_2CH_2N$), 2.82 – 2.96 (m, 4H, 2 x $CH_3CH_2C\underline{H}_2N$), 3.68 – 4.14 (m, 2H, $CH_3C\underline{H}_2O$) ppm; **^{13}C NMR** (100.6 MHz, $CDCl_3$): δ = 11.4 (s, 2 x $\underline{C}H_3CH_2CH_2N$), 14.2 (s, $\underline{C}H_3CH_2CH_2$), 16.4 (d, *J* = 6.9 Hz, $\underline{C}H_3CH_2O$), 22.2 (d, *J* = 1.8 Hz, 2 x $CH_3\underline{C}H_2CH_2N$), 22.5 (d, *J* = 4.3 Hz, $\underline{C}H_2CH_2P$), 22.8 (s, $CH_3\underline{C}H_2CH_2$), 26.9 (d, *J* = 131.3 Hz, $\underline{C}H_2P$), 29.3, 29.4, 29.5, 29.7 (s, $CH_3CH_2CH_2(\underline{C}H_2)_5$), 31.0 (d, *J* = 17.2 Hz, $\underline{C}H_2(CH_2)_2P$), 32.0 (s, $CH_3CH_2\underline{C}H_2$), 46.9 (d, *J* = 4.1 Hz, 2 x $CH_3CH_2\underline{C}H_2N$), 58.9 (d, *J* = 6.7 Hz, $CH_3\underline{C}H_2O$) ppm; **^{31}P NMR** (161.9 MHz, $CDCl_3$): δ = 36.34 ($\underline{P}$) ppm; **IR** (ATR, cm^{-1}): ν = 2959 (C-H), 2924 (C-H), 2855 (C-H), 1458, 1227, 1036 (ν_{max}), 1016, 947, 729; **MS** (ES^+): *m/z* (%): 348 ($M+H^+$, 20), 695 ($2M+H^+$, 100); **EI-MS** *m/z* (rel. int.): 347 [M] (2), 318 (100), 290 (10), 274 (7), 247 (9), 219 (5), 164 (7), 136 (9), 100 (11), 72 (5); **HRMS** (ESI^+): m/z calculated: $C_{19}H_{43}NO_2P^+$ [$M+H^+$] 348.3026, found: 348.3032.

Ethyl *P*-pentyl-*N*-((*S*)-2-oxotetrahydrofuran-3-yl)phosphonamidate **6a**

Diethyl pentylphosphonate **1a** (3.7 mmol, 1 equiv.), oxalyl chloride (1.32 equiv.) and L-homoserine lactone hydrobromide (5.5 mmol, 1.5 equiv.) were used as starting products for general procedure B. Together with the amino acid, 1.6 equiv. of triethylamine (5.9 mmol) was added to neutralize the hydrobromide salt. The crude product was purified using normal phase automated column chromatography (SiO_2, EtOAc:hexane 3:1 v:v, gradient MeOH 0-7 v%) after which, 830 mg of product **6a** was obtained as a yellowish oil with a yield of 81%.

Spectral data derived from the mixture of diastereomers (dr = 50/50).

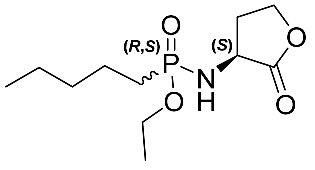

^{1}H NMR (400 MHz, $CDCl_3$): δ = 0.88 (t, *J* = 6.5 Hz, 3H, $C\underline{H}_3CH_2CH_2$), 1.22 - 1.41 (m, 7H, $CH_3(C\underline{H}_2)_2$ and $C\underline{H}_3CH_2O$), 1.52 - 1.66 (m, 2H, $C\underline{H}_2CH_2P$), 1.68 - 1.85 (m, 2H, $C\underline{H}_2P$), 2.04 - 2.21 (m, 1H, $C\underline{H}_aH_bCHN$), 2.64 - 2.75 (m, 1H, $CH_a\underline{H}_bCHN$), 2.96 - 3.05 (m, 1H, $CHN\underline{H}$), 3.98 - 4.29 (m, 4H, $C\underline{H}N$, $CH_3C\underline{H}_2O$ and $C\underline{H}_aH_bOC=O$), 4.39 (t, *J* = 8.9 Hz, 1H, $CH_a\underline{H}_bOC=O$) ppm; **^{13}C NMR** (100.6 MHz, $CDCl_3$): δ = 14.0 (s, $\underline{C}H_3CH_2CH_2$), 16.4 (app. t, *J* = 5.8 Hz, $\underline{C}H_3CH_2O$), 22.1 (d, *J* = 10.3 Hz, $\underline{C}H_2CH_2P$), 22.1 (d, *J* = 10.3 Hz, $\underline{C}H_2CH_2P$), 22.2 (s, $CH_3\underline{C}H_2CH_2$), 28.3 (d, *J* = 131 Hz, $\underline{C}H_2P$), 28.4 (d, *J* = 128 Hz, $\underline{C}H_2P$), 32.6 (s, $\underline{C}H_2CHN$), 32.8 (s, $\underline{C}H_2CHN$), 32.8 (d, *J* = 16.9 Hz, $CH_3CH_2\underline{C}H_2$), 32.9 (d, *J* = 16.9 Hz, $CH_3CH_2\underline{C}H_2$), 50.3 (s, $\underline{C}HNH$), 50.6 (s, $\underline{C}HNH$), 60.0 (d, *J* = 6.6 Hz, $CH_3\underline{C}H_2O$), 60.1 (d, *J* = 6.6 Hz, $CH_3\underline{C}H_2O$), 65.2 (s, $\underline{C}H_2OC=O$), 65.3 (s, $\underline{C}H_2OC=O$), 176.6 (app. t, *J* = 7 Hz, $\underline{C}=O$) ppm; **^{31}P NMR** (161.9 MHz, $CDCl_3$): δ = 34.59 ($\underline{P}$), 35.01 ($\underline{P}$) ppm; **IR** (ATR, cm^{-1}): ν = 3180 (N-H), 2929 (C-H), 1776 (C=O), 1452, 1375, 1199, 1149 (C-O, ν_{max}), 1022, 948, 758; **MS** (ES^+): *m/z* (%): 264 ($M+H^+$, 100), 527 ($2M+H^+$, 17), 549 ($2M+Na^+$, 15); **EI-MS** *m/z* (rel. int.): 263 [M] (53), 190 (99), 181 (40), 164 (25), 162 (30), 135 (38), 108 (25), 57 (26), 56 (10); **HRMS** (ESI^+): m/z calculated: $C_{11}H_{23}NO_4P^+$ [$M+H^+$] 264.1359, found: 264.1351.

Ethyl *P*-heptyl-*N*-((*S*)-2-oxotetrahydrofuran-3-yl)phosphonamidate **6b**

Diethyl heptylphosphonate **1e** (8.8 mmol, 1 equiv.), oxalyl chloride (1.32 equiv.) and L-homoserine lactone hydrobromide (13.0 mmol, 1.5 equiv.) were used as starting products for general procedure B. Together with the amino acid, 1.6 equiv. of triethylamine (13.9 mmol) was added to neutralize the hydrobromide salt. The crude product was purified using normal phase automated column chromatography (SiO_2, EtOAc:hexane 3:1 v:v, gradient MeOH 0-7 v%) after which, 880 mg of product **6b** was obtained as a yellowish oil with a yield of 35%.

Spectral data derived from the mixture of diastereomers (dr = 41/59).

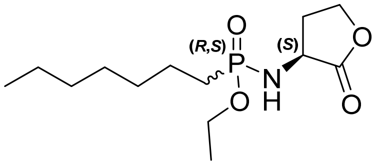

^{1}H NMR (400 MHz, $CDCl_3$): δ = 0.88 (t, *J* = 6.5 Hz, 3H, $C\underline{H}_3CH_2CH_2$), 1.22 - 1.41 (m, 11H, $CH_3(C\underline{H}_2)_4$ and $C\underline{H}_3CH_2O$), 1.52 - 1.66 (m, 2H, $C\underline{H}_2CH_2P$), 1.68 - 1.85 (m, 2H, $C\underline{H}_2P$), 2.04 - 2.21 (m, 1H, $C\underline{H}_aH_bCHN$), 2.64 - 2.75 (m, 1H, $CH_a\underline{H}_bCHN$), 2.89 - 2.97 (m, 1H, $CHN\underline{H}$), 3.98 - 4.29 (m, 4H, $C\underline{H}N$, $CH_3C\underline{H}_2O$ and $C\underline{H}_aH_bOC=O$), 4.39 (t, *J* = 8.9 Hz, 1H, $CH_a\underline{H}_bOC=O$) ppm; **^{13}C NMR** (100.6 MHz, $CDCl_3$): δ = 14.0 (s, $\underline{C}H_3CH_2CH_2$), 16.4 (app. t, *J* = 5.8 Hz, $\underline{C}H_3CH_2O$), 22.4 (d, *J* = 9.7 Hz, $\underline{C}H_2CH_2P$), 22.4 (d, *J* =9.7 Hz, $\underline{C}H_2CH_2P$), 22.6 (s, $CH_3\underline{C}H_2CH_2$), 28.4 (d, *J* = 131 Hz, $\underline{C}H_2P$), 28.4 (d, *J* = 128 Hz, $\underline{C}H_2P$), 28.8 (s, $CH_3(CH_2)_2\underline{C}H_2$), 30.6 (d, *J* = 17.6 Hz, $\underline{C}H_2(CH_2)_2P$), 30.7 (d, *J* = 17.6 Hz, $\underline{C}H_2(CH_2)_2P$), 31.6 (s, $CH_3CH_2\underline{C}H_2$), 32.6 (s, $\underline{C}H_2CHN$), 32.9 (s, $\underline{C}H_2CHN$), 50.3 (s, $\underline{C}HN$), 50.6 (s, $\underline{C}HN$), 59.9 (d, *J* = 6.6 Hz, $CH_3\underline{C}H_2O$), 60.1 (d, *J* = 6.6 Hz, $CH_3\underline{C}H_2O$), 65.2 (s, $\underline{C}H_2OC=O$), 65.3 (s, $\underline{C}H_2OC=O$), 176.6 (app. t, *J* = 7.2 Hz, $\underline{C}=O$) ppm; **^{31}P NMR** (161.9 MHz, $CDCl_3$): δ = 34.64 ($\underline{P}$), 35.04 ($\underline{P}$) ppm; **IR** (ATR, cm^{-1}): ν = 3184 (N-H), 2932 (C-H), 2858 (C-H), 1778 (C=O), 1454, 1371, 1199, 1149 (C-O, ν_{max}), 1037, 1001, 948, 816; **MS** (ES^+): *m/z*(%): 292 ($M+H^+$, 100), 583 ($2M+H^+$, 17), 605 ($2M+Na^+$, 15); **EI-MS** *m/z* (rel. int.): 291 [M] (59), 218 (89), 209 (43), 207 (31), 176 (31), 163 (22), 125 (24), 108 (24), 57 (38), 56 (100); **HRMS** (ESI^+): m/z calculated: $C_{13}H_{27}NO_4P^+$ [$M+H^+$] 292.1672, found: 292.1683.

Ethyl *P*-nonyl-*N*-((*S*)-2-oxotetrahydrofuran-3-yl)phosphonamidate **6c**

Diethyl nonylphosphonate **1f** (8.8 mmol, 1 equiv.), oxalyl chloride (1.32 equiv.) and L-homoserine lactone hydrobromide (13.0 mmol, 1.5 equiv.) were used as starting products for general procedure B. Together with the amino acid, 1.6 equiv. of triethylamine (13.9 mmol) was added to neutralize the hydrobromide salt. The crude product was purified using normal phase automated column chromatography (SiO_2, EtOAc:hexane 3:1 v:v, gradient MeOH 0-7 v%) after which, 680 mg of product **6c** was obtained as a yellowish oil with a yield of 24%.

Spectral data derived from the mixture of diastereomers (dr= 48/52).

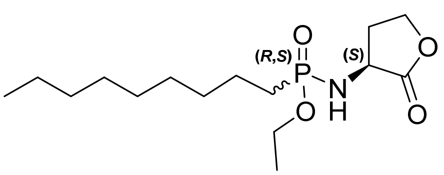

^{1}H NMR (400 MHz, $CDCl_3$): δ = 0.88 (t, *J* = 6.7 Hz, 3H, $C\underline{H}_3CH_2CH_2$), 1.22 - 1.42 (m, 15H, $CH_3(C\underline{H}_2)_6$ and $C\underline{H}_3CH_2O$), 1.54 - 1.68 (m, 2H, $C\underline{H}_2CH_2P$), 1.70 - 1.85 (m, 2H, $C\underline{H}_2P$), 2.05 - 2.22 (m, 1H, $C\underline{H}_aH_bCHN$), 2.66 - 2.76 (m, 1H, $CH_a\underline{H}_bCHN$), 2.78 - 2.83 (m, 1H, $CHN\underline{H}$), 3.99 - 4.29 (m, 4H, $C\underline{H}N$, $CH_3C\underline{H}_2O$ and $C\underline{H}_aH_bOC$=O), 4.40 (t, *J* = 9 Hz, 1H, $CH_a\underline{H}_bOC$=O) ppm; **^{13}C NMR** (100.6 MHz, $CDCl_3$): 14.1 (s, $\underline{C}H_3CH_2CH_2$), 16.4 (app. t, *J* = 5.8 Hz, $\underline{C}H_3CH_2O$), 22.4 (d, *J* = 9.5 Hz, $\underline{C}H_2CH_2P$), 22.4 (d, *J* = 10 Hz, $\underline{C}H_2CH_2P$), 22.7 (s, $CH_3\underline{C}H_2CH_2$), 28.4 (d, *J* = 131 Hz, $\underline{C}H_2P$), 28.4 (d, *J* = 128 Hz, $\underline{C}H_2P$), 29.2, 29.3, 29.4 (s, $CH_3CH_2CH_2(\underline{C}H_2)_3$), 30.7 (d, *J* = 17.6 Hz, $\underline{C}H_2(CH_2)_2P$), 30.7 (d, *J* = 17.6 Hz, $\underline{C}H_2(CH_2)_2P$), 31.9 (s, $CH_3CH_2\underline{C}H_2$), 32.6 (s, $\underline{C}H_2CHN$), 32.9 (s, $\underline{C}H_2CHN$), 50.3 (s, $\underline{C}HN$), 50.6 (s, $\underline{C}HN$), 59.9 (d, *J* = 6.6 Hz, $CH_3\underline{C}H_2O$), 60.1 (d, *J* = 7.0 Hz, $CH_3\underline{C}H_2O$), 65.2 (s, $\underline{C}H_2OC$=O), 65.3 (s, $\underline{C}H_2OC$=O), 176.6 (app. t, *J* = 6.2 Hz, $\underline{C}$=O); **^{31}P NMR** (161.9 MHz, $CDCl_3$): δ = 34.65 ($\underline{P}$), 35.10 ($\underline{P}$) ppm; **IR** (ATR, cm^{-1}): ν = 3201 (N-H), 2926 (C-H), 1780 (C=O), 1600, 1448, 1219, 1151 (C-O), 1026 (ν_{max}), 947, 692; **MS** (ES^+): *m/z* (%): 320 ($M+H^+$, 100), 639 ($2M+H^+$, 12), 661 ($2M+Na^+$, 12); **EI-MS** *m/z* (rel. int.): 319 [M] (68), 246 (81), 237 (39), 220 (26), 207 (37), 176 (30), 125 (27), 108 (23), 57 (31), 56 (100); **HRMS** (ESI^+): m/z calculated: $C_{15}H_{31}NO_4P^+$ [$M+H^+$] 320.1985, found: 320.1983.

Ethyl *P*-undecyl-*N*-((*S*)-2-oxotetrahydrofuran-3-yl)phosphonamidate **6d**

Diethyl undecylphosphonate **1b** (8.8 mmol, 1 equiv.), oxalyl chloride (1.32 equiv.) and L-homoserine lactone hydrobromide (13.0 mmol, 1.5 equiv.) were used as starting products for general procedure B. Together with the amino acid, 1.6 equiv. of triethylamine (13.9 mmol) was added to neutralize the hydrobromide salt. The crude product was purified using normal phase automated column chromatography (SiO_2, EtOAc:hexane 3:1 v:v, gradient MeOH 0-7 v%) after which, 890 mg of product **6d** was obtained as a yellowish oil with a yield of 29%.

Spectral data derived from the mixture of diastereomers (dr = 50/50).

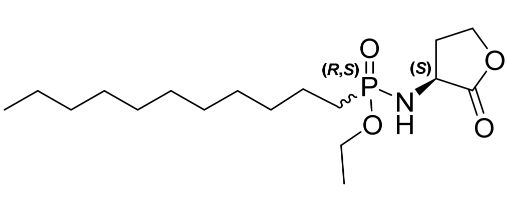

^{1}H NMR (400 MHz, $CDCl_3$): δ = 0.88 (t, *J* = 6.8 Hz, 3H, $C\underline{H}_3CH_2CH_2$), 1.21 - 1.40 (m, 19H, $CH_3(C\underline{H}_2)_8$ and $C\underline{H}_3CH_2O$), 1.55 - 1.65 (m, 2H, $C\underline{H}_2CH_2P$), 1.70 - 1.83 (m, 2H, $C\underline{H}_2P$), 2.04 - 2.21 (m, 1H, $C\underline{H}_aH_bCHN$), 2.66 - 2.75 (m, 1H, $CH_a\underline{H}_bCHN$), 2.83 - 2.88 (m, 1H, $CHN\underline{H}$), 4.00 - 4.29 (m, 4H, $C\underline{H}N$, $CH_3C\underline{H}_2O$ and $C\underline{H}_aH_bOC$=O), 4.40 (t, *J* = 8.8 Hz, 1H, $CH_a\underline{H}_bOC$=O) ppm; **^{13}C NMR** (100.6 MHz, $CDCl_3$): δ = 14.1 (s,

$\underline{C}H_3CH_2CH_2$), 16.4 (app. t, *J* = 6.2 Hz, $\underline{C}H_3CH_2O$), 22.5 (d, *J* = 9.5 Hz, $\underline{C}H_2CH_2P$), 22.5 (d, *J* =9.5 Hz, $\underline{C}H_2CH_2P$), 22.7 (s, $CH_3\underline{C}H_2CH_2$), 28.5 (d, *J* = 131 Hz, $\underline{C}H_2P$), 28.5 (d, *J* = 128 Hz, $\underline{C}H_2P$), 29.2, 29.3, 29.4, 29.6 (s, $CH_3CH_2CH_2(\underline{C}H_2)_5$), 30.7 (d, *J* = 16.8 Hz, $\underline{C}H_2(CH_2)_2P$), 30.7 (d, *J* = 16.8 Hz, $\underline{C}H_2(CH_2)_2P$), 31.9 (s, $CH_3CH_2\underline{C}H_2$), 32.7 (s, $\underline{C}H_2CHN$), 32.9 (s, $\underline{C}H_2CHN$), 50.3 (s, $\underline{C}HN$), 50.6 (s, $\underline{C}HN$), 60.0 (d, *J* = 18 Hz, $CH_3\underline{C}H_2O$), 60.0 (d, *J* = 18 Hz, $CH_3\underline{C}H_2O$), 65.2 (s, $\underline{C}H_2OC{=}O$), 65.3 (s, $\underline{C}H_2OC{=}O$), 176.7 (app. t, *J* = 6.0 Hz, $\underline{C}{=}O$) ppm; **^{31}P NMR** (161.9 MHz, $CDCl_3$): δ = 34.61 ($\underline{P}$), 35.07 ($\underline{P}$) ppm; **IR** (ATR, cm^{-1}): ν = 3142 (N-H), 2916 (C-H), 1848 (C-H), 1778 (C=O), 1465, 1381, 1163, 1031 (ν_{max}), 954; **MS** (ES$^+$): *m/z* (%): 348 (M+H$^+$, 100), 695 (2M+H$^+$, 10), 717 (2M+Na$^+$, 15); **EI-MS** *m/z* (rel. int.): 347 [M] (100), 274 (82), 265 (36), 220 (26), 207 (50), 176 (29), 125 (26), 57 (29), 56 (83); **HRMS** (ESI$^+$): m/z calculated: $C_{17}H_{35}NO_4P^+$ [M+H$^+$] 348.2298, found: 348.2291.

Isopropyl *P*-pentyl-*N*-((*S*)-2-oxotetrahydrofuran-3-yl)phosphonamidate **7a**

Diisopropyl pentylphosphonate **1c** (3.9 mmol, 1 equiv.), oxalyl chloride (1.32 equiv.) and L-homoserine lactone hydrobromide (5.9 mmol, 1.5 equiv.) were used as starting products for general procedure B. Together with the amino acid, 1.6 equiv. of triethylamine (6.2 mmol) was added to neutralize the hydrobromide salt. The crude product was purified using normal phase automated column chromatography (SiO_2, 0-75 v% (EtOAc + 5v% Et_3N) in hexane), after which, 750 mg of product **7a** was obtained as an orange oil with a yield of 69%.

Spectral data derived from the mixture of diastereomers (dr = 52/48).

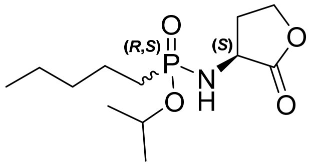

^{1}H NMR (400 MHz, $CDCl_3$): δ = 0.90 (t, *J* = 6.9 Hz, 3H, $C\underline{H}_3CH_2CH_2$), 1.20 – 1.43 (m, 10H, $CH_3(C\underline{H}_2)_2$ and $(C\underline{H}_3)_2CHO$), 1.51 – 1.85 (m, 4H, $C\underline{H}_2CH_2P$ and $C\underline{H}_2P$), 2.08 – 2.23 (m, 1H, $C\underline{H}_aH_bCHN$), 2.64 – 2.78 (m, 1H, $CH_a\underline{H}_bCHN$), 2.89 (t, *J* = 8.8 Hz, 1H, $CHN\underline{H}$), 4.07 – 4.33 (m, 2H, $C\underline{H}N$ and $C\underline{H}_aH_bOC{=}O$), 4.39 (t, *J* = 8.9 Hz, 1H, $CH_a\underline{H}_bOC{=}O$), 4.65 – 4.86 (m, 1H, $C\underline{H}O$) ppm; **^{13}C NMR** (100.6 MHz, $CDCl_3$): δ = 14.0 (s, $\underline{C}H_3CH_2CH_2$), 22.2 (d, *J* = 4.4 Hz, $\underline{C}H_2CH_2P$), 22.3 (d, *J* = 4.4 Hz, $\underline{C}H_2CH_2P$), 22.3 (s, $CH_3\underline{C}H_2CH_2$), 24.3 (d, *J* = 4.8 Hz, 2 x $\underline{C}H_3CHO$), 24.5 (d, *J* = 3.7 Hz, 2 x $\underline{C}H_3CHO$), 29.0 (d, *J* = 129.1 Hz, $\underline{C}H_2P$), 29.1 (d, *J* = 132.1 Hz, $\underline{C}H_2P$), 32.8 (d, *J* = 2.2 Hz, $\underline{C}H_2CHN$), 32.9 (d, *J* = 16.9 Hz, $CH_3CH_2\underline{C}H_2$), 33.0 (d, *J* = 16.9 Hz, $CH_3CH_2\underline{C}H_2$), 33.1 (d, *J* = 1.5 Hz, $\underline{C}H_2CHN$), 50.5 (s, $\underline{C}HN$), 50.8 (s, $\underline{C}HN$), 65.2 (s, $\underline{C}H_2OC{=}O$), 65.4 (s, $\underline{C}H_2OC{=}O$), 69.0 (d, *J* = 14.7 Hz, $CH_3\underline{C}HO$), 69.0 (d, *J* = 14.7 Hz, $CH_3\underline{C}HO$), 176.8 (d, *J* = 7.0 Hz, $\underline{C}{=}O$) ppm; **^{31}P NMR** (161.9 MHz, $CDCl_3$): δ = 33.46 ($\underline{P}$), 33.99 ($\underline{P}$) ppm; **IR** (ATR, cm^{-1}): ν = 3173 (N-H), 2932 (C-H), 1775 (C=O), 1466, 1373, 1199, 1149 (C-O), 980 (ν_{max}); **MS** (ES$^+$): *m/z* (%): 278 (M+H$^+$, 100), 555 (2M+H$^+$, 5), 577 (2M+Na$^+$, 20); **EI-MS** *m/z* (rel. int.): 277 [M] (2), 235 (72), 218 (16), 191 (29), 176 (9), 160 (100), 135 (26), 121 (14), 101 (12), 71 (14), 56 (33), 41 (33); **HRMS** (ESI$^+$): m/z calculated: $C_{12}H_{25}NO_4P^+$ [M+H$^+$] 278.1516, found: 278.1520.

Isopropyl *P*-undecyl-*N*-((*S*)-2-oxotetrahydrofuran-3-yl)phosphonamidate **7b**

Diisopropyl undecylphosphonate **1g** (8.8 mmol, 1 equiv.), oxalyl chloride (1.32 equiv.) and L-homoserine lactone hydrobromide (13.0 mmol, 1.5 equiv.) were used as starting products for general procedure B. Together with the amino acid, 1.6 equiv. of triethylamine (13.9 mmol) was added to neutralize the

hydrobromide salt. The crude product was purified using normal phase automated column chromatography (SiO_2, EtOAc:hexane 3:1 v:v, gradient MeOH 0-7 v%) after which, 810 mg of product **7b** was obtained as a yellowish oil with a yield of 25%.

Spectral data derived from the mixture of diastereomers (dr = 45/55).

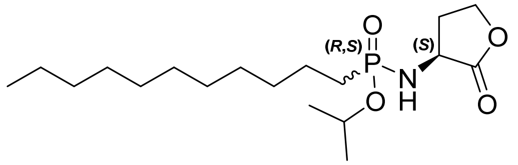

^{1}H NMR (400 MHz, $CDCl_3$): δ = 0.88 (t, *J* = 6.8 Hz, 3H, $C\underline{H}_3CH_2CH_2$), 1.19 - 1.41 (m, 22H, $CH_3(C\underline{H}_2)_8$ and $CH(C\underline{H}_3)_2$), 1.53 – 1.65 (m, 2H, $C\underline{H}_2CH_2P$), 1.66 - 1.82 (m, 2H, $C\underline{H}_2P$), 2.07 - 2.21 (m, 1H, $C\underline{H}_aH_bCHN$), 2.67 - 2.76 (m, 1H, $CH_a\underline{H}_bCHN$), 2.79 - 2.84 (m, 1H, $CHN\underline{H}$), 4.14 - 4.31 (m, 2H, $C\underline{H}_2OC{=}O$), 4.39 (t, *J* = 9.0 Hz, 1H, $C\underline{H}N$), 4.68 - 4.84 (m, 1H, $C\underline{H}(CH_3)_2$) ppm; **^{13}C NMR** (100.6 MHz, $CDCl_3$): δ = 14.2 (s, $\underline{C}H_3CH_2CH_2$), 22.5 (d, *J* = 8.8 Hz, $\underline{C}H_2CH_2P$), 22.5 (d, *J* = 8.8 Hz, $\underline{C}H_2CH_2P$), 22.8 (s, $CH_3\underline{C}H_2CH_2$), 24.2 (d, *J* = 9.5 Hz, 2 x $\underline{C}H_3CH$), 24.3 (d, *J* = 9.5 Hz, 2 x $\underline{C}H_3CH$), 29.0 (d, *J* = 132.0 Hz, $\underline{C}H_2P$), 29.0 (d, *J* = 132.0 Hz, $\underline{C}H_2P$), 29.2, 29.3, 29.4, 29.6 (s, $CH_3CH_2CH_2(\underline{C}H_2)_5$), 30.7 (d, *J* = 16.9 Hz, $\underline{C}H_2(CH_2)_2P$), 30.7 (d, *J* = 17.6 Hz, $\underline{C}H_2(CH_2)_2P$), 32.0 (s, $CH_3CH_2\underline{C}H_2$), 32.7 (s, $\underline{C}H_2CHN$), 33.0 (s, $\underline{C}H_2CHN$), 50.4 (s, $\underline{C}HN$), 50.7 (s, $\underline{C}HN$), 65.2 (s, $\underline{C}H_2OC{=}O$), 65.3 (s, $\underline{C}H_2OC{=}O$), 68.9 (d, *J* = 14.7 Hz, $(CH_3)_2\underline{C}H$), 68.9 (d, *J* = 14.7 Hz, $(CH_3)_2\underline{C}H$), 176.6 (d, *J* = 7.6 Hz, $\underline{C}{=}O$), 176.6 (d, *J* = 8.1 Hz, $\underline{C}{=}O$) ppm; **^{31}P NMR** (161.9 MHz, $CDCl_3$): δ = 33.47 ($\underline{P}$), 34.0 ($\underline{P}$) ppm; **IR** (ATR, cm^{-1}): ν = 3149 (N-H), 2916 (C-H), 2848 (C-H), 1780 (C=O), 1475, 1182 (C-O), 1031 (ν_{max}), 991, 962, 794, 767; **MS** (ES^+): *m/z* (%): 362 ($M+H^+$, 100), 723 ($2M+H^+$, 20), 745 ($2M+Na^+$, 10); **EI-MS** *m/z* (rel. int.): 361 [M] (6), 319 (100), 246 (67), 180 (24), 102 (24), 56 (74), 41 (60); **HRMS** (ESI^+): m/z calculated: $C_{18}H_{37}NO_4P^+$ [$M+H^+$] 362.2455, found: 362.2454.

Synthesis of dimethyl alkylphosphonates 11a – 11b

The synthesis of dimethyl alkylphosphonates **11** was adapted from Delorme et al.[12] To a suspension of washed sodium hydride (60 % suspension in mineral oil, 1079.6 mg, 45 mmol, 1.8 equiv.) in dry THF (100 mL), dimethyl phosphite **10** (4814.7 mg, 4012 µL, 43.8 mmol, 1.75 equiv.) was added dropwise over a period of 10 min at room temperature under a nitrogen atmosphere. After the addition was complete, the reaction mixture was stirred at 0 °C for 1 additional hour. The corresponding alkyl bromide (25 mmol, 1 equiv.) was then added and the reaction mixture was refluxed overnight. The solvent was removed and 100 mL diethyl ether was added. The solution was filtered to remove the salts, washed 3 times with water, dried over $MgSO_4$ and solvent was evaporated. The resulting product **11** was used without further purification.

Dimethyl pentylphosphonate **11a**

For this product, 1-bromopentane (25 mmol) was used as starting product. Finally, 1230 mg dimethyl pentylphosphonate **11a** was collected as a transparent oil with a yield of 27%. The ^{1}H, ^{13}C and ^{31}P NMR data were in agreement with those reported by Antczak and Montchamp.[13]

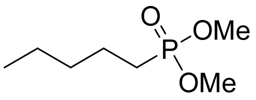

^{1}H NMR (400 MHz, $CDCl_3$): δ = 0.90 (t, *J* = 7.0 Hz, 3H, $C\underline{H}_3CH_2CH_2$), 1.23 – 1.42 (m, 4H, $CH_3(C\underline{H}_2)_2$), 1.53 – 1.80 (m, 4H, $C\underline{H}_2CH_2P$ and $C\underline{H}_2P$), 3.74 (d, *J* = 10.7 Hz, 6H, 2 x $OC\underline{H}_3$) ppm; **^{13}C NMR** (100.5 MHz, $CDCl_3$): δ = 13.8 (s, $\underline{C}H_3CH_2CH_2$), 22.0 (d, *J* = 5.4 Hz, $\underline{C}H_2CH_2P$), 22.2 (s, $CH_3\underline{C}H_2CH_2$), 24.7 (d, *J* = 140.1 Hz, $\underline{C}H_2P$), 32.8 (d, *J* = 16.9 Hz, $\underline{C}H_2(CH_2)_2P$), 52.3 (d, *J* = 6.6 Hz, 2 x $O\underline{C}H_3$) ppm; **^{31}P NMR** (161.9 MHz, $CDCl_3$): δ = 35.21 ($\underline{P}$) ppm; **IR** (ATR, cm^{-1}): ν = 2953 (C-H), 2927 (C-H), 2853 (C-H), 1468, 1348, 1240, 1055, 1024 (ν_{max}), 837; **MS** (ES^+): *m/z* (%): 181 ($M+H^+$, 100), 361 ($2M+H^+$, 59); **EI-MS** *m/z* (rel. int.): 180 [M] (1), 179 (2), 165 (6), 152 (37), 137 (20), 124 (100), 110 (18), 94 (15), 79 (9).

Dimethyl undecylphosphonate **11b**

For this product, 1-bromoundecane was used as starting product. Finally, 2.1 g dimethyl undecylphosphonate **11b** was collected as a transparent oil with a yield of 40%. The ^{1}H, ^{13}C and ^{31}P NMR data were in agreement with those reported by Li and coworkers.[14]

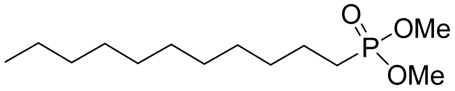

^{1}H NMR (400 MHz, $CDCl_3$): δ = 0.88 (t, *J* = 7.5 Hz, 3H, $C\underline{H}_3CH_2CH_2$), 1.21 - 1.41 (m, 16H, $CH_3(C\underline{H}_2)_8$), 1.52 – 1.66 (m, 2H, $C\underline{H}_2CH_2P$), 1.66 – 1.80 (m, 2H, $C\underline{H}_2P$), 3.73 (d, *J* = 10.7 Hz, 6H, 2 x $OC\underline{H}_3$) ppm; **^{13}C NMR** (100.6 MHz, $CDCl_3$): δ = 14.2 (s, $\underline{C}H_3CH_2CH_2$), 22.4 (d, *J* = 5.3 Hz, $\underline{C}H_2CH_2P$), 22.8 (s, $CH_3\underline{C}H_2CH_2$), 24.8 (d, *J* = 140.3 Hz, $\underline{C}H_2P$), 29.2, 29.2, 29.5, 29.5, 29.7 (s, $CH_3CH_2CH_2(\underline{C}H_2)_5$), 30.7 (d, *J* = 16.8 Hz, $\underline{C}H_2(CH_2)_2P$), 32.0 (s, $CH_3CH_2\underline{C}H_2$), 52.4 (d, *J* = 6.6 Hz, 2 x $O\underline{C}H_3$) ppm; **^{31}P NMR** (161.9 MHz, $CDCl_3$): δ = 35.25 ($\underline{P}$) ppm; **IR** (ATR, cm^{-1}): ν = 2922 (C-H), 2853 (C-H), 1460, 1244, 1057, 1026 (ν_{max}), 810; **MS** (ES^+): *m/z* (%): 265 ($M+H^+$, 100), 529 ($2M+H^+$, 99); **EI-MS** *m/z* (rel. int.): 264 [M] (2), 235 (5), 221 (6), 207 (8), 193 (9), 179 (15), 165 (15), 152 (18), 137 (33), 124 (100), 110 (43), 94 (39), 79 (22).

Synthesis of methyl *P*-alkyl-*N*-((S)-2-oxotetrahydrofuran-3-yl) phosphonamidates 8a – 8b

Under nitrogen atmosphere, oxalyl chloride (2.64 equiv., 9.7 mmol) is added dropwise to a cooled solution (0 °C) of the corresponding dimethyl *P*-alkylphosphonate **11** (1 equiv., 3.7 mmol) and 3 drops of DMF in 20 mL dry DCM. This solution is stirred for 16 hours and allowed to warm to room temperature and the conversion is monitored via ^{31}P NMR. After completion of the reaction, solvent is evaporated under reduced pressure to yield a crude mixture of phosphonochloridate. Due to the instability of the compound, the crude compound is immediately used in subsequent reactions.

The corresponding phosphonochloridate and triethylamine (1.1 equiv., 4.0 mmol) were dissolved in 30 mL dry THF under nitrogen atmosphere and left stirring for 30 min at room temperature, after which the reaction mixture was analysed using ^{31}P NMR. Triethylamine (1.6 equiv., 5.9 mmol) and L-homoserine lactone hydrobromide (1.5 equiv., 5.5 mmol) was dissolved in 30 mL dry ACN under nitrogen atmosphere, while stirring at room temperature. The solution containing the phosphonochloridate was added dropwise to the amine solution at room temperature and stirred for 1 hour (monitored with ^{31}P NMR).

Subsequently, the solids were removed via filtration, after addition of 60 mL diethyl ether and the solvent was evaporated under reduced pressure. The remaining mixture was purified via normal phase automatic flash chromatography.

Methyl *P*-pentyl-*N*-((*S*)-2-oxotetrahydrofuran-3-yl)phosphonamidate **8a**

Dimethyl pentylphosphonate **11a** was used as starting product on a scale of 2.1 mmol. The crude product was purified using normal phase automated column chromatography (SiO_2, EtOAc:hexane 3:1 v:v, gradient MeOH 0-10 v%) after which, 75 mg of product **8a** was obtained as an orange oil with a yield of 15%.

Spectral data derived from the mixture of diastereomers (dr = 50/50).

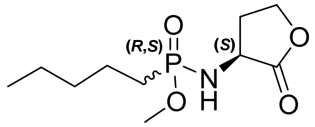

^{1}H NMR (400 MHz, $CDCl_3$): δ = 0.90 (t, *J* = 7.2 Hz, 3H, $C\underline{H}_3CH_2CH_2$), 1.30 – 1.40 (m, 4H, $CH_3(C\underline{H}_2)_2$), 1.55 - 1.70 (m, 2H, $C\underline{H}_2CH_2P$), 1.70 – 1.93 (m, 2H, $C\underline{H}_2P$), 2.09 – 2.28 (m, 1H, $C\underline{H}_aH_bCHN$), 2.65 – 2.78 (m, 1H, $CH_a\underline{H}_bCHN$), 2.79 - 2.89 (m, 1H, $CHN\underline{H}$), 3.69, 3.74 (2 x d, *J* = 11.1 Hz, 3H, $C\underline{H}_3O$), 4.08 – 4.34 (m, 2H, $C\underline{H}N$ and $C\underline{H}_aH_bOC{=}O$), 4.40 (t, *J* = 9.0 Hz, 1H, $CH_a\underline{H}_bOC{=}O$) ppm; **^{13}C NMR** (100.6 MHz, $CDCl_3$): δ = 14.0 (s, $\underline{C}H_3CH_2CH_2$), 22.1 (d, *J* =5.0 Hz, $\underline{C}H_2CH_2P$), 22.2 (d, *J* = 5.0 Hz, $\underline{C}H_2CH_2P$), 22.3 (s, $CH_3\underline{C}H_2CH_2$), 28.1 (d, *J* = 132.1 Hz, $\underline{C}H_2P$), 28.2 (d, *J* = 128.5 Hz, $\underline{C}H_2P$), 32.7 (s, 2 x $\underline{C}H_2CHN$), 32.9 (d, *J* = 17.2 Hz, $CH_3CH_2\underline{C}H_2$), 33.0 (d, *J* = 17.0 Hz, $CH_3CH_2\underline{C}H_2$), 50.4 (s, $\underline{C}HNH$), 50.6 (d, *J* = 7.3 Hz, $\underline{C}H_3O$), 50.8 (s, $\underline{C}HNH$), 50.8 (d, *J* = 7.3 Hz, $\underline{C}H_3O$), 65.3 (s, $\underline{C}H_2OC{=}O$), 65.4 (s, $\underline{C}H_2OC{=}O$), 176.7 (s, $\underline{C}{=}O$) ppm; **^{31}P NMR** (161.9 MHz, $CDCl_3$): δ = 34.51 ($\underline{P}$), 34.89 ($\underline{P}$) ppm; **IR** (ATR, cm^{-1}): ν = 3169 (N-H), 2953 (C-H), 2930 (C-H), 2872 (C-H), 1776 (C=O), 1452, 1377, 1152 (C-O, ν_{max}), 1024, 949; **MS** (ES^+, 70 eV): *m/z* (%): 250 ($M+H^+$, 100), 521 ($2M+Na^+$, 22); **EI-MS** *m/z* (rel. int.): 249 [M] (31), 207 (22), 193 (13), 176 (76), 167 (38), 149 (33), 135 (22), 121 (13), 109 (27), 94(31), 79 (56), 56 (100), 44 (51); **HRMS** (ESI^+): m/z calculated: $C_{10}H_{20}NO_4P^+$ [$M+H^+$] 250.1203, found: 250.1206.

Methyl *P*-undecyl-*N*-((*S*)-2-oxotetrahydrofuran-3-yl)phosphonamidate **8b**

Dimethyl undecylphosphonate **11b** was used as starting product on a scale of 3.7 mmol. The crude product was purified using normal phase automated column chromatography (SiO_2, EtOAc:hexane 3:1 v:v, gradient MeOH 0-10 v%) after which, 500 mg of product **8b** was obtained as a yellow solid with a yield of 41%.

Spectral data derived from the mixture of diastereomers (dr = 51/49).

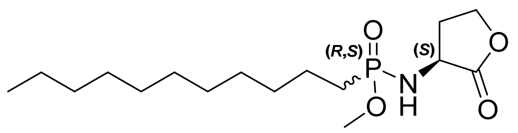

^{1}H NMR (400 MHz, $CDCl_3$): δ = 0.88 (t, *J* = 6.9 Hz, 3H, $C\underline{H}_3CH_2CH_2$), 1.18 – 1.46 (m, 16H, $CH_3(C\underline{H}_2)_8$), 1.50 – 1.68 (m, 2H, $C\underline{H}_2CH_2P$), 1.69 – 1.88 (m, 2H, $C\underline{H}_2P$), 2.15 (app pd, *J* = 11.9, 8.6 Hz, 1H, $C\underline{H}_aH_bCHN$), 2.65 – 2.78 (m, 1H, $CH_a\underline{H}_bCHN$), 2.81 – 2.90 (m, 1H, $CHN\underline{H}$), 3.69, 3.74 (2 x d, *J* = 11.1 Hz, 3H, $C\underline{H}_3O$), 4.10 – 4.32 (m, 2H, $C\underline{H}N$ and $C\underline{H}_aH_bOC{=}O$), 4.40 (t, *J* = 9.1 Hz, 1H, $CH_a\underline{H}_bOC{=}O$) ppm; **^{13}C NMR** (100.6 MHz, $CDCl_3$): δ = 14.1 (s, $\underline{C}H_3CH_2CH_2$), 22.5 (d, *J* = 9.5 Hz, $\underline{C}H_2CH_2P$), 22.5 (d, *J* =9.5 Hz, $\underline{C}H_2CH_2P$), 22.7 (s, $CH_3\underline{C}H_2CH_2$), 28.1 (d, *J* =

131.6 Hz, $\underline{C}H_2P$), 28.5 (d, *J* = 128 Hz, $\underline{C}H_2P$), 29.3, 29.4, 29.5, 29.7 (s, $CH_3CH_2CH_2(\underline{C}H_2)_5$), 30.8 (d, *J* = 16.9 Hz, $\underline{C}H_2(CH_2)_2P$), 30.9 (d, *J* = 17.6 Hz, $\underline{C}H_2(CH_2)_2P$), 31.9 (s, $CH_3CH_2\underline{C}H_2$), 32.7 (s, $\underline{C}H_2CHN$), 33.0 (s, $\underline{C}H_2CHN$), 50.4 (s, $\underline{C}HNH$), 50.6 (d, *J* = 7.3 Hz, $\underline{C}H_3O$), 50.8 (s, $\underline{C}HNH$), 50.8 (d, *J* = 6.9 Hz, $\underline{C}H_3O$), 65.3 (s, $\underline{C}H_2OC{=}O$), 65.4 (s, $\underline{C}H_2OC{=}O$), 176.43 (d, *J* = 7.7 Hz, $\underline{C}{=}O$) ppm; **^{31}P NMR** (161.9 MHz, $CDCl_3$): δ =35.69 ($\underline{P}$), 36.44 ($\underline{P}$) ppm; **IR** (ATR, cm^{-1}): ν = 3150 (N-H), 2918 (C-H), 2851 (C-H), 1788 (C=O), 1773 (C=O), 1466, 1377, 1165 (ν_{max}), 1034, 951, 795; **MS** (ES^+): *m/z* (%): 334 ($M+H^+$, 100), 667 ($2M+H^+$, 11), 689 ($2M+Na^+$, 21); **EI-MS** *m/z* (rel. int.): 333 [M] (22), 281 (40), 260 (24), 251 (15), 207 (100), 193 (13), 162 (11), 134 (10), 109 (11), 79 (13), 56 (26), 44 (40); **HRMS** (ESI^+): m/z calculated: $C_{16}H_{33}NO_4P^+$ [$M+H^+$] 334.2142, found: 334.2153.

Synthesis of phenyl *P*-alkyl-*N*-((S)-2-oxotetrahydrofuran-3-yl) phosphonamidates 9a – 9b

The procedure for the McKenna dealkylation was adapted from Dash et al.[15] Ethyl phenyl *P*-alkylphosphonate **2a** or **2b** (2 mmol, 1 equiv.) was dissolved in anhydrous acetonitrile (25 mL) and the reaction mixture was cooled to 0 °C. Trimethylsilyl bromide (16 mmol, 8 equiv.) was added portionwise and the resulting mixture was allowed to come to room temperature overnight. The solvent was evaporated and residue re-evaporated with acetonitrile (25 mL) and subsequently dissolved in dioxane (25 mL). After addition of water (8 mmol, 4 equiv.), the solution was stirred for 30 min at room temperature followed by evaporation of the solvents. Afterwards, the residue was redissolved in 30 mL diethyl ether, extracted with 30 mL 3N HCl, dried and evaporated under reduced pressure to yield product **13**. This product is used in the coupling step without further purification.

Under nitrogen atmosphere, oxalyl chloride (1.32 equiv., 2.64 mmol) is added dropwise to a solution of the corresponding hydrogen phenyl *P*-alkylphosphonate **13** (2 mmol, 1 equiv.) and 3 drops of DMF in 15 mL dry DCM. This solution is stirred for 3 hours at room temperature and the conversion is monitored via ^{31}P NMR. After completion of the reaction, solvent is evaporated under reduced pressure to yield a crude mixture of phosphonochloridate. Due to the instability of the compound, the crude compound is immediately used in subsequent reactions.

The corresponding phosphonochloridate and triethylamine (1.1 equiv., 2.2 mmol) were dissolved in 30 mL dry THF under nitrogen atmosphere and left stirring for 30 min at room temperature, after which the reaction mixture was analysed using ^{31}P NMR. Triethylamine (1.6 equiv., 3.2 mmol) and L-homoserine lactone hydrobromide (1.5 equiv., 3 mmol) was dissolved in 15 mL dry ACN under nitrogen atmosphere, while stirring at room temperature. The solution containing the phosphonochloridate was added dropwise to the amine solution at room temperature and stirred for 1 hour (monitored with ^{31}P NMR). After completion of the reaction the organic layer was diluted with 60 mL diethyl ether, washed with 50 mL water, 50 mL bicarbonate and 50 mL brine and dried with $MgSO_4$. The solvent was evaporated under reduced pressure and the remaining mixture was purified via normal phase automatic flash chromatography.

Phenyl *P*-pentyl-*N*-((*S*)-2-oxotetrahydrofuran-3-yl)phosphonamidate **9a**

Ethyl phenyl *P*-pentylphosphonate **2a** was used as starting product. The crude product was purified using normal phase automated column chromatography (SiO_2, EtOAc:hexane 1:1 v:v, 10 CV gradient to EtOAc:hexane 3:1 v:v) after which, 150 mg of product **9a** was obtained as an orange oil with a yield of 24%.

Spectral data derived from the mixture of diastereomers (dr = 53/47).

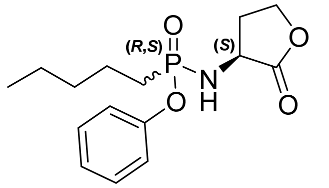

^{1}H NMR (400 MHz, $CDCl_3$): δ = 0.91 (td, *J* = 7.1, 1.9 Hz, 3H, $C\underline{H}_3CH_2CH_2$), 1.28 – 1.48 (m, 4H, $CH_3(C\underline{H}_2)_2$), 1.62 – 2.19 (m, 5H, $C\underline{H}_2P$, $C\underline{H}_2CH_2P$ and $C\underline{H}_aH_bCHN$), 2.45 (ddd, *J* = 12.7, 8.1, 5.1 Hz, 0.5H, $CH_a\underline{H}_bCHN$), 2.45 (ddd, *J* = 12.8, 8.2, 5.0 Hz, 0.5H, $CH_a\underline{H}_bCHN$), 3.10 (dd, *J* = 11.8, 8.0 Hz, 0.5H, $CHN\underline{H}$), 3.37 (dd, *J* = 9.5, 6.9 Hz, 0.5H, $CHN\underline{H}$), 4.02 – 4.39 (m, 3H, $C\underline{H}NH$ and $C\underline{H}_2OC{=}O$), 7.13 – 7.36 (m, 5H, 5 x $C\underline{H}_{arom}$) ppm; **^{13}C NMR** (100.6 MHz, $CDCl_3$): δ = 14.0 (s, $\underline{C}H_3CH_2CH_2$), 22.0 (d, *J* =5.0 Hz, $\underline{C}H_2CH_2P$), 22.2 (d, *J* = 5.1 Hz, $\underline{C}H_2CH_2P$), 22.3 (s, $CH_3\underline{C}H_2CH_2$), 28.4 (d, *J* = 130.6 Hz, $\underline{C}H_2P$), 28.9 (d, *J* = 129.8 Hz, $\underline{C}H_2P$), 32.6 (s, $\underline{C}H_2CHN$), 32.8 (s, $\underline{C}H_2CHN$), 32.9 (d, *J* = 16.9 Hz, $CH_3CH_2\underline{C}H_2$), 32.9 (d, *J* = 16.9 Hz, $CH_3CH_2\underline{C}H_2$), 50.6 (s, $\underline{C}HNH$), 50.7 (s, $\underline{C}HNH$), 65.4 (s, $\underline{C}H_2OC{=}O$), 65.6 (s, $\underline{C}H_2OC{=}O$), 120.7 (d, *J* = 4.4 Hz, 2 x $\underline{C}H_{arom,ortho}$), 121.0 (d, *J* = 4.4 Hz, 2 x $\underline{C}H_{arom,ortho}$), 125.0 (s, $\underline{C}H_{arom,para}$), 125.0 (s, $\underline{C}H_{arom,para}$), 129.8 (s, 2 x $\underline{C}H_{arom,meta}$), 129.9 (s, 2 x $\underline{C}H_{arom,meta}$), 150.4 (d, *J* = 9.4 Hz, $\underline{C}_{arom,quat}$), 150.6 (d, *J* = 8.8 Hz, $\underline{C}_{arom,quat}$), 176.2 (d, *J* = 8.8 Hz, $\underline{C}{=}O$), 176.5 (d, *J* = 9.5 Hz, $\underline{C}{=}O$) ppm; **^{31}P NMR** (161.9 MHz, $CDCl_3$): δ = 33.22 ($\underline{P}$), 33.38 ($\underline{P}$) ppm; **IR** (ATR, cm^{-1}): ν = 3177 (N-H), 2955 (C-H), 2930 (C-H), 2872 (C-H), 1778 (C=O), 1591, 1489, 1375, 1201, 1150 (C-O, ν_{max}), 1003, 914, 897; **MS** (ES^+): *m/z* (%): 312 ($M+H^+$, 100), 645 ($2M+Na^+$, 23); **EI-MS** *m/z* (rel. int.): 311 [M] (10), 281 (5), 238 (5), 218 (100), 190 (32), 162 (10), 135 (7), 94 (71), 77 (12), 66 (16), 56 (19); **HRMS** (ESI^+): m/z calculated: $C_{15}H_{23}NO_4P^+$ [$M+H^+$] 312.1359, found: 312.1360.

Phenyl *P*-undecyl-*N*-((*S*)-2-oxotetrahydrofuran-3-yl)phosphonamidate **9b**

Ethyl phenyl *P*-undecylphosphonate **2b** was used as starting product. The crude product was purified using normal phase automated column chromatography (SiO_2, EtOAc:hexane 1:1 v:v, 10 CV gradient to EtOAc:hexane 3:1 v:v) after which, 260 mg of product **9b** was obtained as a yellow solid with a yield of 33%.

Spectral data derived from the mixture of diastereomers (dr = 54/46).

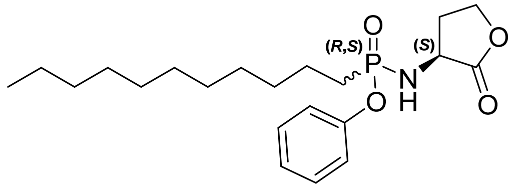

^{1}H NMR (400 MHz, $CDCl_3$): δ = 0.88 (t, *J* = 6.9 Hz, 3H, $C\underline{H}_3CH_2CH_2$), 1.22 - 1.36 (m, 14H, $CH_3(C\underline{H}_2)_7$), 1.37 - 1.45 (m, 2H, $C\underline{H}_2CH_2CH_2P$), 1.64 – 2.19 (m, 5H, $C\underline{H}_2P$, $C\underline{H}_2CH_2P$ and $C\underline{H}_aH_bCHN$), 2.46 (ddd, *J* = 12.7, 8.2, 5.0 Hz, 0.5H, $CH_a\underline{H}_bCHN$), 2.61 (ddd, *J* = 12.5, 8.2, 5.4 Hz, 0.5H, $CH_a\underline{H}_bCHN$), 3.08 (dd, *J* = 12.0, 8.0 Hz, 0.5H, $CHN\underline{H}$), 3.34 (dd, *J* = 9.2, 7.0 Hz, 0.5H, $CHN\underline{H}$), 4.00 – 4.41 (m, 3H, $C\underline{H}NH$ and $C\underline{H}_2OC{=}O$), 7.13 – 7.35 (m, 5H, 5 x $C\underline{H}_{arom}$) ppm; **^{13}C NMR** (100.6 MHz, $CDCl_3$): δ = 14.3 (s, $\underline{C}H_3CH_2CH_2$), 22.4 (d, *J* = 5.1 Hz, $\underline{C}H_2CH_2P$), 22.5 (d, *J* = 5.1 Hz, $\underline{C}H_2CH_2P$), 22.8 (s, $CH_3\underline{C}H_2CH_2$), 28.5 (d, *J* = 130.6 Hz, $\underline{C}H_2P$), 28.9 (d, *J* = 128.9 Hz, $\underline{C}H_2P$), 29.3, 29.5, 29.5, 29.7 (s, $CH_3CH_2CH_2(\underline{C}H_2)_5$), 30.8 (d, *J* = 17.6

Hz, $CH_2(CH_2)_2P$), 30.8 (d, *J* = 17.6 Hz, $CH_2(CH_2)_2P$), 32.0 (s, $CH_3CH_2CH_2$), 32.6 (s, CH_2CHN), 32.8 (s, CH_2CHN), 50.6 (s, CHNH), 50.7 (s, CHNH), 65.4 (s, CH_2OC=O), 65.6 (s, CH_2OC=O), 120.7 (d, *J* = 5.1 Hz, 2 x $CH_{arom,ortho}$), 121.03 (d, *J* = 4.6 Hz, 2 x $CH_{arom,ortho}$), 125.0 (s, $CH_{arom,para}$), 125.0 (s, $CH_{arom,para}$), 129.8 (s, 2 x $CH_{arom,meta}$), 129.9 (s, 2 x $CH_{arom,meta}$), 150.4 (d, *J* = 9.5 Hz, $C_{arom,quat}$), 150.6 (d, *J* = 9.3 Hz, $C_{arom,quat}$), 176.2 (d, *J* = 8.8 Hz, C=O), 176.4 (d, *J* = 9.6 Hz, C=O) ppm; **^{31}P NMR** (161.9 MHz, $CDCl_3$): δ = 33.24 (P), 33.39 (P) ppm; **IR** (ATR, cm^{-1}): ν = 3164 (N-H), 2916 (C-H), 2851 (C-H), 1775 (C=O), 1591, 1489, 1204, 1159, 1001, 891 (ν_{max}), 773; **MS** (ES^+): *m/z* (%): 396 ($M+H^+$, 100), 813 ($2M+Na^+$, 14); **HRMS** (ESI^+): m/z calculated: $C_{21}H_{35}NO_4P^+$ [$M+H^+$] 396.2298, found: 396.2302.

General procedure C: synthesis of phosphoramidates 15a – 15d

The corresponding dialkyl chlorophosphate **14** (3.7 mmol) and triethylamine (1.1 equiv.; 567 µL; 4.0 mmol) were dissolved in 25 mL dry THF under nitrogen atmosphere and left stirring for 30 min at room temperature, after which the reaction mixture was analysed using ^{31}P NMR. The amino acid ester salt (1.5 equiv.; 5.5 mmol) and triethylamine (1.6 equiv.; 825 µL; 5.9 mmol) were dissolved in 25 mL dry ACN under nitrogen atmosphere, while stirring at room temperature. The solution containing the phosphorochloridate was added dropwise to the amino acid solution at room temperature and stirred for 1 hour. Subsequently, the excess salts were removed via filtration, upon addition of 60 mL diethyl ether and the solvent was evaporated under reduced pressure. The remaining mixture was purified via normal phase automatic flash chromatography.

Methyl *N*-(diethoxyphosphoryl)-glycinate **15a**

Glycine methyl ester hydrochloride and diethyl chlorophosphate **14a** were used as starting product for general procedure C. The crude product was purified using normal phase automatic flash chromatography (SiO_2, 40% (EtOAc:iPA) 3:1 v:v in hexane), after which 760 mg of product **15a** was obtained as a pale yellow oil in a yield of 95%.

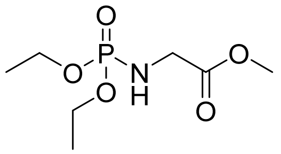

^{1}H NMR (400 MHz, $CDCl_3$): δ = 1.31 (td, *J* = 7.1, 0.9 Hz, 6H, 2 x CH_3CH_2O), 3.21 – 3.38 (m, 1H, $NHCH_2$), 3.64 – 3.82 (m, 5H, $COOCH_3$ and $NHCH_2$), 3.99 – 4.19 (m, 4H, 2 x CH_3CH_2O) ppm; **^{13}C NMR** (100.6 MHz, $CDCl_3$): δ = 16.1 (d, *J* = 7.0 Hz, 2 x CH_3CH_2O), 42.8 (d, *J* = 1.4 Hz, $NHCH_2$), 52.2 (s, OCH_3), 62.5 (d, *J* = 5.2 Hz, 2 x CH_3CH_2O), 171.4 (d, *J* = 7.5 Hz, COO) ppm; **^{31}P NMR** (161.9 MHz, $CDCl_3$): δ = 7.63 (P) ppm; **IR** (ATR, cm^{-1}): ν = 3240 (N-H), 2983 (C-H), 1753 (C=O), 1436, 1205, 1151, 1020 (ν_{max}), 960, 869, 794, 731; **MS** (ES^+): *m/z* (%): 226 ($M+H^+$, 100), 451 ($2M+H^+$, 21), 473 ($2M+Na^+$, 23); **EI-MS** *m/z* (rel. int.): 225 [M] (2), 166 (100), 152 (4), 138 (27), 122 (8), 110 (54), 94 (6), 81 (4); **HRMS** (ESI^+): m/z calculated: $C_7H_{16}NO_5P^+$ [$M+H^+$] 226.0839, found: 226.0841.

Diethyl (S)-(2-oxotetrahydrofuran-3-yl)phosphoramidate **15b**

L-homoserine lactone hydrobromide and diethyl chlorophosphate **14a** were used as starting product for general procedure C. The crude product was purified using normal phase automatic flash chromatography (SiO_2, 40% (EtOAc:iPA) 3:1 v:v in hexane), after which 860 mg of product **15b** was obtained as a yellow oil in a yield of 89%.

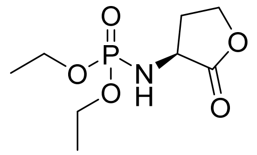

^{1}H NMR (400 MHz, $CDCl_3$): δ = 1.34 (2 x td, *J* = 7.1, 0.8 Hz, 6H, 2 x C<u>H</u>$_3$CH$_2$O), 2.13 – 2.26 (m, 1H, NHCHC<u>H</u>$_a$H$_b$), 2.65 – 2.73 (m, 1H, NHCHCH$_a$<u>H</u>$_b$), 3.44 (t, *J* = 8.2, 1H, N<u>H</u>CH), 3.98 – 4.25 (m, 6H, 2 x CH$_3$C<u>H</u>$_2$O, NHC<u>H</u> and C<u>H</u>$_a$H$_b$OCO), 4.41 (t, *J* = 8.9 Hz, 1H, CH$_a$<u>H</u>$_b$OCO) ppm; **^{13}C NMR** (100.6 MHz, $CDCl_3$): δ = 16.2 (d, *J* = 4.4 Hz, <u>C</u>H$_3$CH$_2$O), 16.3 (d, *J* = 5.1 Hz, <u>C</u>H$_3$CH$_2$O), 32.1 (d, *J* = 1.5 Hz, NHCH<u>C</u>H$_2$), 50.8 (s, NH<u>C</u>H), 62.9, 63.0 (2 x d, *J* = 5.2 Hz, 2 x CH$_3$<u>C</u>H$_2$O), 65.4 (s, <u>C</u>H$_2$OCO), 176.0 (d, *J* = 11.0 Hz, <u>C</u>OO) ppm; **^{31}P NMR** (161.9 MHz, $CDCl_3$): δ = 7.20 (<u>P</u>) ppm; **IR** (ATR, cm^{-1}): ν = 3196 (N-H), 2982 (C-H), 2911 (C-H), 1778 (C=O), 1452, 1229, 1155, 1055, 1022 ($ν_{max}$), 972, 732; **MS** (ES$^+$): *m/z* (%): 238 (M+H$^+$, 100), 475 (2M+H$^+$, 5), 497 (2M+Na$^+$, 25); **EI-MS** *m/z* (rel. int.): 237 [M] (17), 193 (98), 179 (26), 164 (100), 152 (7), 136 (98), 124 (11), 109 (13), 81 (22), 56 (78); **HRMS** (ESI$^+$): m/z calculated: $C_8H_{16}NO_5P^+$ [M+H$^+$] 238.0839, found: 238.0841.

Methyl *N*-(diethoxyphosphoryl)-L-prolinate **15c**

L-proline methyl ester hydrochloride and diethyl chlorophosphate **14a** were used as starting product for general procedure C. The crude product was purified using normal phase automatic flash chromatography (SiO_2, 40% (EtOAc:iPA) 3:1 v:v in hexane), after which 865 mg of product **15c** was obtained as an orange oil in a yield of 88%.

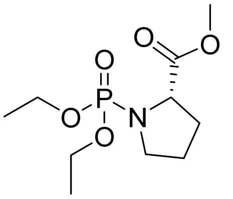

^{1}H NMR (400 MHz, $CDCl_3$): δ = 1.22 – 1.39 (m, 6H, 2 x C<u>H</u>$_3$CH$_2$O), 1.83 – 2.07 (m, 3H, CHC<u>H</u>$_a$H$_b$ and CHCH$_2$C<u>H</u>$_2$), 2.11 – 2.24 (m, 1H, CHCH$_a$<u>H</u>$_b$), 3.22 – 3.36 (m, 2H, NC<u>H</u>$_2$), 3.72 (s, 3H, COOC<u>H</u>$_3$), 3.93 – 4.22 (m, 4H, 2 x CH$_3$C<u>H</u>$_2$O), 4.23 – 4.31 (m, 1H, C<u>H</u>N) ppm; **^{13}C NMR** (100.6 MHz, $CDCl_3$): δ = 16.1, 16.2 (2 x d, *J* = 7.1 Hz, 2 x <u>C</u>H$_3$CH$_2$O), 25.2 (d, *J* = 8.4 Hz, CHCH$_2$<u>C</u>H$_2$), 31.3 (d, *J* = 8.4 Hz, CH<u>C</u>H$_2$), 47.0 (d, *J* = 4.7 Hz, N<u>C</u>H$_2$), 52.0 (s, O<u>C</u>H$_3$), 60.0 (d, *J* = 6.6 Hz, N<u>C</u>H), 62.2, 62.3 (2 x d, *J* = 5.2 Hz, 2 x CH$_3$<u>C</u>H$_2$O), 174.5 (s, <u>C</u>OO) ppm; **^{31}P NMR** (161.9 MHz, $CDCl_3$): δ = 6.03 (<u>P</u>) ppm; **IR** (ATR, cm^{-1}): ν = 2981 (C-H), 1747 (C=O), 1444, 1253, 1209, 1151 (C-O), 1020 ($ν_{max}$), 962, 794, 727; **MS** (ES$^+$): *m/z* (%): 266 (M+H$^+$, 100), 553 (2M+Na$^+$, 25); **EI-MS** *m/z* (rel. int.): 265 [M] (2), 206 (100), 178 (33), 150 (85), 132 (11), 70 (7); **HRMS** (ESI$^+$): m/z calculated: $C_{10}H_{20}NO_5P^+$ [M+H$^+$] 266.1152, found: 266.1154.

Methyl *N*-(diphenoxyphosphoryl)-glycinate **15d**

Glycine methyl ester hydrochloride and diphenyl chlorophosphate **14b** were used as starting product for general procedure C. The crude product was dissolved in 10 mL methanol, diluted with water and filtered off to yield the desired product, after which 1068 mg of product **15d** was obtained as a white powder in a yield of 91%.

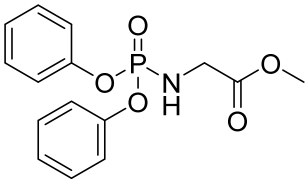

Melting point: 87 °C; **^{1}H NMR** (400 MHz, $CDCl_3$): δ = 3.67 – 3.76 (m, 4H, N$\underline{H}$CH$_2$ and COOC$\underline{H}_3$), 3.87 (dd, *J* = 9.3, 6.0 Hz, 2H, NHC$\underline{H}_2$), 7.17 (t, *J* = 7.2 Hz, 2H, 2 x C$\underline{H}_{arom,para}$), 7.22 - 7.26 (m, 4H, 4 x C$\underline{H}_{arom,ortho}$), 7.30 -7.36 (m, 4H, 4 x C$\underline{H}_{arom,meta}$) ppm; **^{13}C NMR** (100.6 MHz, $CDCl_3$): δ = 43.2 (s, NH$\underline{C}$H$_2$), 52.6 (s, O$\underline{C}$H$_3$), 120.3 (d, *J* = 5.2 Hz, 4 x $\underline{C}$H$_{arom,ortho}$), 125.3 (s, 2 x $\underline{C}$H$_{arom,para}$), 129.9 (s, 4 x $\underline{C}$H$_{arom,meta}$), 150.7 (d, *J* = 6.6 Hz, 2 x $\underline{C}$H$_{arom,quat}$), 170.7 (d, *J* = 9.5 Hz, $\underline{C}$OO) ppm; **^{31}P NMR** (161.9 MHz, $CDCl_3$): δ = -2.27 ($\underline{P}$) ppm; **IR** (ATR, cm^{-1}): ν = 3231 (N-H), 1740 (C=O), 1587, 1485, 1240, 1180, 1147, 927 (ν_{max}), 772, 692; **MS** (ES$^+$): *m/z* (%): 322 (M+H$^+$, 100), 643 (2M+H$^+$, 5), 665 (2M+Na$^+$, 25); **EI-MS** *m/z* (rel. int.): 321 [M] (11), 262 (29), 228 (100), 200 (89), 168 (23), 94 (69), 77 (31), 65 (17); **HRMS** (ESI$^+$): m/z calculated: $C_{15}H_{16}NO_5P^+$ [M+H$^+$] 322.0839, found: 322.0839.

References

(1) Desmedt, W.; Ameye, M.; Filipe, O.; De Waele, E.; Van Nieuwerburgh, F.; Deforce, D.; Van Meulebroek, L.; Vanhaecke, L.; Kyndt, T.; Höfte, M.; Audenaert, K. Molecular Analysis of Broad-Spectrum Induced Resistance in Rice by the Green Leaf Volatile Z-3-Hexenyl Acetate. *J Exp Bot* **2023**, *74* (21), 6804–6819.

(2) De Zutter, N.; Ameye, M.; Debode, J.; De Tender, C.; Ommeslag, S.; Verwaeren, J.; Vermeir, P.; Audenaert, K.; De Gelder, L. Shifts in the Rhizobiome during Consecutive in Planta Enrichment for Phosphate-Solubilizing Bacteria Differentially Affect Maize P Status. *Microb Biotechnol* **2021**, *14* (4), 1594–1612.

(3) Baker, N. R. Chlorophyll Fluorescence: A Probe of Photosynthesis In Vivo. *Annu Rev Plant Biol* **2008**, *59*, 89–113.

(4) Wu, C.; Varanasi, V.; Perez-Jones, A. A Nondestructive Leaf-Disk Assay for Rapid Diagnosis of Weed Resistance to Multiple Herbicides. *Weed Sci* **2021**, *69* (3), 274–283.

(5) Backx, S.; Dejaegere, A.; Simoens, A.; Van de Poel, J.; Krasowska, D.; Stevens, C. V.; Mangelinckx, S. Triethylamine-Mediated Transformation of Phosphonates into Phosphonamidates. *European J Org Chem* **2023**, *26* (26), e202300172.

(6) Koehne, I.; Pietschnig, R. Synthesis of Geminal Bis- and Tetrakisphosphonate Ester Derivatives and Their Coordination Behavior Towards Ca(II) Ions. *Eur J Inorg Chem* **2022**, *2022* (17), e202200194.

(7) Nathanael, J. G.; White, J. M.; Richter, A.; Nuske, M. R.; Wille, U. Oxidative Damage of Proline Residues by Nitrate Radicals ($NO3^{\cdot}$): A Kinetic and Product Study. *Org Biomol Chem* **2020**, *18* (35), 6949–6957.

(8) Syrpas, M.; Ruysbergh, E.; Stevens, C. V.; De Kimpe, N.; Mangelinckx, S. Synthesis and Biological Evaluation of Novel N-α-Haloacylated Homoserine Lactones as Quorum Sensing Modulators. *Beilstein Journal of Organic Chemistry* **2014**, *10* (1), 2539–2549.

(9) Syrpas, M.; Ruysbergh, E.; Blommaert, L.; Vanelslander, B.; Sabbe, K.; Vyverman, W.; De Kimpe, N.; Mangelinckx, S. Haloperoxidase Mediated Quorum Quenching by Nitzschia Cf Pellucida: Study of the Metabolization of N-Acyl Homoserine Lactones by a Benthic Diatom. *Mar Drugs* **2014**, *12* (1), 352–367.

(10) Persson, T.; Hansen, T. H.; Rasmussen, T. B.; Skindersø, M. E.; Givskov, M.; Nielsen, J. Rational Design and Synthesis of New Quorum-Sensing Inhibitors Derived from Acylated Homoserine Lactones and Natural Products from Garlic. *Org. Biomol. Chem.* **2005**, *3* (2), 253–262.

(11) Angle, S. R.; Henry, R. M. Studies toward the Synthesis of (+)-Palustrine: The First Asymmetric Synthesis of (−)-Methyl Palustramate. *Journal of Organic Chemistry* **1998**, *63* (21), 7490–7497.

(12) Delorme, V.; Raux, B.; Puppo, R.; Leclaire, J.; Cavalier, J. F.; Marc, S.; Kamarajugadda, P. K.; Buono, G.; Fotiadu, F.; Canaan, S.; Carrière, F. Supported Inhibitor for Fishing Lipases in Complex Biological Media and Mass Spectrometry Identification. *Biochimie* **2014**, *107* (Part A), 124–134.

(13) Antczak, M. I.; Montchamp, J.-L. Mild Synthesis of Organophosphorus Compounds: Reaction of Phosphorus-Containing Carbenoids with Organoboranes. *Org Lett* **2008**, *10* (5), 977–980.

(14) Li, C. K.; Tao, Z. K.; Shoberu, A.; Zhang, W.; Zou, J. P. Copper-Catalyzed Cross-Coupling of Alkyl and Phosphorus Radicals for C(Sp3)-P Bond Formation. *Org Lett* **2022**, *24* (32), 6083–6087.

(15) Dash, R. P.; Tichý, T.; Veeravalli, V.; Lam, J.; Alt, J.; Wu, Y.; Tenora, L.; Majer, P.; Slusher, B. S.; Rais, R. Enhanced Oral Bioavailability of 2-(Phosphonomethyl)-Pentanedioic Acid (2-PMPA) from Its (5-Methyl-2-Oxo-1,3-Dioxol-4-Yl)Methyl (ODOL)-Based Prodrugs. *Mol Pharm* **2019**, *16* (10), 4292–4301.

NMR spectra

Compound **1c**: ^{1}**H NMR** (400 MHz, $CDCl_3$), ^{13}**C NMR** (100.6 MHz, $CDCl_3$) and ^{31}**P NMR** (161.9 MHz, $CDCl_3$).

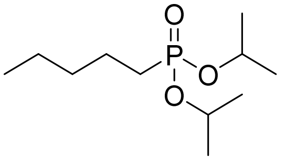

1c

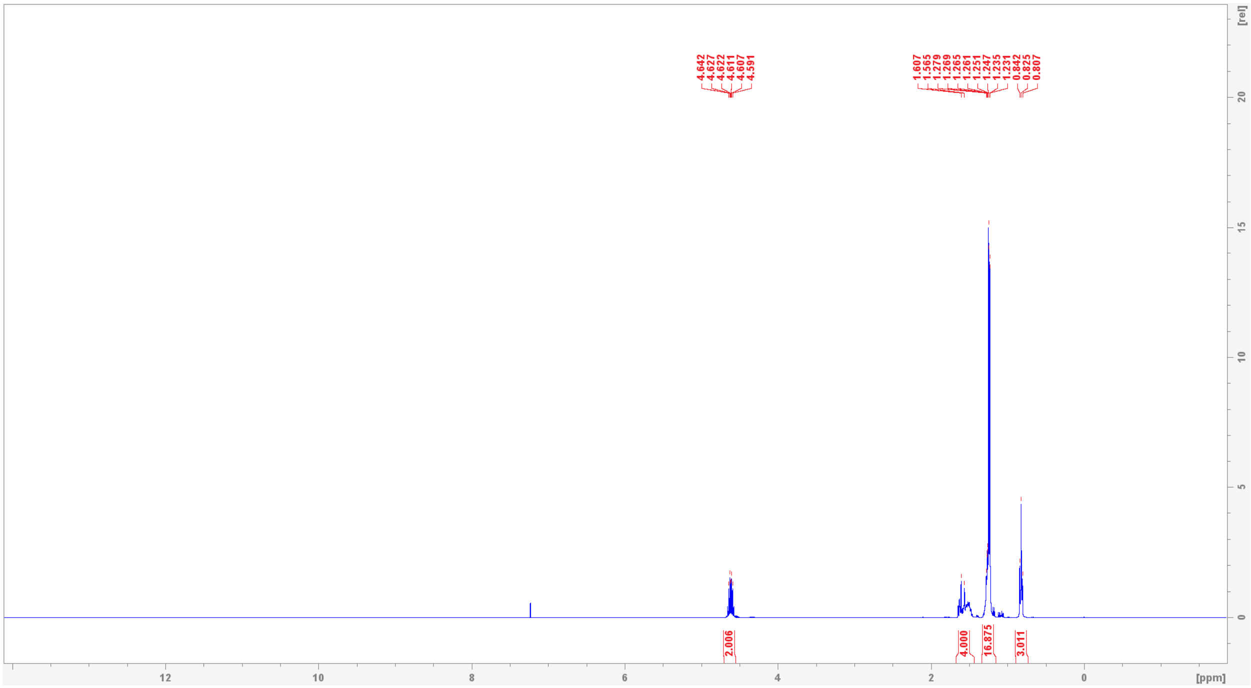

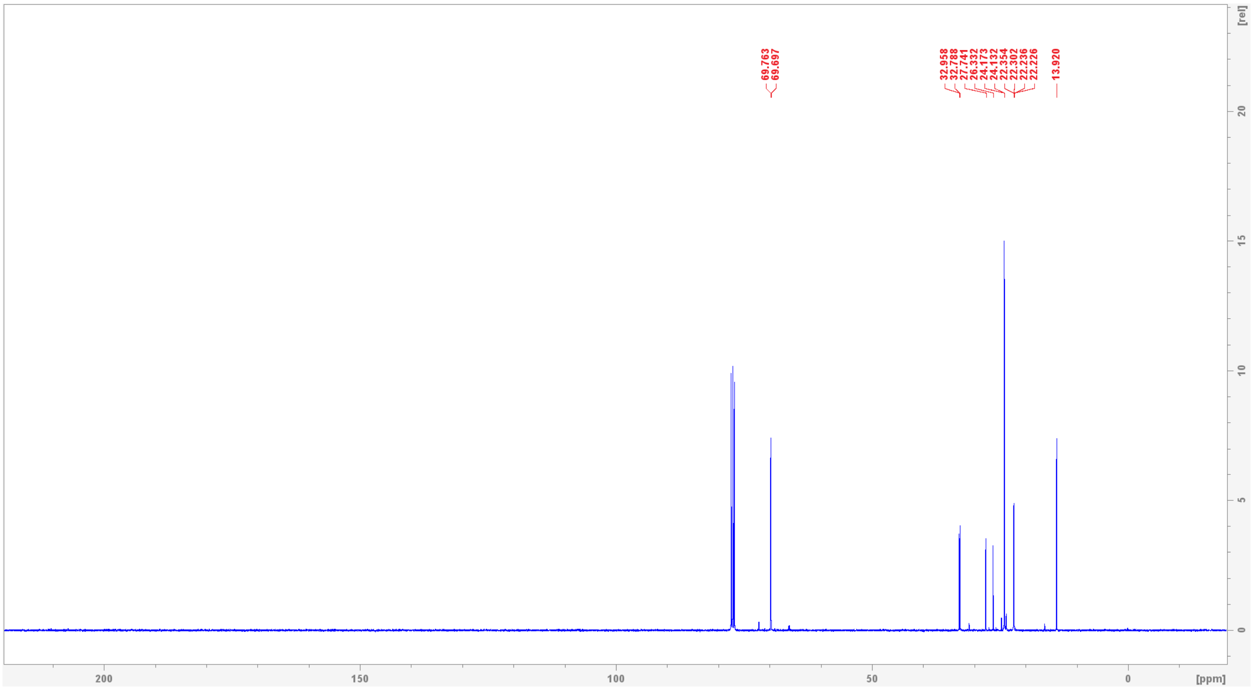
69.763
69.697
32.958
32.788
27.741
26.332
24.173
24.132
22.354
22.302
22.236
22.226
13.920
[rel]
20
15
10
5
0
200
150
100
50
0
[ppm]

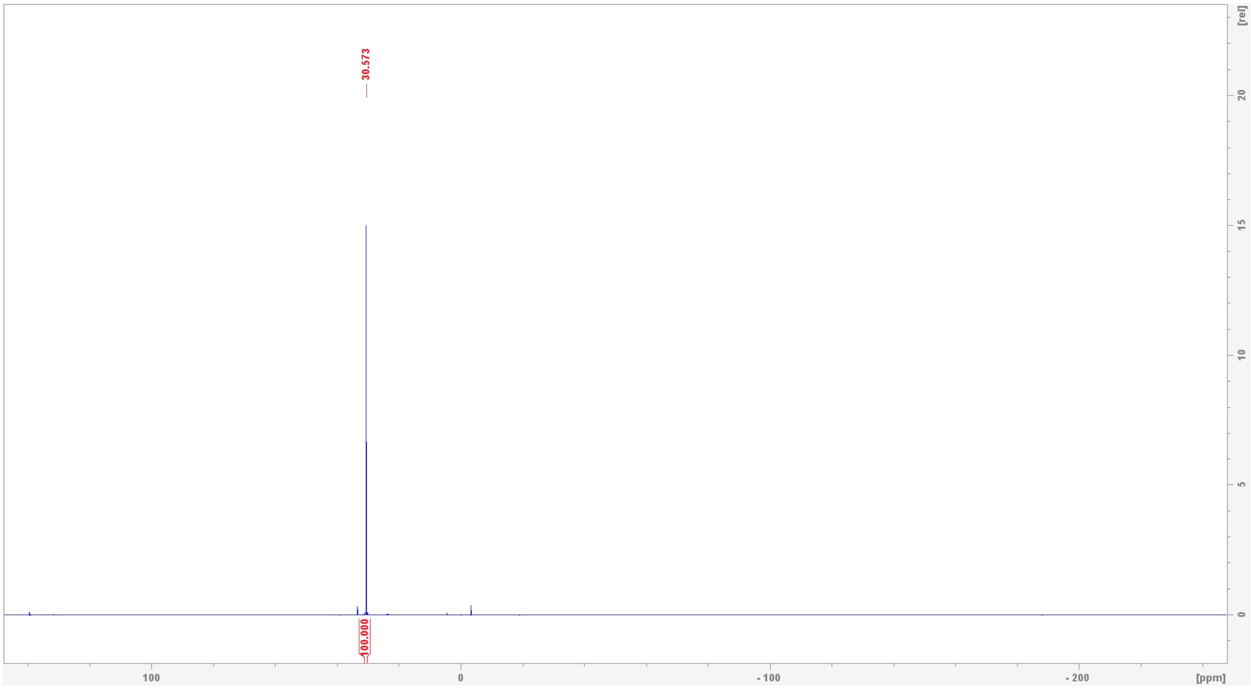
30.573
100.000
[rel]
20
15
10
5
0
100
0
- 100
- 200
[ppm]

Compound **1e**: ^{1}**H NMR** (400 MHz, $CDCl_3$), ^{13}**C NMR** (100.6 MHz, $CDCl_3$) and ^{31}**P NMR** (161.9 MHz, $CDCl_3$).

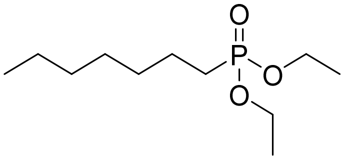

1e

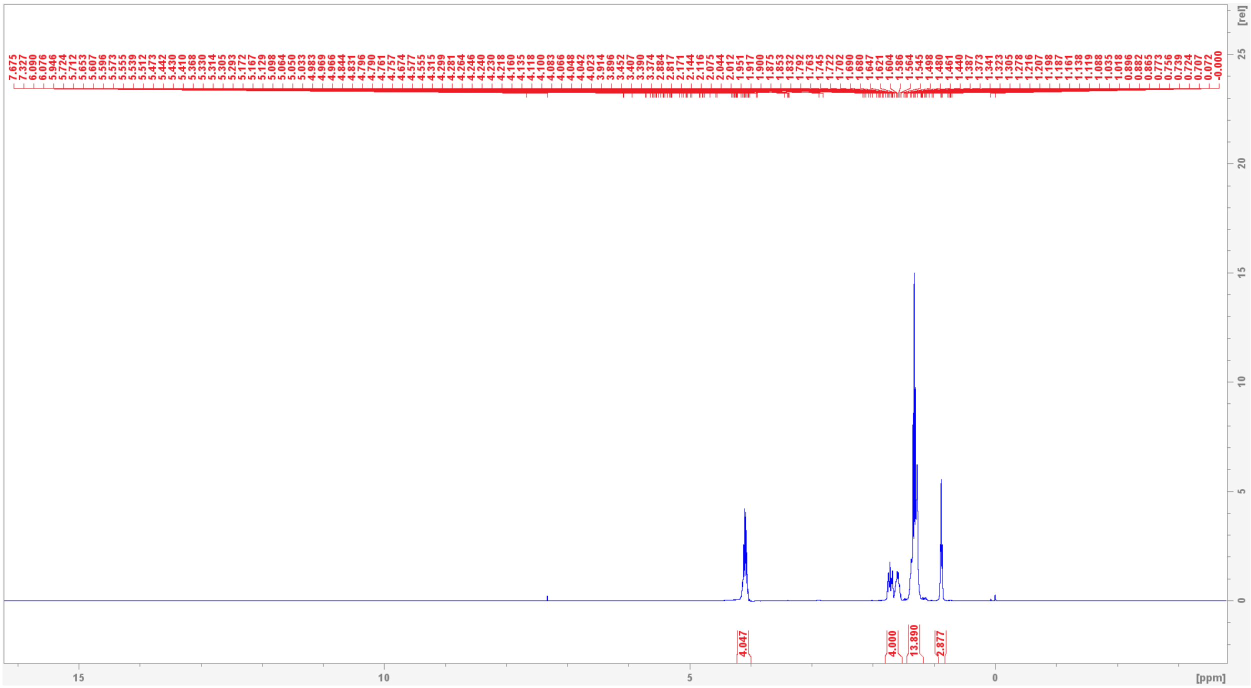

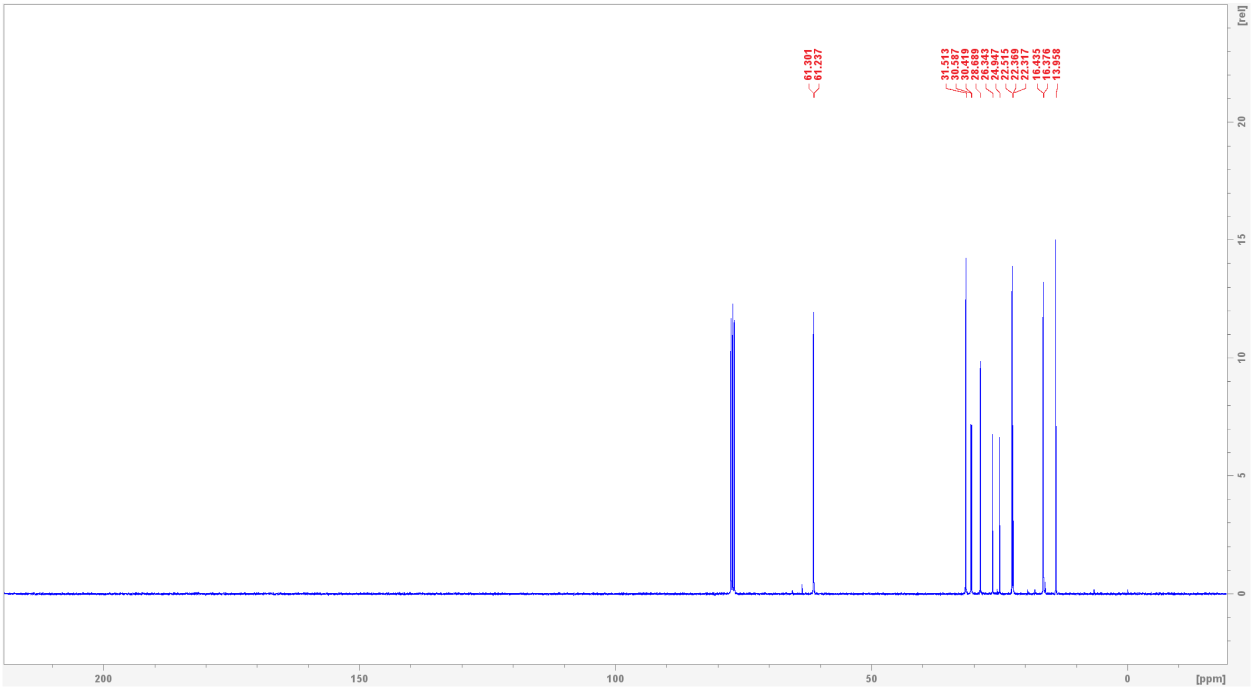

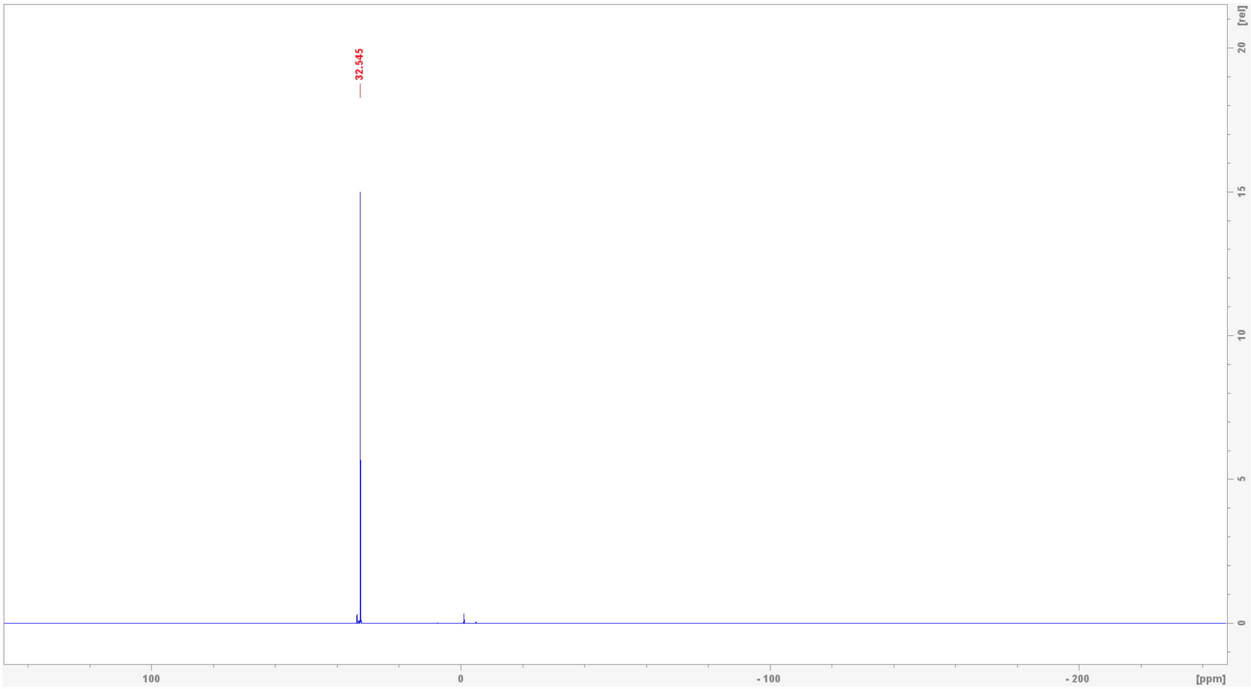

32.545
[rel]
20
15
10
5
0
100
0
- 100
- 200
[ppm]

Compound **1f**: ^{1}**H NMR** (400 MHz, $CDCl_3$), ^{13}**C NMR** (100.6 MHz, $CDCl_3$) and ^{31}**P NMR** (161.9 MHz, $CDCl_3$).

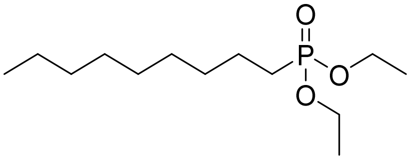

1f

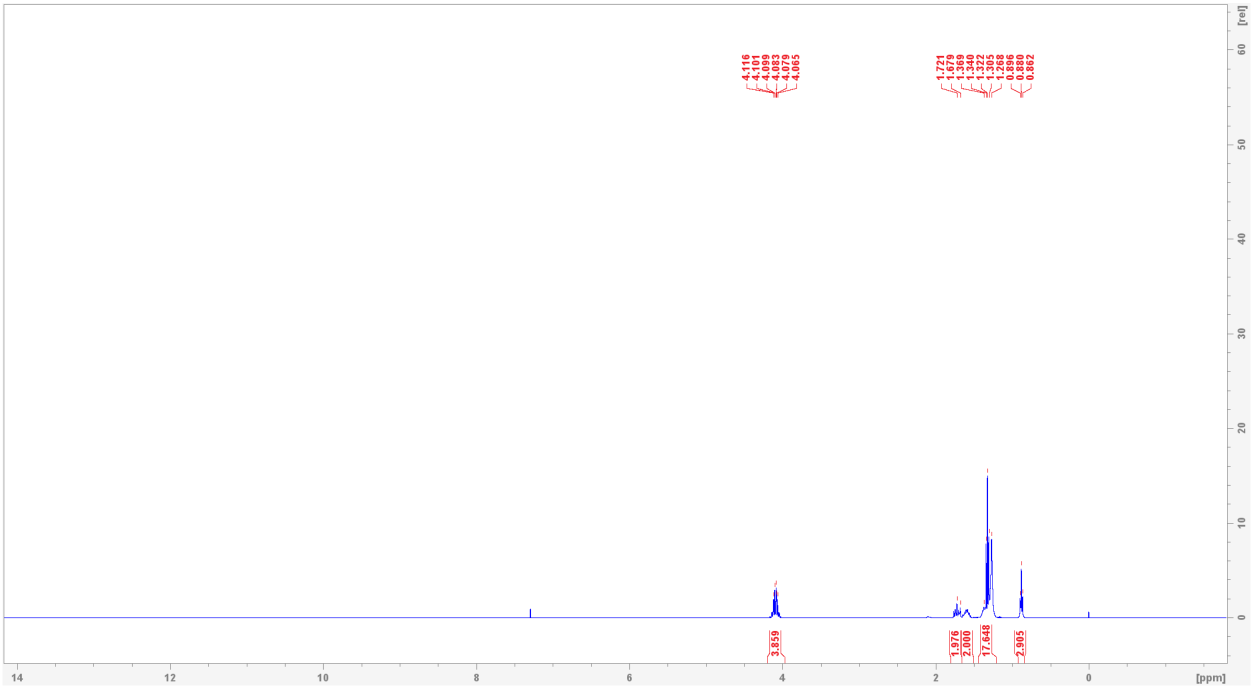

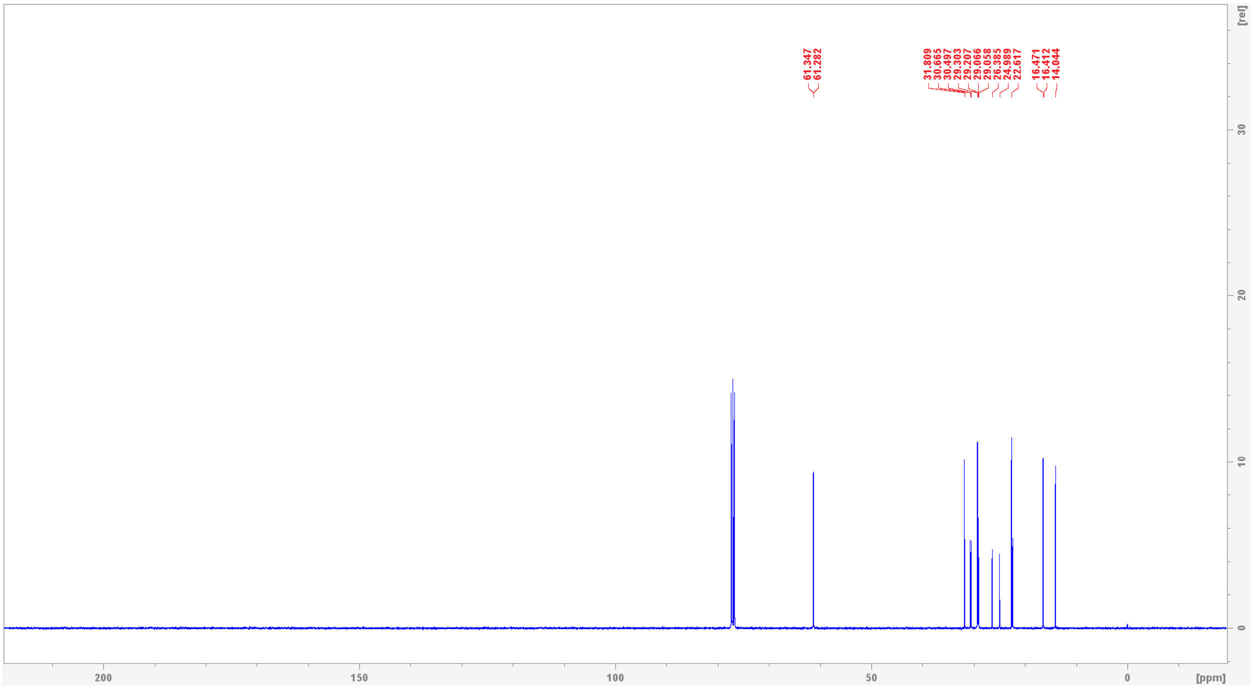

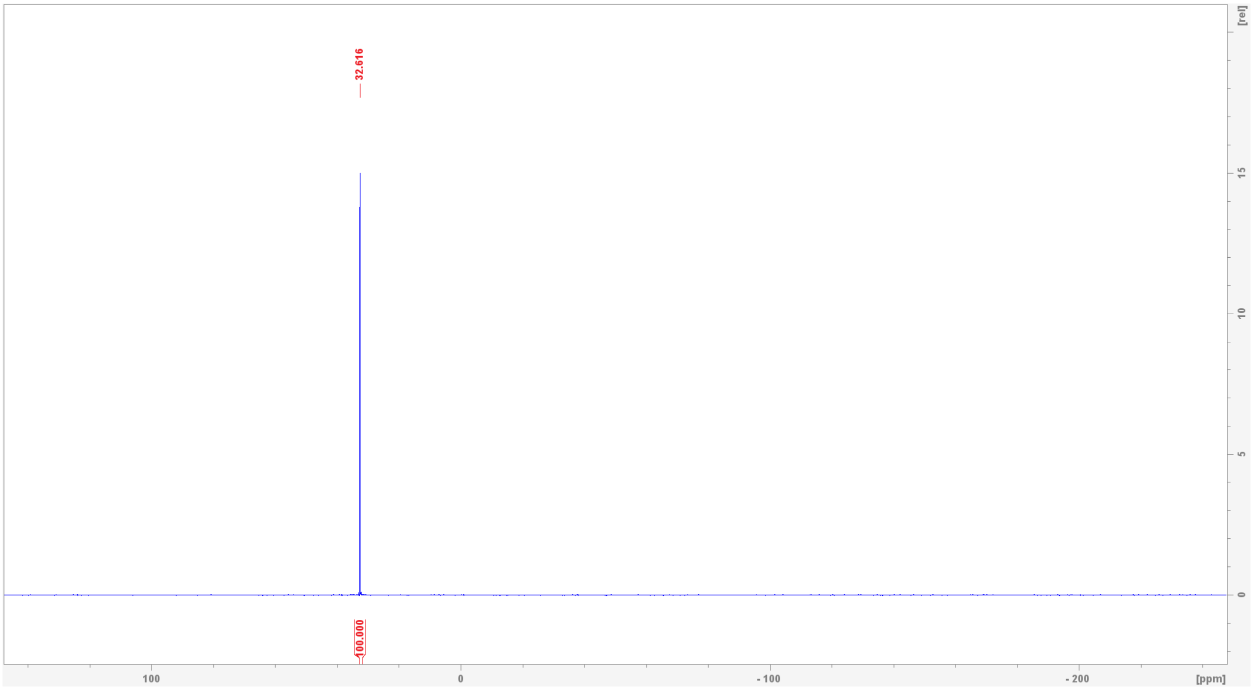
32.616
100.000
100
0
- 100
- 200
[ppm]
[rel]
15
10
5
0

Compound **2a**: **^{1}H NMR** (400 MHz, $CDCl_3$), **^{13}C NMR** (100.6 MHz, $CDCl_3$) and **^{31}P NMR** (161.9 MHz, $CDCl_3$).

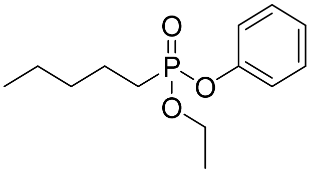

2a

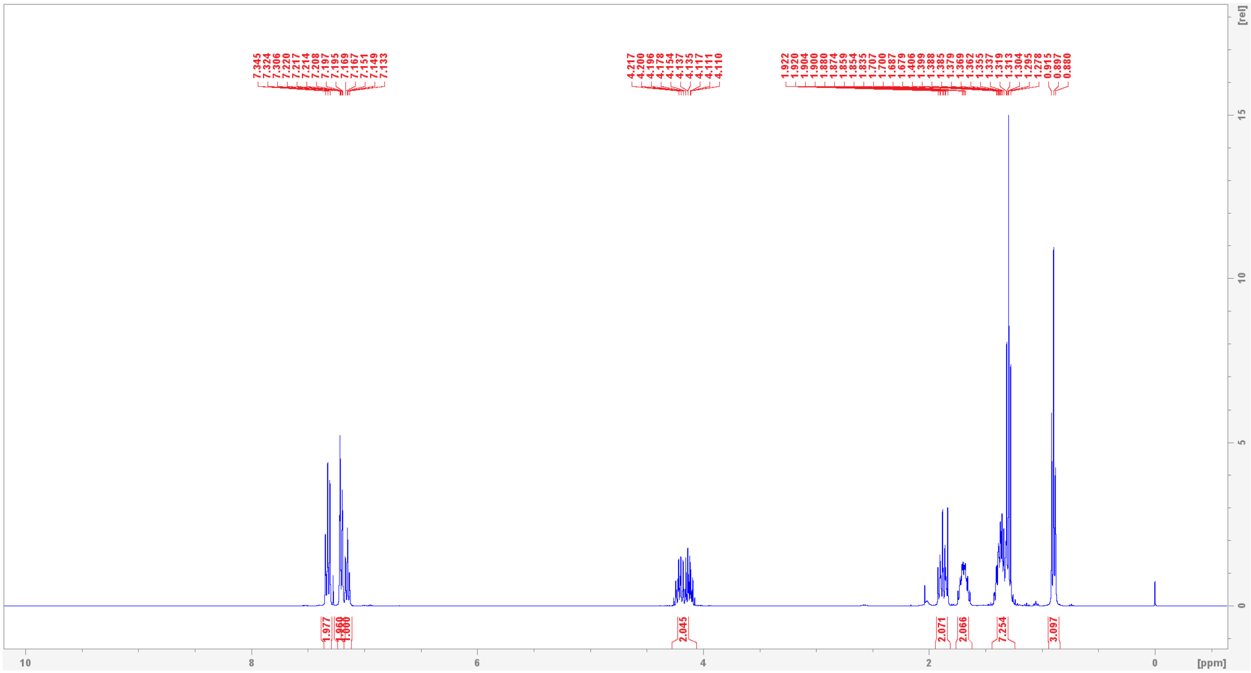

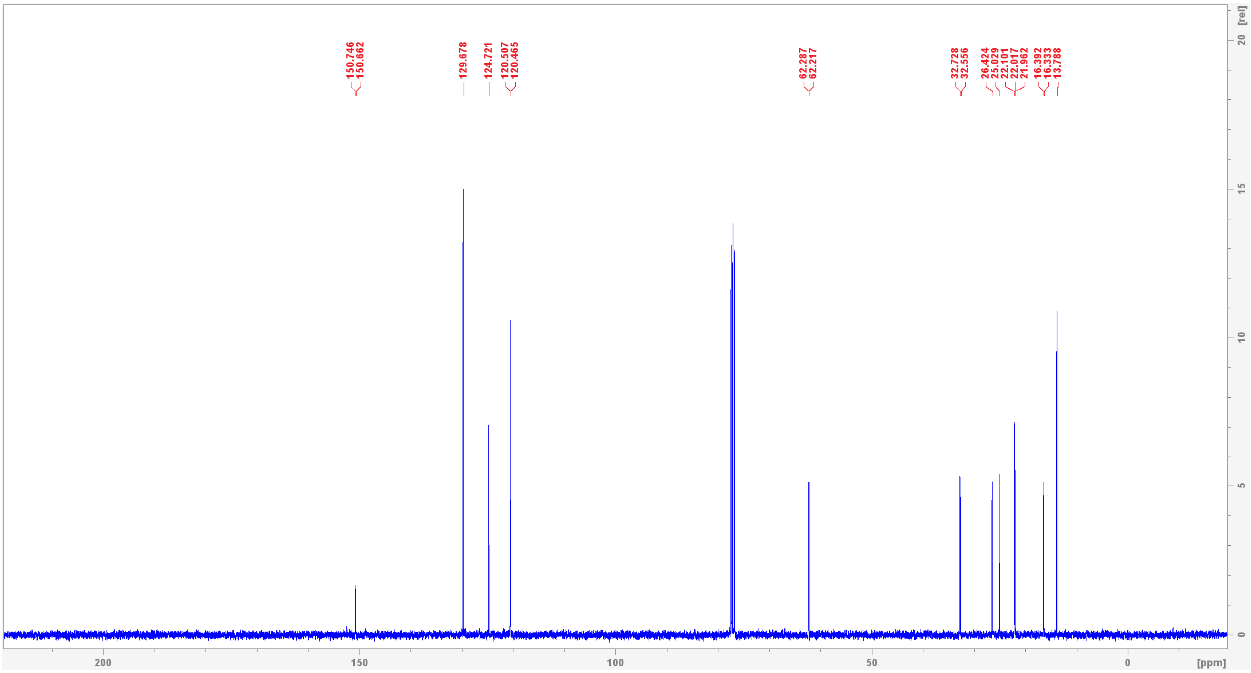

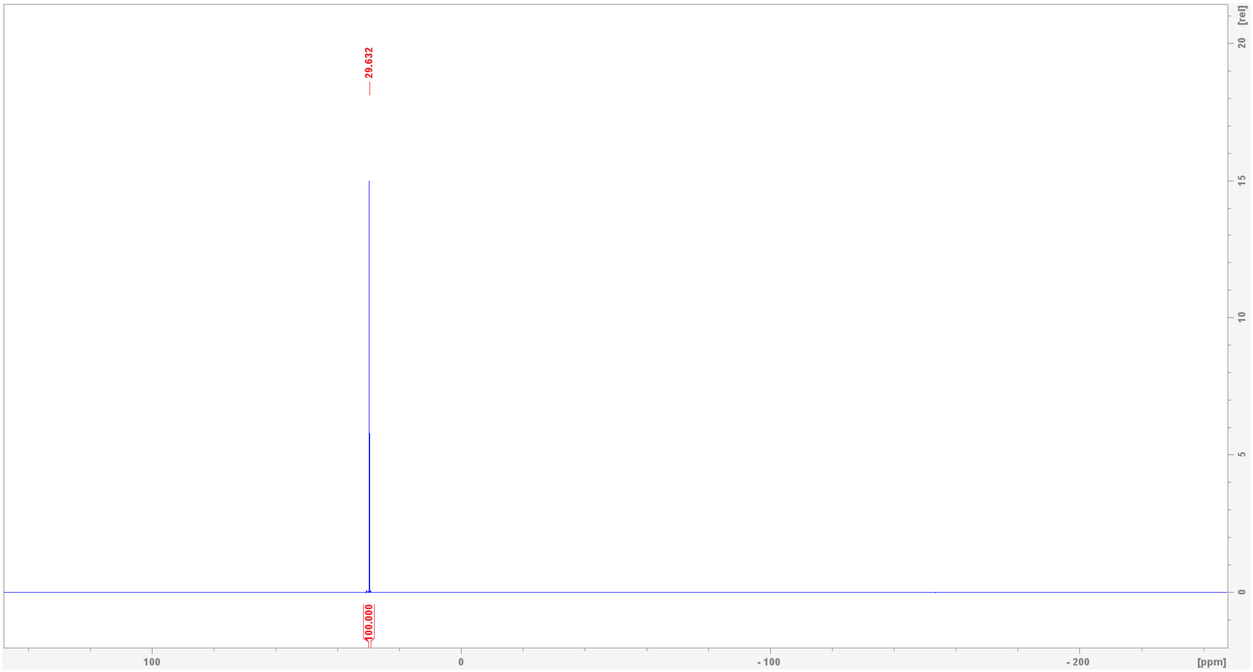
29.632
100.000
100
0
- 100
- 200
[ppm]
[rel]
20
15
10
5
0

Compound **2b**: ^{1}**H NMR** (400 MHz, $CDCl_3$), ^{13}**C NMR** (100.6 MHz, $CDCl_3$) and ^{31}**P NMR** (161.9 MHz, $CDCl_3$).

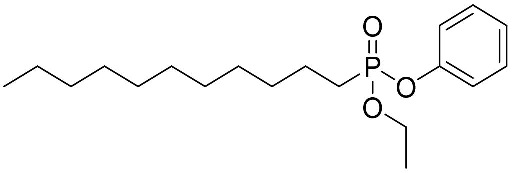

2b

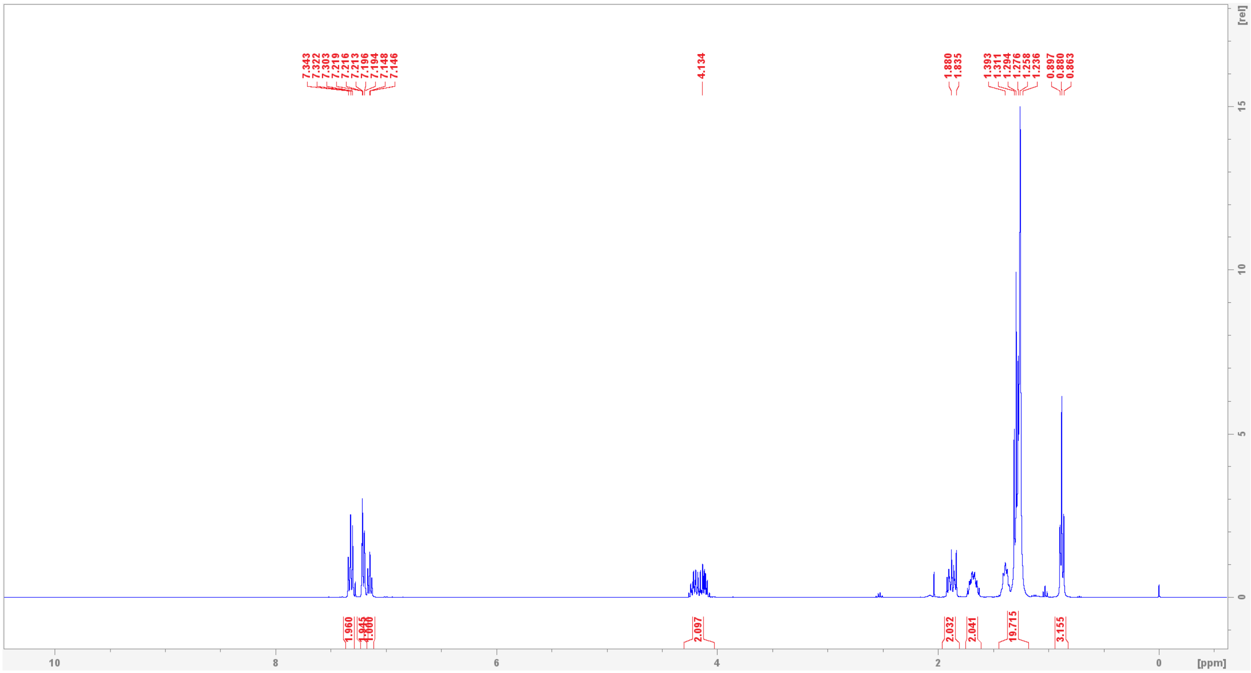

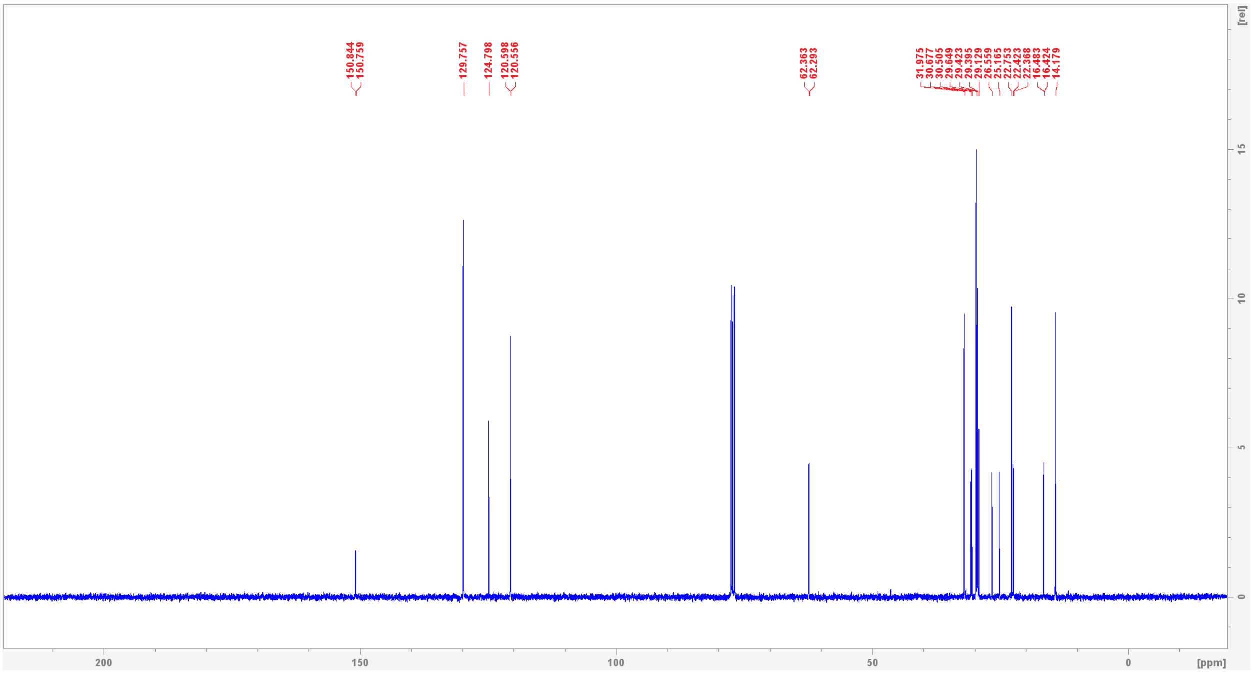

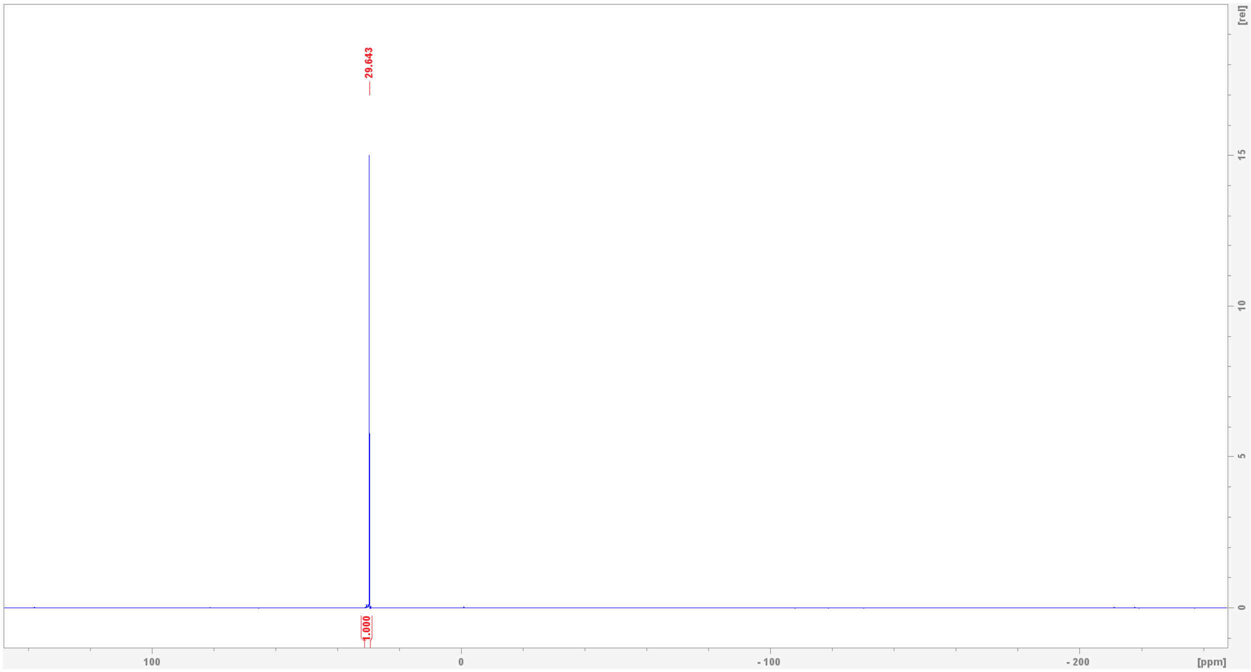

29.643
1.000
[rel]
15
10
5
0
100
0
- 100
- 200
[ppm]

Compound **2c**: **^{1}H NMR** (400 MHz, $CDCl_3$), **^{13}C NMR** (100.6 MHz, $CDCl_3$) and **^{31}P NMR** (161.9 MHz, $CDCl_3$).

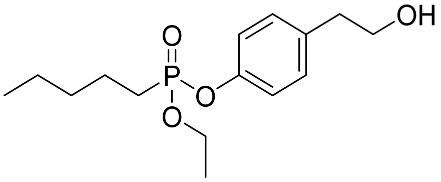

2c

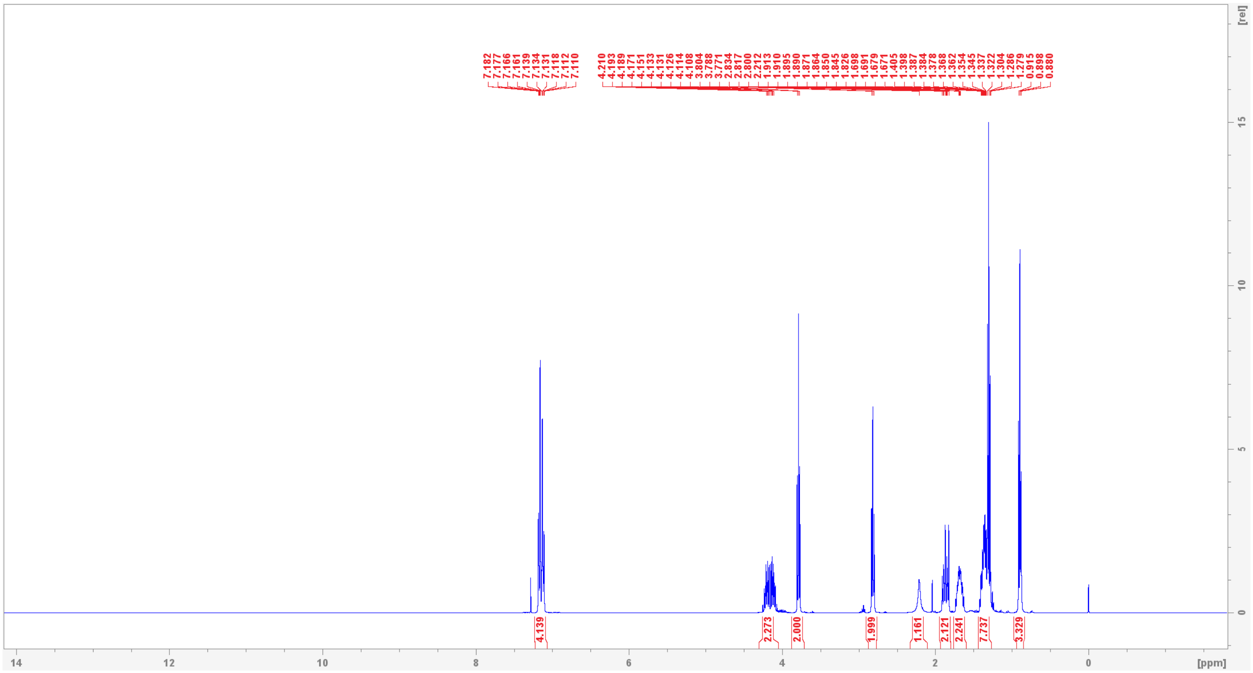

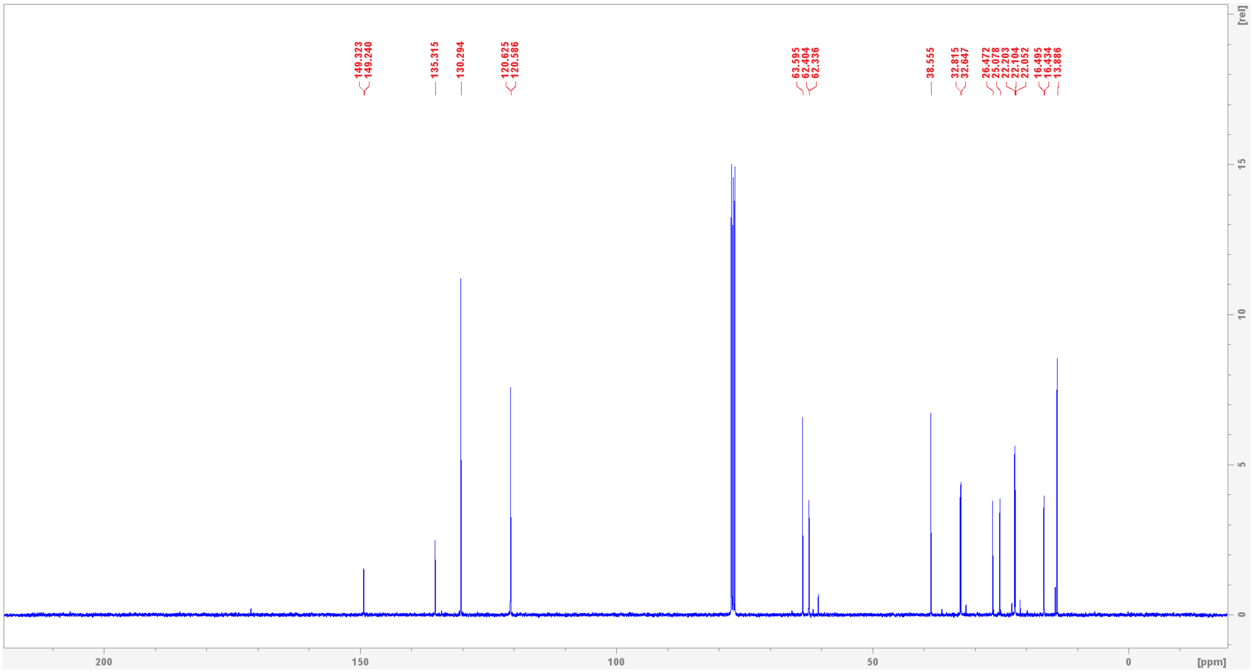

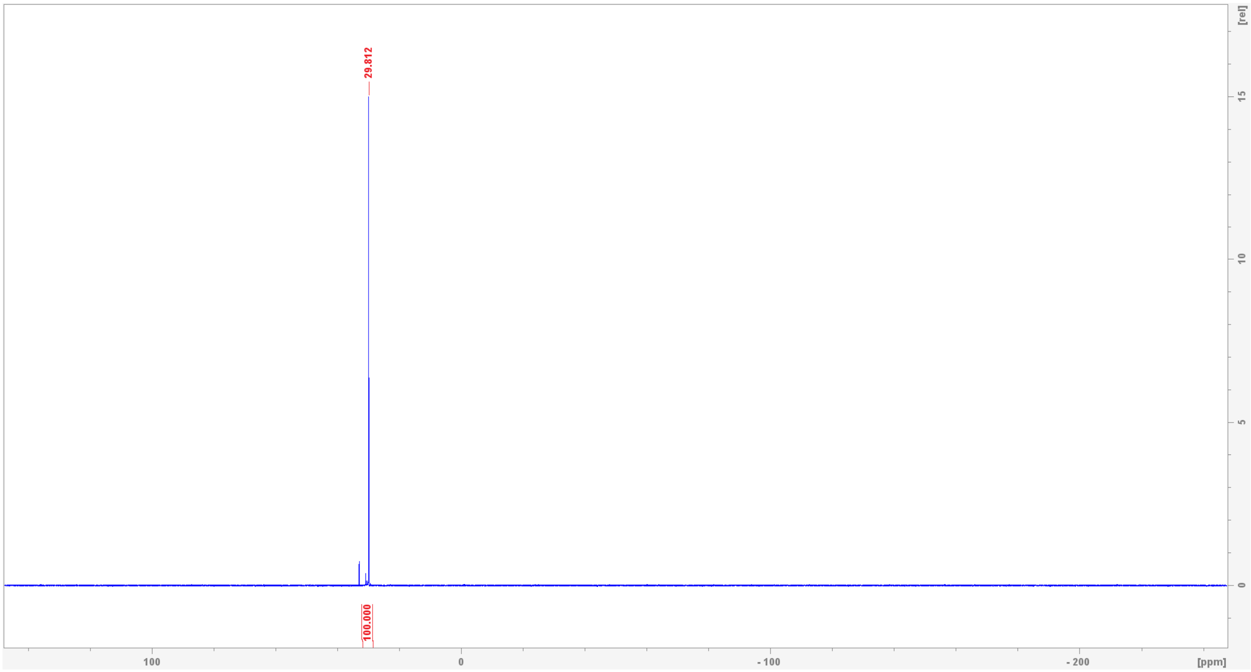

29.812
100.000
100
0
- 100
- 200
[ppm]
[rel]
15
10
5
0

Compound **2d**: **^{1}H NMR** (400 MHz, $CDCl_3$), **^{13}C NMR** (100.6 MHz, $CDCl_3$) and **^{31}P NMR** (161.9 MHz, $CDCl_3$).

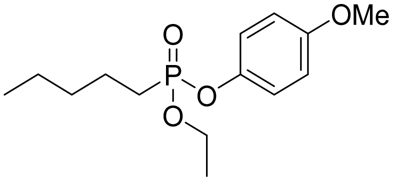

2d

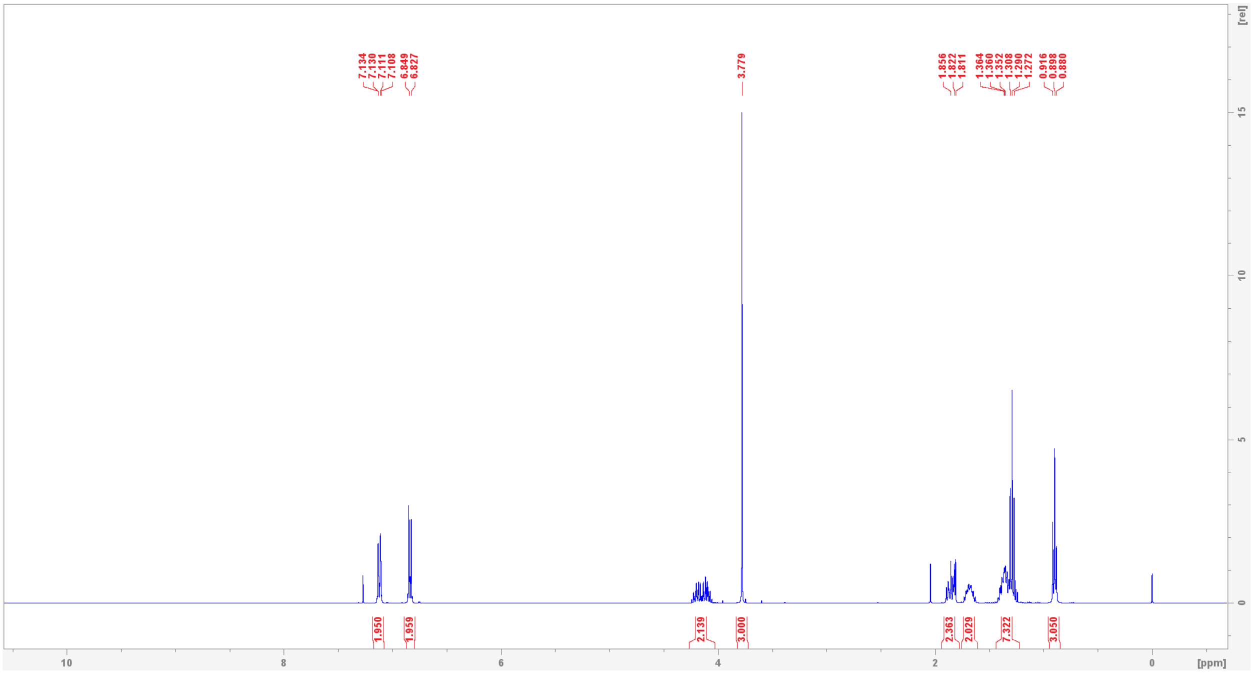

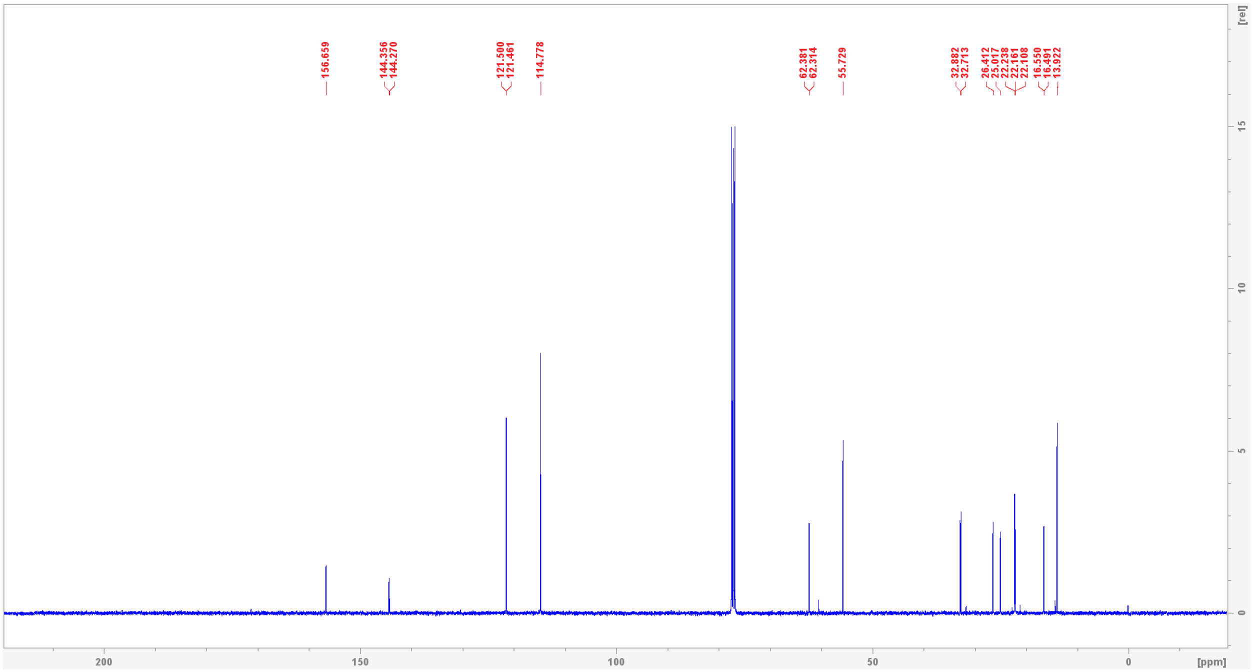

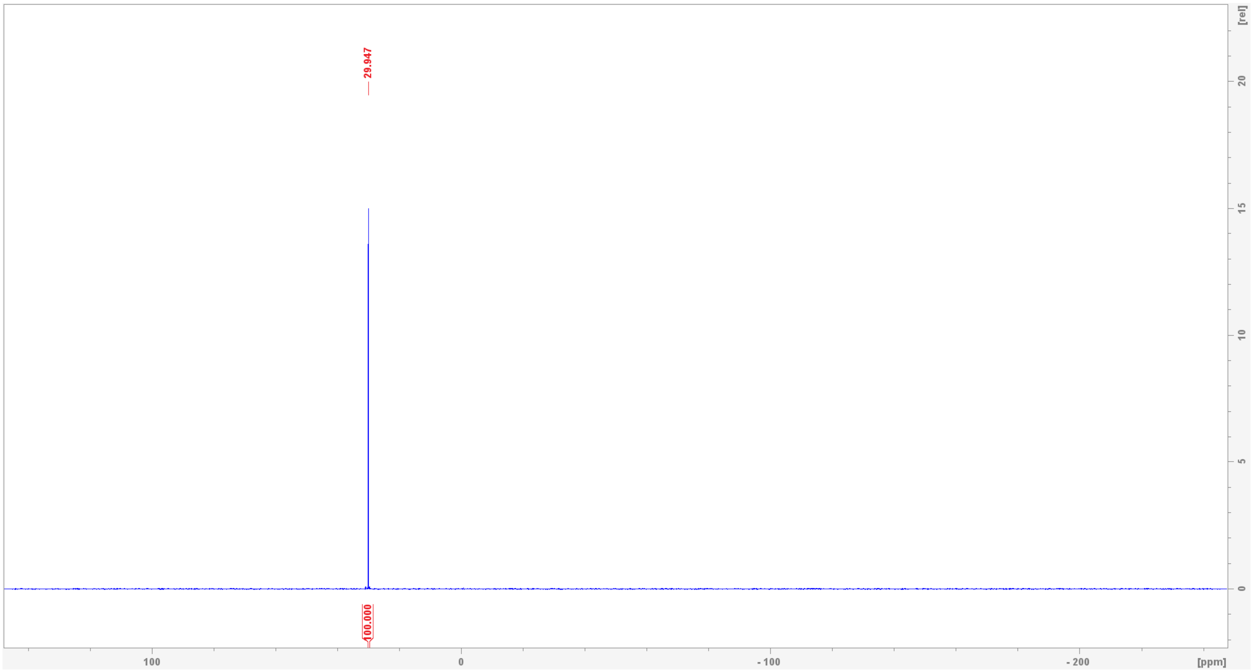

29.947
100.000
100
0
- 100
- 200
[ppm]
[rel]

Compound **2e**: ^{1}**H NMR** (400 MHz, $CDCl_3$), ^{13}**C NMR** (100.6 MHz, $CDCl_3$) and ^{31}**P NMR** (161.9 MHz, $CDCl_3$).

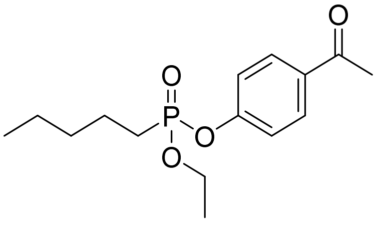

2e

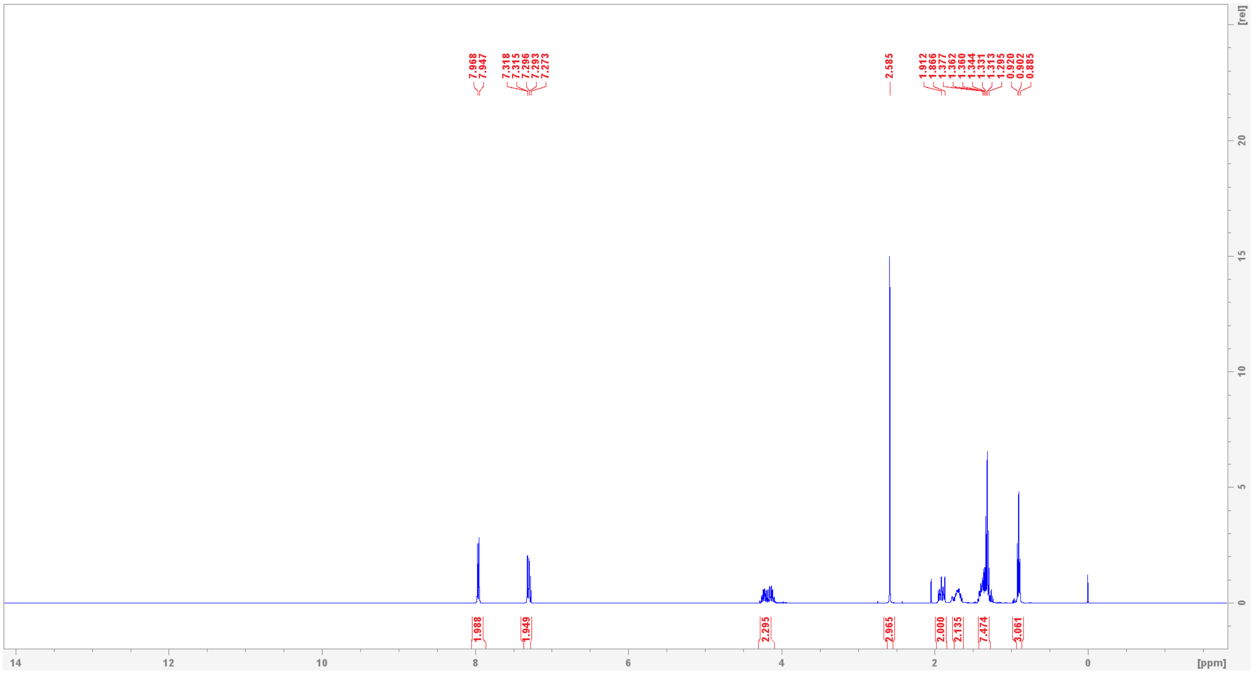

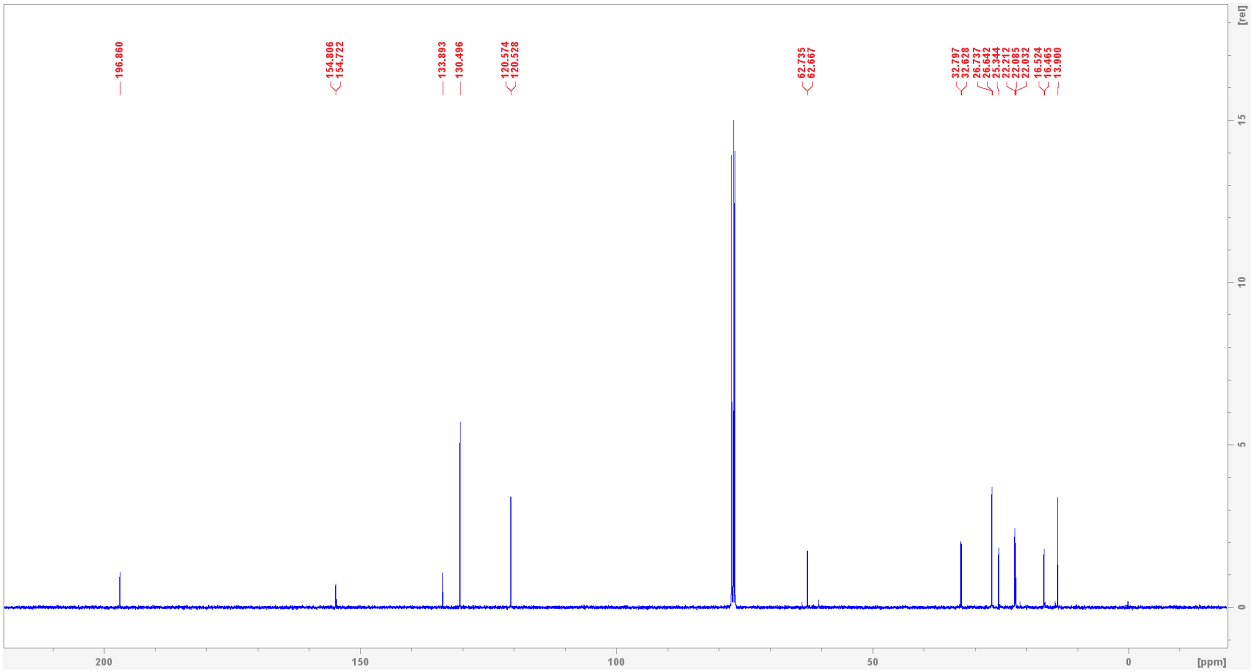

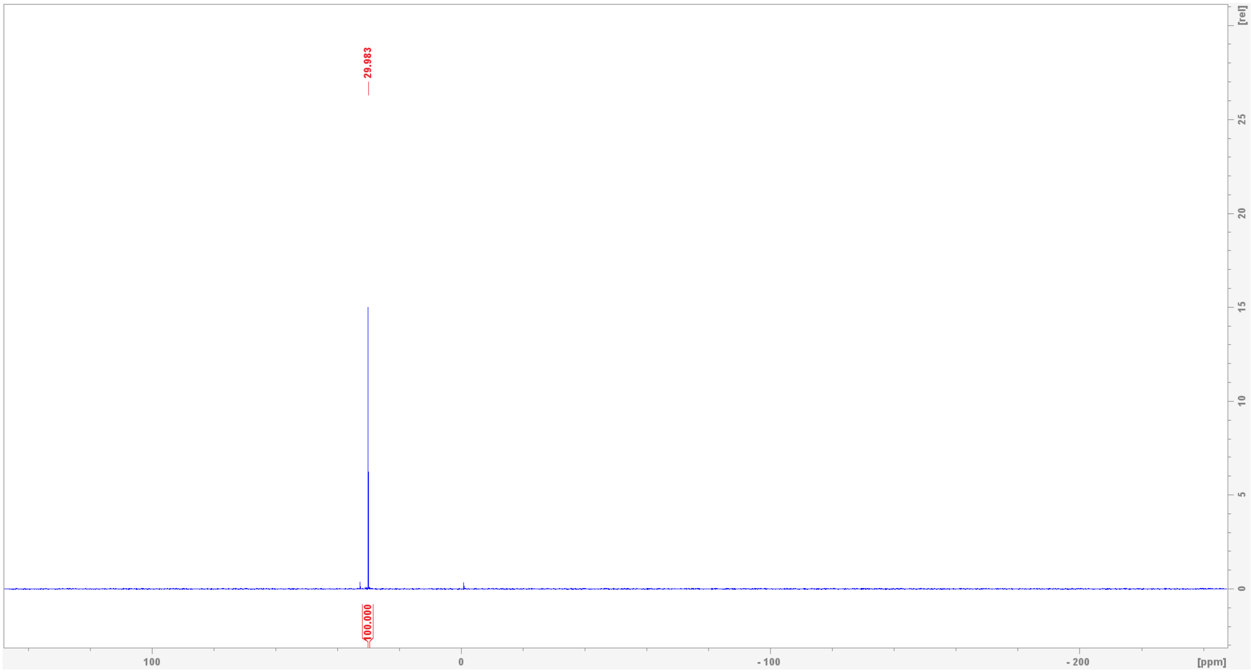

29.983
100.000
100
0
-100
-200
[ppm]
[rel]

Compound **2f**: **^{1}H NMR** (400 MHz, $CDCl_3$), **^{13}C NMR** (100.6 MHz, $CDCl_3$) and **^{31}P NMR** (161.9 MHz, $CDCl_3$).

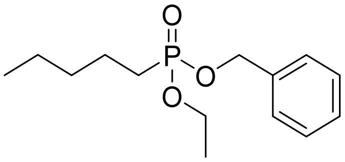

2f

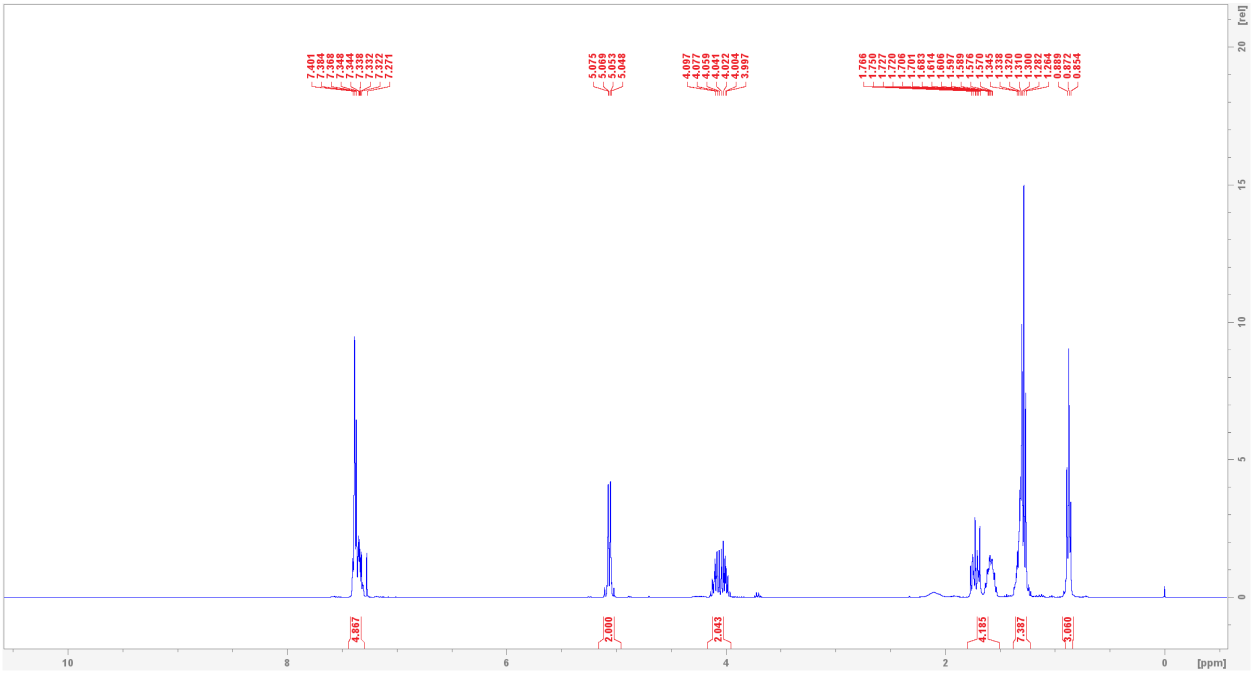

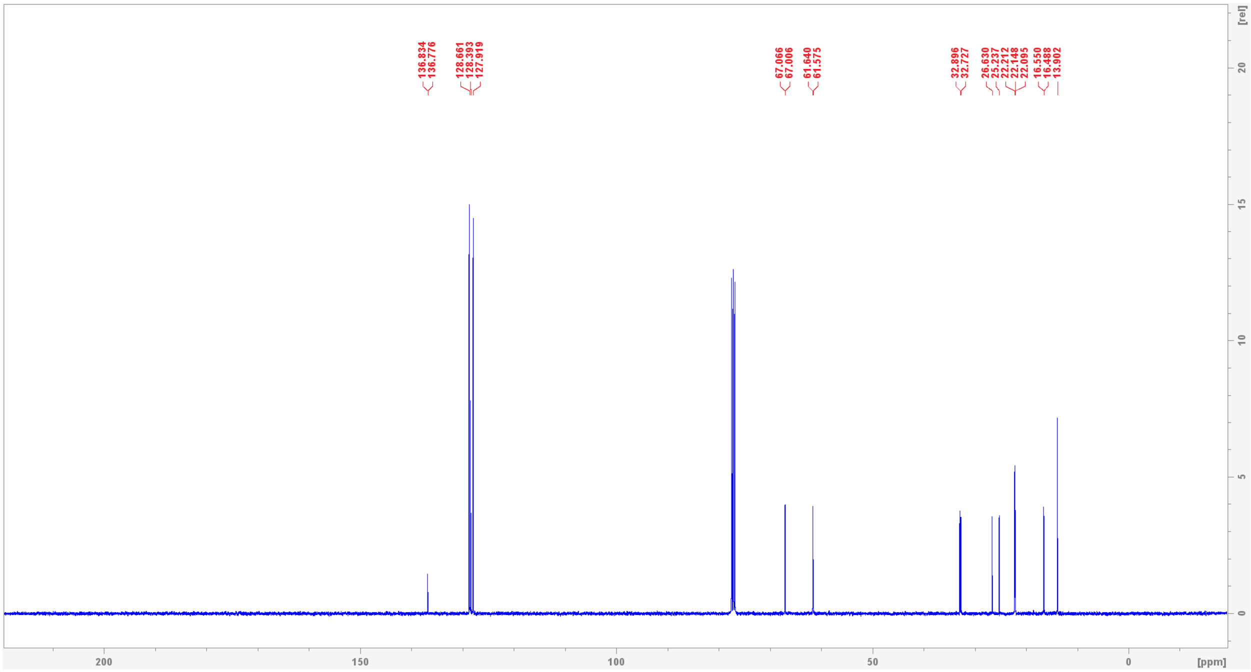

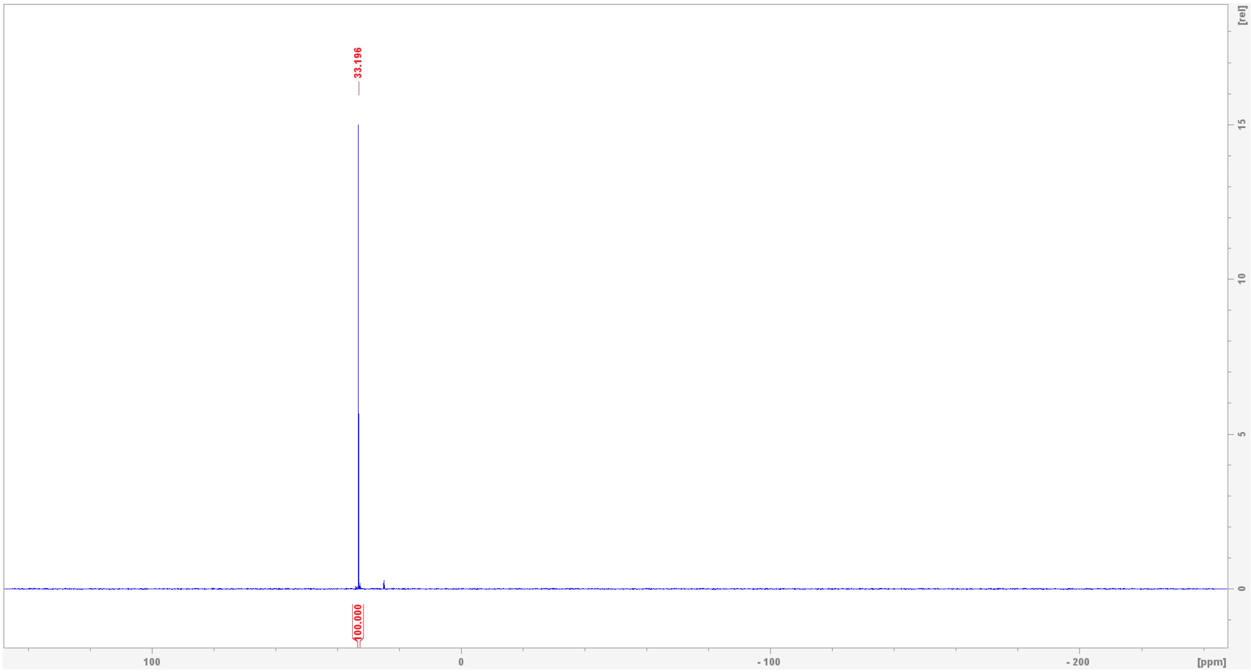
33.196
100.000
100
0
- 100
- 200
[ppm]
[rel]
15
10
5
0

Compound **2g**: ^{1}**H NMR** (400 MHz, $CDCl_3$), ^{13}**C NMR** (100.6 MHz, $CDCl_3$) and ^{31}**P NMR** (161.9 MHz, $CDCl_3$).

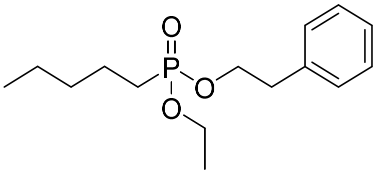

2g

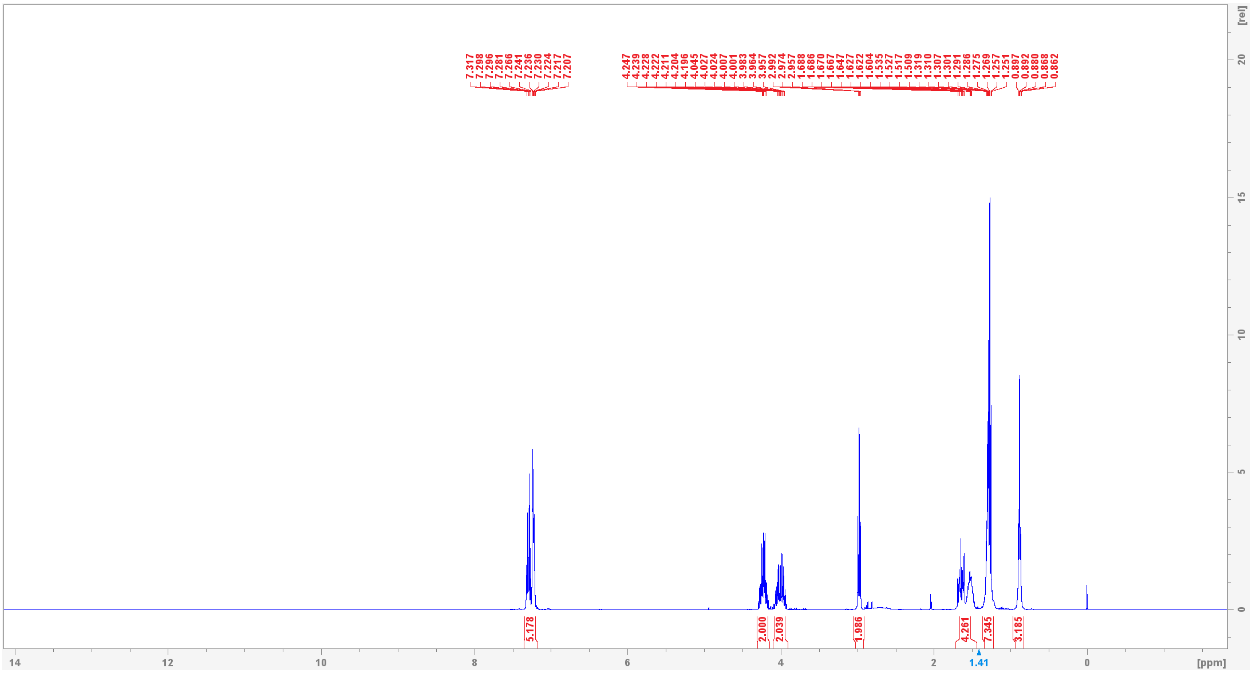

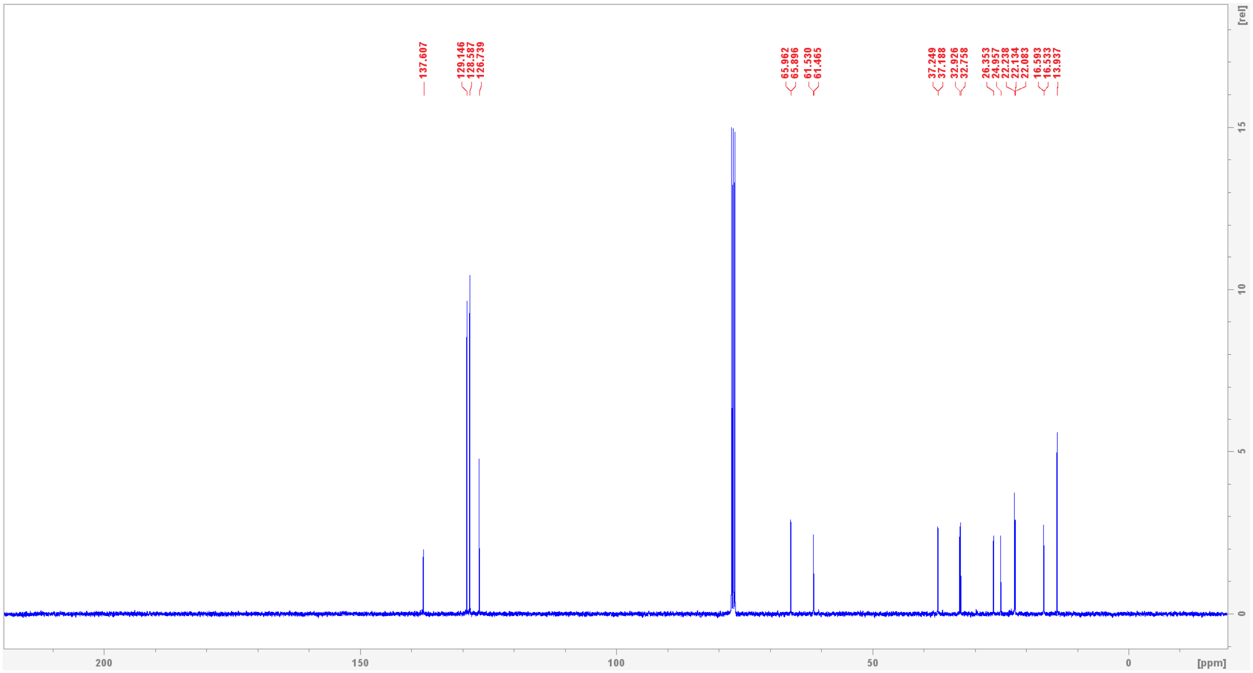

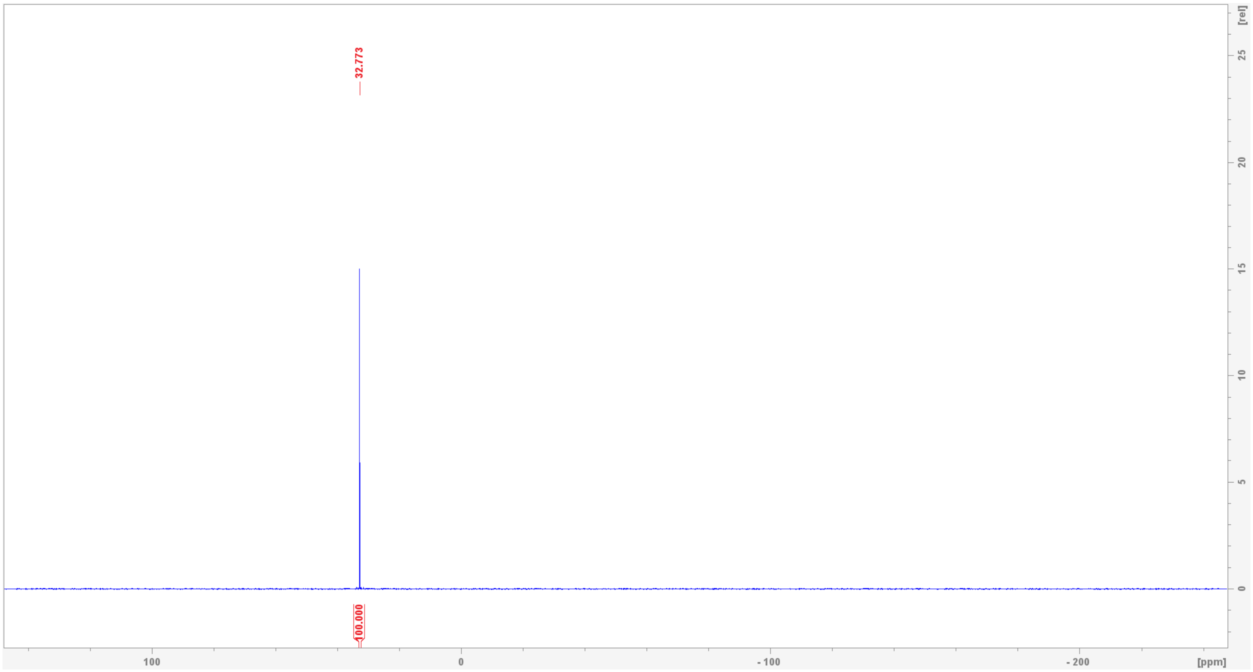

32.773
100.000
[rel]
25
20
15
10
5
0
100
0
- 100
- 200
[ppm]

Compound **3**: [1]**H NMR** (400 MHz, $CDCl_3$), [13]**C NMR** (100.6 MHz, $CDCl_3$) and [31]**P NMR** (161.9 MHz, $CDCl_3$).

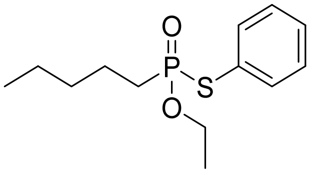

3

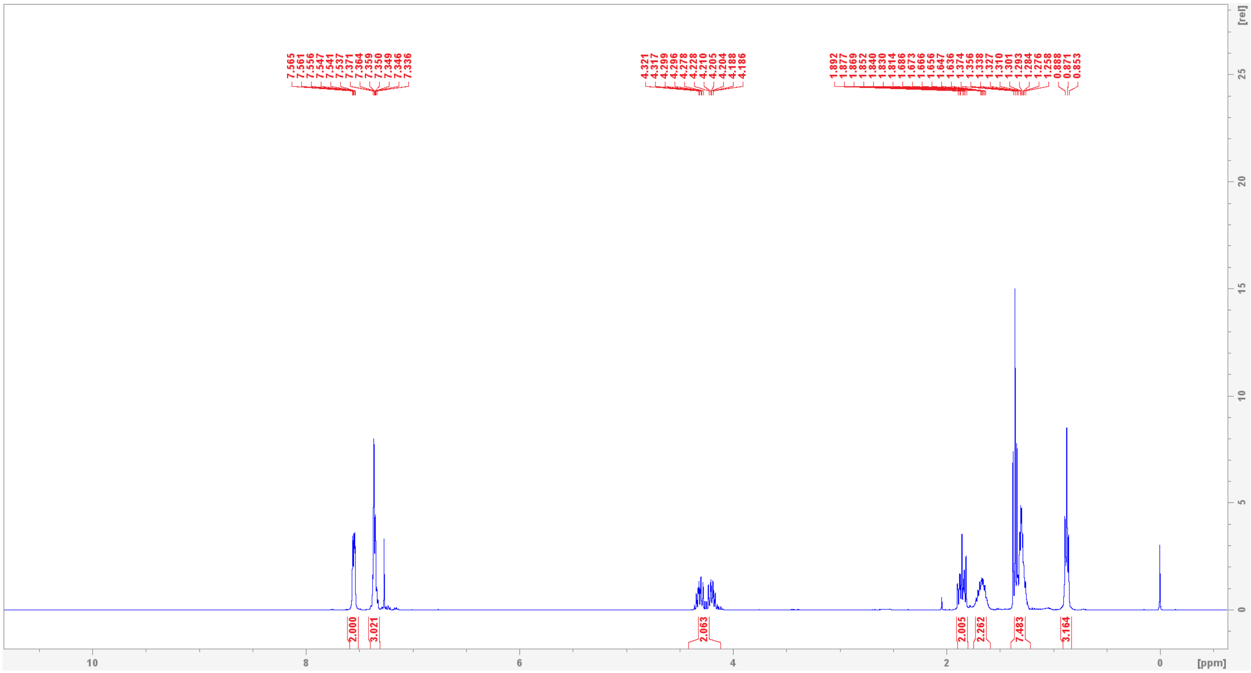

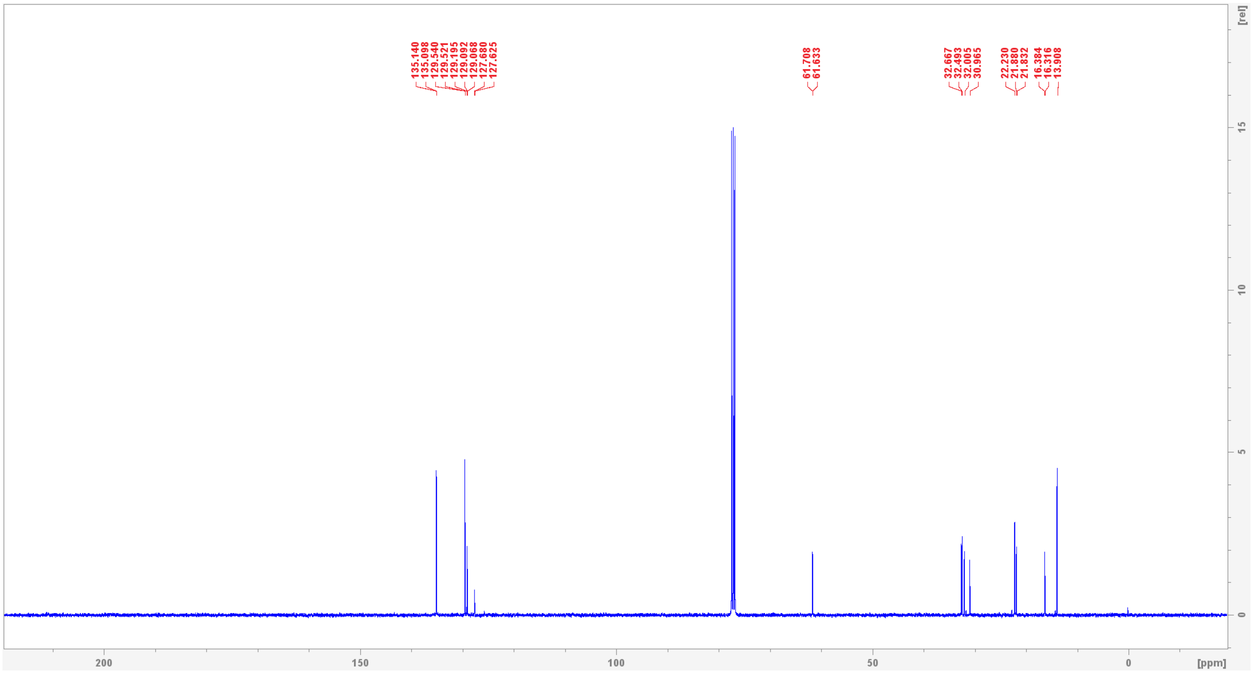

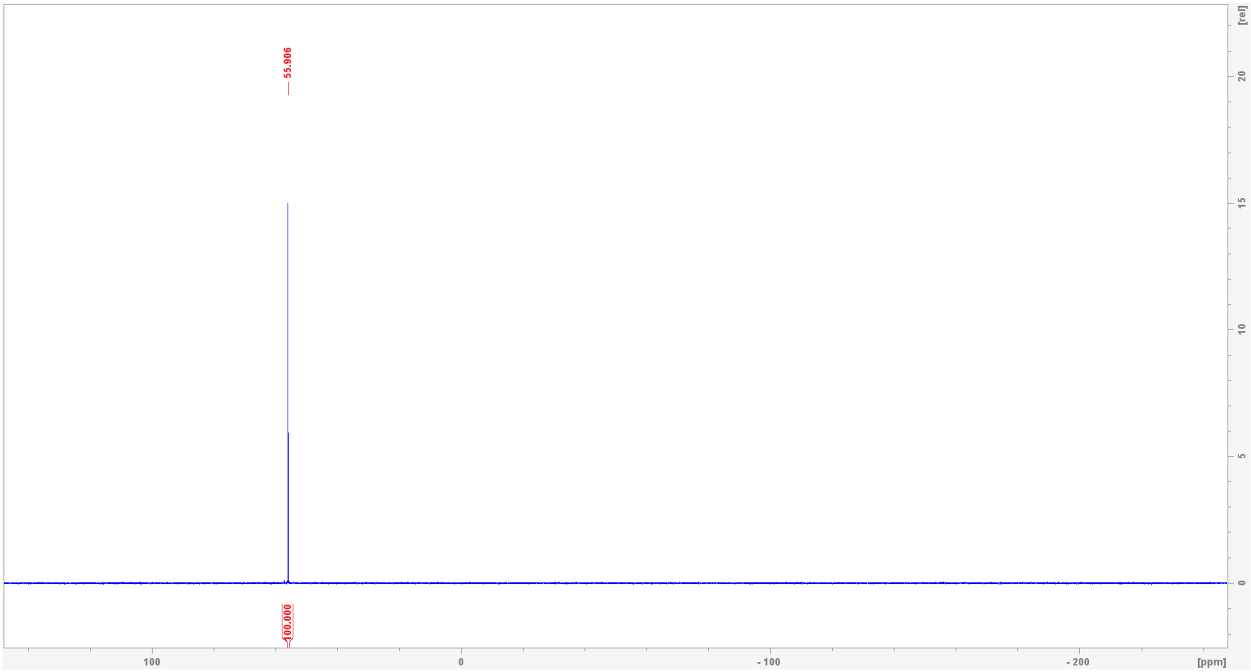

55.906
100.000
100
0
- 100
- 200
[ppm]
[rel]
20
15
10
5
0

Compound **4a**: **^{1}H NMR** (400 MHz, $CDCl_3$), **^{13}C NMR** (100.6 MHz, $CDCl_3$) and **^{31}P NMR** (161.9 MHz, $CDCl_3$).

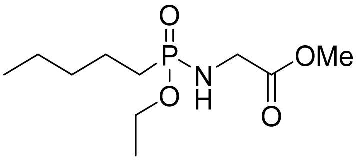

4a

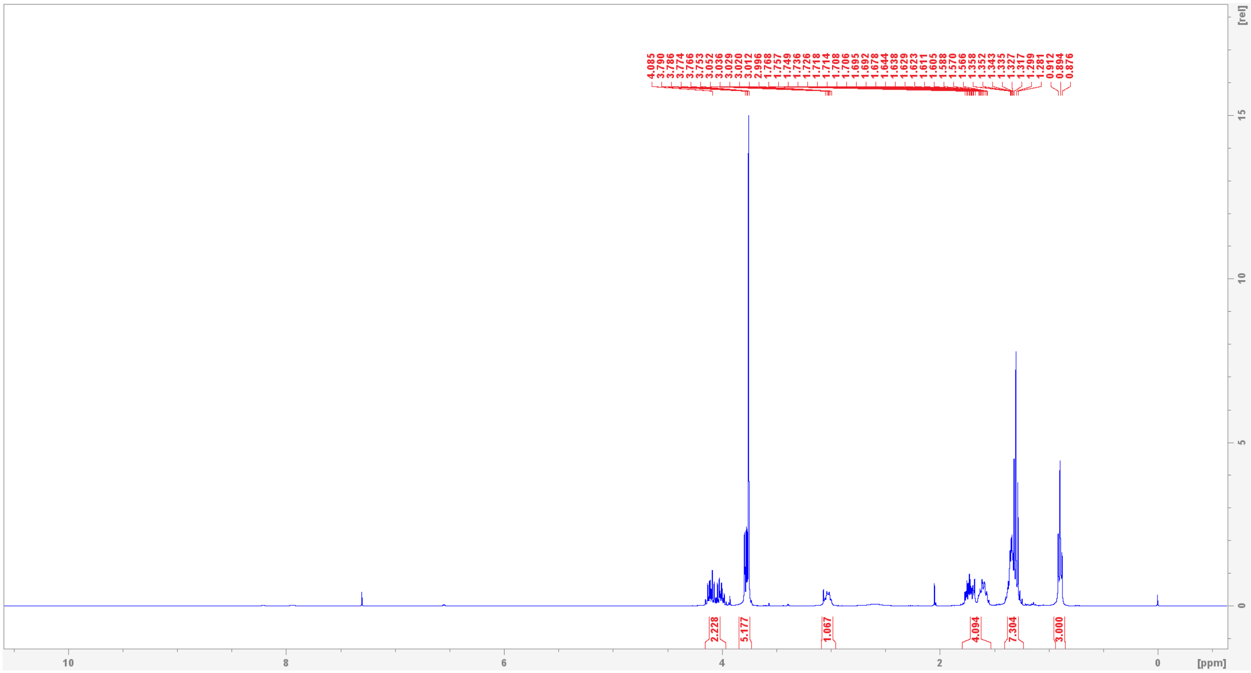

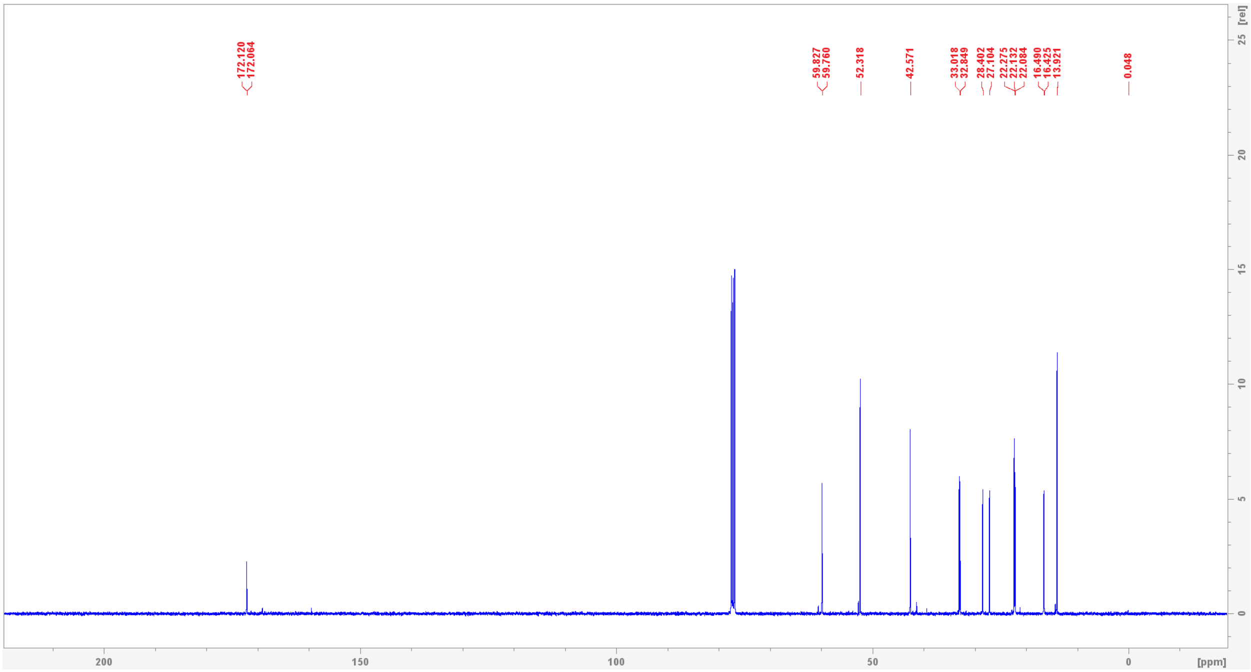

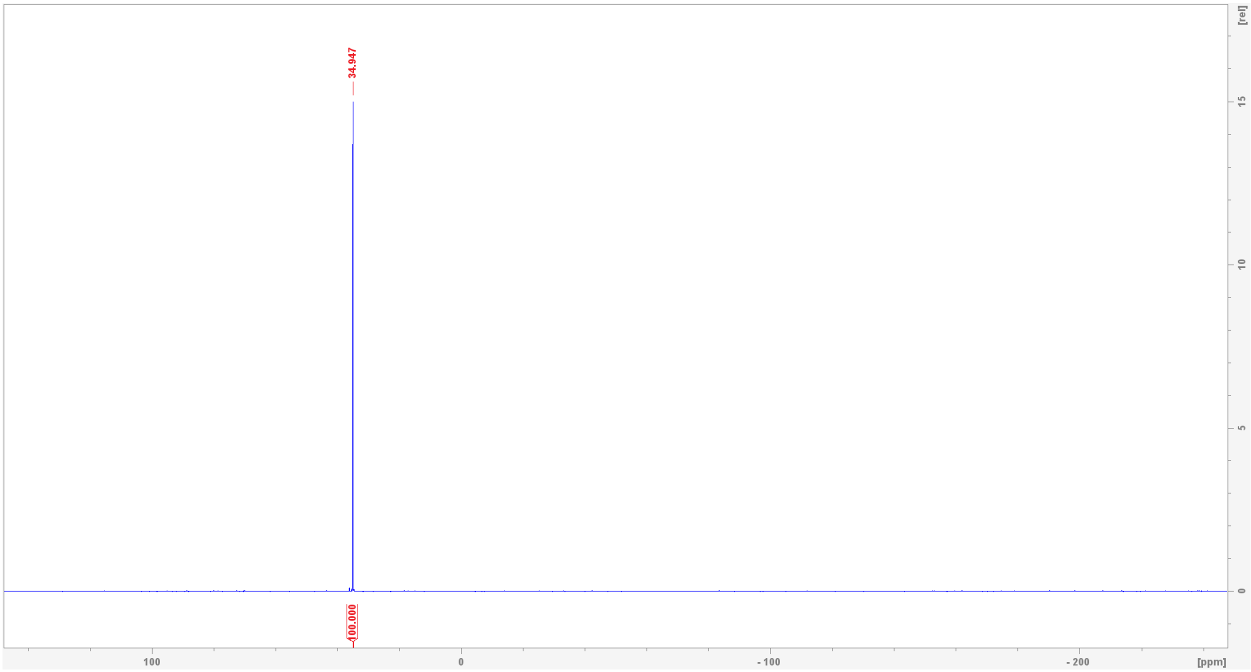

34.947
100.000
[rel]
15
10
5
0
100
0
- 100
- 200
[ppm]

Compound **4b**: ^{1}**H NMR** (400 MHz, $CDCl_3$), ^{13}**C NMR** (100.6 MHz, $CDCl_3$) and ^{31}**P NMR** (161.9 MHz, $CDCl_3$).

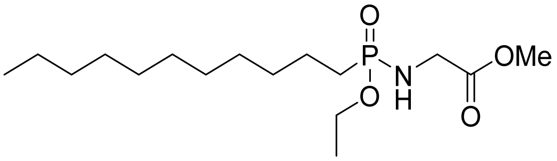

4b

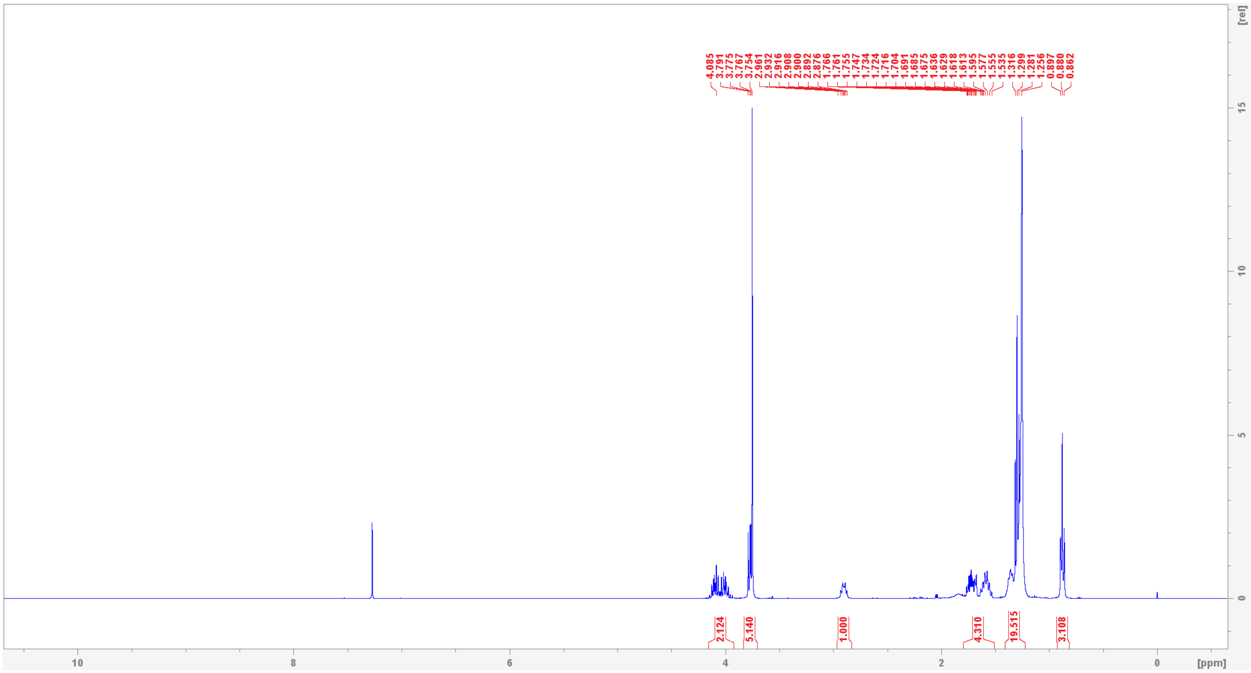

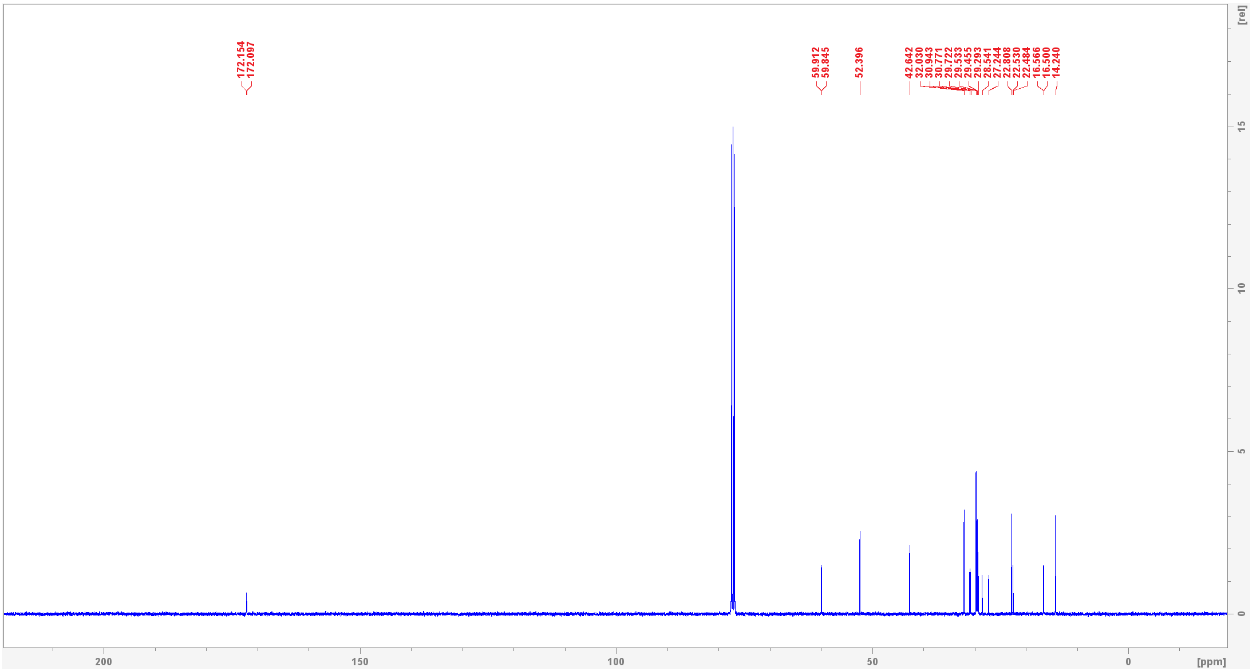

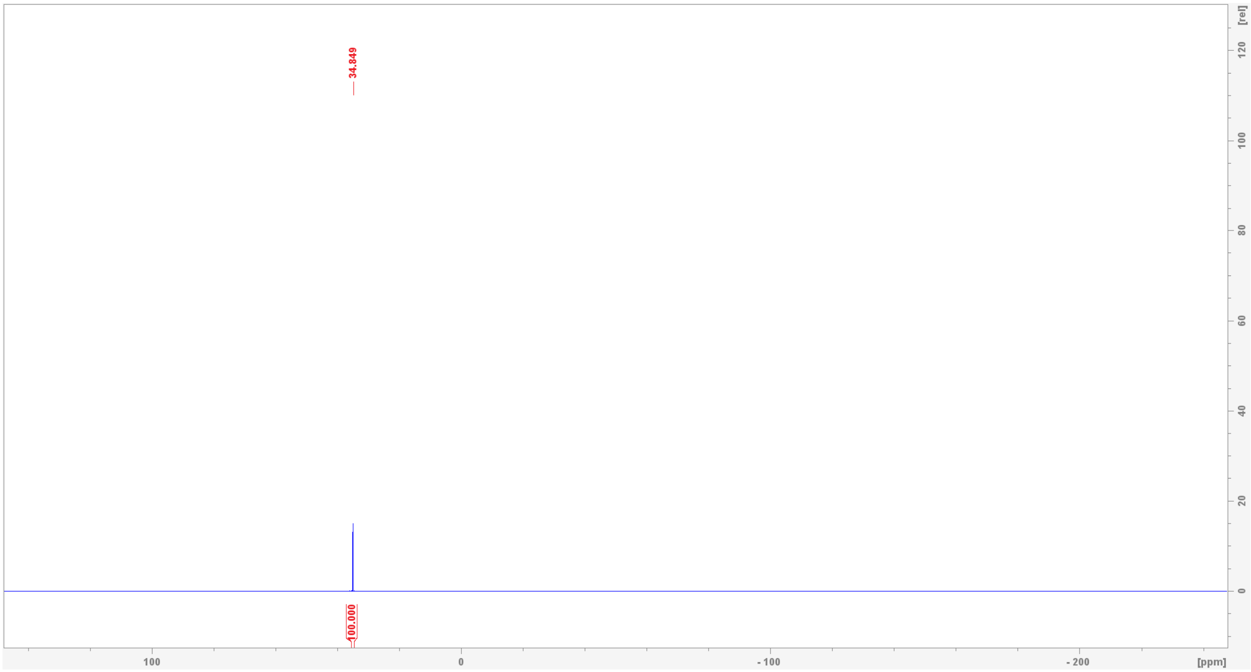
34.849
100.000
100
0
- 100
- 200
[ppm]
[rel]
120
100
80
60
40
20
0

Compound **4c**: **^{1}H NMR** (400 MHz, $CDCl_3$), **^{13}C NMR** (100.6 MHz, $CDCl_3$) and **^{31}P NMR** (161.9 MHz, $CDCl_3$).

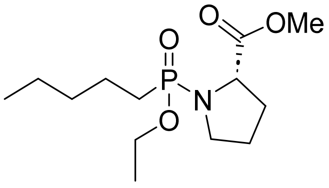

4c

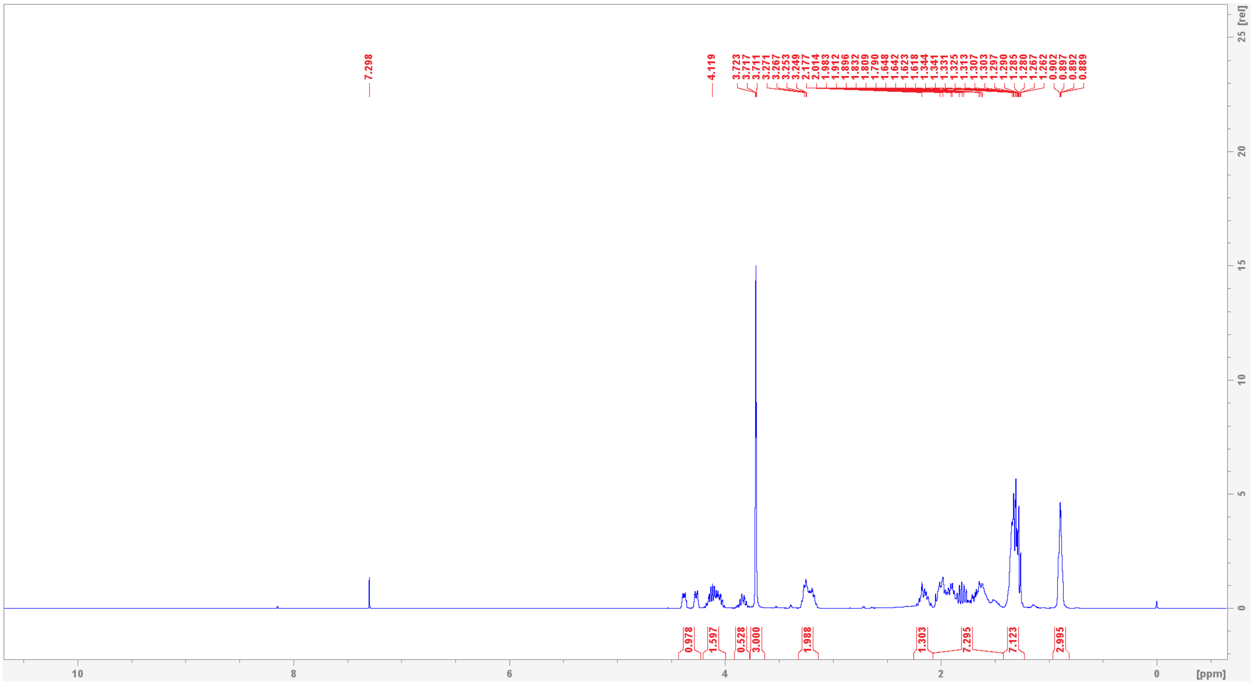

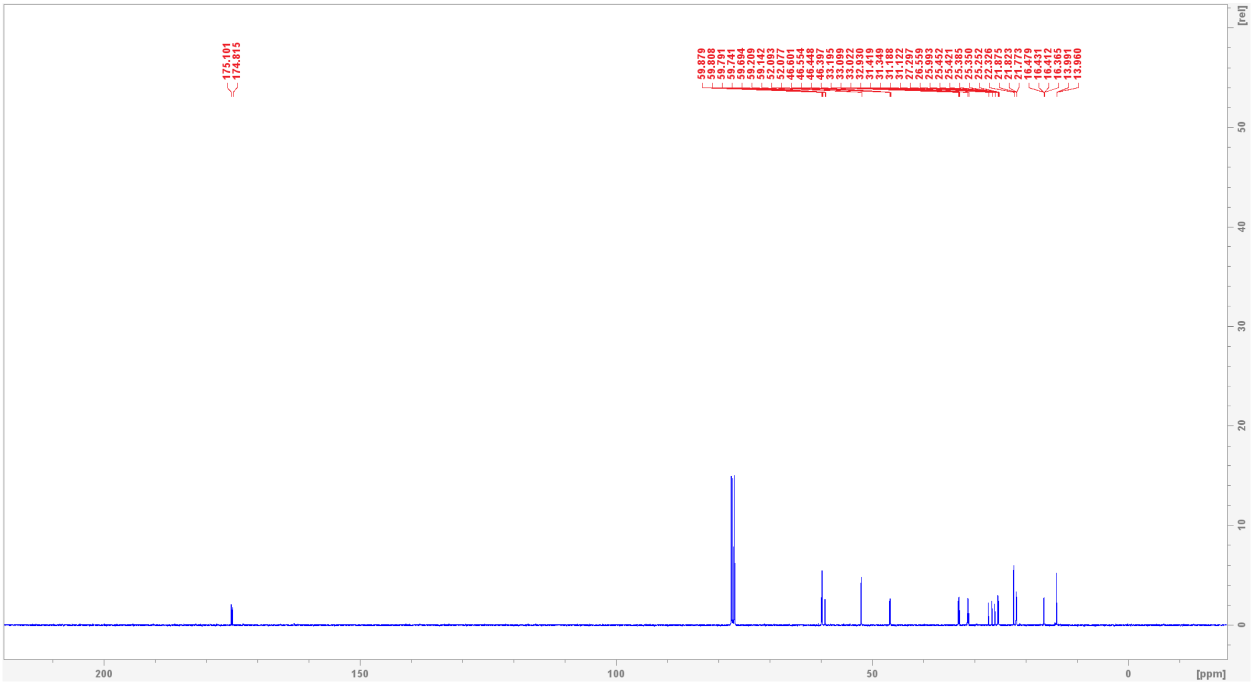

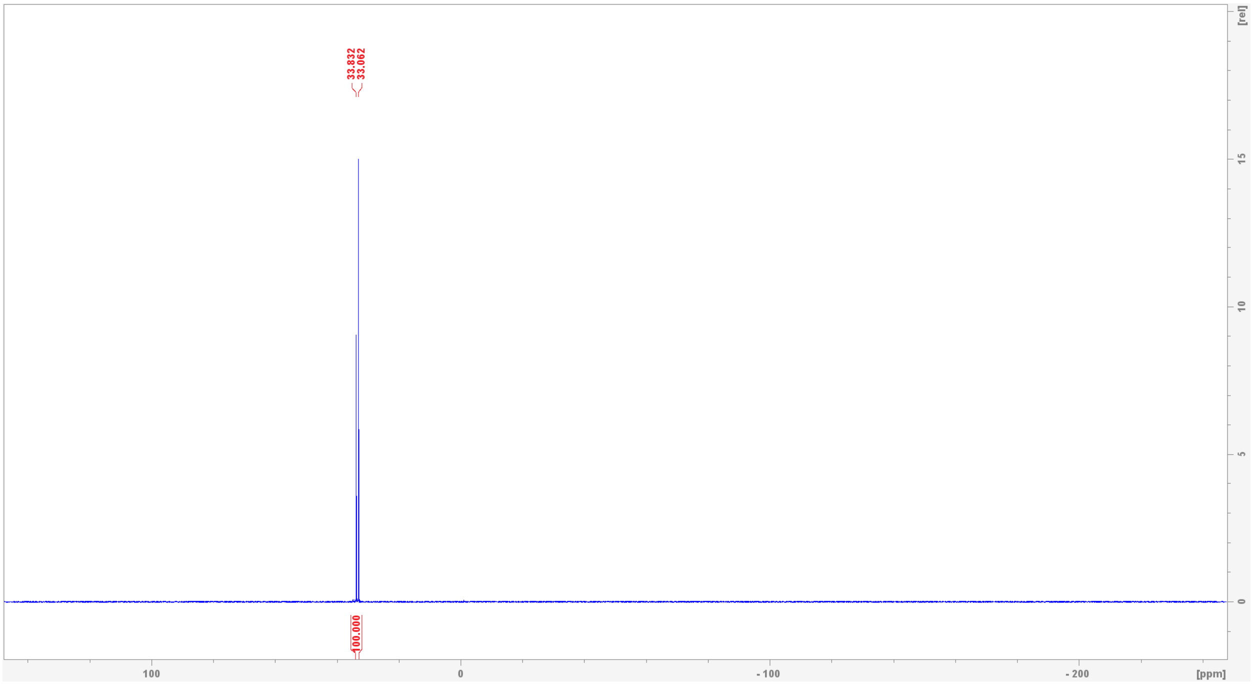

33.832
33.062
100.000
100
0
- 100
- 200
[ppm]
[rel]
15
10
5
0

Compound **4d**: ^{1}**H NMR** (400 MHz, $CDCl_3$), ^{13}**C NMR** (100.6 MHz, $CDCl_3$) and ^{31}**P NMR** (161.9 MHz, $CDCl_3$).

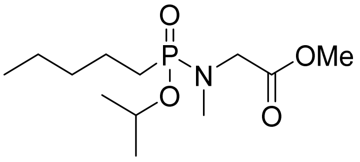

4d

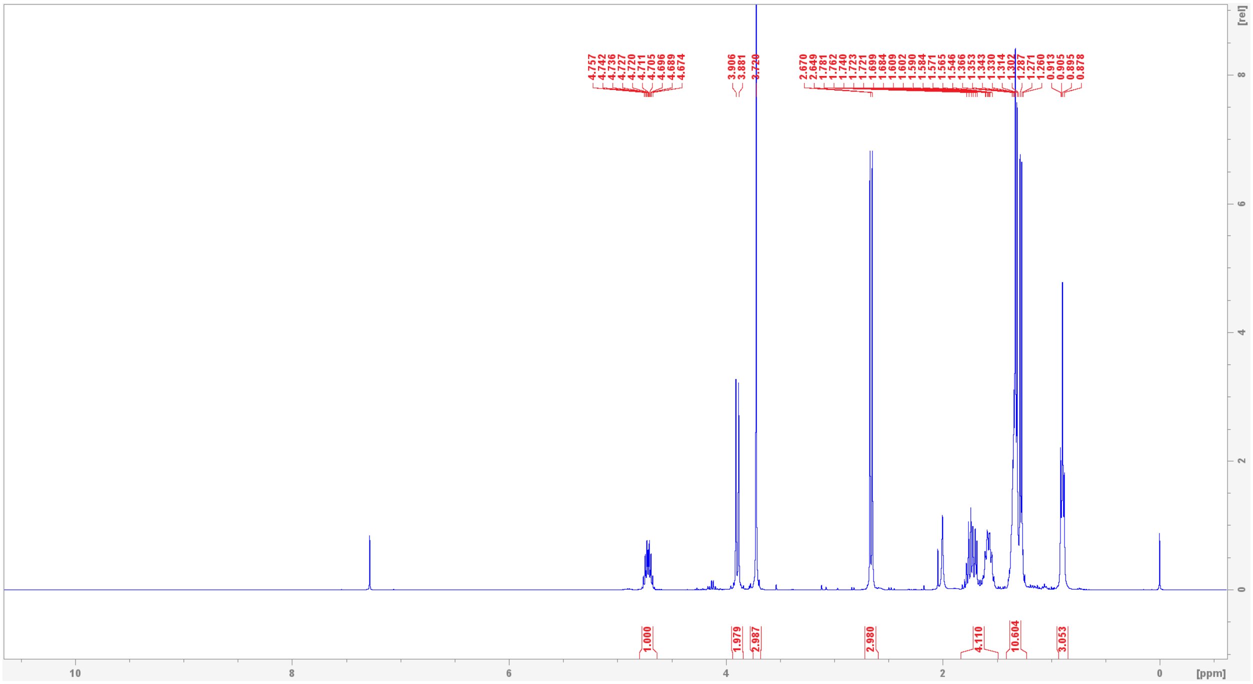

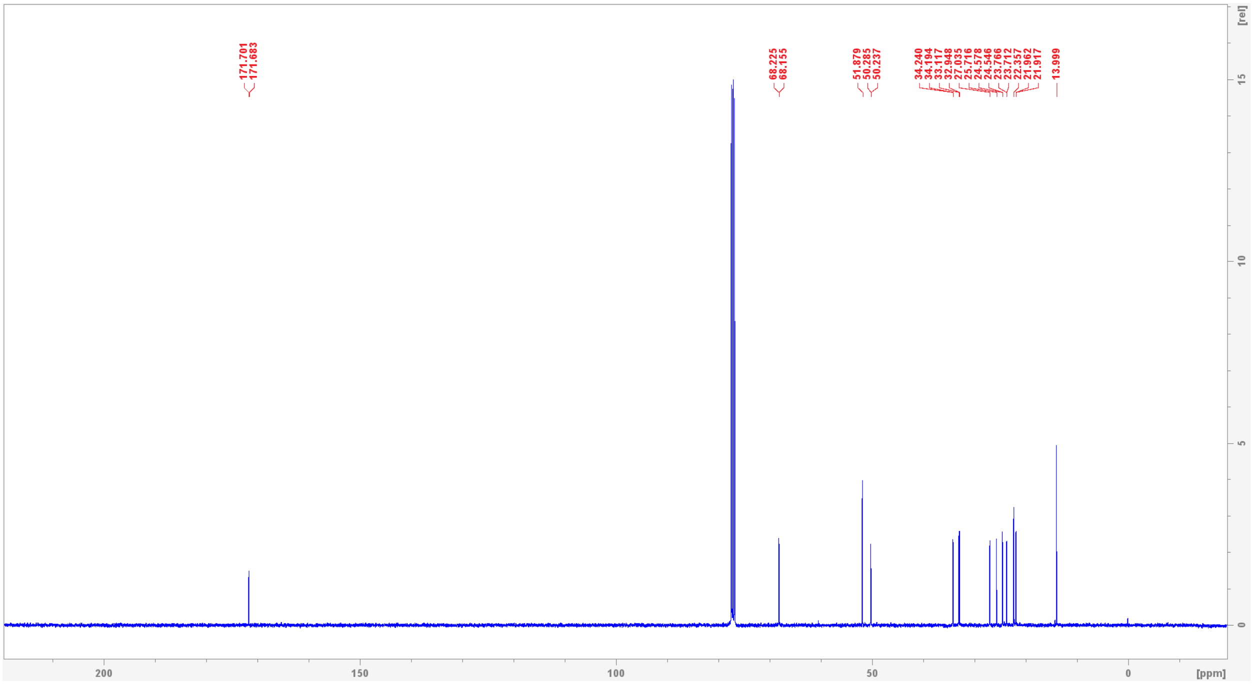

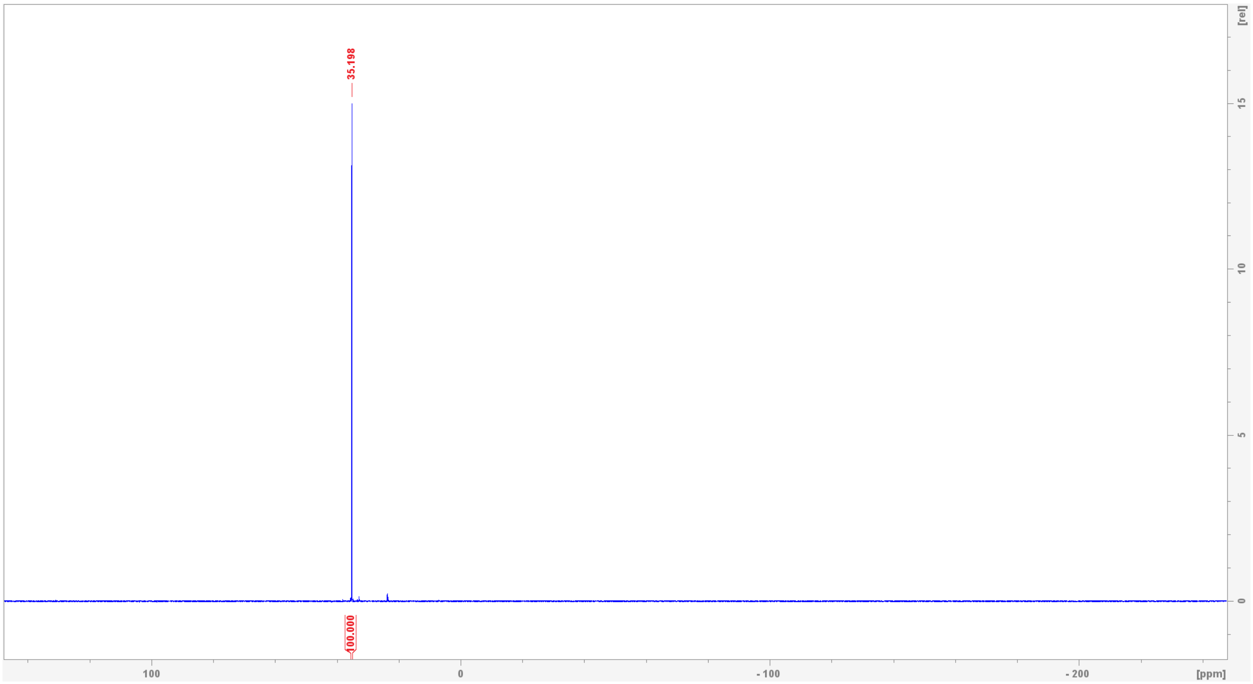

35.198
100.000
100
0
- 100
- 200
[ppm]
[rel]
15
10
5
0

Compound **4e**: ^{1}**H NMR** (400 MHz, $CDCl_3$), ^{13}**C NMR** (100.6 MHz, $CDCl_3$) and ^{31}**P NMR** (161.9 MHz, $CDCl_3$).

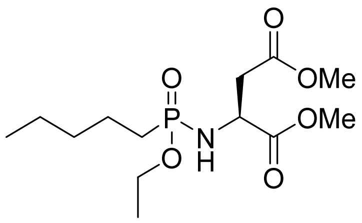

4e

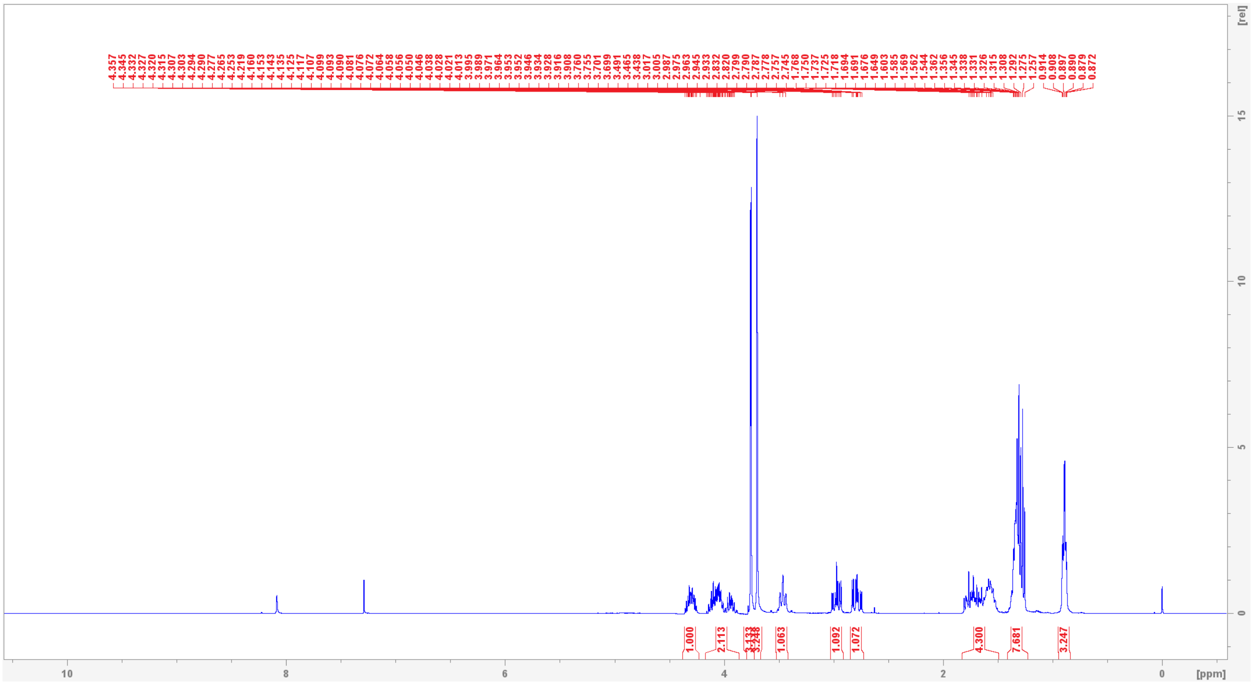

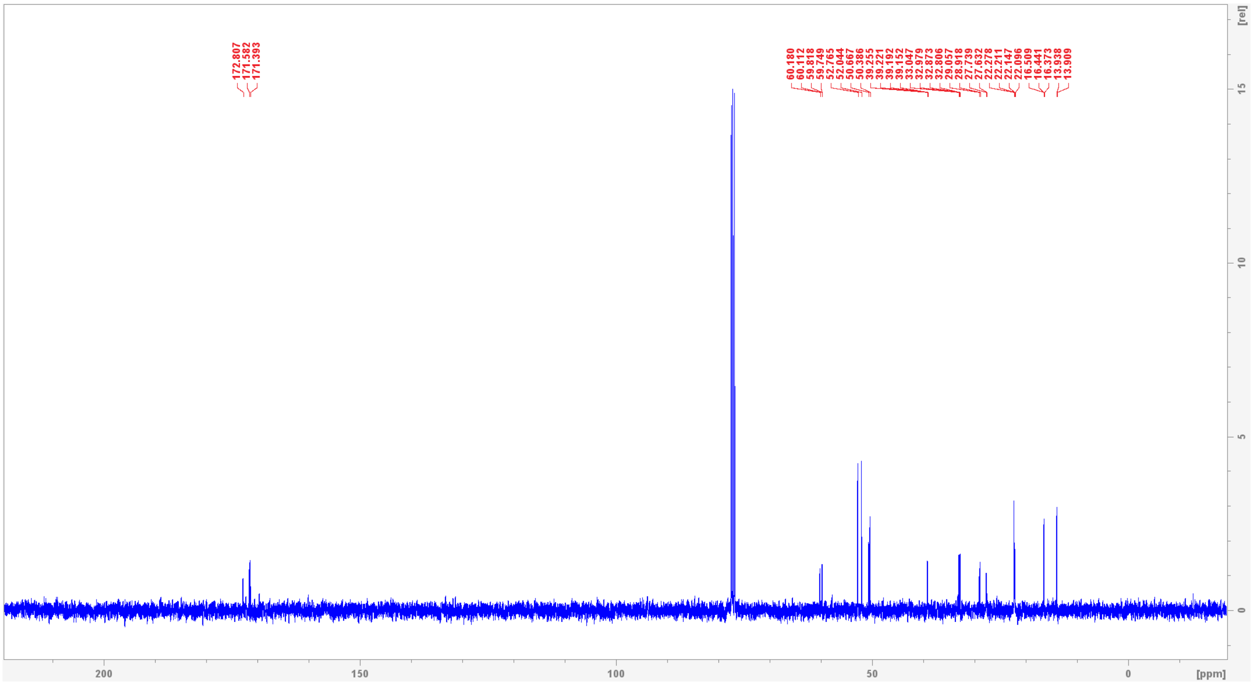

172.807
171.582
171.393
60.180
60.112
59.818
59.749
52.765
52.044
50.667
50.386
39.255
39.221
39.192
39.152
33.047
32.979
32.873
32.806
29.057
28.918
27.739
27.632
22.278
22.211
22.147
22.096
16.509
16.441
16.373
13.938
13.909
[rel]
[ppm]

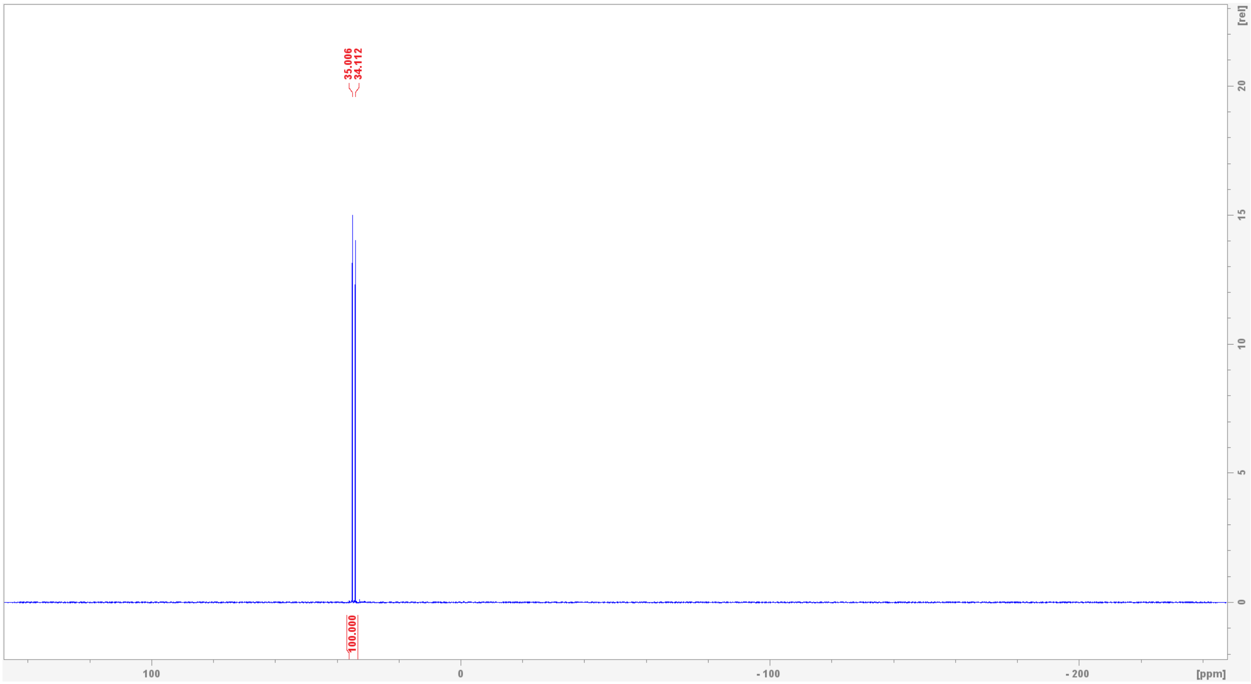

35.006
34.112
100.000
[rel]
[ppm]

Compound **4f**: **^{1}H NMR** (400 MHz, $CDCl_3$), **^{13}C NMR** (100.6 MHz, $CDCl_3$) and **^{31}P NMR** (161.9 MHz, $CDCl_3$).

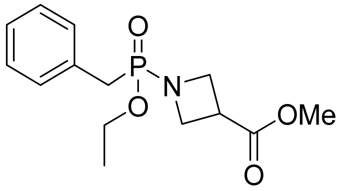

4f

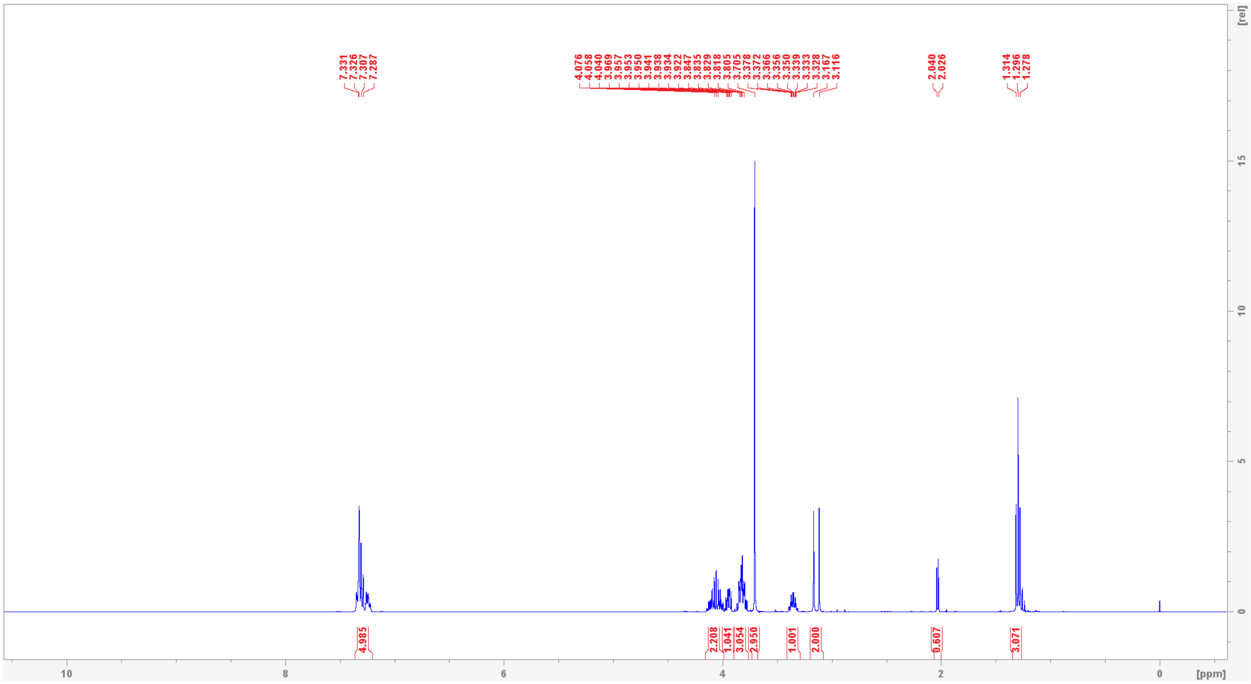

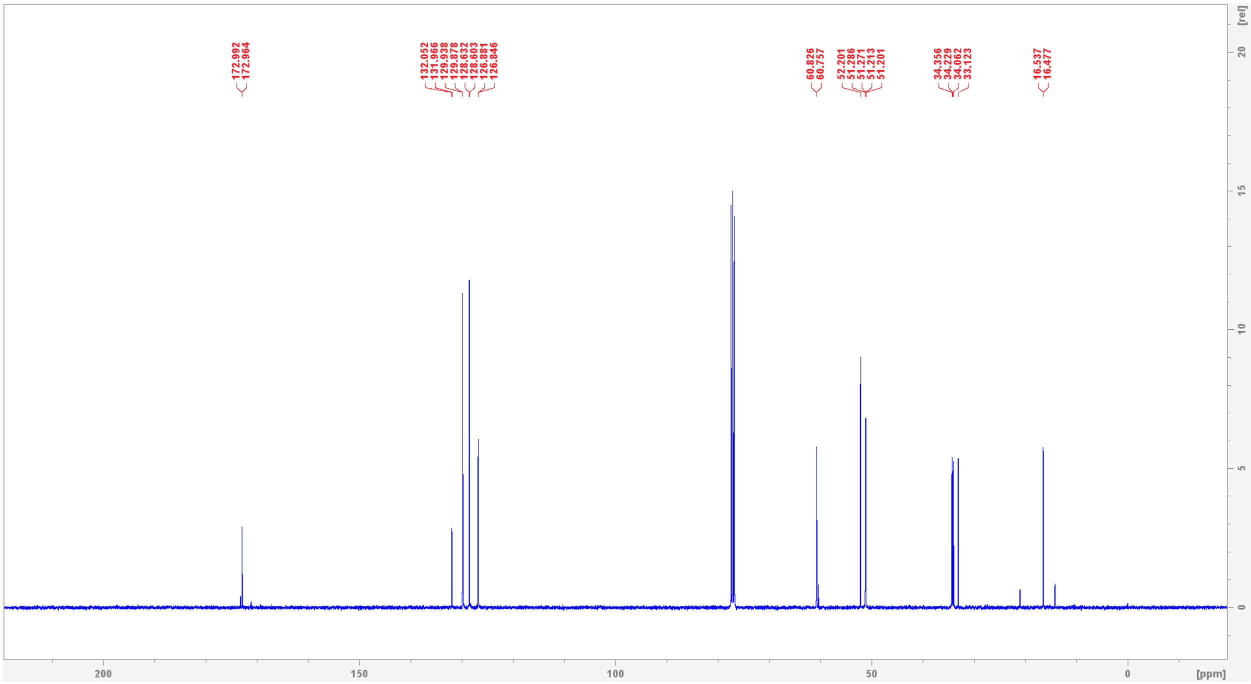

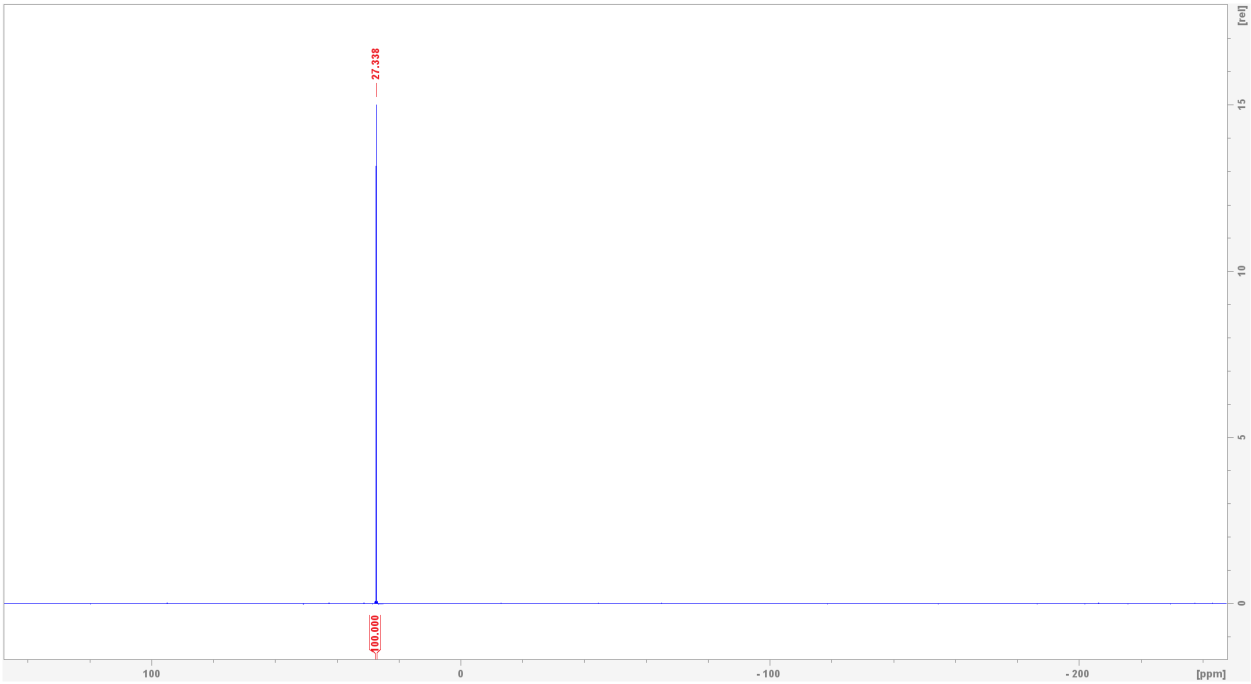

27.338
100.000
100
0
- 100
- 200
[ppm]
[rel]
15
10
5
0

Compound **4g**: ^{1}**H NMR** (400 MHz, $CDCl_3$), ^{13}**C NMR** (100.6 MHz, $CDCl_3$) and ^{31}**P NMR** (161.9 MHz, $CDCl_3$).

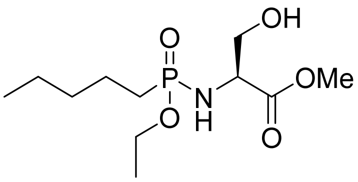

4g

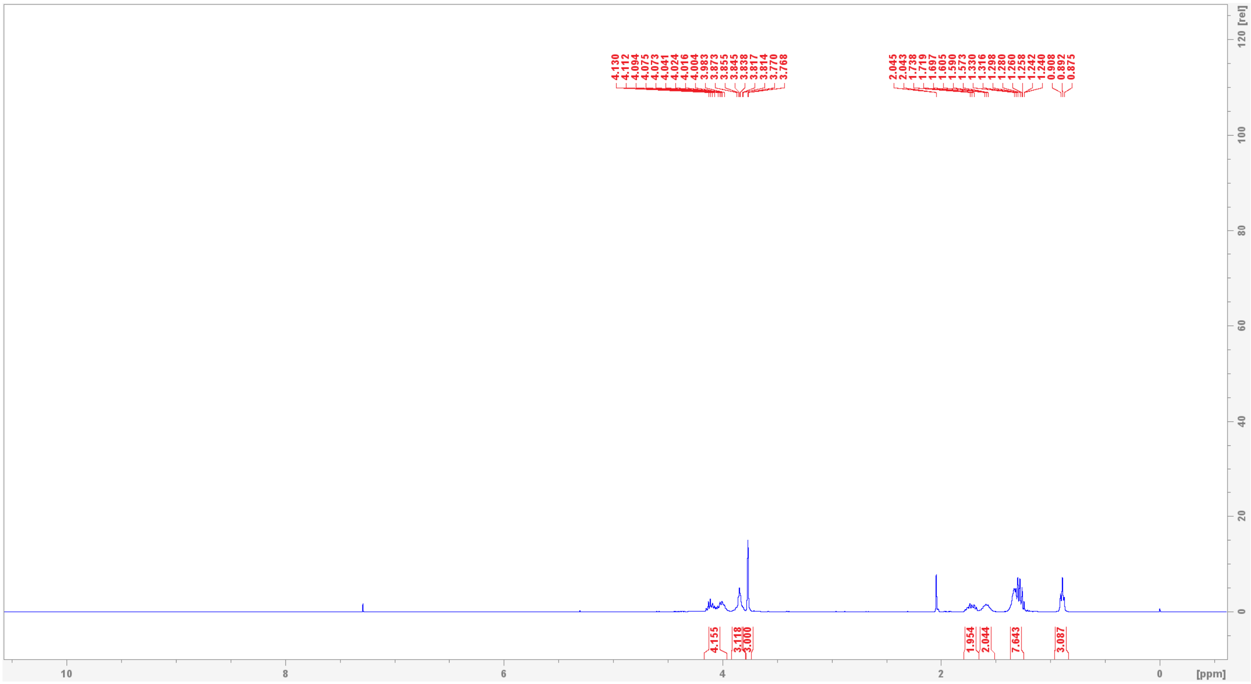

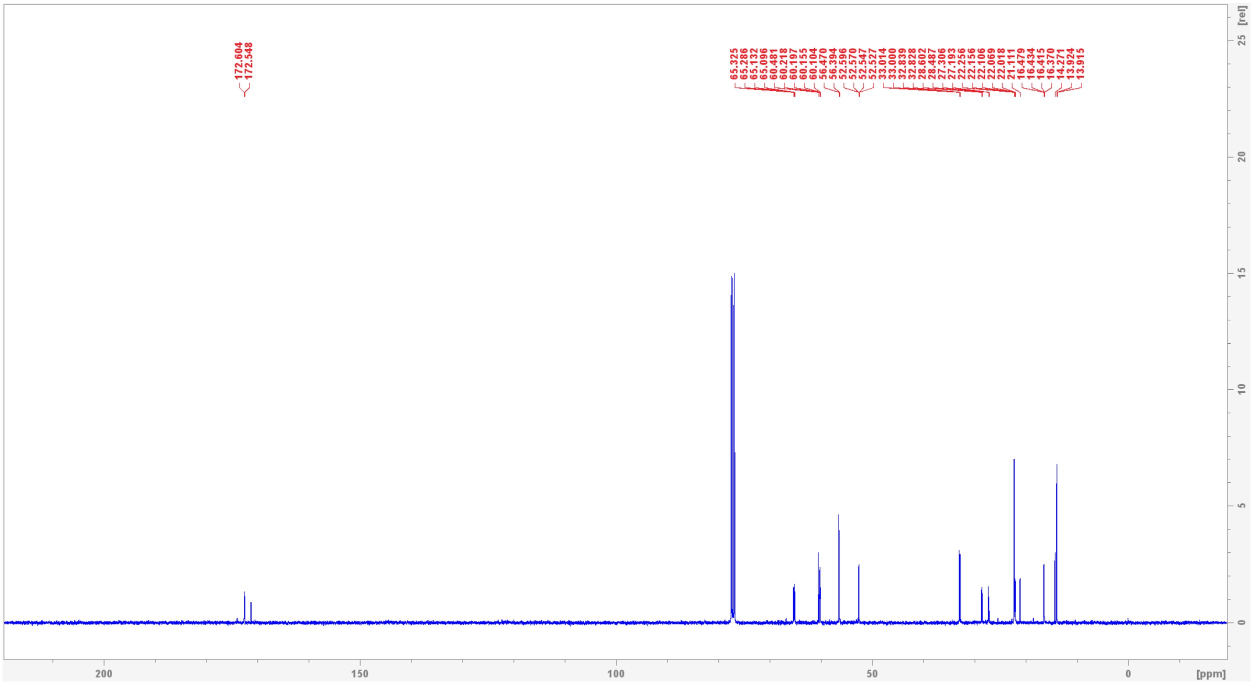

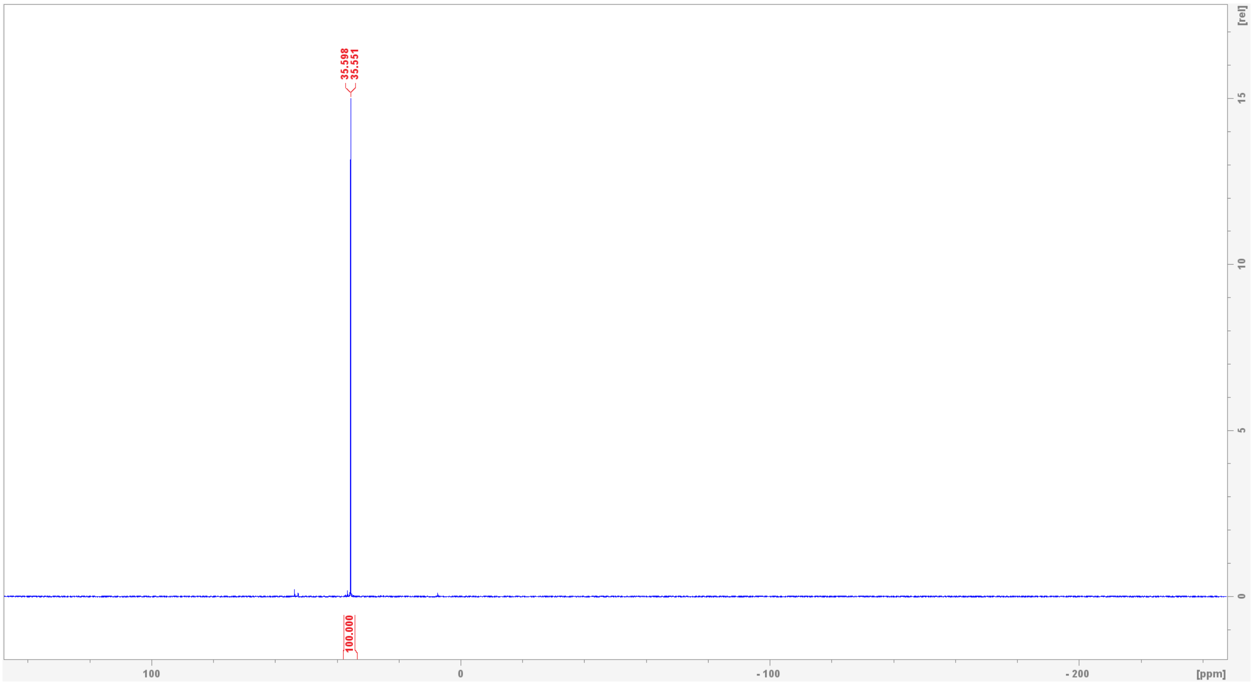
35.598
35.551
100.000
100
0
- 100
- 200
[ppm]
[rel]
15
10
5
0

Compound **4h**: ^{1}**H NMR** (400 MHz, $CDCl_3$), ^{13}**C NMR** (100.6 MHz, $CDCl_3$) and ^{31}**P NMR** (161.9 MHz, $CDCl_3$).

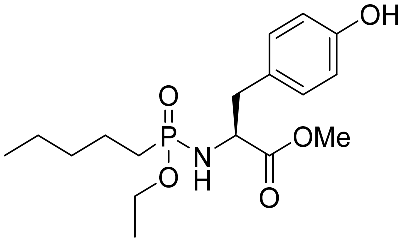

4h

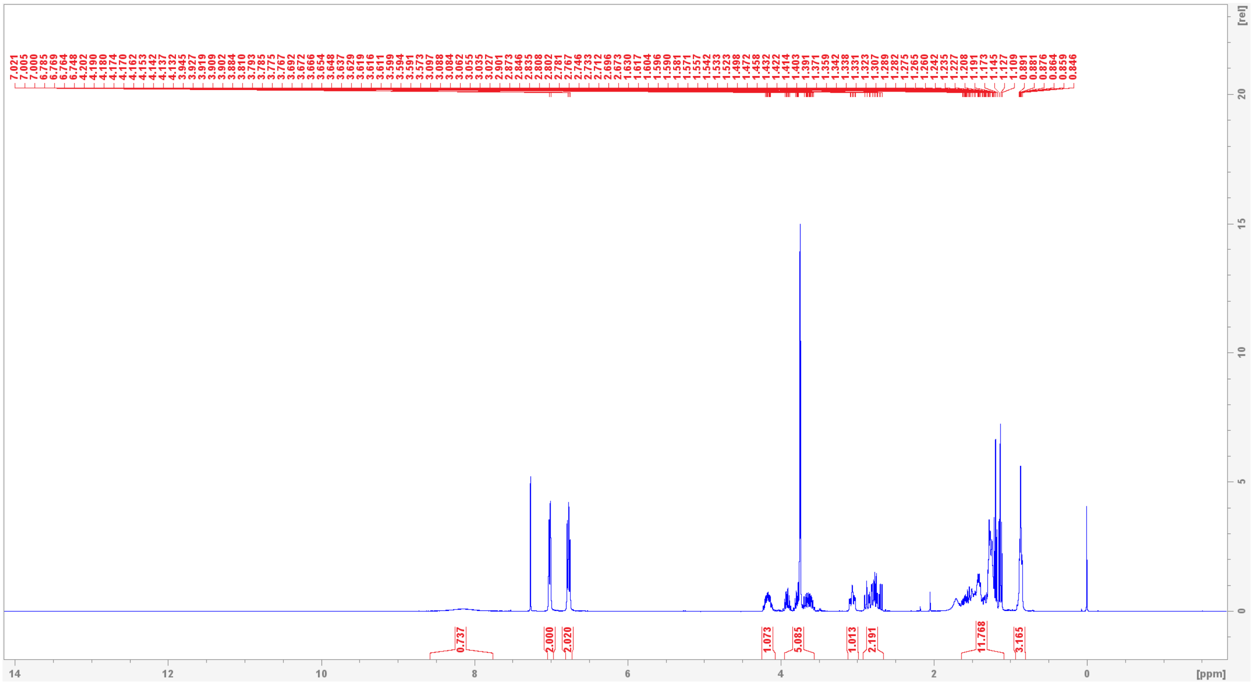

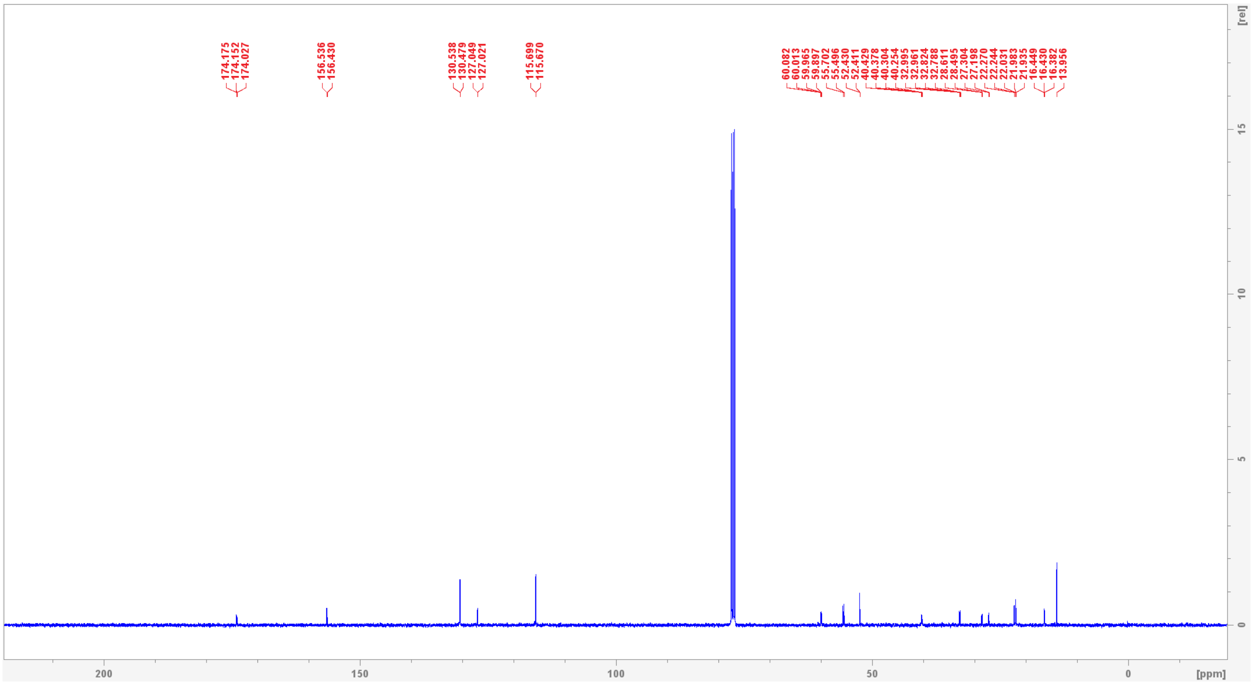
174.175
174.152
174.027
156.536
156.430
130.538
130.479
127.049
127.021
115.699
115.670
60.082
60.013
59.965
59.897
55.702
55.496
52.430
52.411
40.429
40.378
40.304
40.254
32.995
32.961
32.824
32.788
28.611
28.495
27.304
27.198
22.270
22.244
22.031
21.983
21.935
16.449
16.430
16.382
13.956
[rel]
15
10
5
0
200
150
100
50
0
[ppm]

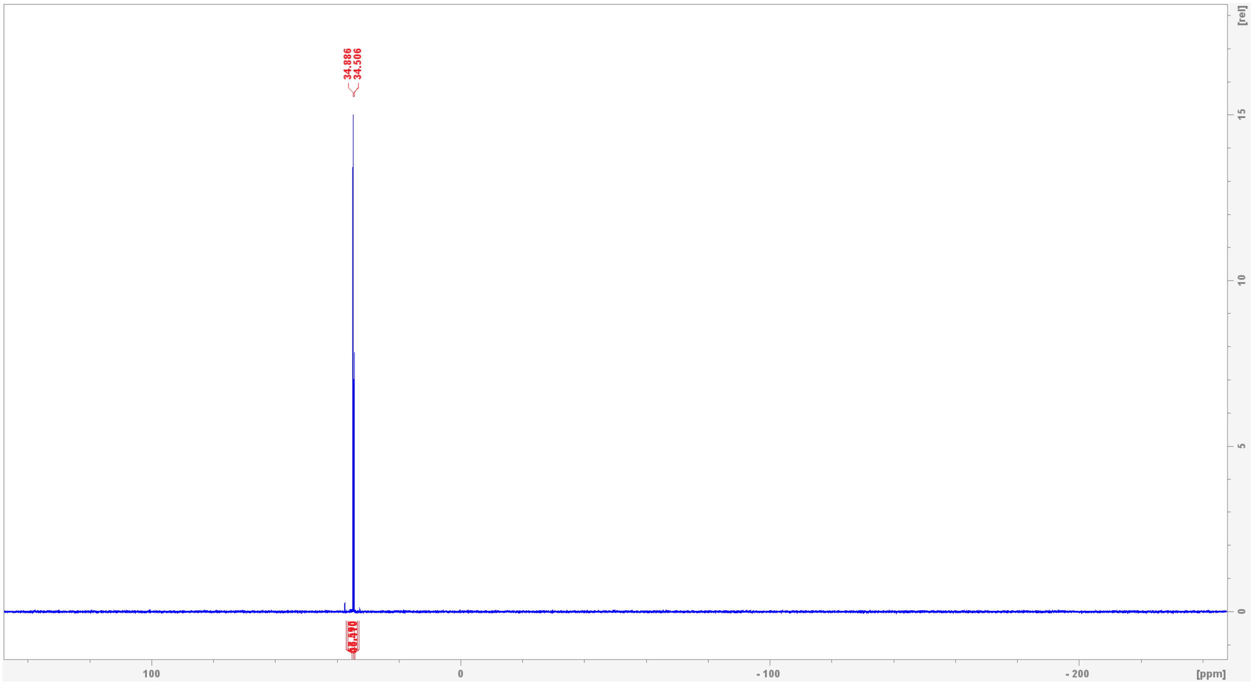
34.886
34.506
[rel]
15
10
5
0
100
0
- 100
- 200
[ppm]

Compound **4j**: ^{1}**H NMR** (400 MHz, $CDCl_3$), ^{13}**C NMR** (100.6 MHz, $CDCl_3$) and ^{31}**P NMR** (161.9 MHz, $CDCl_3$).

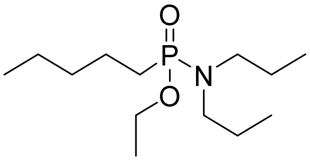

4j

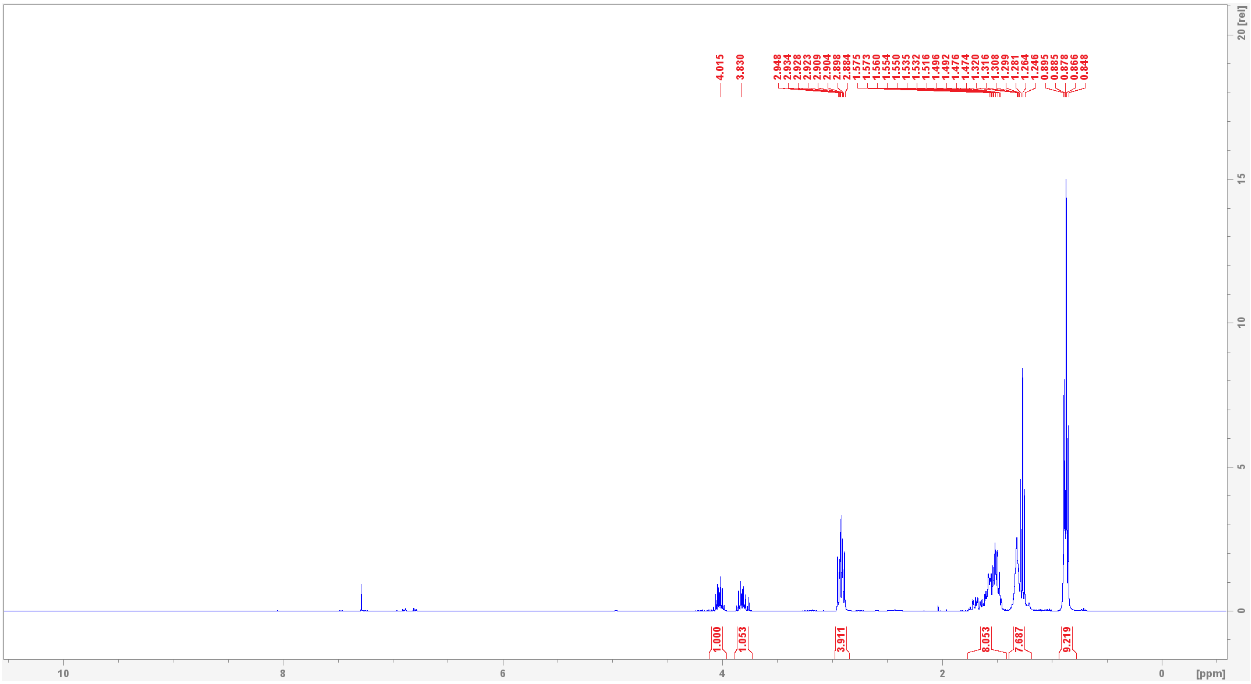

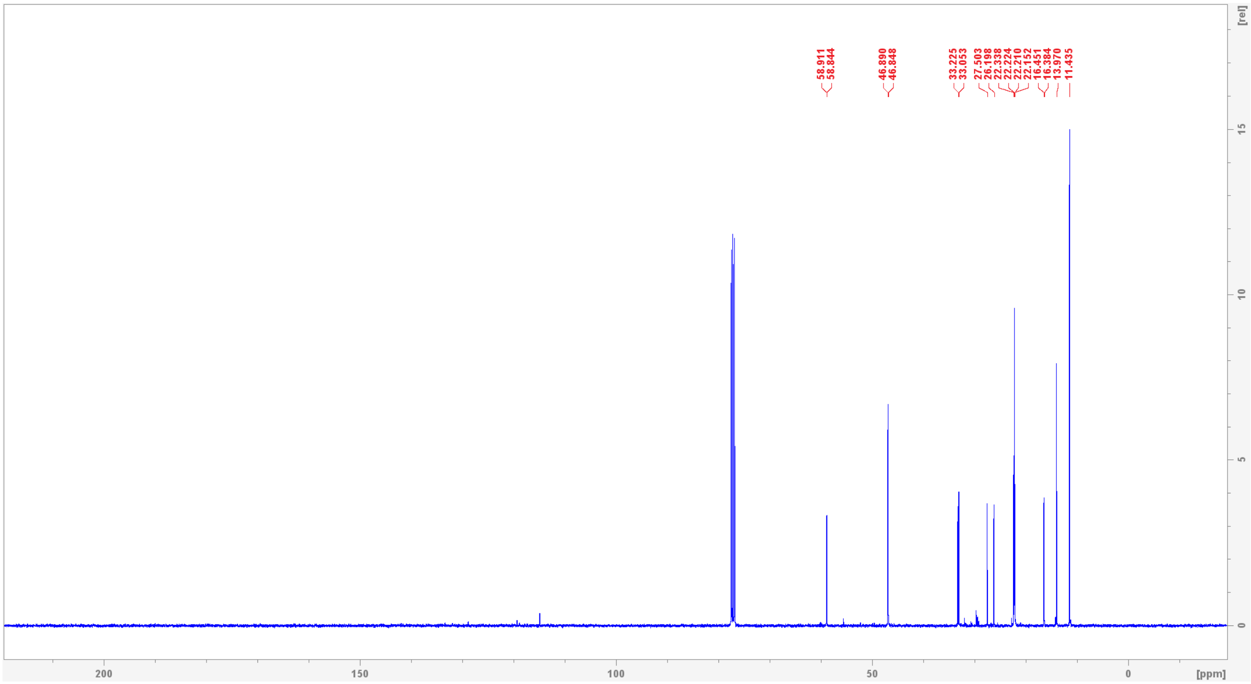

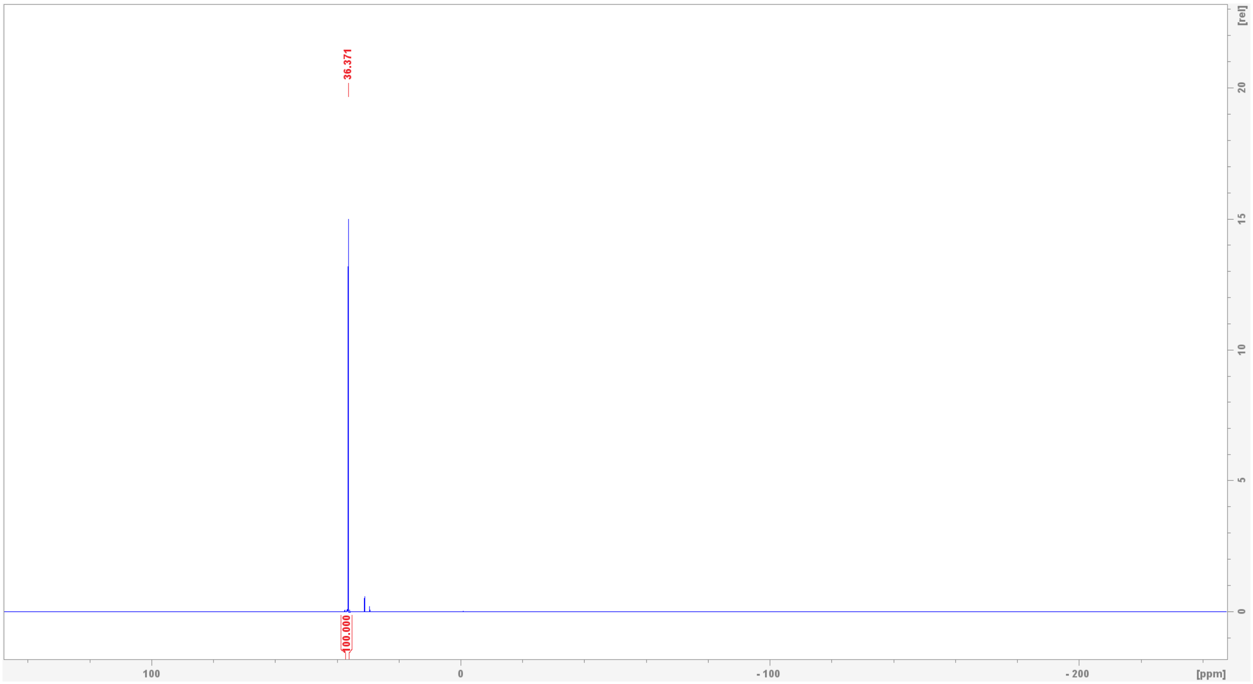
36.371
100.000
100
0
- 100
- 200
[ppm]
[rel]
20
15
10
5
0

Compound **4k**: **^{1}H NMR** (400 MHz, $CDCl_3$), **^{13}C NMR** (100.6 MHz, $CDCl_3$) and **^{31}P NMR** (161.9 MHz, $CDCl_3$).

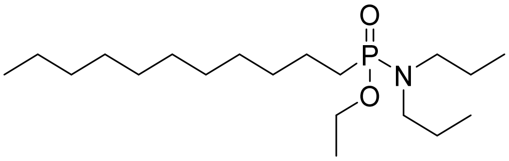

4k

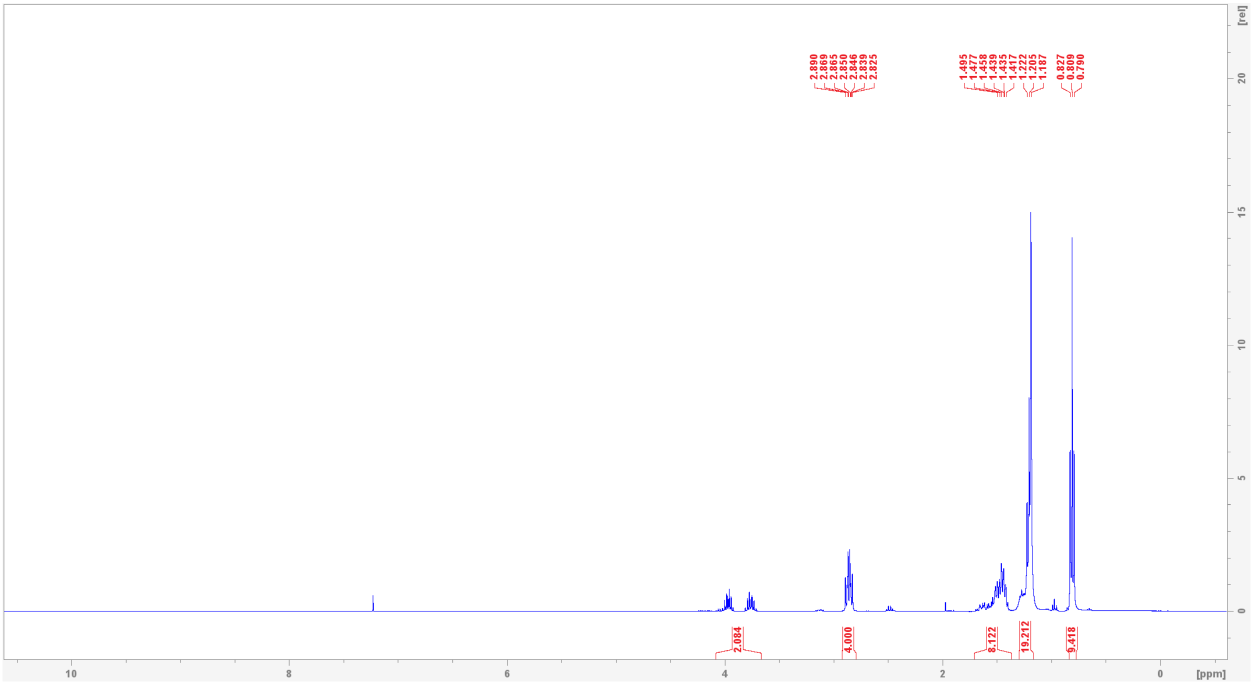

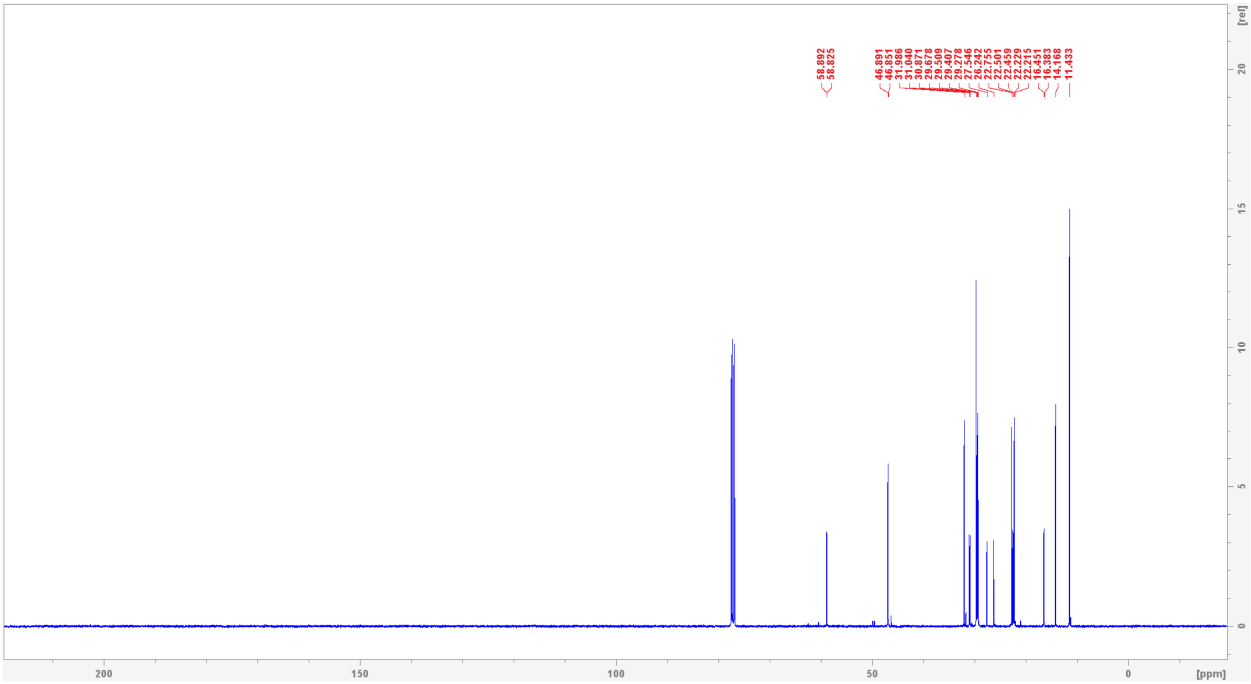

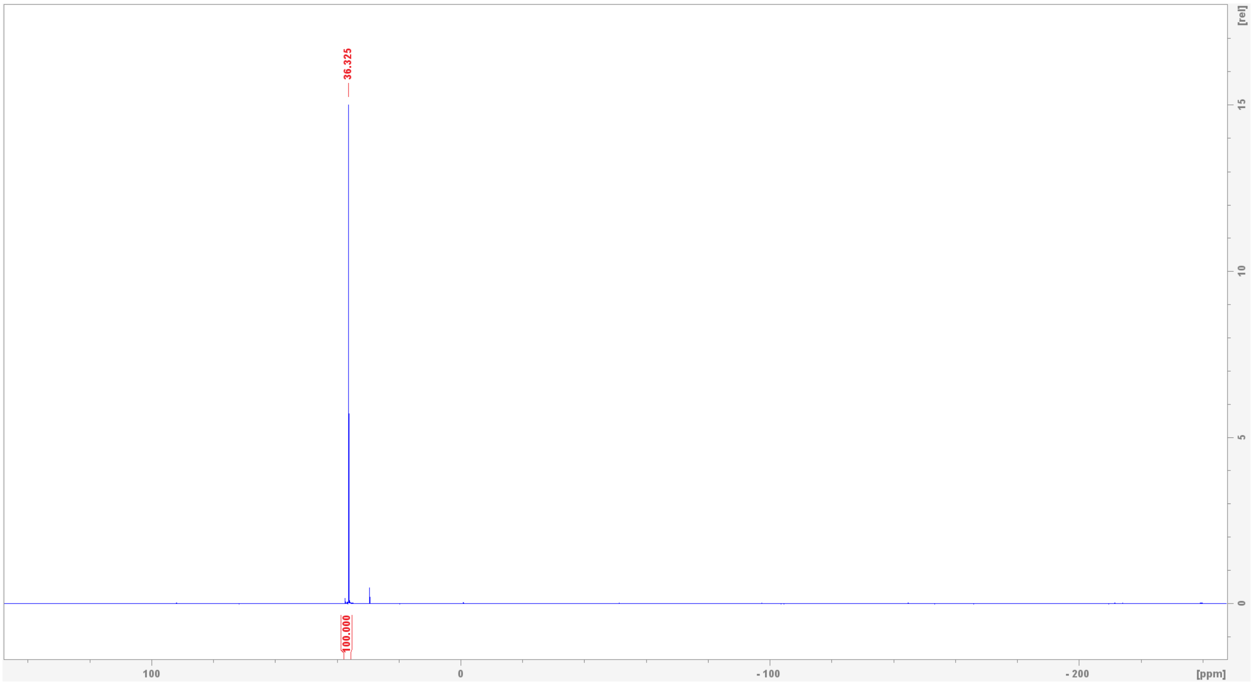

36.325
100.000
[rel]
15
10
5
0
100
0
- 100
- 200
[ppm]

Compound **6a**: ^{1}**H NMR** (400 MHz, $CDCl_3$), ^{13}**C NMR** (100.6 MHz, $CDCl_3$) and ^{31}**P NMR** (161.9 MHz, $CDCl_3$).

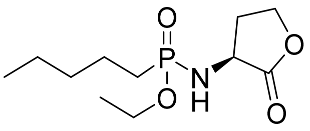

6a

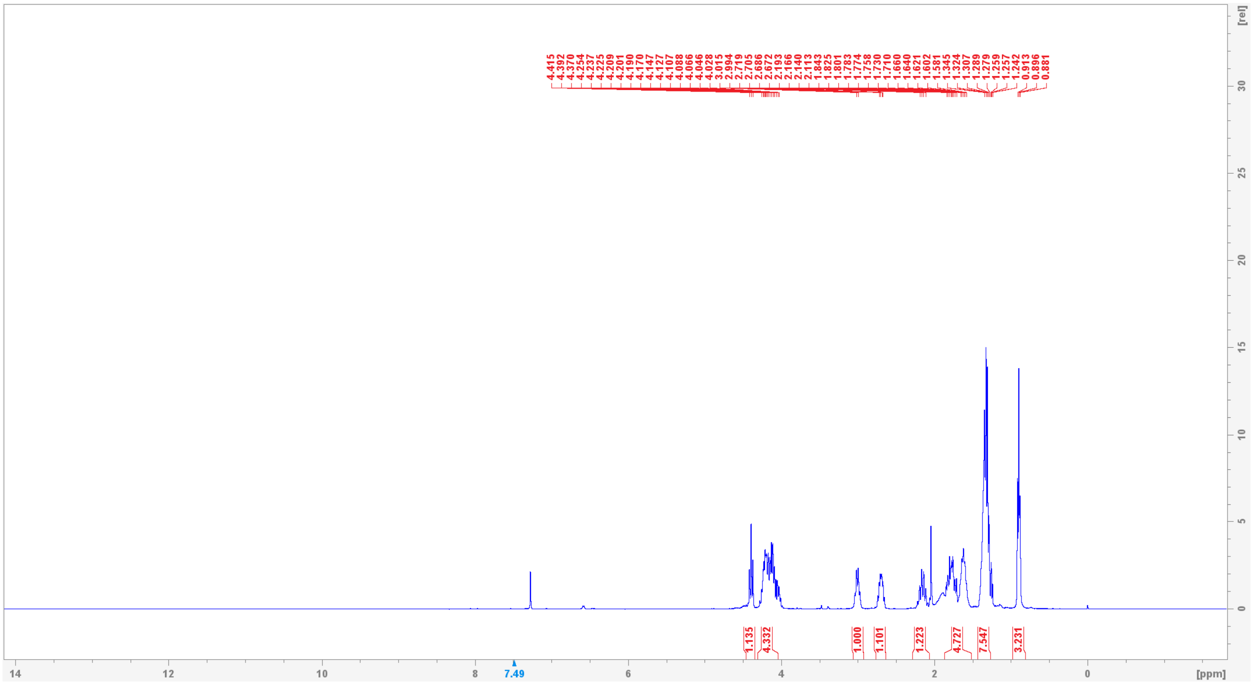

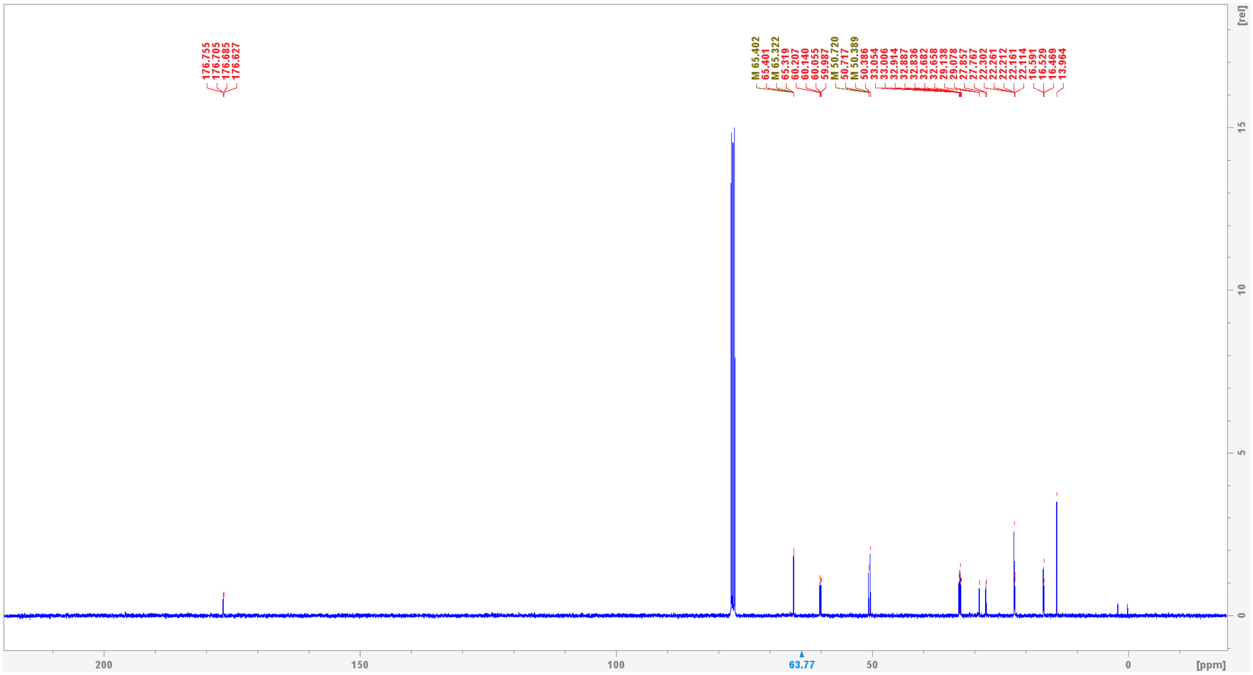

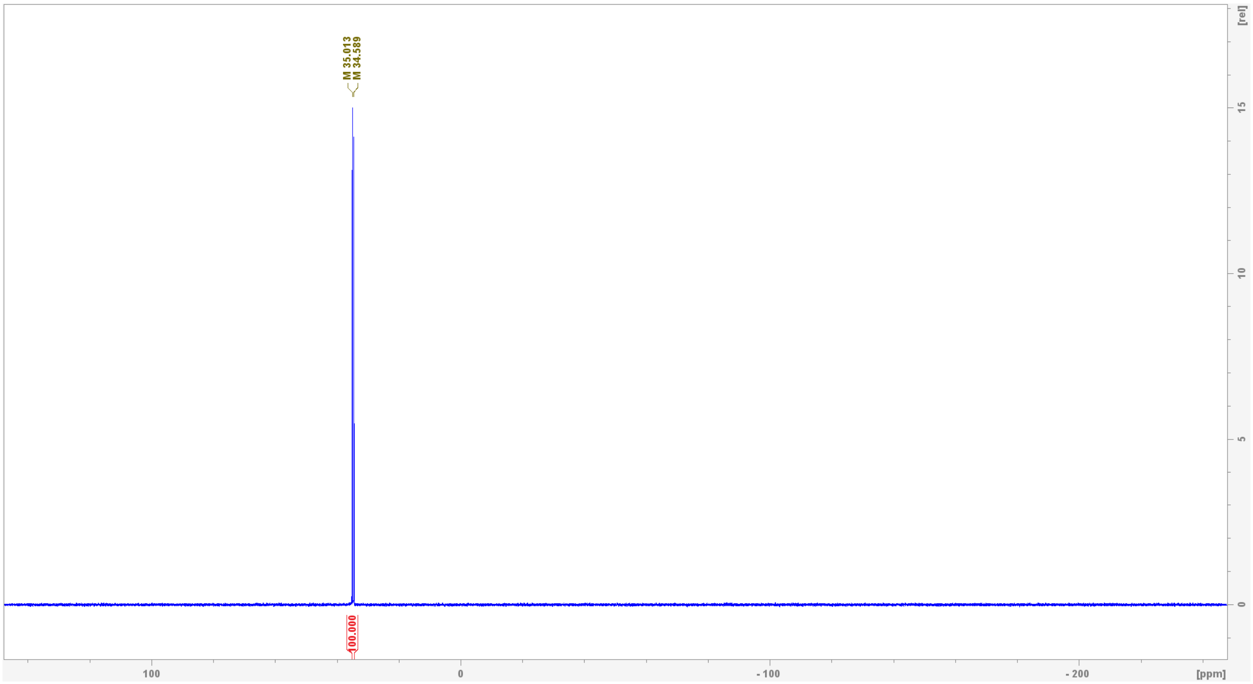

M 35.013
M 34.589
100.000
100
0
- 100
- 200
[ppm]
[rel]
15
10
5
0

Compound **6b**: **^{1}H NMR** (400 MHz, $CDCl_3$), **^{13}C NMR** (100.6 MHz, $CDCl_3$) and **^{31}P NMR** (161.9 MHz, $CDCl_3$).

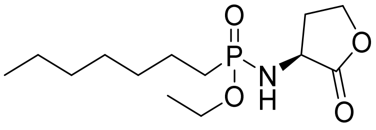

6b

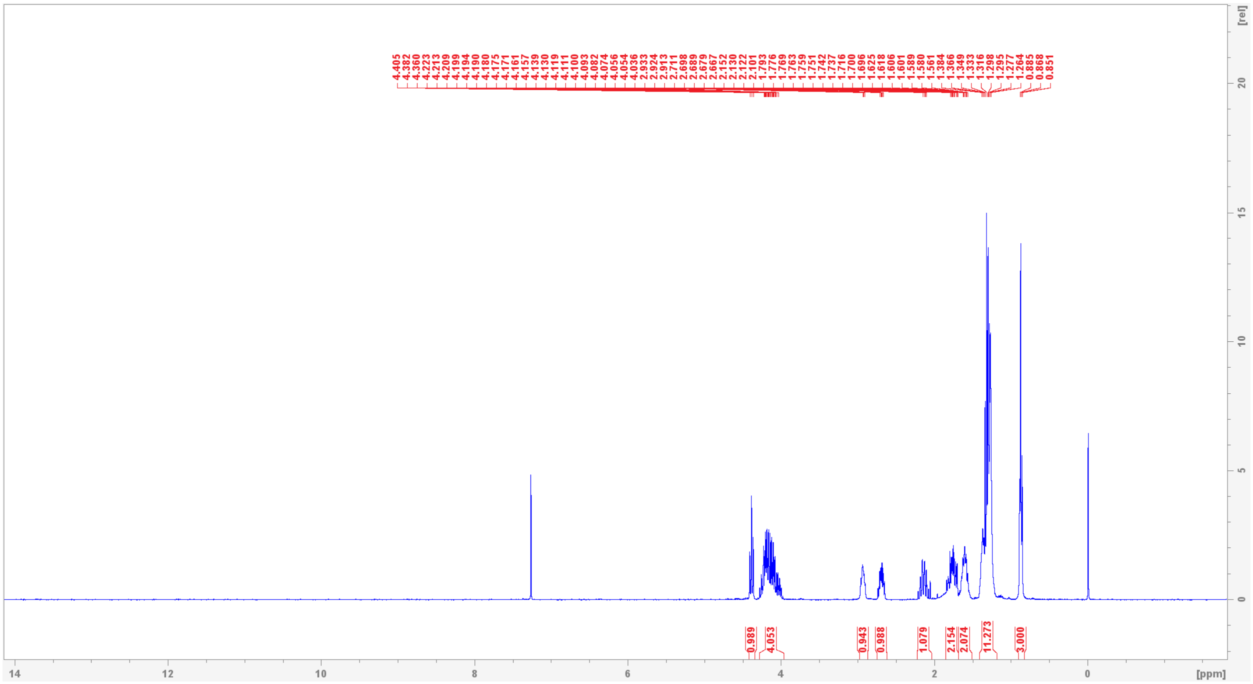

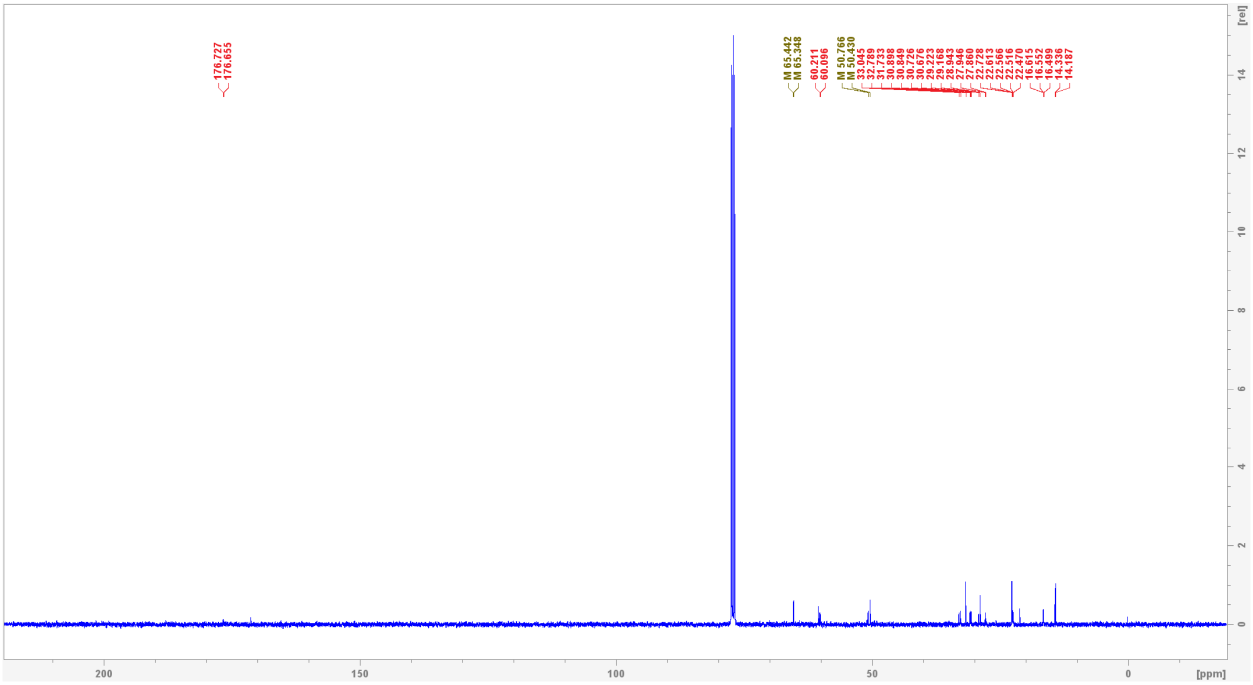

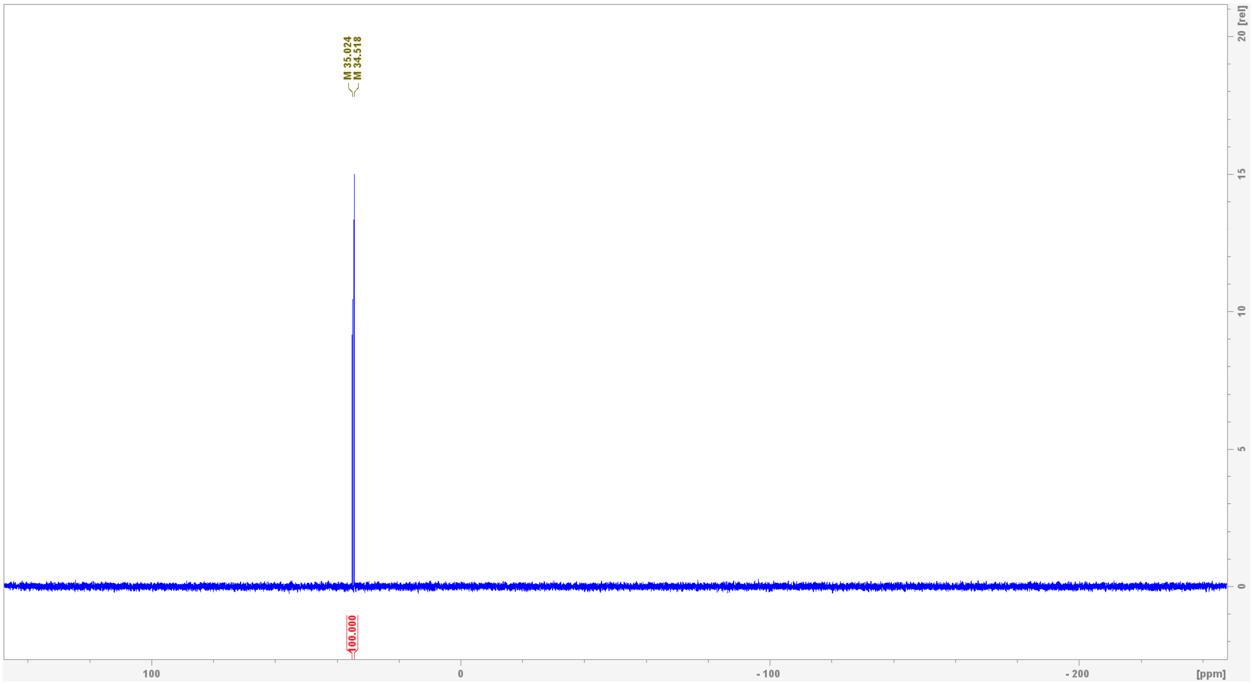

M 35.024
M 34.518
100.000
100
0
- 100
- 200
[ppm]
20 [rel]
15
10
5
0

Compound **6c**: **^{1}H NMR** (400 MHz, $CDCl_3$), **^{13}C NMR** (100.6 MHz, $CDCl_3$) and **^{31}P NMR** (161.9 MHz, $CDCl_3$).

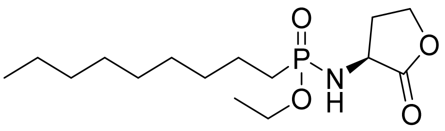

6c

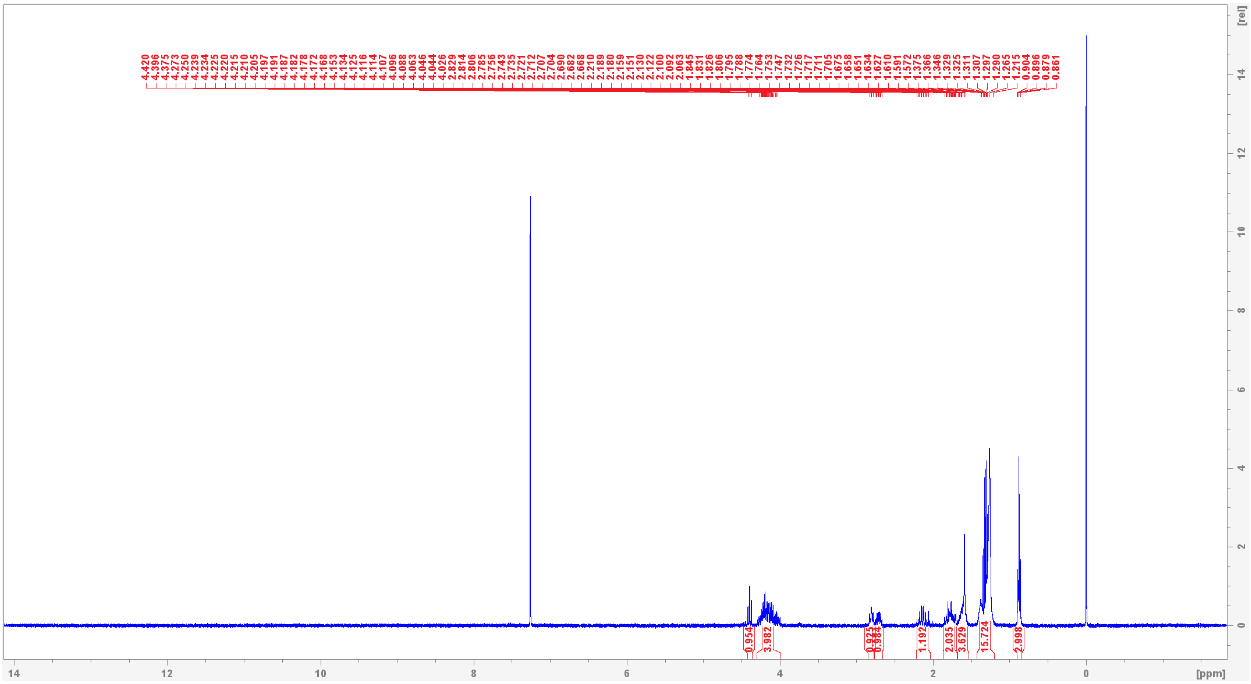

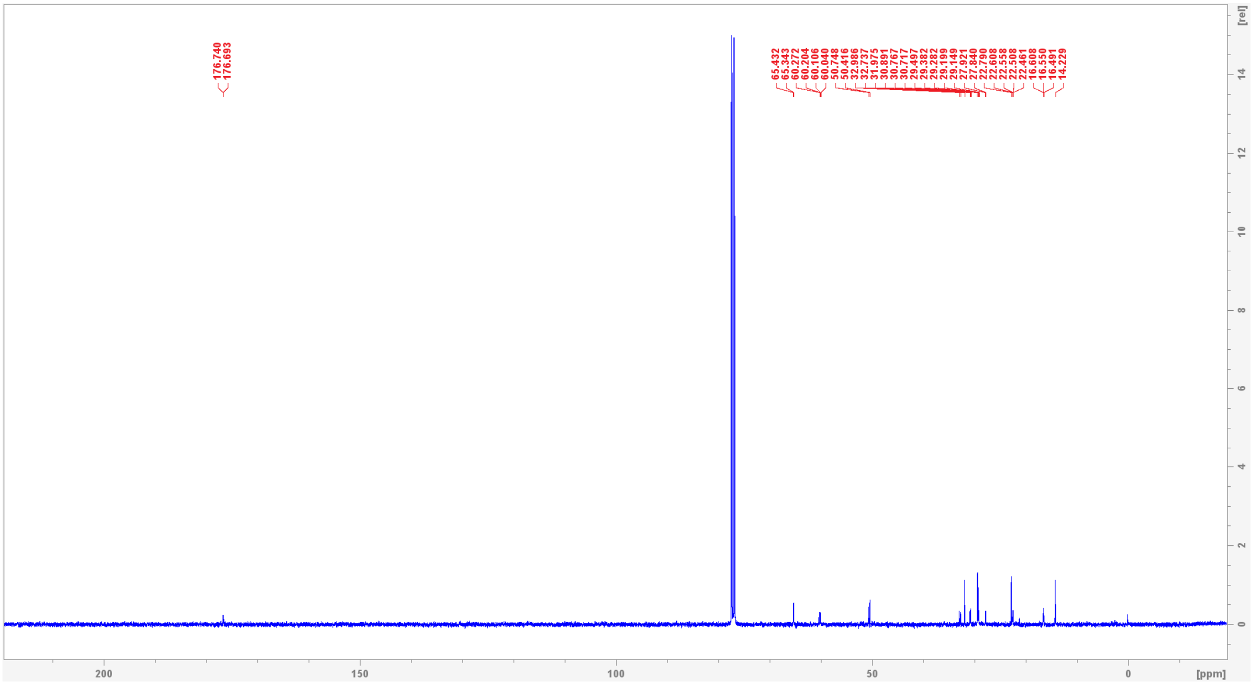

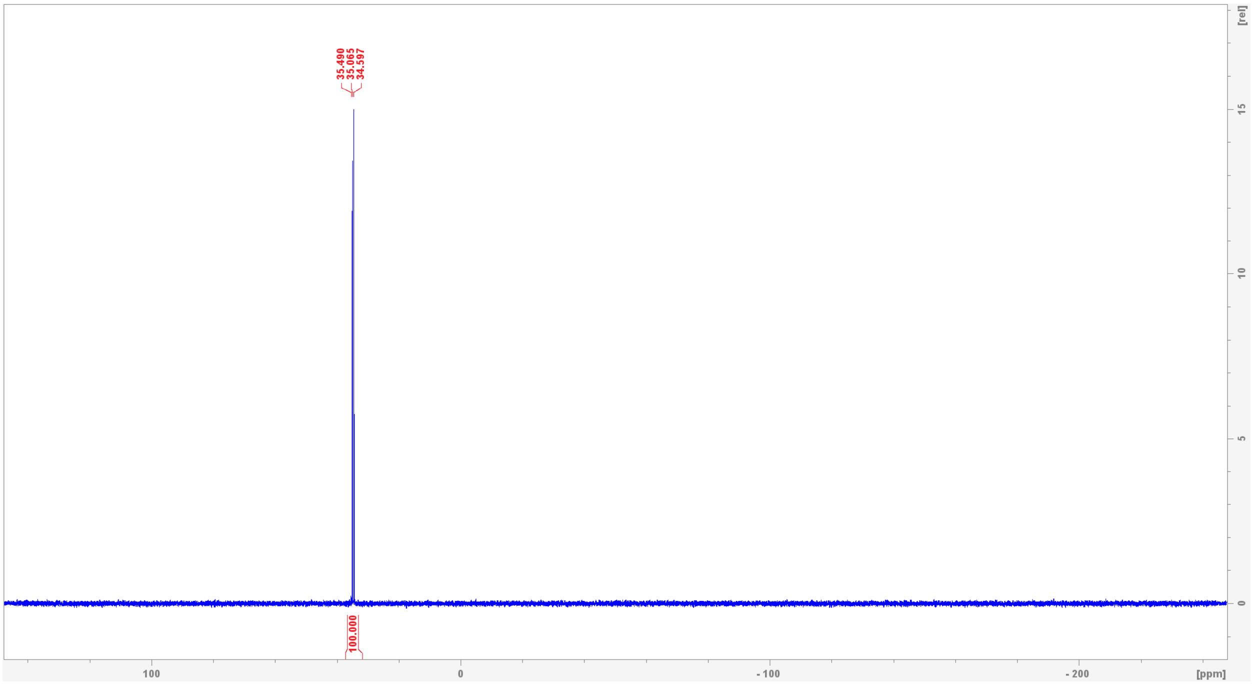

35.490
35.065
34.597
100.000
100
0
- 100
- 200
[ppm]
[rel]
15
10
5
0

Compound **6d**: **^{1}H NMR** (400 MHz, $CDCl_3$), **^{13}C NMR** (100.6 MHz, $CDCl_3$) and **^{31}P NMR** (161.9 MHz, $CDCl_3$).

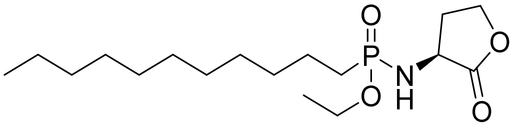

6d

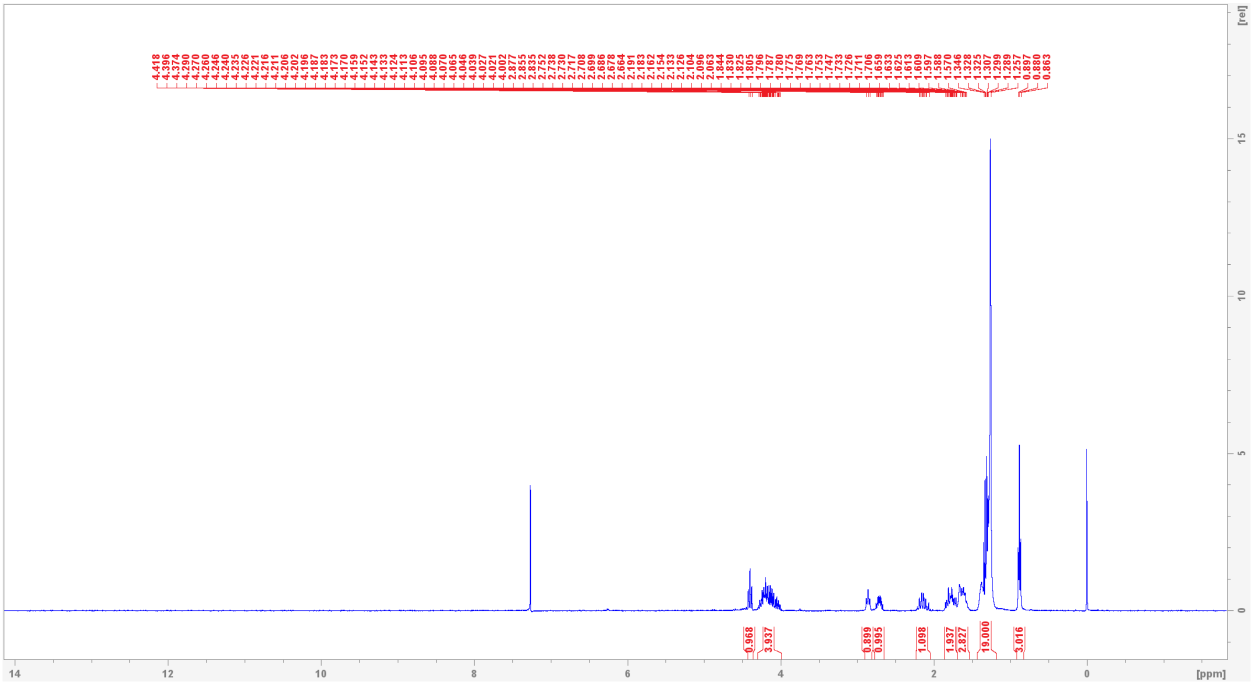

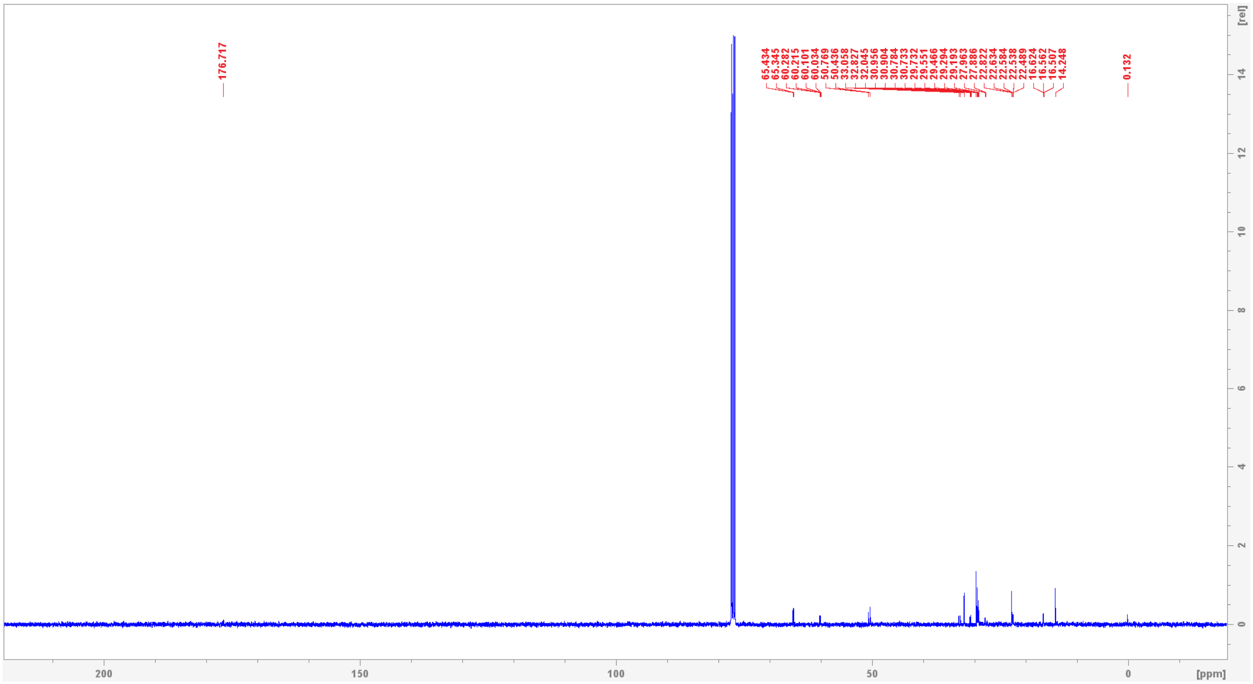

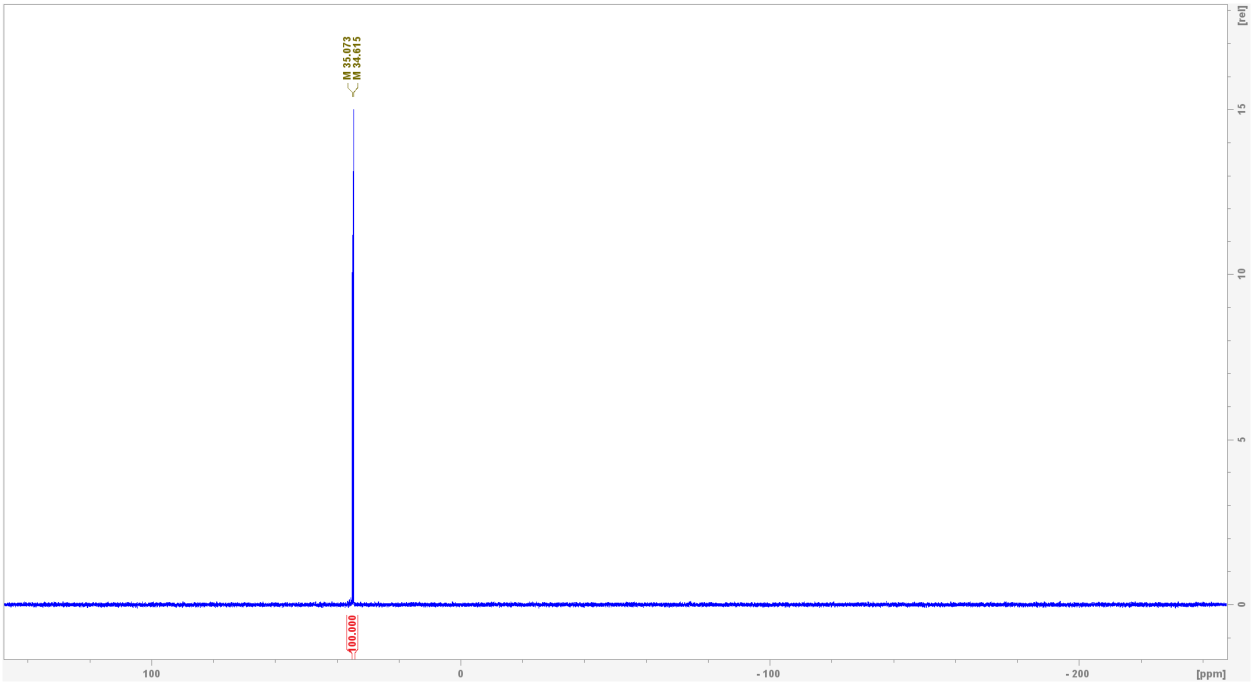

M 35.073
M 34.615
100.000
100
0
- 100
- 200
[ppm]
[rel]
15
10
5
0

Compound **7a**: **^{1}H NMR** (400 MHz, $CDCl_3$), **^{13}C NMR** (100.6 MHz, $CDCl_3$) and **^{31}P NMR** (161.9 MHz, $CDCl_3$).

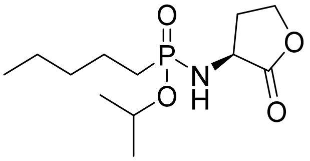

7a

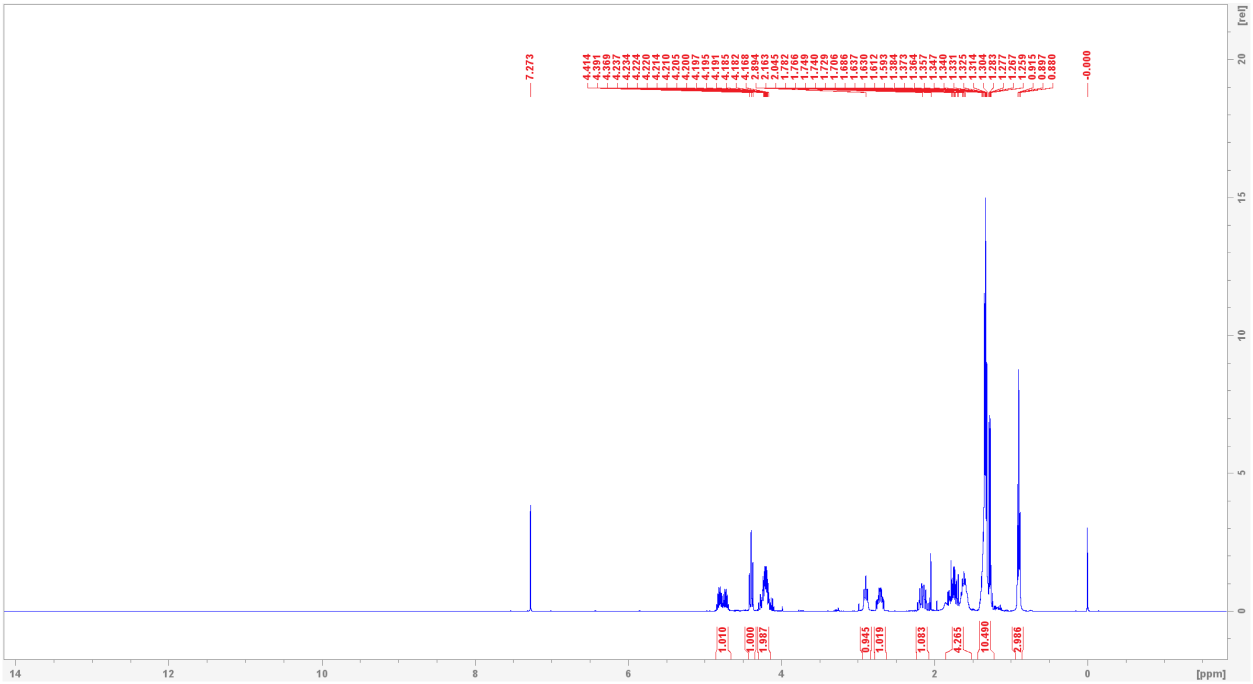

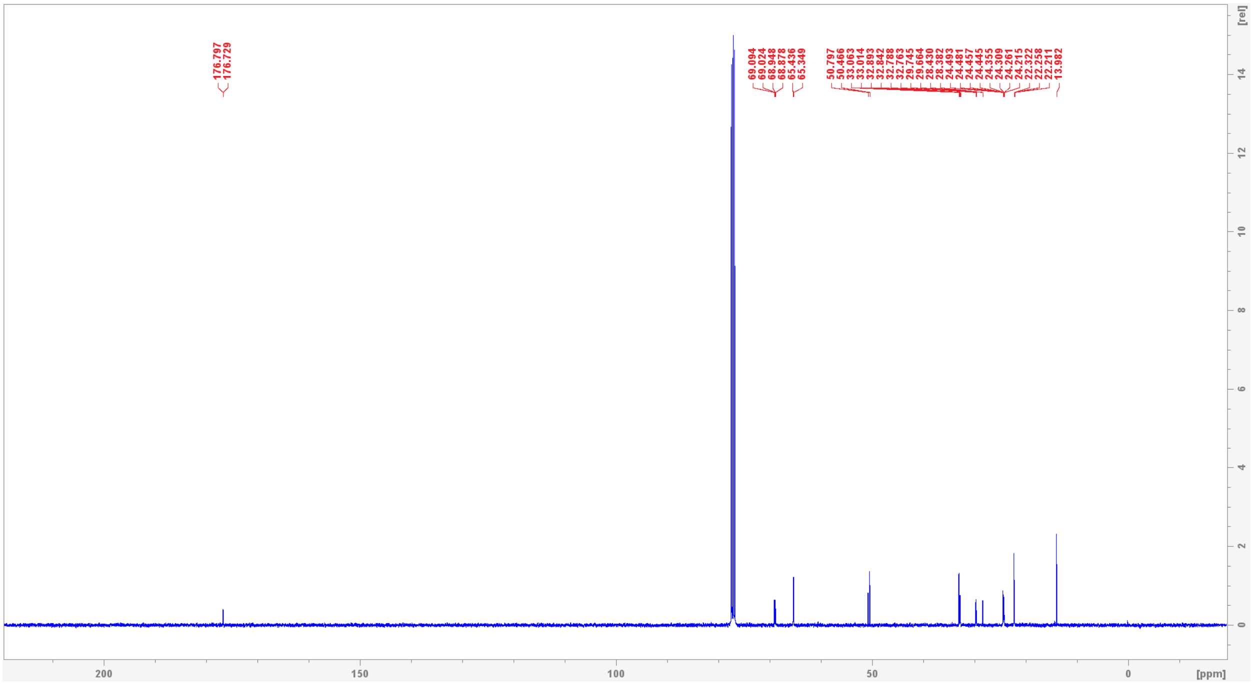

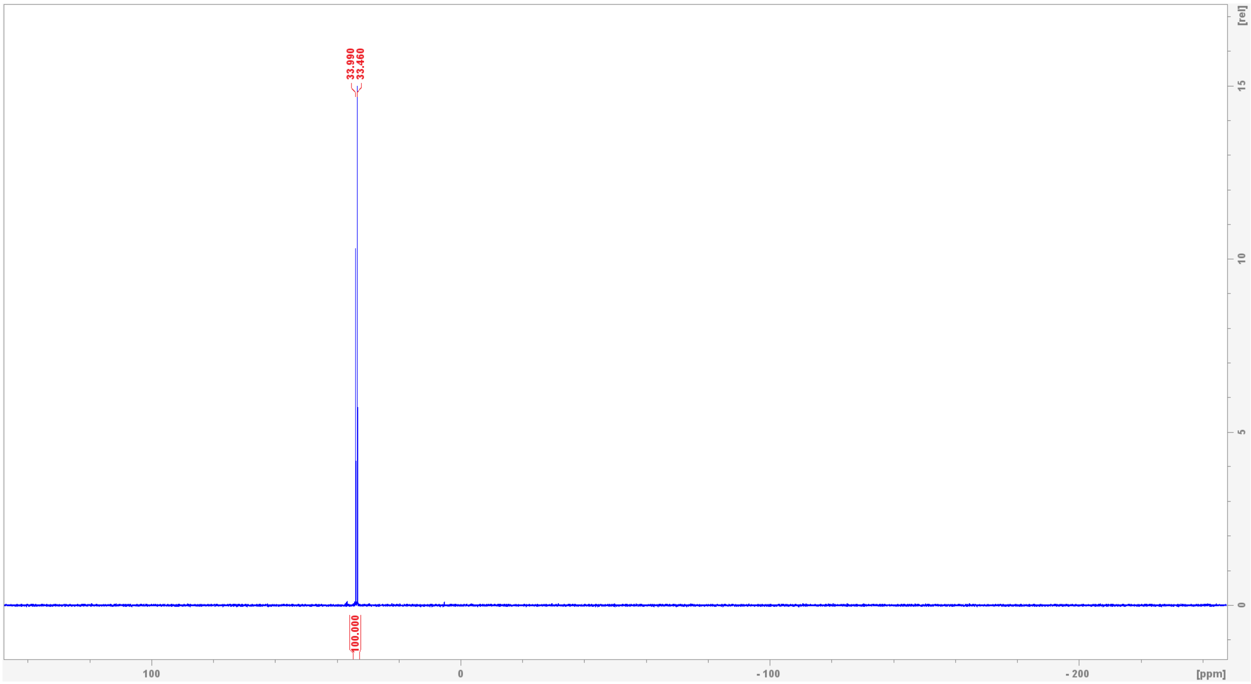

33.990
33.460
100.000
[rel]
15
10
5
0
100
0
- 100
- 200
[ppm]

Compound **7b**: **^{1}H NMR** (400 MHz, $CDCl_3$), **^{13}C NMR** (100.6 MHz, $CDCl_3$) and **^{31}P NMR** (161.9 MHz, $CDCl_3$).

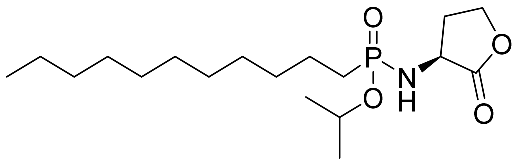

7b

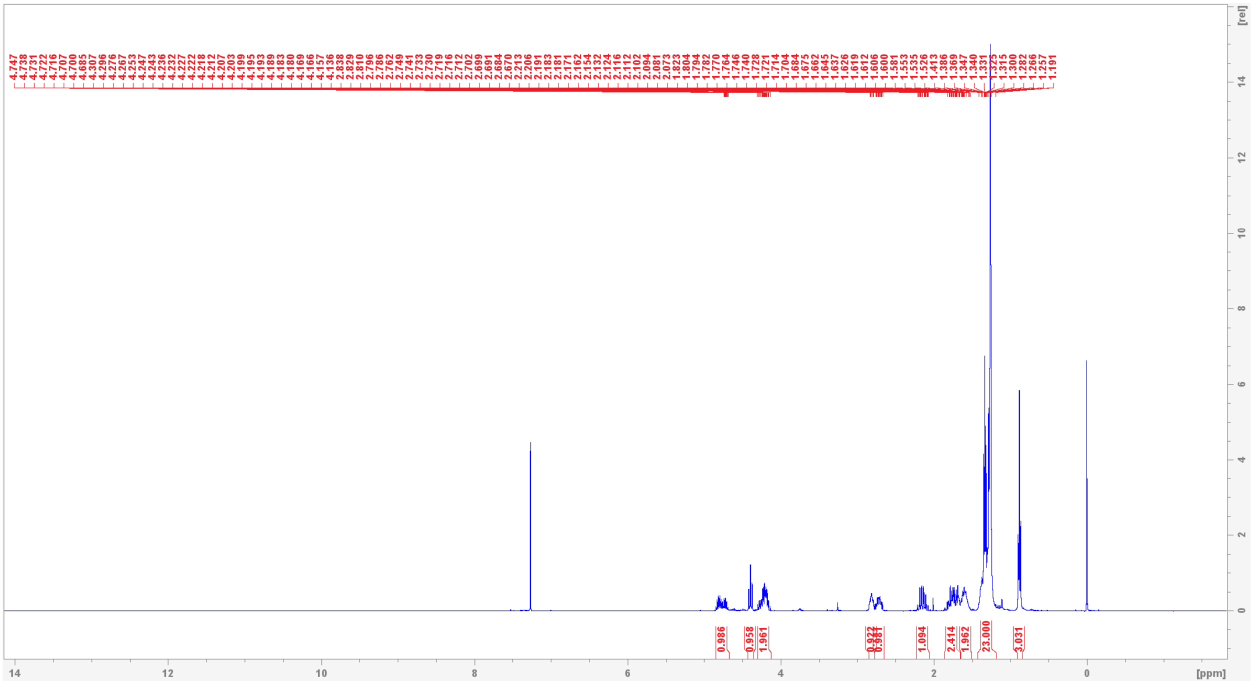

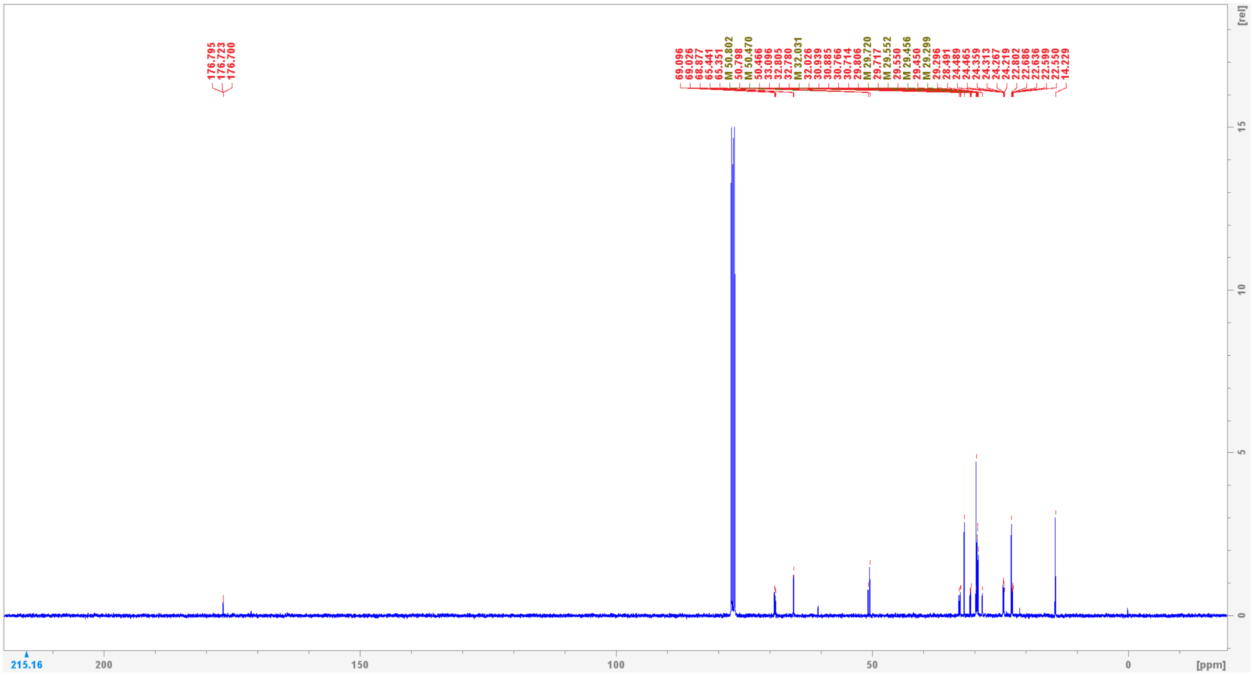

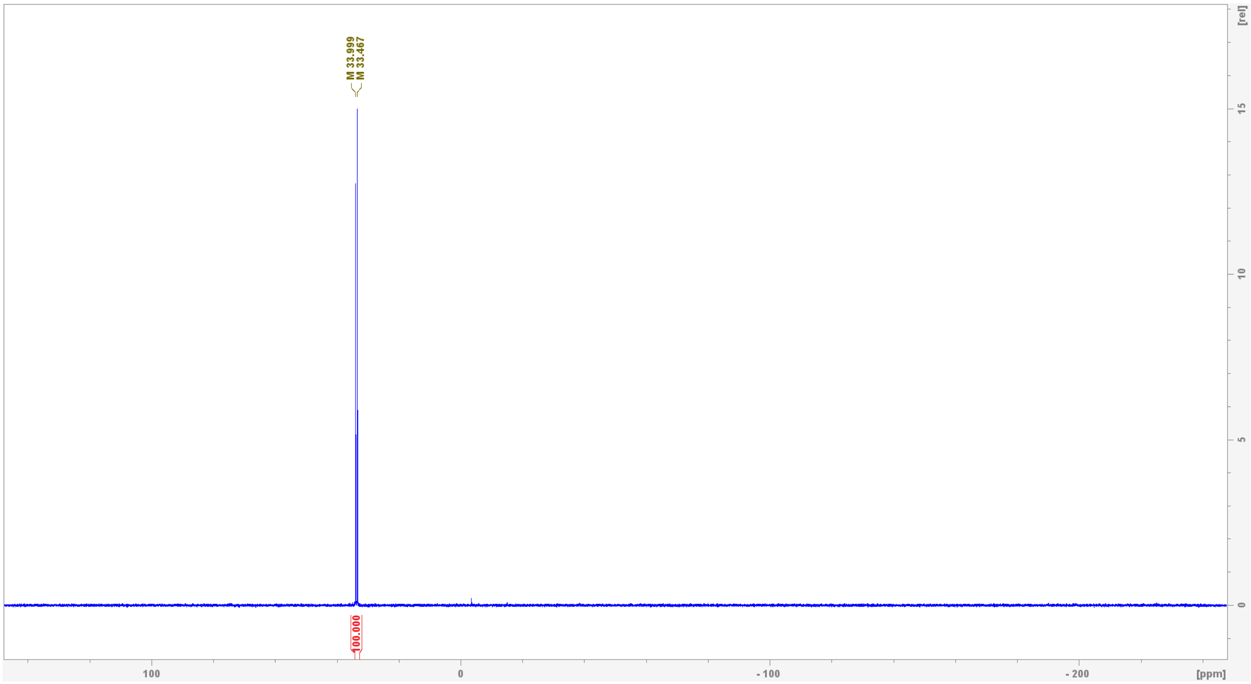

M 33.999
M 33.467
100.000
[rel]
15
10
5
0
100
0
- 100
- 200
[ppm]

Compound **8a**: **^{1}H NMR** (400 MHz, $CDCl_3$), **^{13}C NMR** (100.6 MHz, $CDCl_3$) and **^{31}P NMR** (161.9 MHz, $CDCl_3$).

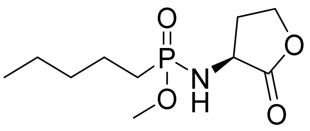

8a

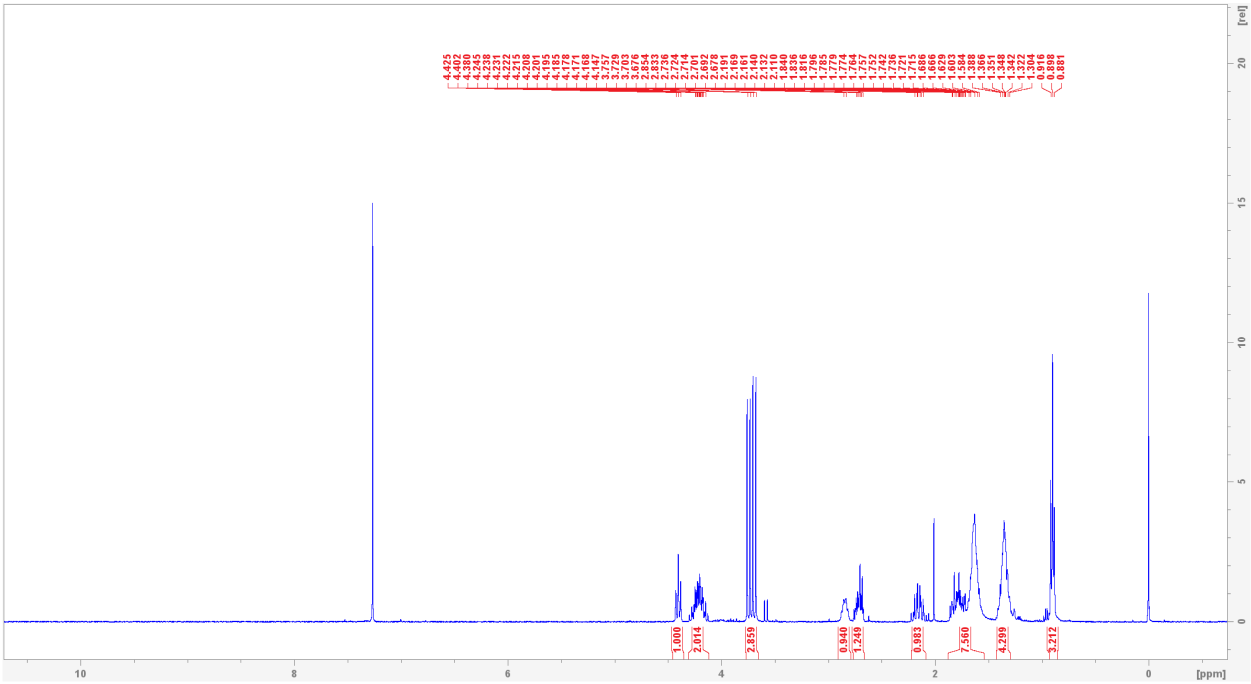

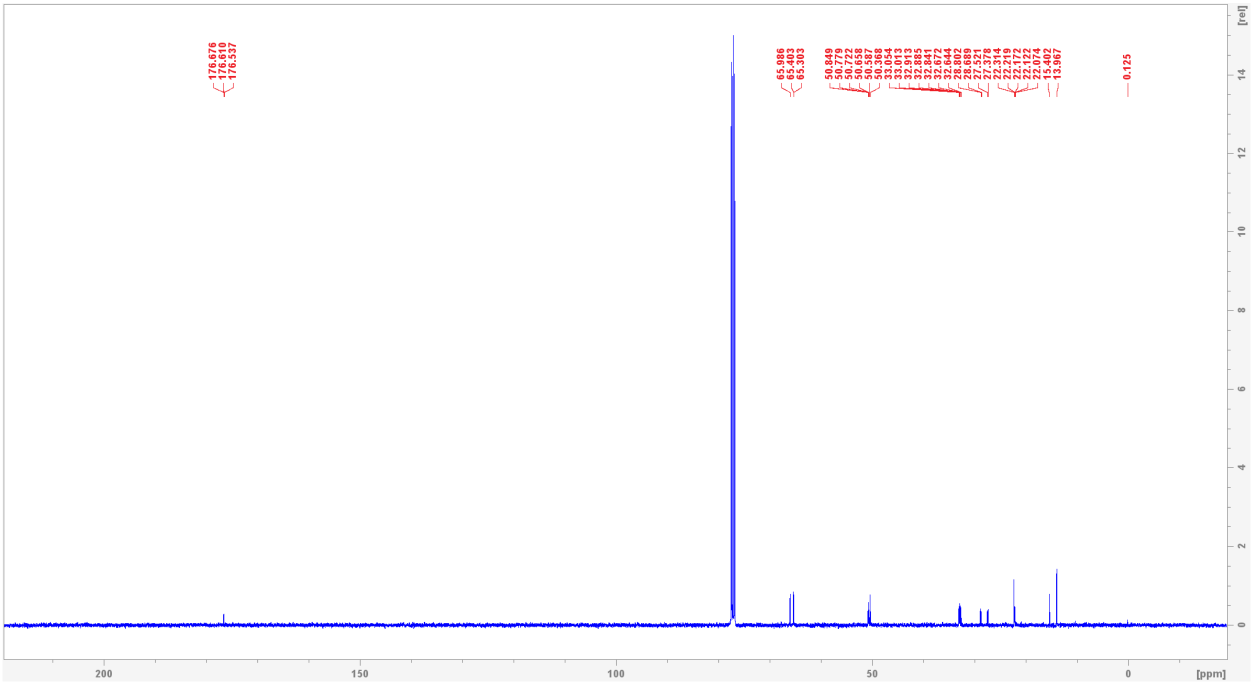

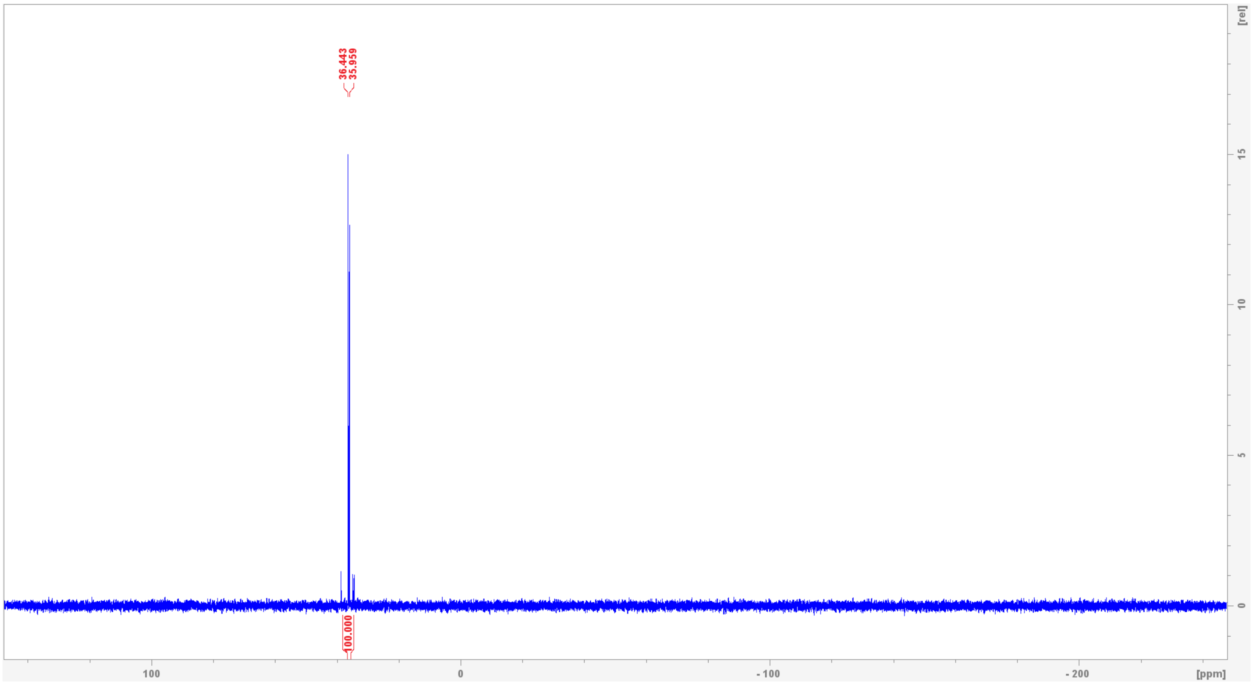

36.443
35.959
100.000
[rel]
15
10
5
0
100
0
- 100
- 200
[ppm]

Compound **8b**: **^{1}H NMR** (400 MHz, $CDCl_3$), **^{13}C NMR** (100.6 MHz, $CDCl_3$) and **^{31}P NMR** (161.9 MHz, $CDCl_3$).

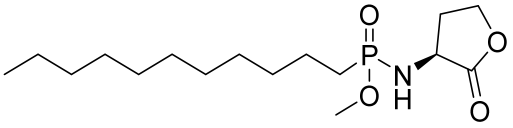

8b

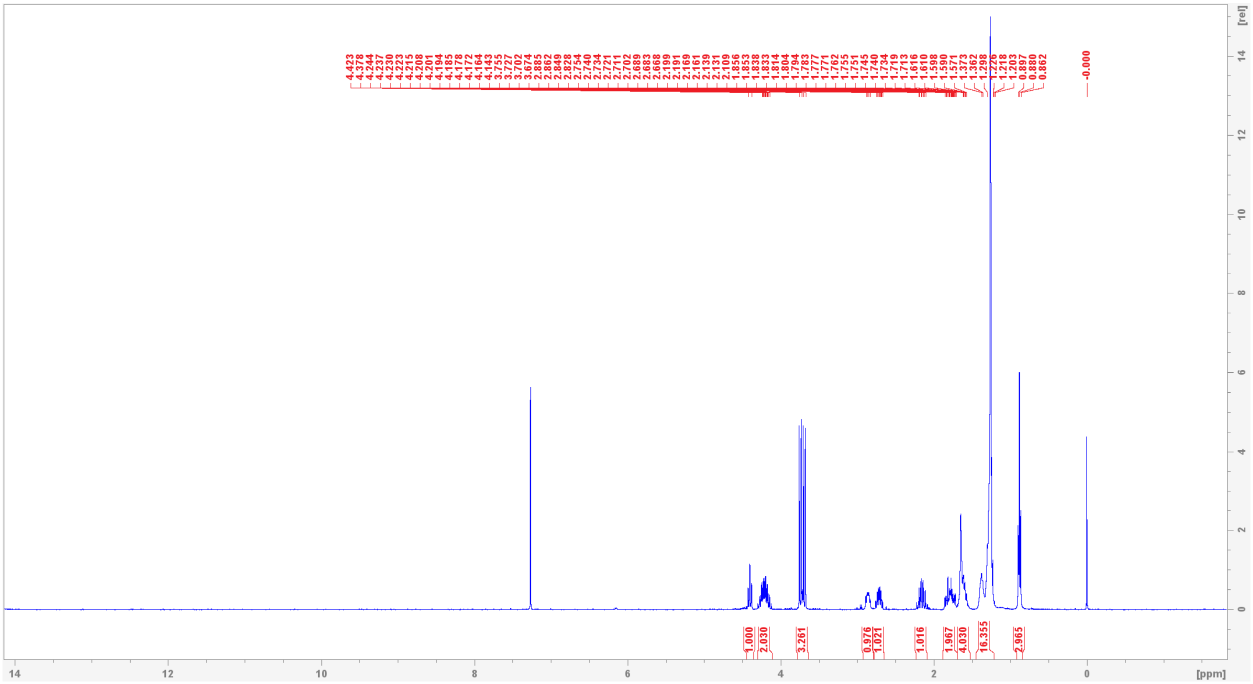

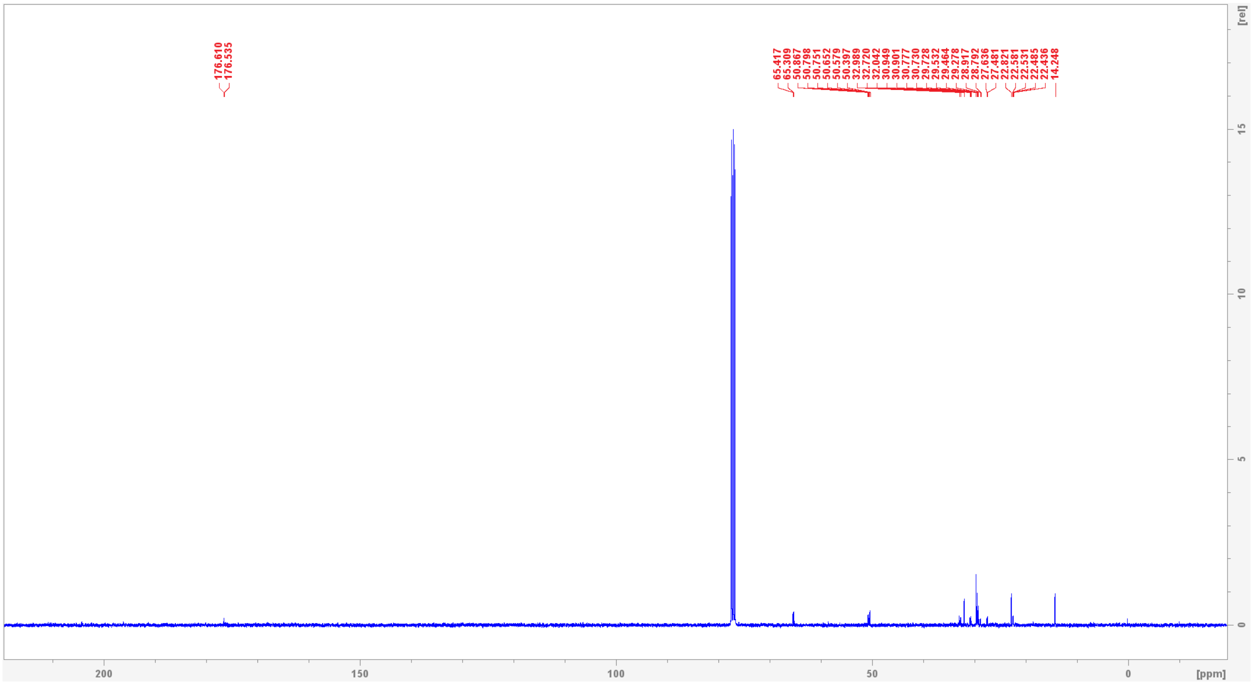

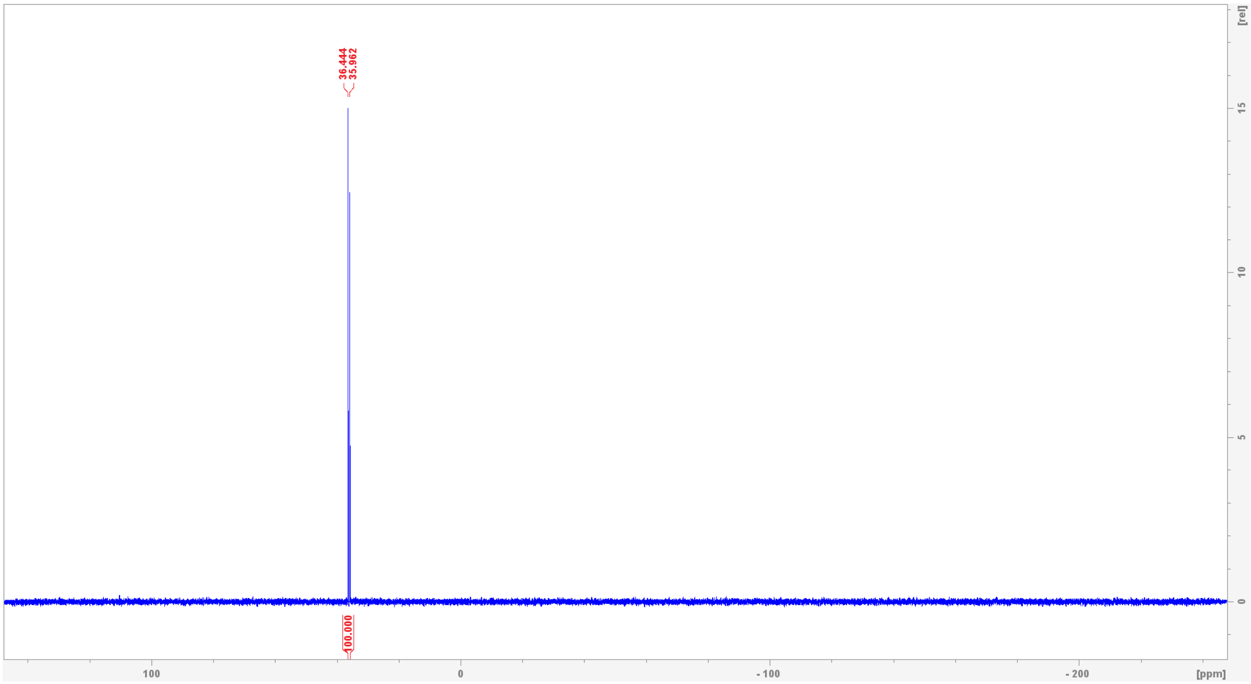

36.444
35.962
100.000
[rel]
15
10
5
0
100
0
- 100
- 200
[ppm]

Compound **9a**: **^{1}H NMR** (400 MHz, $CDCl_3$), **^{13}C NMR** (100.6 MHz, $CDCl_3$) and **^{31}P NMR** (161.9 MHz, $CDCl_3$).

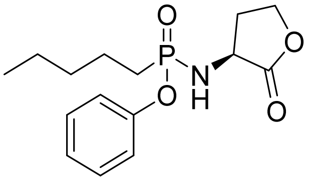

9a

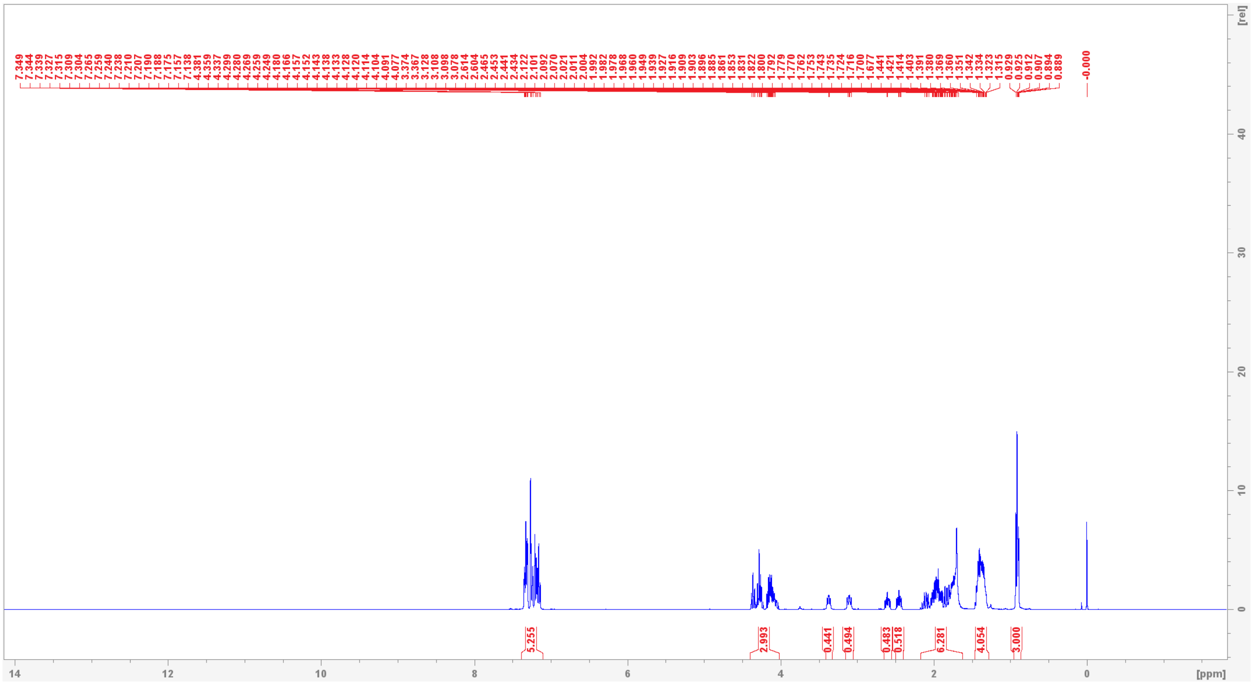

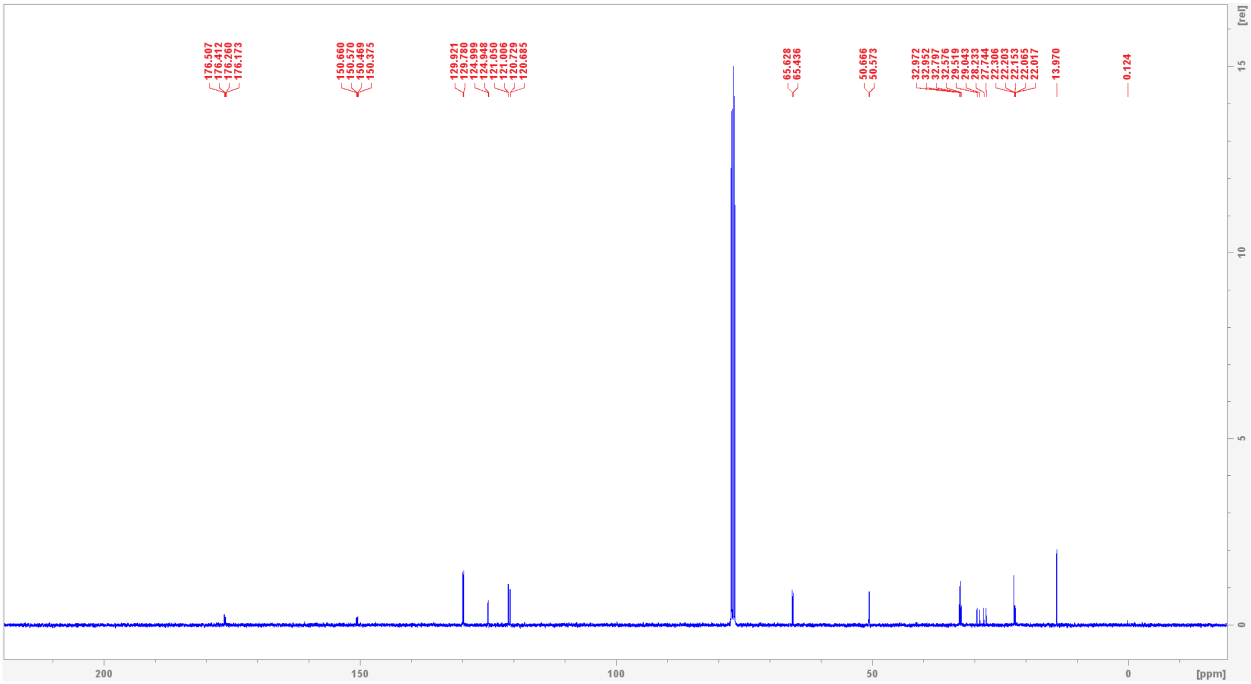

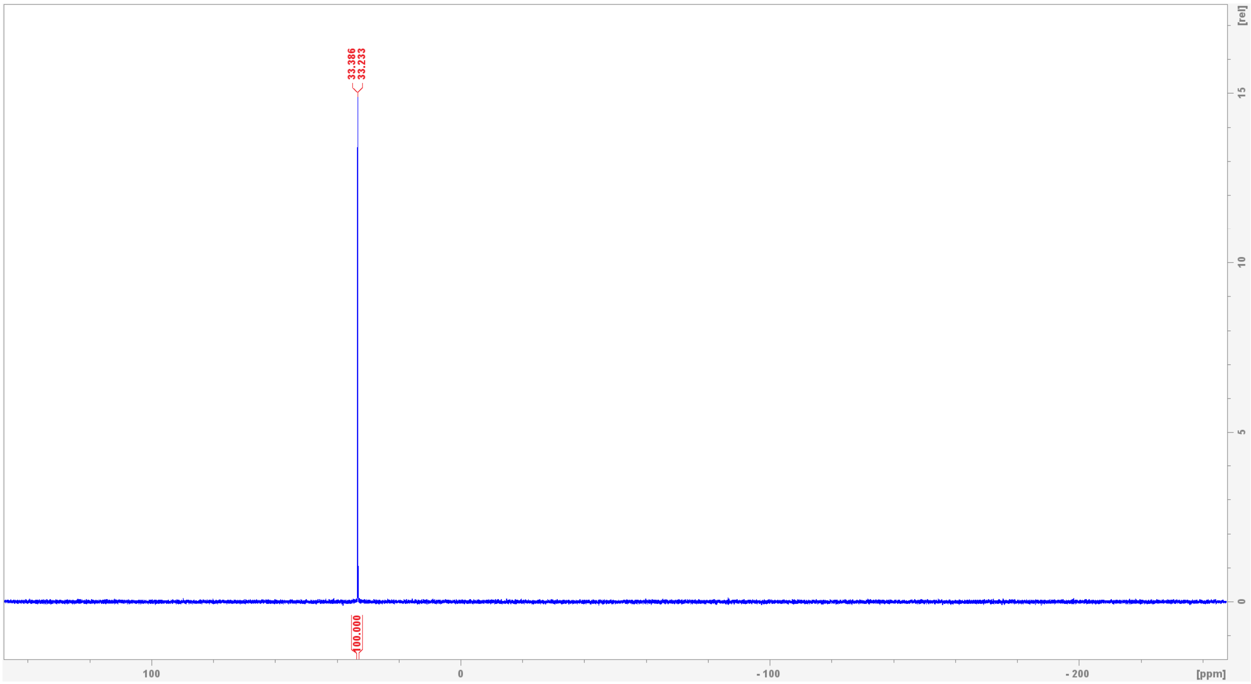

33.386
33.233
100.000
[rel]
15
10
5
0
100
0
- 100
- 200
[ppm]

Compound **9b** **^{1}H NMR** (400 MHz, $CDCl_3$), **^{13}C NMR** (100.6 MHz, $CDCl_3$) and **^{31}P NMR** (161.9 MHz, $CDCl_3$).

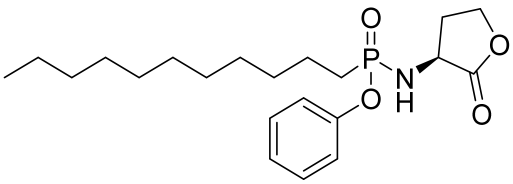

9b

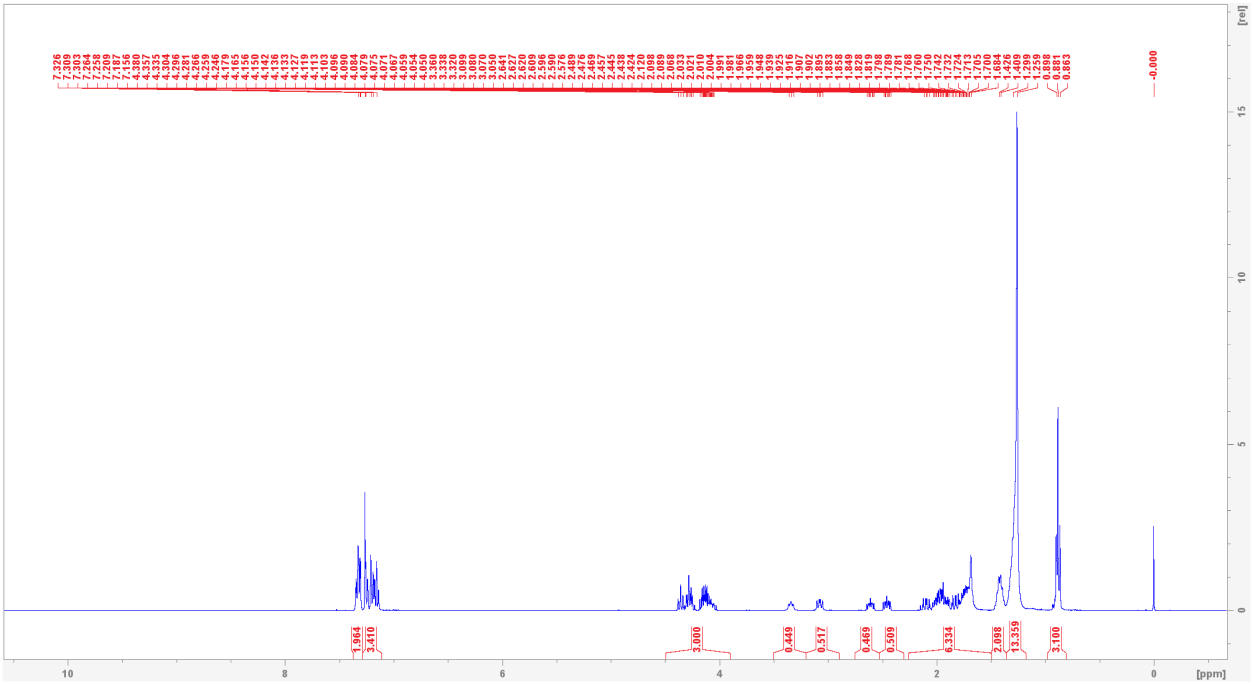

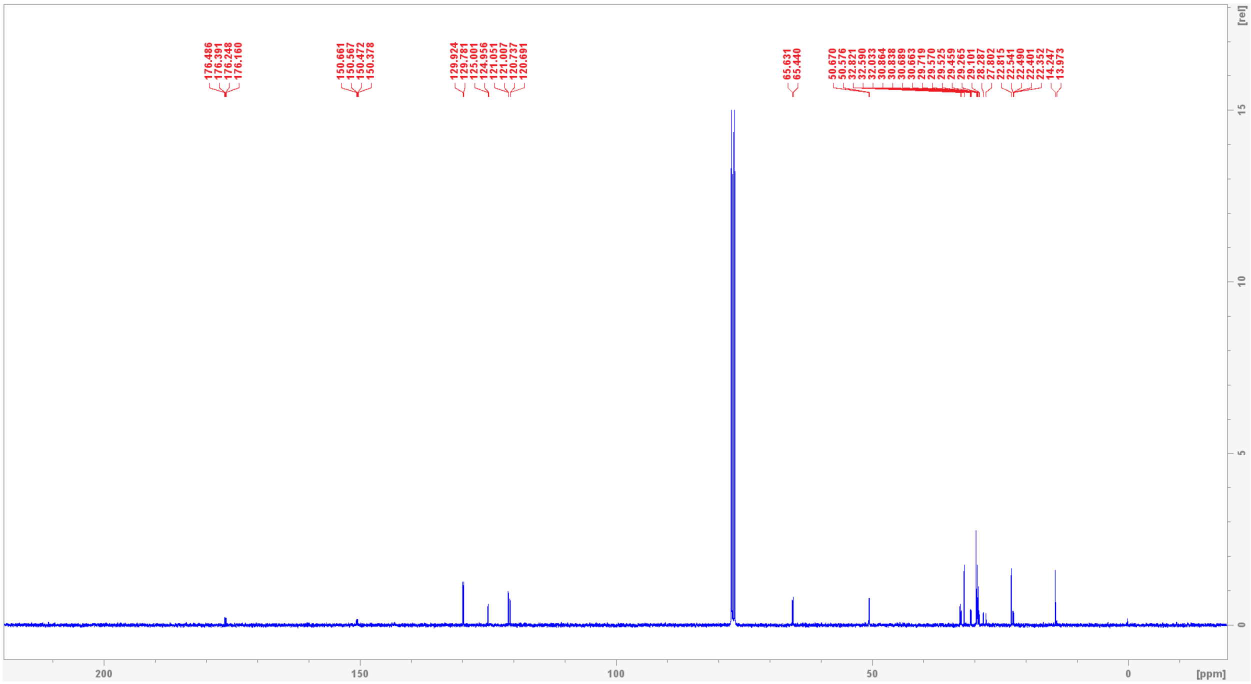
176.486
176.391
176.248
176.160
150.661
150.567
150.472
150.378
129.924
129.781
125.091
124.956
121.051
121.007
120.737
120.691
65.631
65.440
50.670
50.576
32.821
32.590
32.033
30.864
30.838
30.689
30.663
29.719
29.570
29.525
29.459
29.365
29.101
28.287
27.802
22.815
22.541
22.490
22.401
22.352
14.247
13.973
200
150
100
50
0
[ppm]
[rel]
15
10
5
0

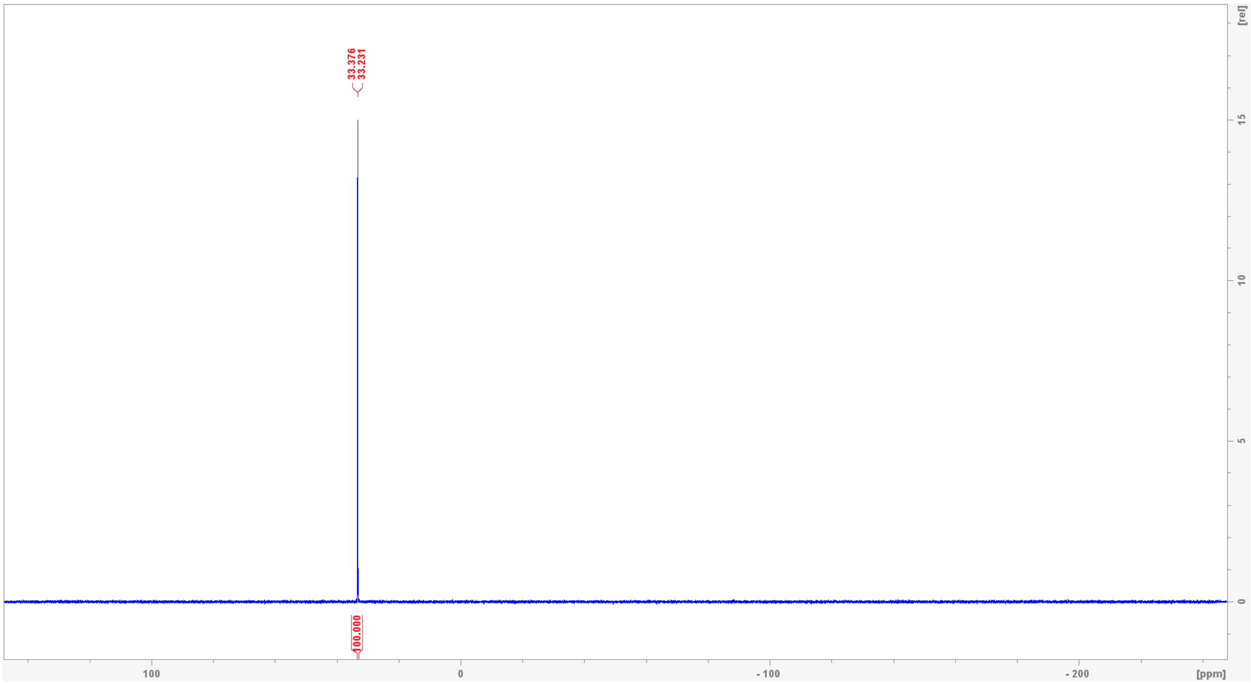
33.376
33.231
100.000
100
0
- 100
- 200
[ppm]
[rel]
15
10
5
0

Compound **15a**: ^{1}**H NMR** (400 MHz, $CDCl_3$), ^{13}**C NMR** (100.6 MHz, $CDCl_3$) and ^{31}**P NMR** (161.9 MHz, $CDCl_3$).

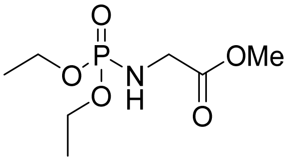

15a

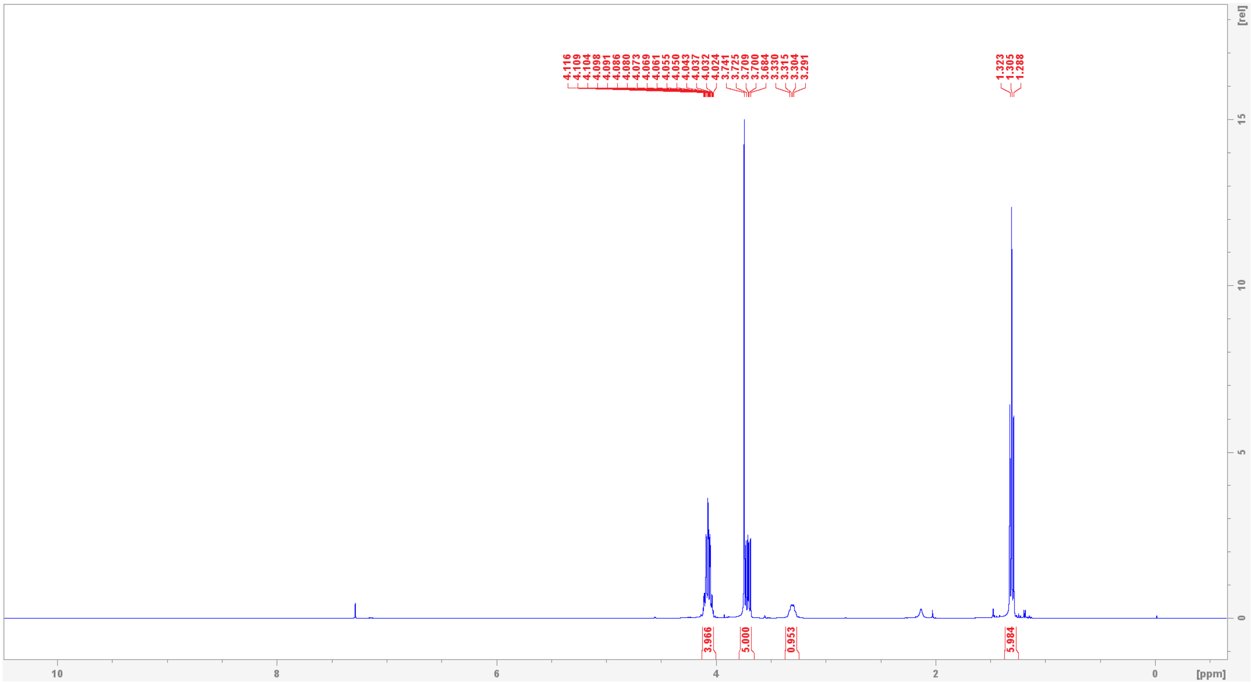

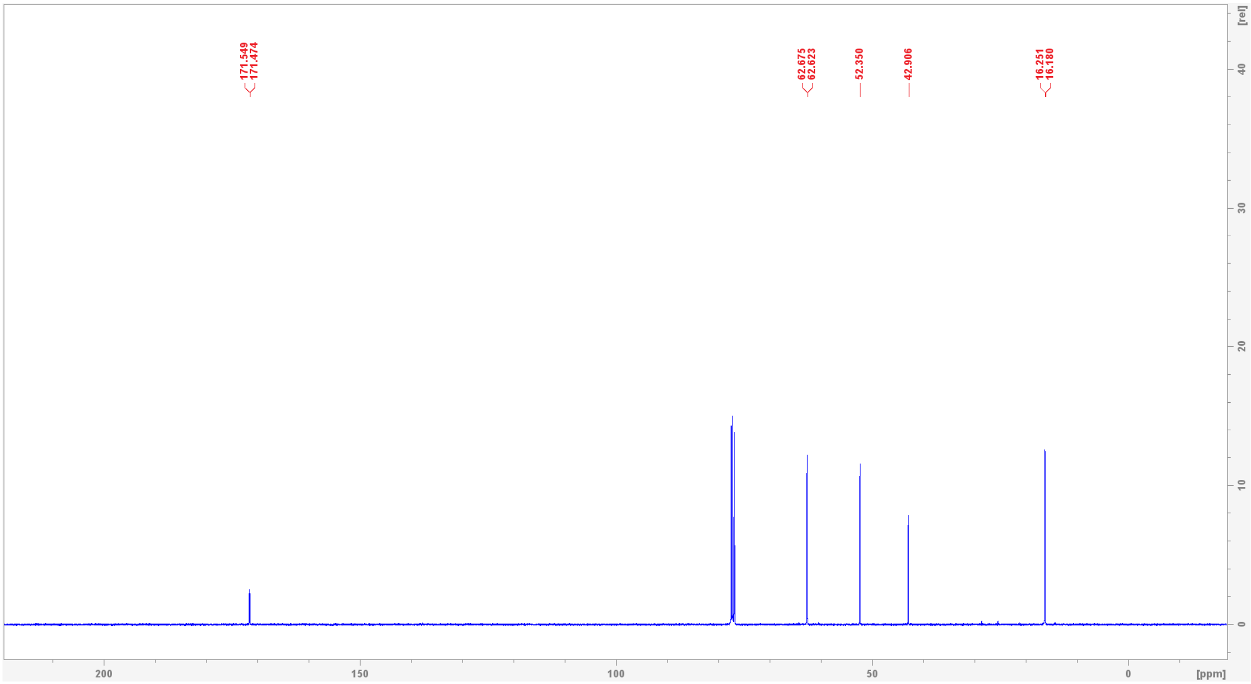

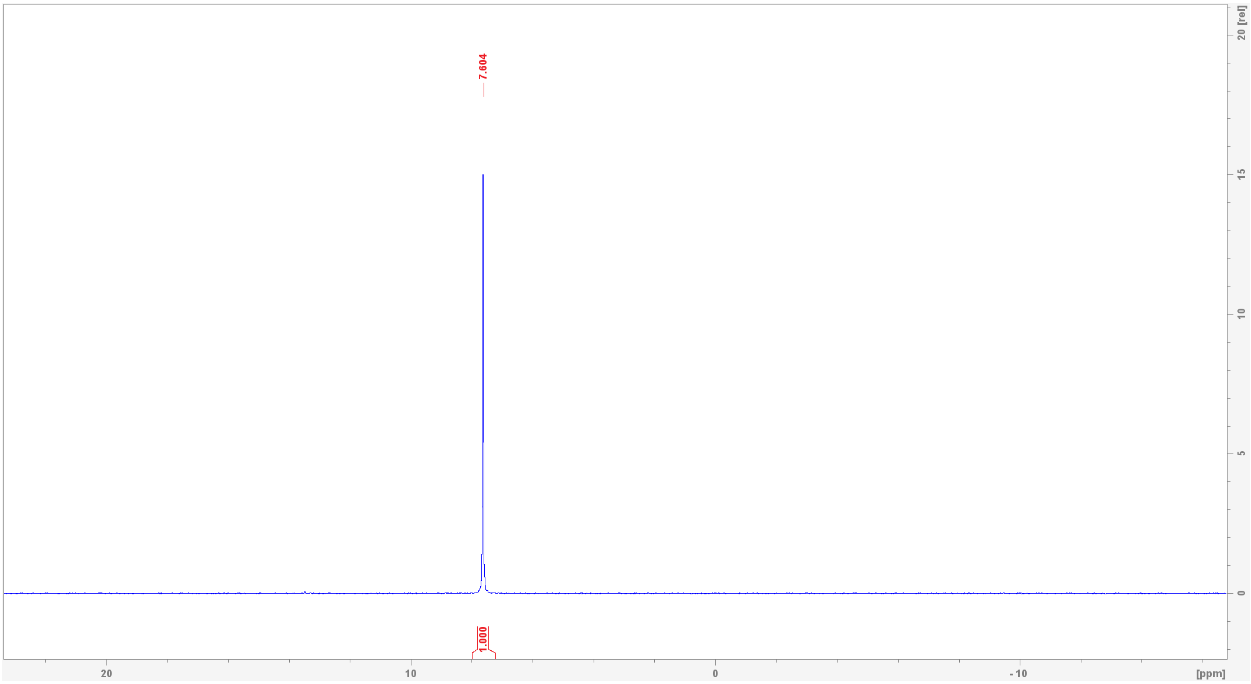

7.604
1.000
20
10
0
- 10
[ppm]
20 [rel]
15
10
5
0

Compound **15b**: **^{1}H NMR** (400 MHz, $CDCl_3$), **^{13}C NMR** (100.6 MHz, $CDCl_3$) and **^{31}P NMR** (161.9 MHz, $CDCl_3$).

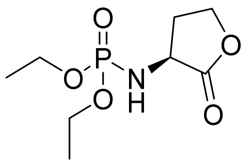

15b

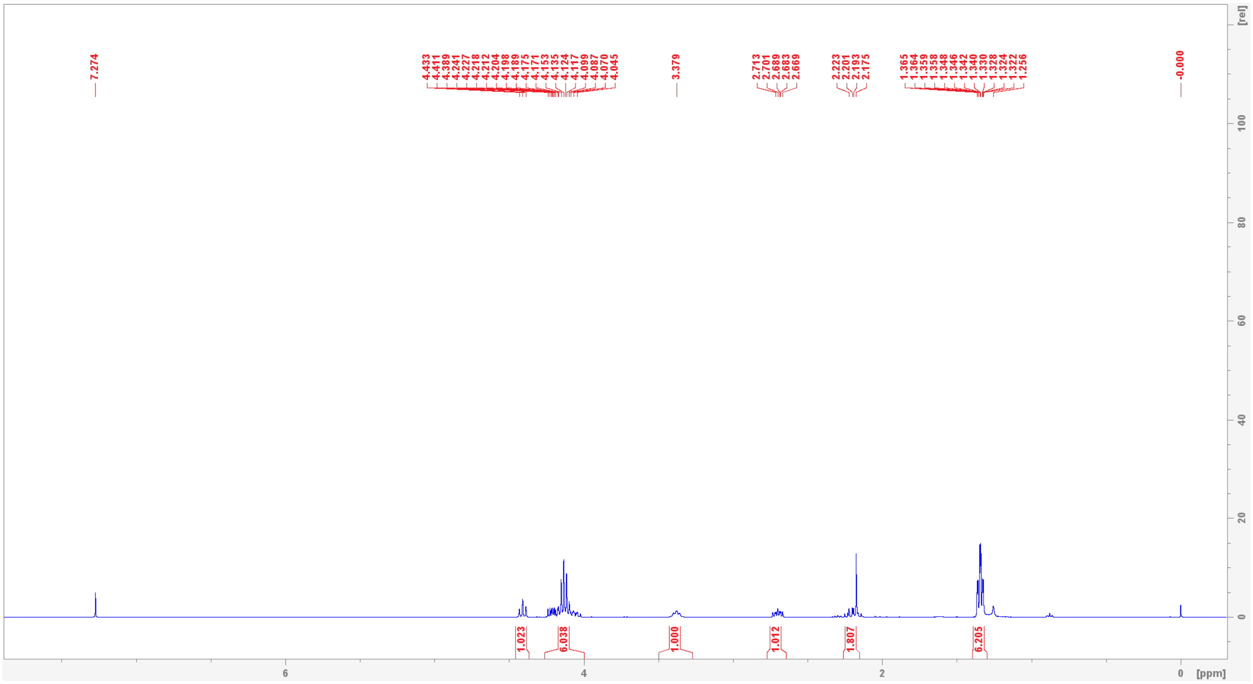

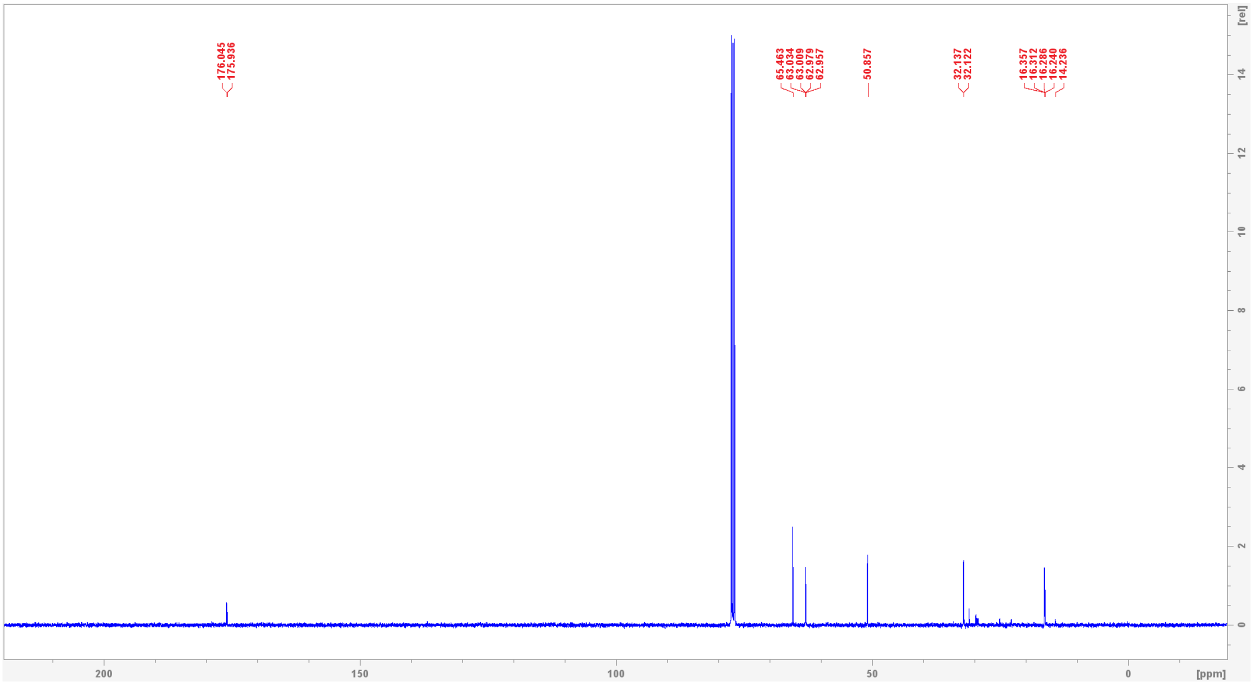

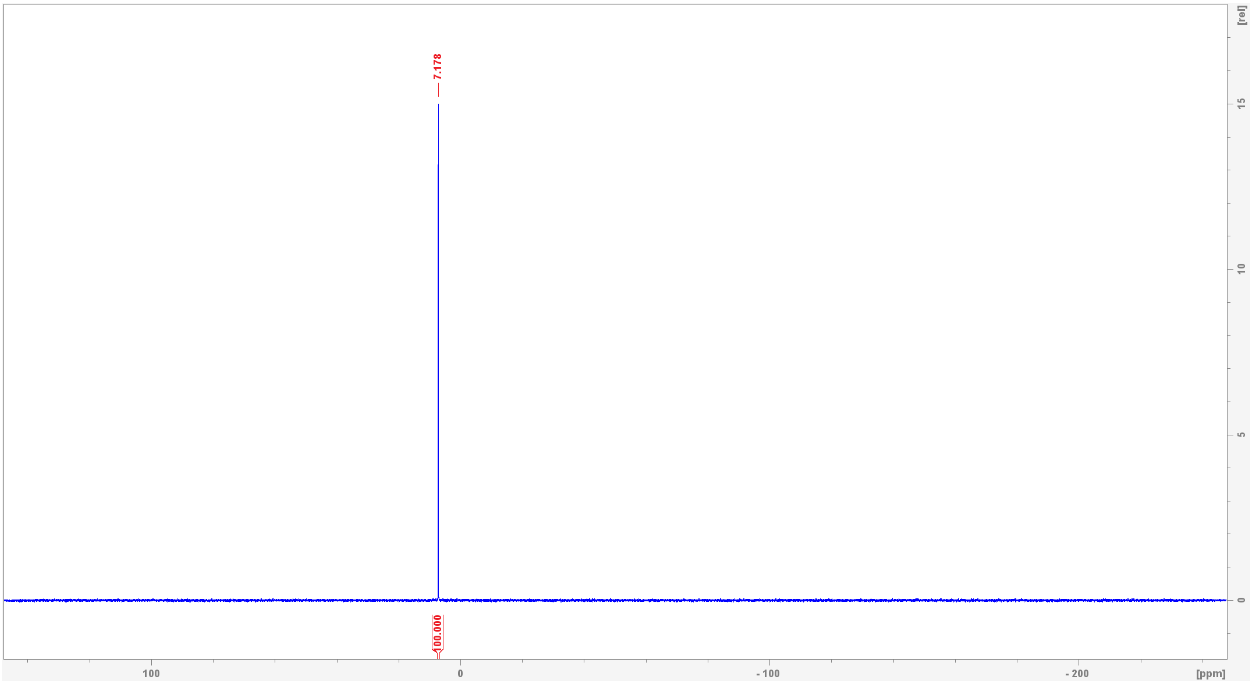

7.178
100.000
100
0
- 100
- 200
[ppm]
[rel]
0
5
10
15

Compound **15c**: **^{1}H NMR** (400 MHz, $CDCl_3$), **^{13}C NMR** (100.6 MHz, $CDCl_3$) and **^{31}P NMR** (161.9 MHz, $CDCl_3$).

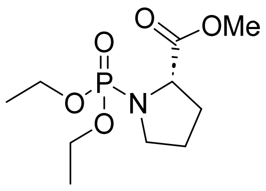

15c

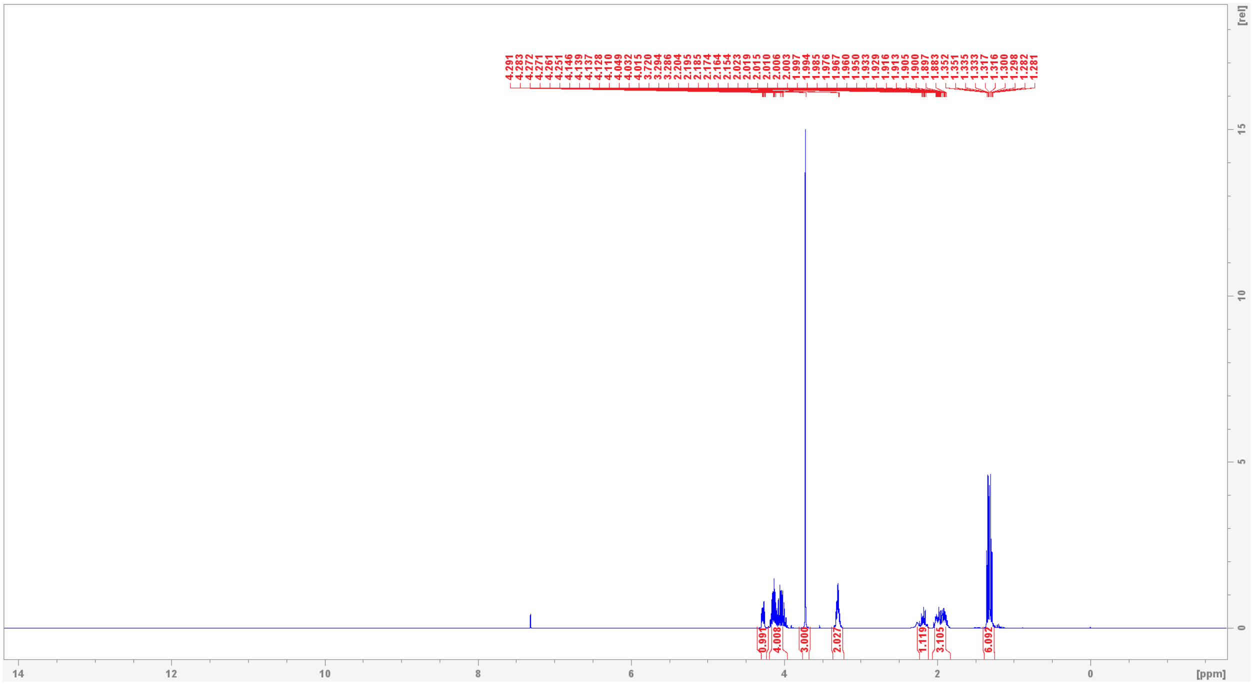

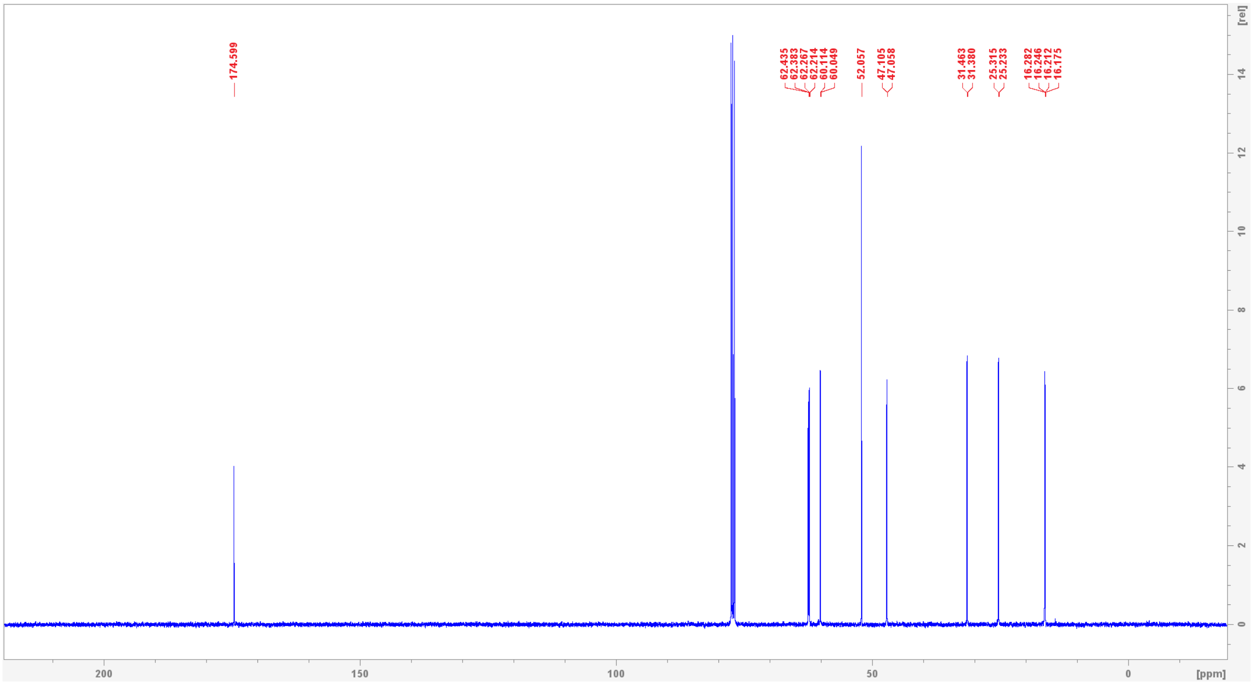

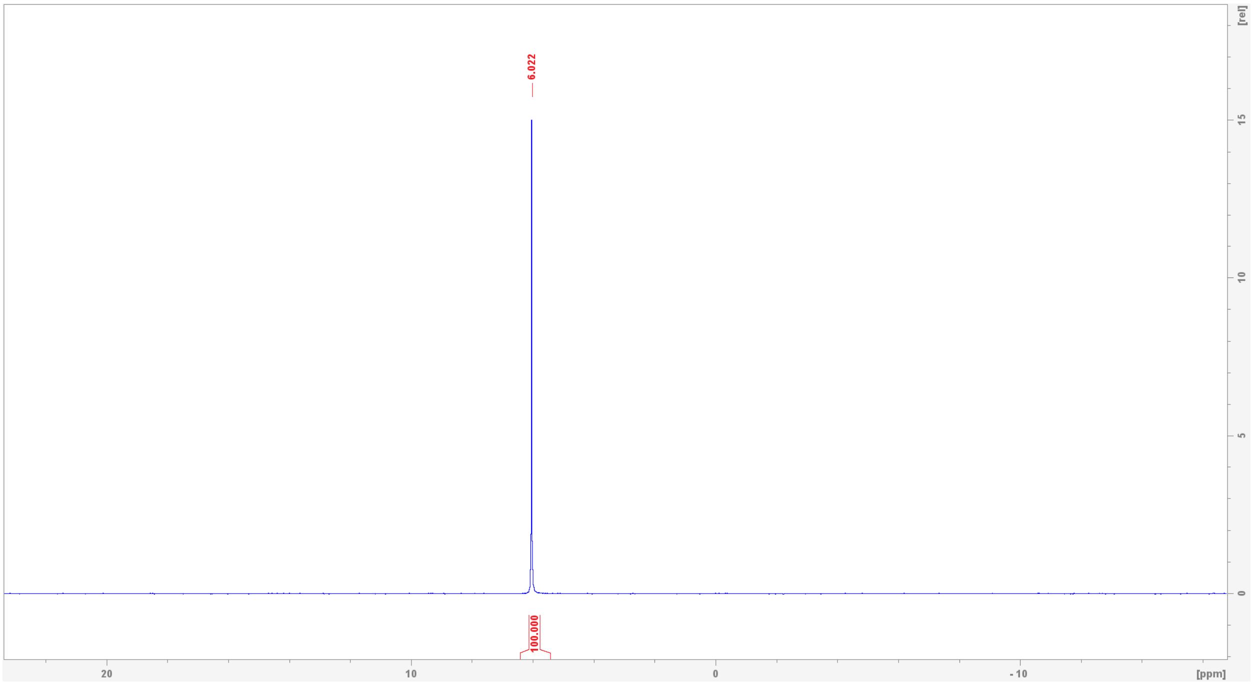

6.022
100.000
20
10
0
-10
[ppm]
[rel]
15
10
5
0

Compound **15d**: **^{1}H NMR** (400 MHz, $CDCl_3$), **^{13}C NMR** (100.6 MHz, $CDCl_3$) and **^{31}P NMR** (161.9 MHz, $CDCl_3$).

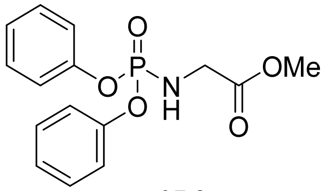

15d

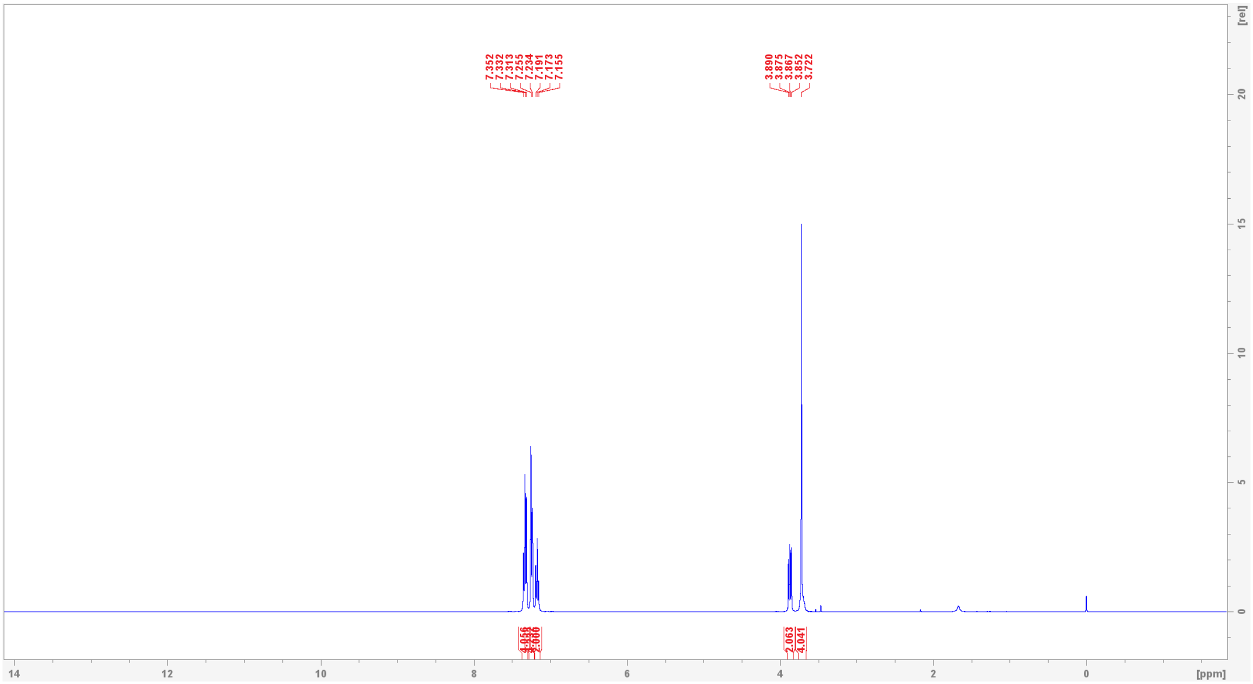

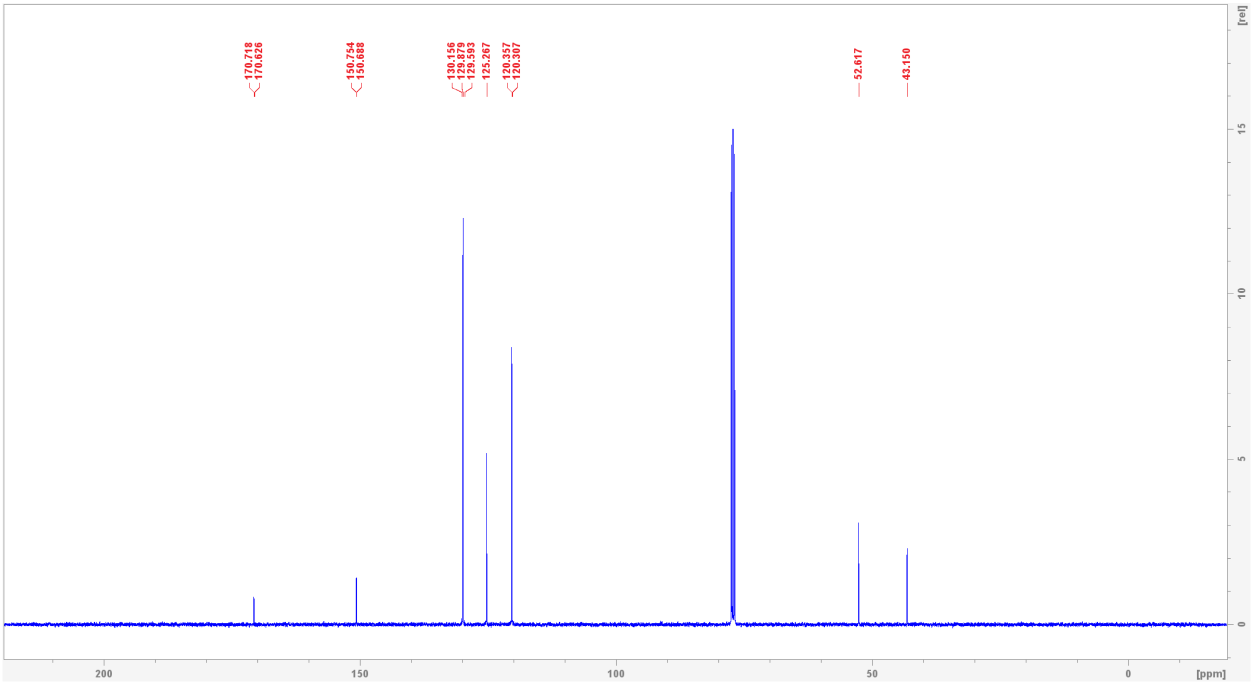

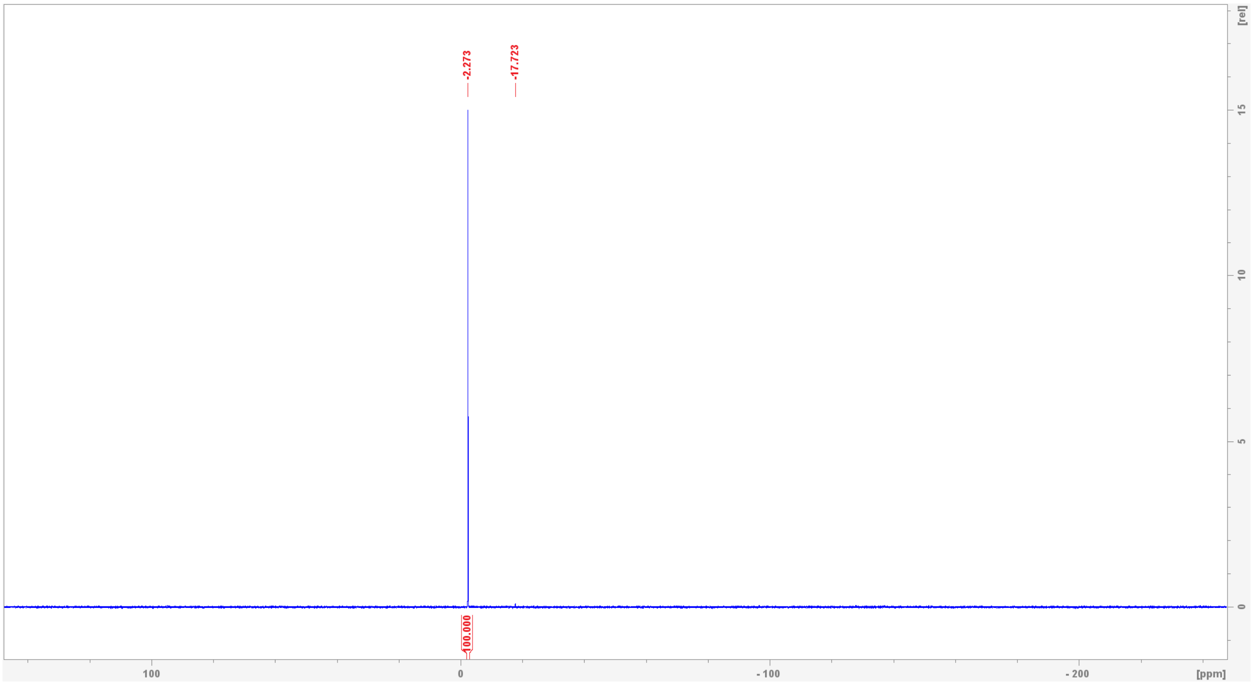

-2.273
-17.723
100.000
[rel]
15
10
5
0
100
0
- 100
- 200
[ppm]
